# Supplementary material for: B cells sustain inflammation and predict response to immune checkpoint blockade in human melanoma
Source: Nat Commun. 2019 Sep 13;10:4186. doi: 10.1038/s41467-019-12160-2 (PMC6744450; doi:10.1038/s41467-019-12160-2)
Supplement: Supplementary file 1 — Supplementary Information [file 41467_2019_12160_MOESM1_ESM.pdf]

B cells sustain inflammation and predict response to immune checkpoint blockade in human melanoma - Supplementary Materials

Griss *et al.*

Table of Contents

Table of Contents 1

Supplementary Figures 2

References 12

Supplementary Figures

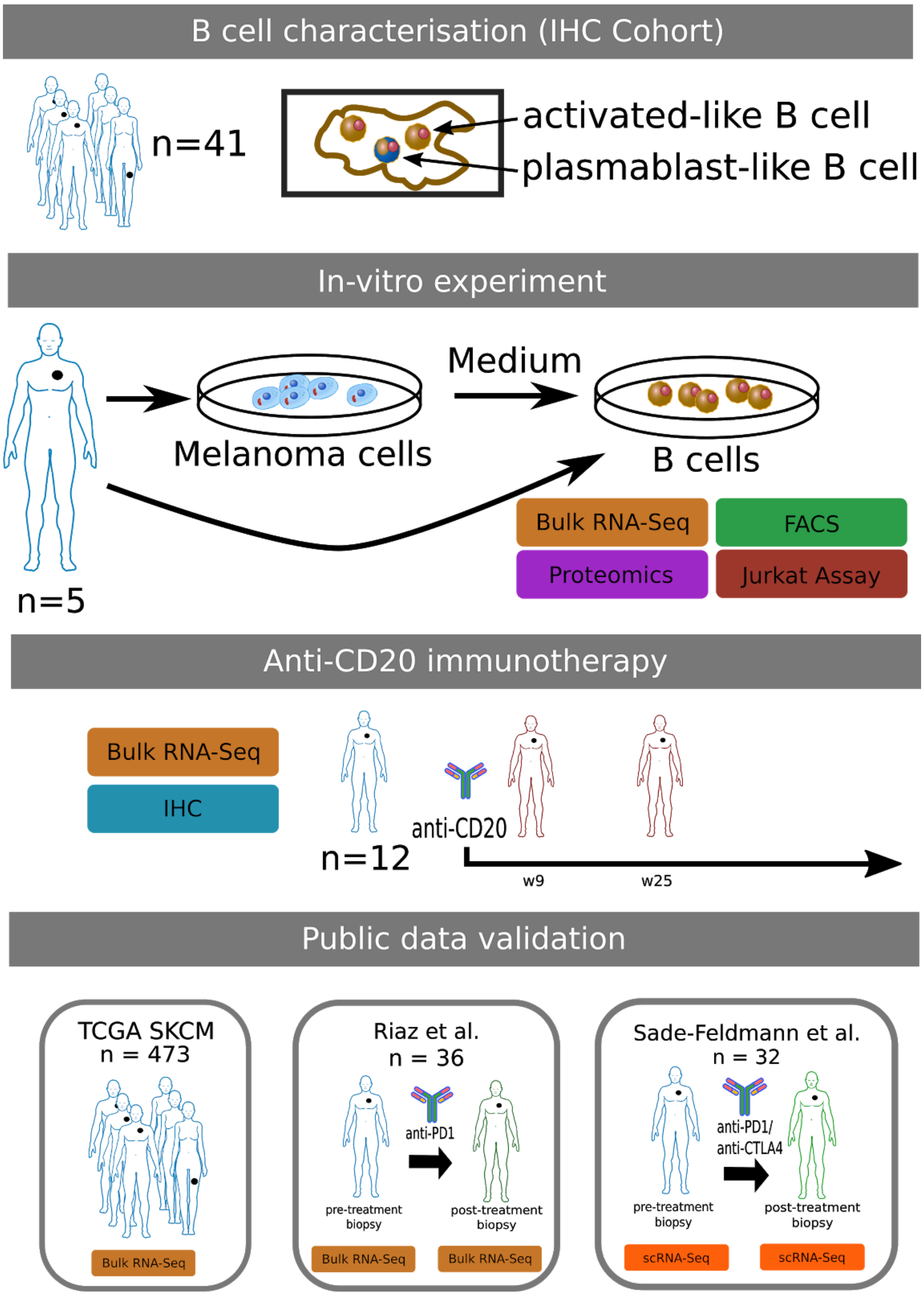

Supplementary Figure 1: Patient cohorts evaluated in this study. Melanoma TAB phenotypes were characterised by multiplex immunostaining of whole tumor sections from 41 patients. B cell changes through melanoma secretomes were analysed in PBMC-and tumor-derived B cells from 5 patients and conditioned media from autologous melanoma cells. B cell-induced changes in the melanoma TME were analyzed in clinical samples from anti-CD20 immunotherapy trials performed by our group. Finally, all findings were validated using 3 public datasets: the TCGA skin cutaneous melanoma cohort, whole tissue

RNA-seq data from melanoma patients pre- and on-anti-PD1 therapy and scRNA-seq data from melanoma lesions from two independent studies. The icons of the cells, man and woman were adapted from icons of the Reactome icon library created by CSHL, OICR and EBI (https://reactome.org/icon-lib)[1].

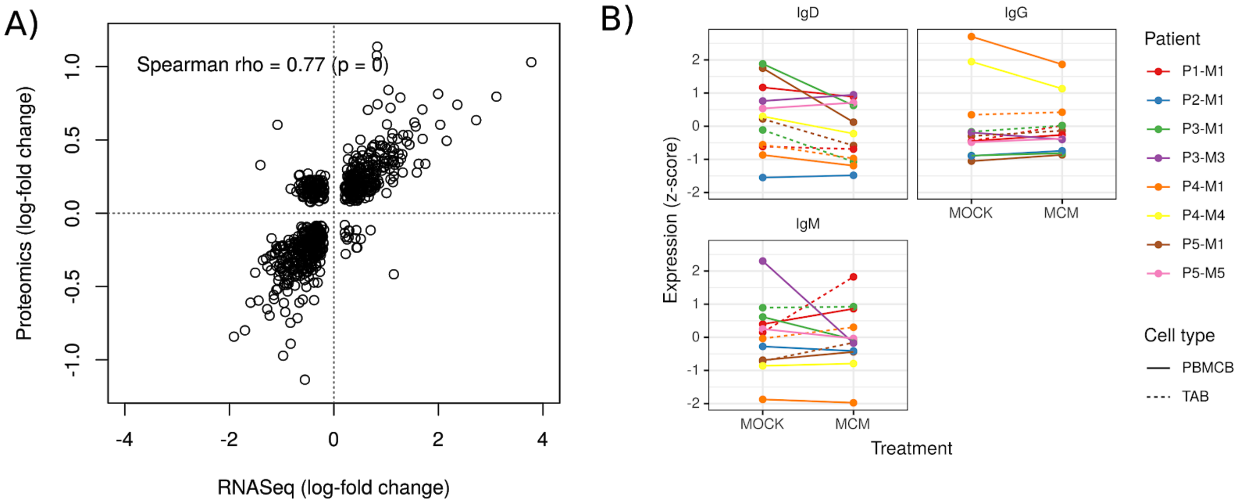

Supplementary Figure 2: Induction experiments, significantly regulated gene/proteins and FACS data in B cells induced with melanoma secretomes. A) Fold changes (log2 transformed) of genes/proteins identified as significantly regulated showed a high correlation between proteomics and RNA-seq results. One group of proteins was consistently upregulated while the corresponding transcripts were downregulated (top-left corner). These all belong to proteins associated with metabolic pathways. **B)** Additional FACS-estimated expression levels of IgD, IgG, and IgM on peripheral blood B cells (PBMCB, solid line)- and tumour -derived B cells (TAB, dashed line) by melanoma conditioned (MCM) and control (MOCK) medium. Patient and medium combination are shown as different colors.

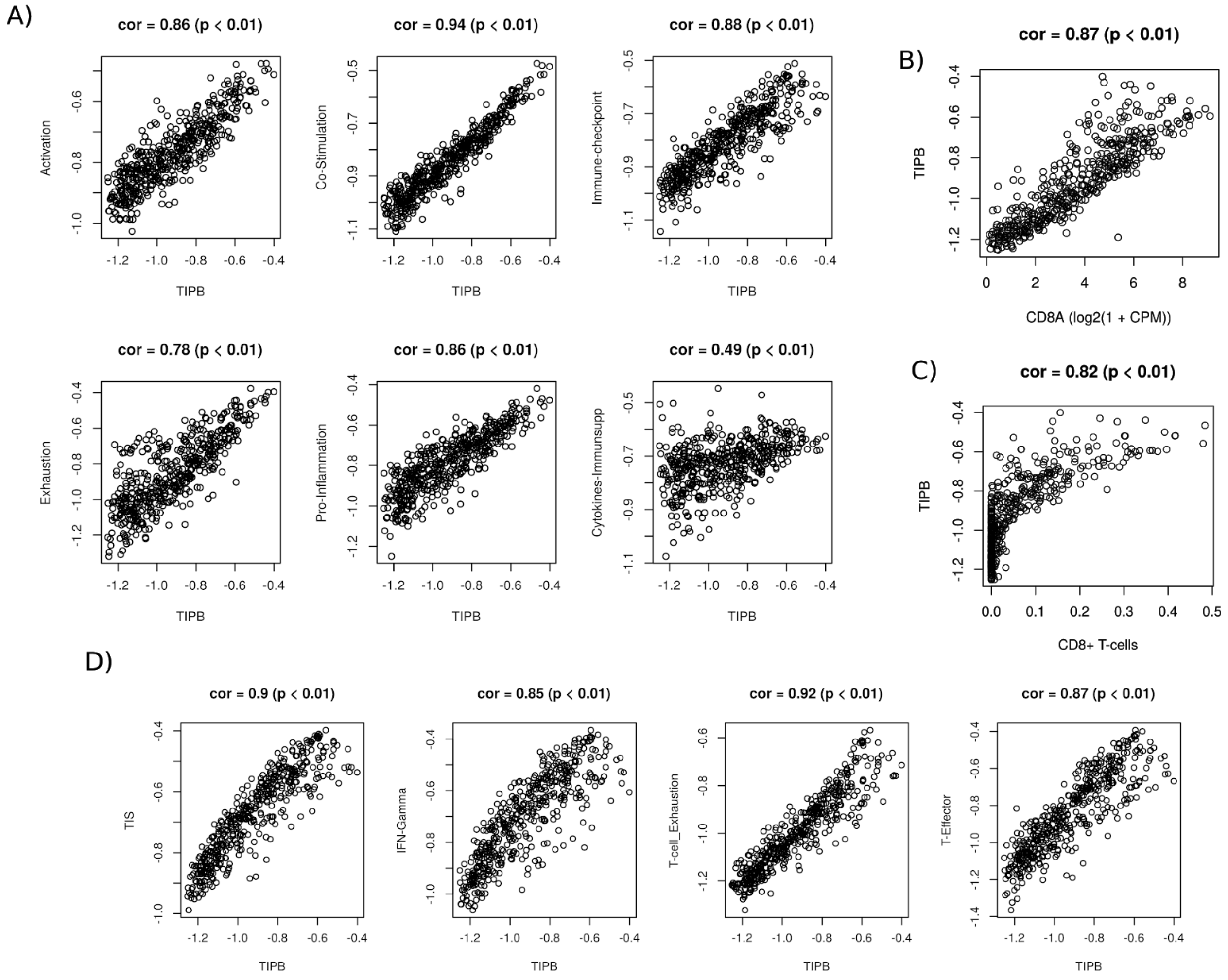

Supplementary Figure 3: Validation of the TIPB and the functional signatures in the TCGA skin cutaneous melanoma cohort. A) Correlation of the functional signatures with the TIPB signature. **B)** Correlation of the TIPB signature with the expression of CD8A and **C)** with the xCell estimated abundance of $CD8^{+}$ T-cells. **D)** Established signatures describing inflammation in the TME and T cell function and phenotype (tumor inflammatory score (TIS), interferon (IFN) gamma, T cell exhaustion, T cell effector (T-effector)) highly correlated with our TIPB signature. All correlation coefficients and p-values refer to the Spearman correlation coefficient.

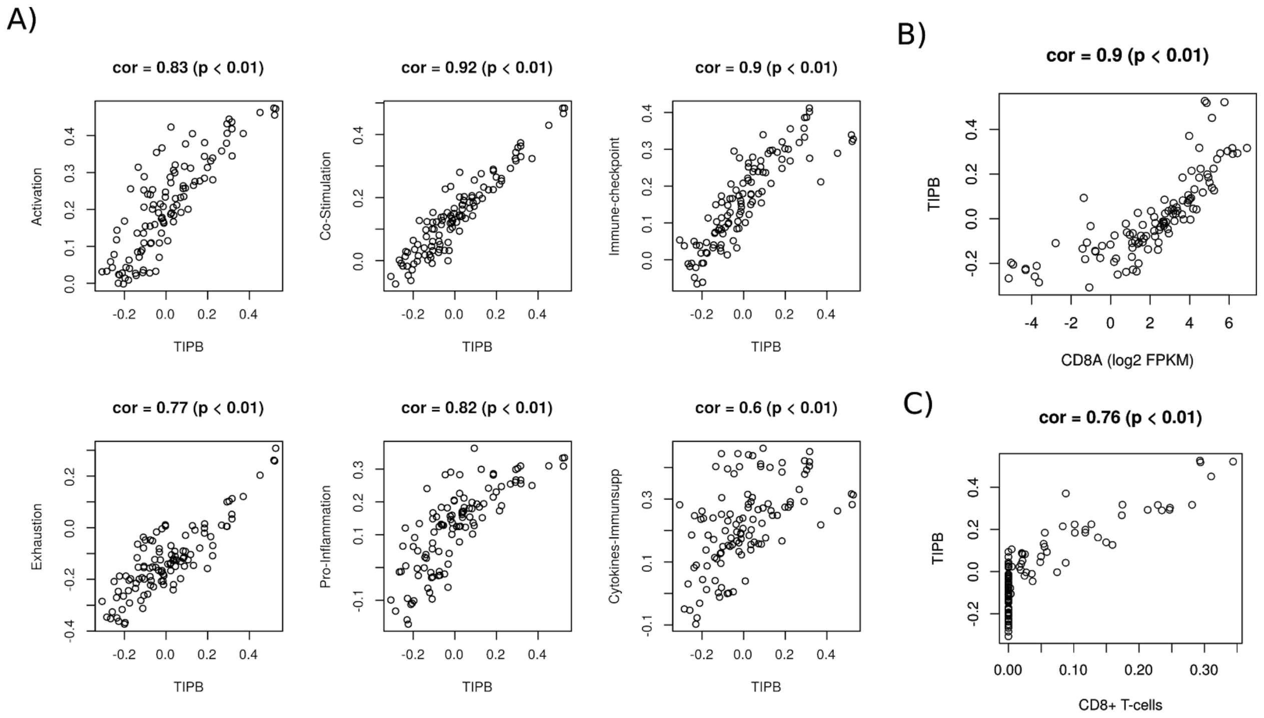

Supplementary Figure 4: Validation of the TIPB and the functional signatures in a melanoma cohort treated with anti-PD1 (Riaz et al. dataset). A) Correlation of functional signatures as estimated by ssGSEA with the TIPB signature. **B)** Correlation of the TIPB signature with the expression of CD8A and **C)** the xCell estimated abundance of $CD8^+$ T cells.

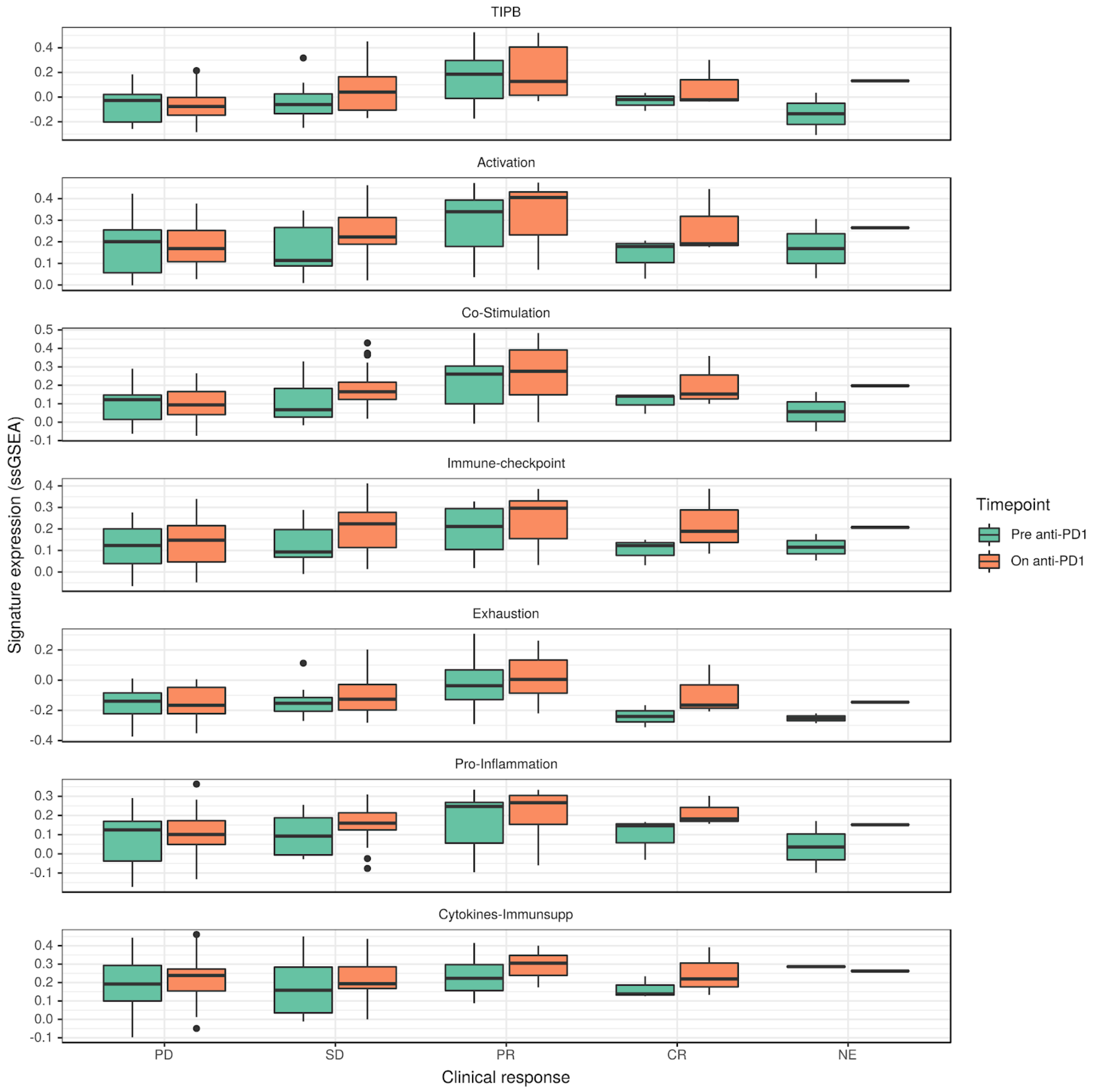

Supplementary Figure 5: Expression of the TIPB signature and the functional signatures before (green) and on (orange) anti-PD1 therapy versus clinical response (Riaz *et al.* dataset). (PD = progressive disease, SD = stable disease, PR = partial response, CR = complete response, NE = not evaluated). Lower and upper hinges correspond to the first and third quartiles, center line to the median. Upper whisker extends from the hinge to the largest value no further than 1.5 times the interquartile range. Values outside this range are shown as outliers.

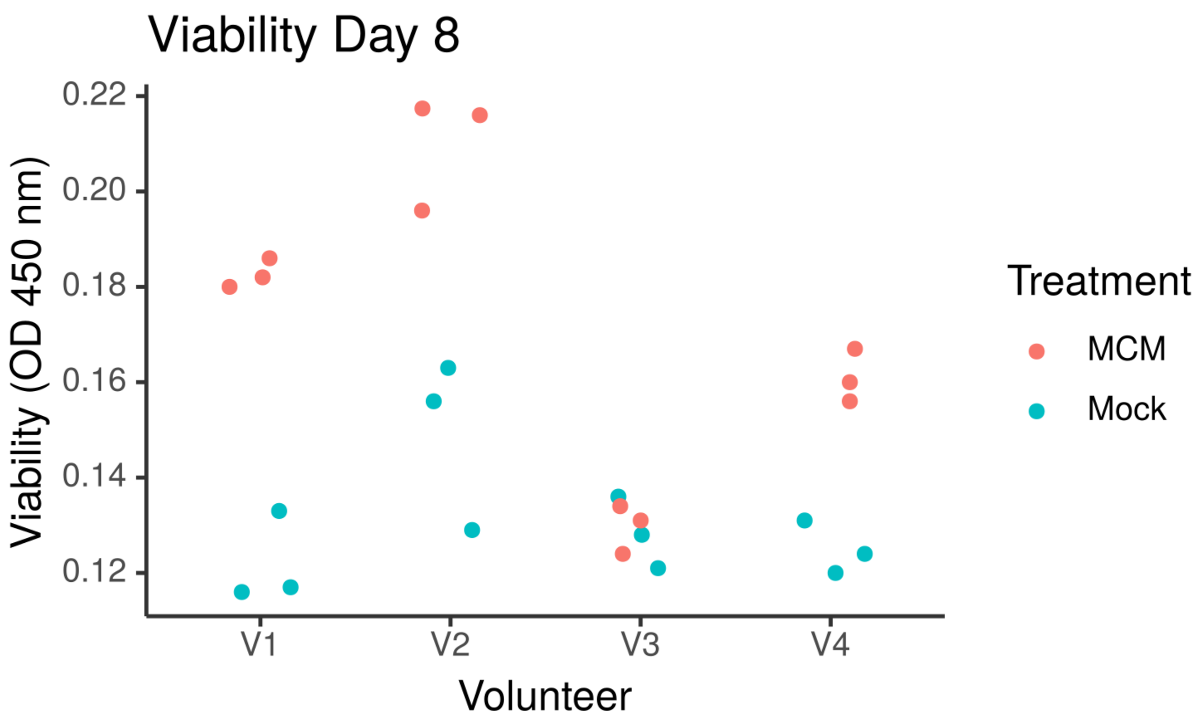

Supplementary Figure 6: Viability of MCM (red) and control (blue) medium conditioned B cells from four healthy volunteers. MCM led to a significant increase in B cell viability at day 8 (paired t-test, $p < 0.01$, $t = -4.8$, $df = 11$).

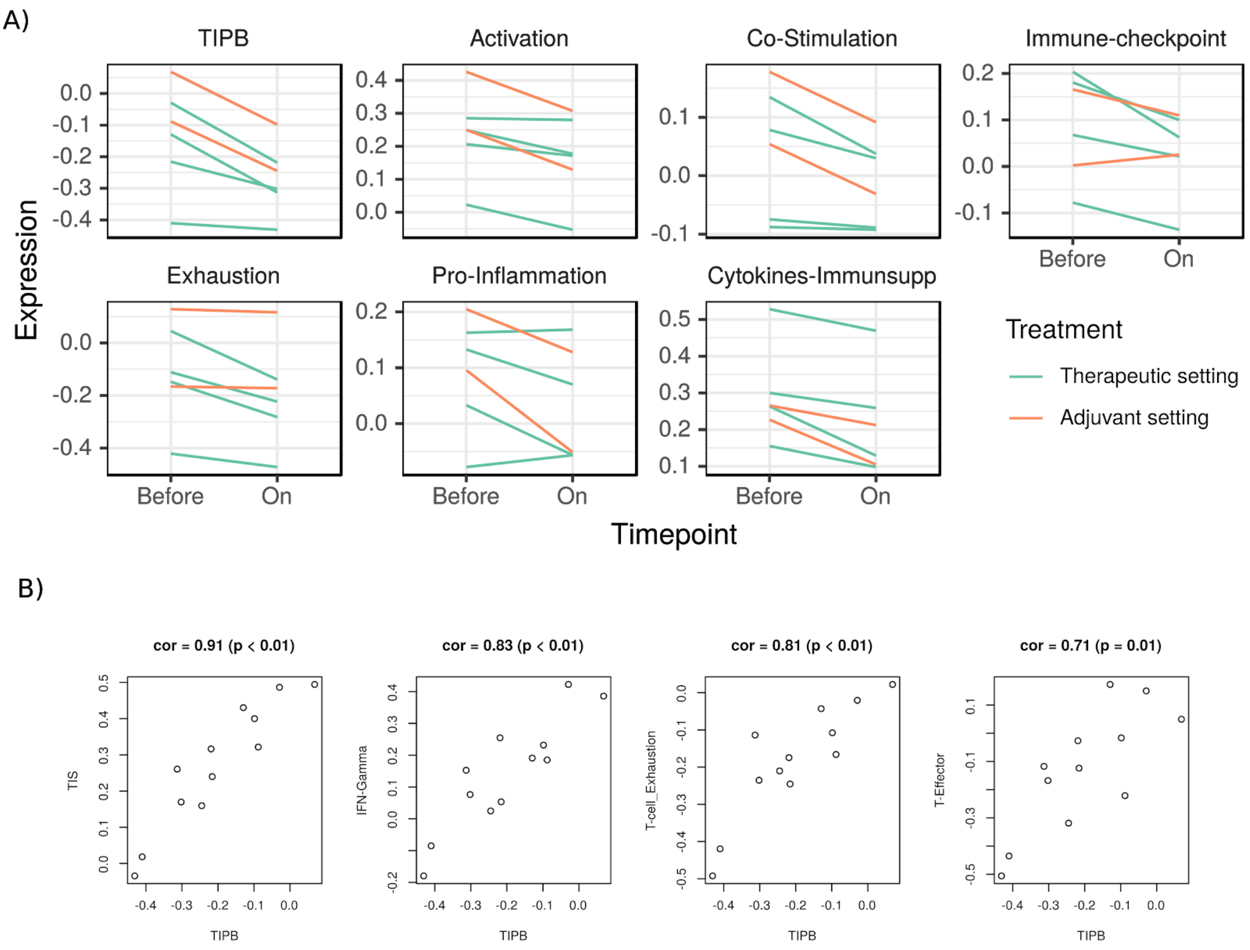

Supplementary Figure 7: Validation of the TIPB and the functional signatures in the anti-CD20 clinical study samples. A) Estimated abundance (ssGSEA) of the TIPB signature and all functional signatures before and on anti-CD20 therapy. Colors represent the two different anti-CD20 studies. Each line rerpesents one patient. B) Correlation of established inflammation and T cell gene signatures with our TIPB signature.

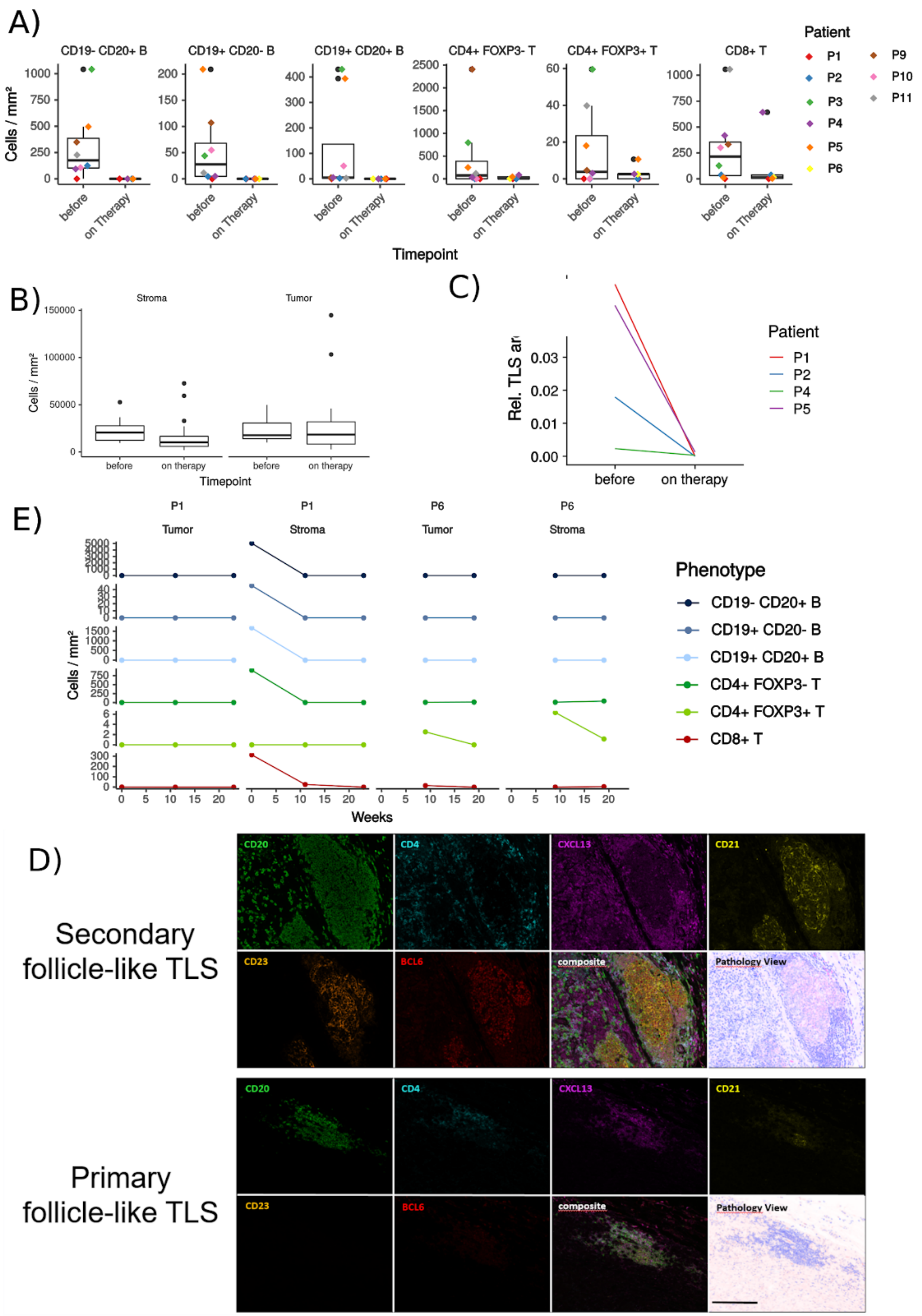

Supplementary Figure 8: Quantification of immune cells using multiplex immunostainings. A) Intratumoral B and T cell numbers quantified using 6 color multiplex immunostaining in tumor samples from 9 patients obtained before and on therapy (at week 9±2). Values per patient are shown as colored

diamonds. **B)** Total cell numbers as quantified by multiplex immunostainings of samples before and on therapy (at week 9±2). Cells were separately quantified for the extratumoral stroma ("Stroma") and intratumorally ("Tumor"). **C)** Relative area occupied by tertiary lymphoid structures (TLS) within metastases before and on anti-CD20 treatment as detected by 7 color multiplex immunostaining. **D)** Examples for both secondary mature and primary follicle-like TLS. Images for each of the individual markers and their composites are shown (for clarity without DAPI nuclear staining), together with the corresponding pathology view (respective bottom right). Note lack of CD23 and Bcl6 immunoreactivity in primary follicle-like TLS. **E)** B and T cells quantified in two patients intratumorally ("Tumor") and at the extratumoral stroma ("Stroma") at two time points on therapy. Different color represent different T and B cell types. Scale bar represents 400 µm.

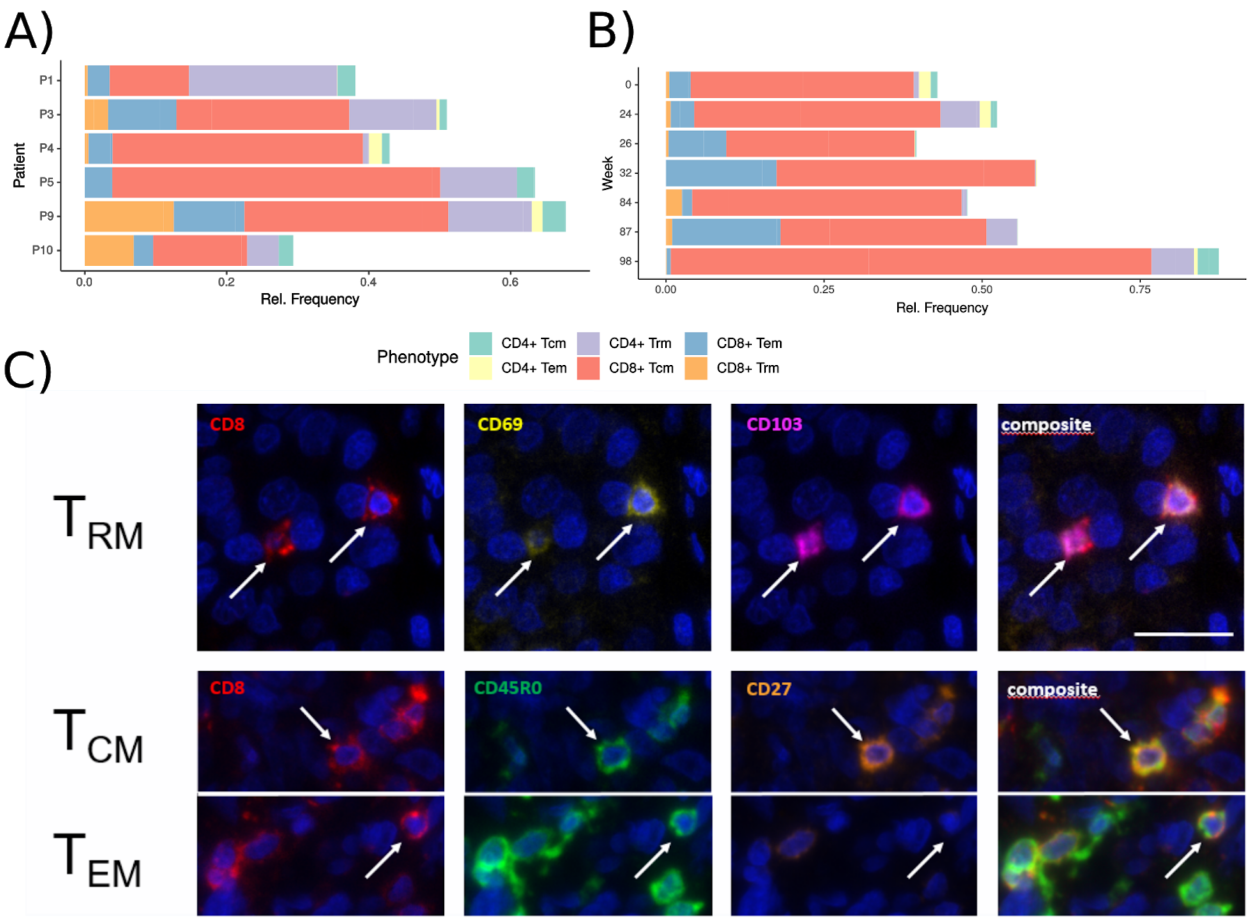

Supplementary Figure 9: Quantification of T cell subtypes using multiplex immunostainings. A,B) Tissue-resident (T_{RM}), central (T_{CM}) and effector memory(T_{EM}) T cell subtypes quantified in samples from 6 patients before therapy (A) and a longitudinal analysis of tumor samples obtained over nearly two years (whenever a T cell infiltrate was present) in patient 4 (B). For corresponding therapy information see Figure 6B. C) Examples for $CD8^{+}$ tissue-resident (T_{RM}), central (T_{CM}) and effector memory(T_{EM}) T cell subtypes from 7 color multiplex immunostaining. Composite images together with DAPI nuclear staining (right) and images for each of the individual markers used. Arrows depict representative cells. Scale bar represents 40µm.

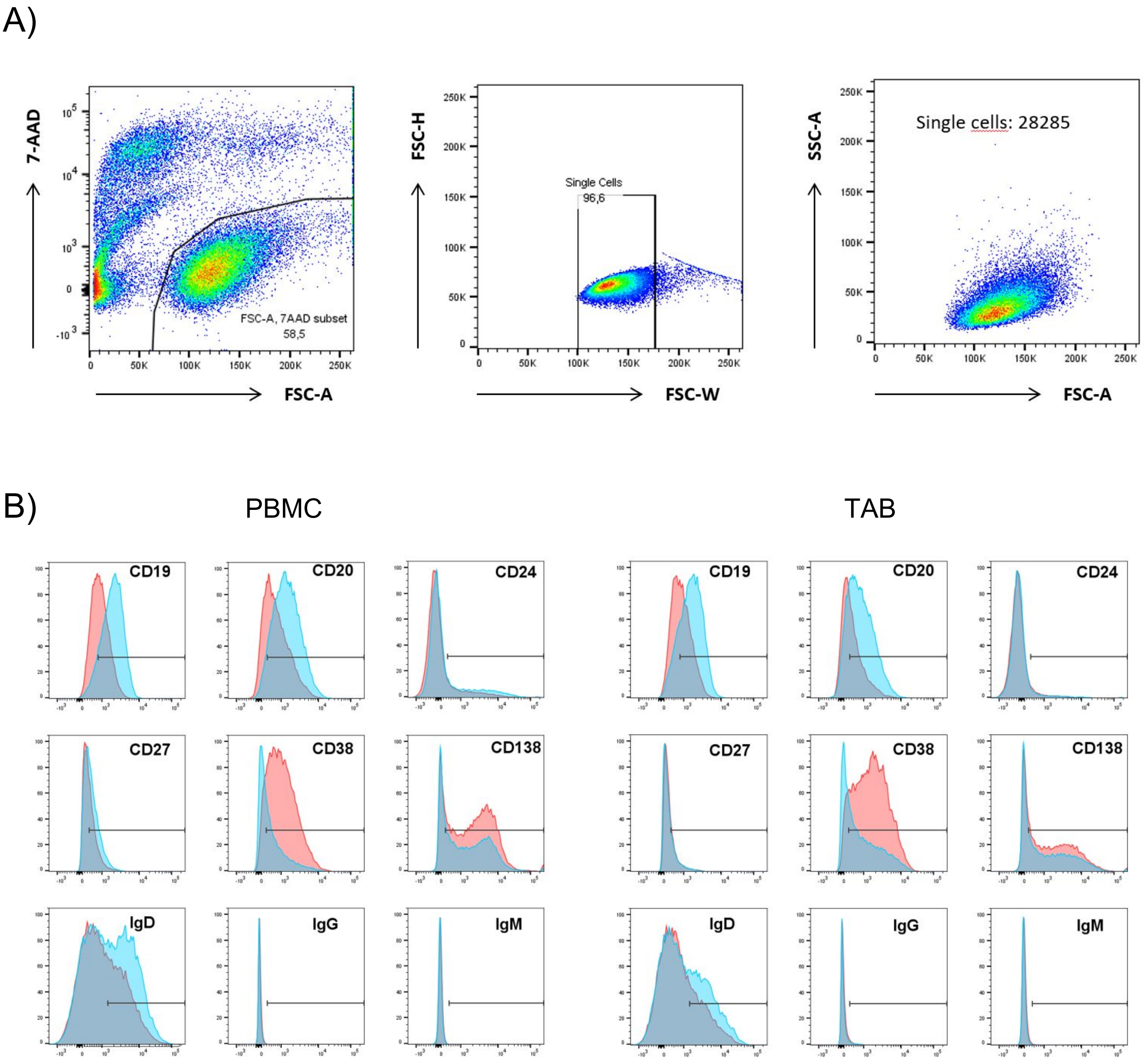

Supplementary Figure 10: FACS Gating Strategy. A) FACS staining of immortalized peripheral blood- and tumor-derived B cells: gating strategy for analysis of viable, single cells. B) Representative FACS stainings of peripheral blood-derived immortalized B cells (PBMC) and tumor-derived B cells (TAB) of one patient induced with control medium (blue histograms) or melanoma-conditioned medium (red histograms) for 48 hours. The gate represents the isotype-defined positivity of the stainings.

References

1. Sidiropoulos, K. *et al.* Reactome enhanced pathway visualization. *Bioinformatics* **33**, 3461–3467 (2017).
