# Supplementary material for: B cells sustain inflammation and predict response to immune checkpoint blockade in human melanoma
Source: Nat Commun. 2019 Sep 13;10:4186. doi: 10.1038/s41467-019-12160-2 (PMC6744450; doi:10.1038/s41467-019-12160-2)
Supplement: Supplementary file 3 — Description of Additional Supplementary Files [file 41467_2019_12160_MOESM3_ESM.pdf]

Description of Additional Supplementary Files

File Name: Supplementary Data 1
Description: Clinical metadata of all patients included in the study.

File Name: Supplementary Data 2
Description: RNA-seq, proteomics, phospho-proteomics, and combined pathway analysis results for the MCM induction experiments. Respective tables contain the list of significantly regulated genes, proteins, and pathways respectively.

File Name: Supplementary Data 3
Description: Average gene expression of all detected genes across the different B cell subtypes in the scRNA-seq dataset from Sade-Feldmann et al

File Name: Supplementary Data 4
Description: List of significantly regulated genes (edgeR FDR <= 5%) before versus on anti-CD20 therapy in whole tissue samples of the patients in the anti-CD20 trials.

Supplementary Software 1
Description: Jupyter notebooks used to create the statistical a`nalyses.
