# Supplementary material for: B cells sustain inflammation and predict response to immune checkpoint blockade in human melanoma
Source: Nat Commun. 2019 Sep 13;10:4186. doi: 10.1038/s41467-019-12160-2 (PMC6744450; doi:10.1038/s41467-019-12160-2)
Supplement: Supplementary file 8 — Supplementary Software 1 [file 41467_2019_12160_MOESM8_ESM.zip › 193518_1_other_1_1560249387.pdf]

B cells sustain inflammation and predict response to immune checkpoint blockade in human melanoma

Johannes Griss[1,2,*], Wolfgang Bauer[1], Christine Wagner[1], Martin Simon[1], Minyi Chen[1], Katharina Grabmeier-Pfistershammer[1,3], Margarita Maurer-Granofszky[1§], Florian Roka[1], Thomas Penz[4], Christoph Bock[4,5], Gao Zhang[6,#], Meenhard Herlyn[6], Katharina Glatz[7], Heinz Läubli[8], Kirsten D Mertz[9], Peter Petzelbauer[1], Thomas Wiesner[1], Markus Hartl[10], Winfried F Pickl[11], Rajasekharan Somasundaram[6], Peter Steinberger[3], Stephan N Wagner[1*]

1 Department of Dermatology, Medical University of Vienna, 1090 Vienna, Austria
2 EMBL-European Bioinformatics Institute, Wellcome Trust Genome Campus, CB10 1SD Hinxton, Cambridge, United Kingdom
3 Division of Immune Receptors and T-cell Activation, Institute of Immunology, Center for Pathophysiology, Infectiology and Immunology, Medical University of Vienna
4 CeMM Research Center for Molecular Medicine of the Austrian Academy of Sciences, 1090 Vienna, Austria
5 Department of Laboratory Medicine, Medical University of Vienna, 1090 Vienna, Austria
6 Molecular & Cellular Oncogenesis Program and Melanoma Research Center, The Wistar Institute, Philadelphia, PA 19104-4265, USA
7 Institute of Pathology, University Hospital Basel, Basel, Switzerland, 4031
8 Division of Medical Oncology, University Hospital Basel, Basel, Switzerland, 4031
9 Institute of Pathology, Cantonal Hospital Baselland, Liestal, Switzerland, 4410
10 Mass Spectrometry Facility, Max F. Perutz Laboratories (MFPL), University of Vienna, Vienna BioCenter (VBC), 1030 Vienna, Austria
11 Division of Cellular Immunology and Immunohematology, Institute of Immunology, Center for Pathophysiology, Infectiology and Immunology, Medical University of Vienna
# Present address: Department of Neurosurgery & The Preston Robert Tisch Brain Tumor Center, Duke University Medical Center, Durham, NC 27710, USA
§ Present address: Children's Cancer Research Institute, 1090 Vienna, Austria

* Corresponding Authors:

Johannes Griss, johannes.griss@meduniwien.ac.at, Tel: +43 1 40400 77020

Stephan N. Wagner, stephan.wagner@meduniwien.ac.at, Tel: +43 1 40400 77020

Abstract

Tumor associated inflammation predicts response to immune checkpoint blockade in human melanoma. Established mechanisms that underlie therapy response and resistance center on anti-tumor T cell responses.

Here we show that tumor associated B cells are vital to melanoma associated inflammation. Autologous B cells which we exposed to melanoma conditioned medium expressed pro- and anti-inflammatory factors, and differentiated towards a plasmablast-like phenotype *in vitro*. In human melanomas this phenotype could be reconciled by multiplex immunostaining and independent single-cell RNA-seq data. There, this phenotype expressed T cell-recruiting chemokines CCL3, CCL4, CCL5. Depletion of B cells in melanoma patients by anti-CD20 immunotherapy decreased tumor associated inflammation and $CD8^+$ T cell numbers. Plasmablast-like B cells also increased activation of $PD\text{-}1^+$ T cells through anti-PD-1 blockade *in vitro* and their frequency in pretherapy melanomas predicted response and survival to immune checkpoint blockade in two independent patient cohorts. Tumor associated B cells therefore orchestrate and sustain melanoma inflammation, recruit $CD8^+$ effector T cells and may represent a predictor for survival and response to immune checkpoint blockade.

Cancers such as melanoma, lung, and kidney cancer often present with an inflamed but immunosuppressed tumor microenvironment (TME). Immune checkpoint blocking (ICB) antibodies have significantly improved cancer therapy by overcoming inhibition of T cell effector functions. Yet, a considerable number of patients does not benefit from ICB therapy[1]. It is therefore key to understand the mechanisms that regulate inflammation within the TME to develop novel therapies and improve patient survival.

Existing data on the role of tumor associated B cells (TAB) is inconsistent. Mouse cancer models show that TAB promote tumor inflammation[2,3] but may also inhibit anti-tumor T cell-dependent therapy responses[4–7]. The immuno-inhibitory function of TAB in the latter models resembles that of regulatory B cells (Breg), which are an established source of inhibitory cytokines such as IL-10 and TGF-b (reviewed in[8]). In human cancer, Breg frequencies commonly increase with tumor progression and are enriched in tumors compared to peripheral blood or adjacent normal tissue. Increased IL-10^{+} B cell numbers in tumor tissues can also be accompanied by increased numbers of CD4^{+}CD25$^{+/high}$CD127$^{low/-}$ and Foxp3^{+} Tregs[9–12], which are independently associated with tumor progression or reduced patient survival.

In human melanoma, up to 33% of the immune cells can be TAB[13,14]. Phenotypic analyses show the presence of CD20^{+} TAB (reviewed in[15]) and CD138^{+} or IgA^{+}CD138^{+} plasma cells[14,16]. Conclusions about their impact on disease progression and outcome are, however, inconsistent. Similarly, *in vivo* melanoma transplantation models revealed pro- as well as anti-tumorigenic effects of B cell-deficiency or -depletion in syngeneic mice (reviewed in[17]). In addition, advanced models of human melanoma such as genetically engineered mouse models fail to adequately picture tumor infiltration by TAB[18] and patient-derived xenografts in

immune-compromised mice fail to develop a relevant TME. Thus, conclusions about the function of TAB in melanoma can only be drawn from studies in human so far.

In vitro, human melanoma cells provide antigens for prolonged B cell receptor stimulation and release inflammation- and B cell-modulating cytokines such as IL-1b and IL-6[19] as well as IL-35[20]. While anti-inflammatory IL-35 promotes the induction of Breg functions[21], pro-inflammatory IL-1b and IL-6 have also been shown to indirectly or directly promote activation and differentiation of B cells[22,23]. We recently showed that human melanoma cells release FGF-2 which induces TAB to secrete IGF-1, a source of acquired drug resistance of melanoma cells to mitogen-activated protein kinase (MAPK) inhibitors[13]. Consistently, clinical data from our pilot trial and an independent case series indicate objective tumor responses and clinical benefit through B cell-depletion by anti-CD20 antibodies in end-stage, even MAPK inhibitor resistant melanoma patients[13,24].

As MAPK inhibitor resistance frequently co-evolves with fundamental TME changes in inflammation and immune cell infiltration and polarization[25], we investigated the impact of TAB on the melanoma TME and its response to ICB therapy. We systematically characterized the effect of human melanoma cells on autologous B cells *in vitro* (Supplementary Figure 1) and observed induction of a plasmablast-like phenotype with expression of both, pro- and anti-inflammatory molecules. Independent single-cell (sc) RNA-sequencing (seq) data confirmed this phenotype in human melanoma metastases together with the expression of chemokines critical for immune cell recruitment. Consistently, depletion of TAB from the TME drastically decreased overall inflammation and immune cell numbers and, vice versa, the frequency of plasmablast-like TAB in pre-therapy melanoma samples correlated with improved response and patient survival to ICB therapy.

Results

Melanoma TAB are composed of multiple phenotypes

We used 7 color multiplex immunostaining (CD20, CD19, CD5, CD27, CD38, CD138, and DAPI) to approximate 6 different TAB-subpopulations (Figure 1A, 1B) in whole tissue sections of melanoma metastases from 41 different patients (Figure 1A). In total, we observed plasma cell-like TAB in 40 samples (98%, Figure 1C), plasmablast-like TAB in 39 samples (95%, Figure 1D), activated B cell-like TAB in 37 samples (90%), germinal-center B cell-like TAB in 31 samples (76%), transitional B cell-like TAB in 29 samples (71%), and memory B cell-like TAB in 26 samples (63%). TAB were primarily located at the invasive tumor-stroma margin in line with previous reports[13,14,26]. This suggests a preferentially contact independent communication between melanoma cells and TAB.

A)

TAB subpopulations	IHC-staining pattern
Plasmablast-like	CD19^{+} CD20^{-} CD38^{+} CD138^{-}
Plasma cell-like	CD19^{+} CD20^{-} CD138^{+}
Memory B cell-like	CD19^{+} CD20^{+} CD38^{-} CD138^{-} CD27^{+}
Germinal center B cell-like	CD20^{+} CD38^{+} CD138^{-} CD5^{-}
Activated B cell-like	CD19^{+} CD20^{-} CD38^{-} CD138^{-} CD27^{+}
Transitional cell-like	CD20^{+} CD19^{-} CD138^{-} CD5^{+}

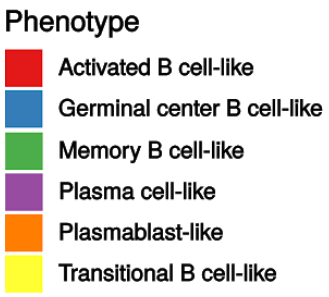

B)

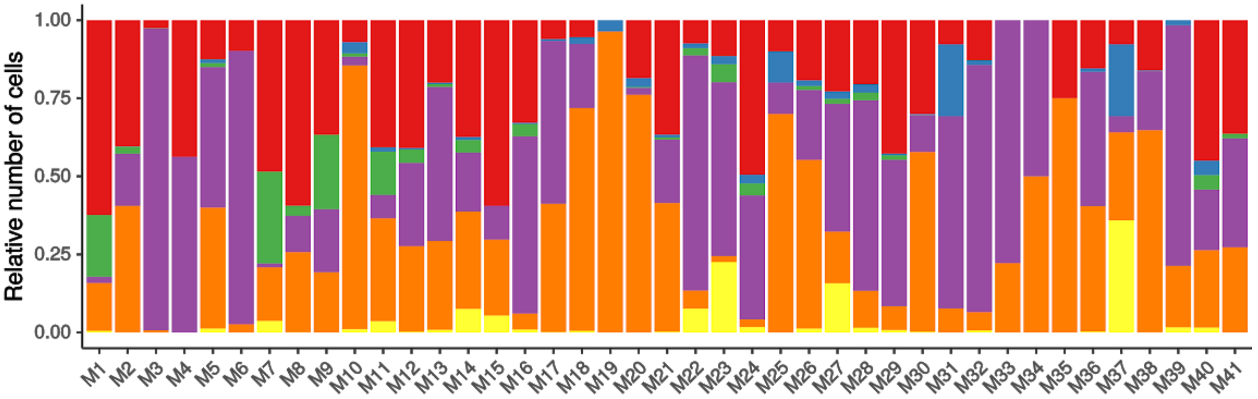

C)

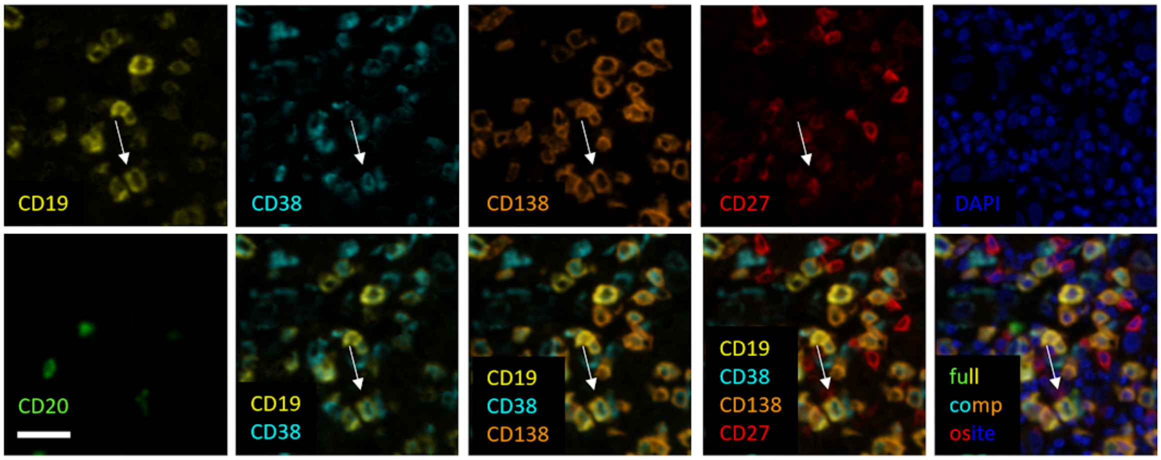

D)

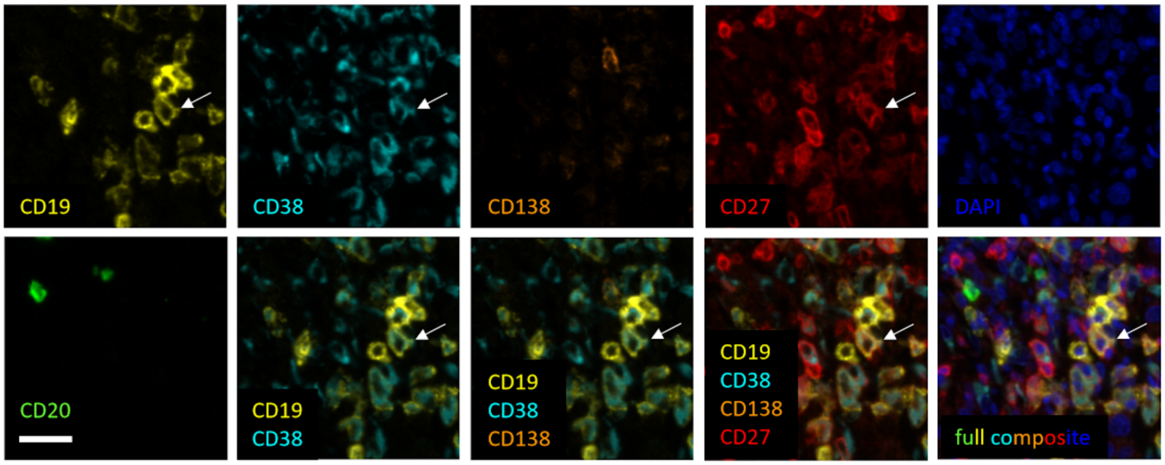

Figure 1: Melanoma TAB are enriched for plasmablast- and plasma cell-like phenotypes. A) Marker combinations used to identify TAB subpopulations by 7 color multiplex immunostaining. B) Relative frequency of different TAB phenotypes in whole tissue sections of 41 metastatic melanomas C), D) Multiplex immunostaining identifies in (C) $CD19^{+}CD20^{-}CD38^{+}CD138^{+}CD27^{+}$ plasma cell-like and in (D) $CD19^{+}CD20^{-}CD38^{+}CD138^{-}CD27^{+}$ plasmablast-like TAB. Here, serial images display the same cells from a stromal area at the invasive tumor margin. A full composite image together with DAPI nuclear staining (bottom right) and images for each of the individual markers and different combinations from the composite image are shown. Arrow depicts one of several plasma cell-like (C) and plasmablast-like (D) TAB. Scale bars represent 20µm.

Human melanoma cells directly induce NFKB activation in TAB through soluble factors

The location of TAB at the invasive tumor-stroma margin suggests that melanoma cells communicate with TAB through soluble factors. We therefore exposed immortalized peripheral blood- and tumor-derived B cells derived from 4 patients with metastatic melanoma to melanoma-conditioned medium (MCM) collected from autologous early passage melanoma cells. As we were unable to establish a tumor cell line for a 5th patient, we additionally exposed this patient's B cells, as well as the B cells of all other patients with MCM from patient 1. From these B cell cultures we were able to generate RNAseq (n=11) and complementary proteomic (n=8) profiles.

752 genes/proteins were identified as significantly regulated by MCM in both, the RNA-seq and proteomics results. Additionally, 802 proteins were found only significantly regulated in the proteomics data and 4,231 genes found only significantly regulated in the

RNA-seq data (Benjamini-Hochberg (BH) corrected p-value < 0.05, Supplementary Table 1). There was no marked difference between peripheral blood- and tumor-derived B cells with no significantly differentially expressed proteins observed in the proteomics data (Figure 2A). The estimated fold changes of genes identified as significantly regulated in both approaches showed a high linear correlation (Spearman cor = 0.77, $p < 0.01$, Supplementary Figure 2A). The subsequent pathway analysis (see Methods) showed upregulation of pathways associated with inflammation, immunity, B cell receptor signaling and intracellular signal transduction, and downregulation of pathways associated with cell cycle, cell division, DNA replication, DNA repair, translation, and transcription (Supplementary Table 1).

One of the most significantly upregulated pathways was “tumor necrosis factor (TNF) signalling via NFKB”. NFKB is a key transcription factor for B cell activation in inflammation and immune response[27]. This pathway includes, among others, CD69, CD80, CD30 (TNFRSF8), and CD137 (4-1BB/TNFRSF9) which were significantly upregulated in our proteomics and transcriptomics data (with CD30 (TNFRSF8) among the top-20 upregulated genes) and are known to be upregulated in B cells upon activation. Even though TNF itself showed no significant differences, TNF alpha induced protein 2 (TNFAIP2) was significantly upregulated as indirect evidence for TNF signalling. In addition, we observed the up-regulation of CD40 signalling genes which also acts via NFKB. We also found increased phosphorylation of PRKCB which plays a key role in B cell activation by regulating BCR-induced NFKB activation (with no significant differences in transcriptomics or global proteomics analysis). Similarly, we observed an increased phosphorylation of NKAP which is involved in TNF- and IL-1 induced NFKB activation. Therefore, melanoma-derived soluble factors directly induce a signalling pattern in B cells associated with activation in inflammation and immune response.

Melanoma conditioned medium induces a plasmablast-like dominated TAB population

The downregulation of cell cycle associated pathways coincided with an unexpected down-regulation of CD20 (MS4A1, $p = 0.05$ RNA-seq, $p = 0.08$ proteomics) and CD19 (BH adjusted $p < 0.01$ proteomics). This indicates a phenotypic change of B cells. To identify this phenotype, we first used the Reactome functional interaction network[28] and extracted all neighbours of CD19 and their neighbours (Figure 2B). While known key developmental phenotypes, including reported regulatory B cell markers such as CD147, CD24, CD27, CD25, CD39, CD73, or CD138 together with transcription factors BLIMP-1, XBP-1 were not significantly regulated, downregulated (IRF4, CD71) or not detectable (CD5, TIM1), we found a significant upregulation of CD38 (proteomics, BH adjusted $p = 0.01$), together with PAX5 (BH adjusted $p < 0.01$ RNA-seq).

9-color FACS staining ($n = 12$) showed changes of CD19, CD20, CD24, CD27, CD38 and CD138 consistent with proteomics and RNA-seq results (Figure 2C). Immunoglobulin D, M and G expression was unchanged (Supplementary Figure 2B). The induction of CD38 points towards a plasmablast/-cytoid differentiation. This is counter-regulated by increased PAX5 expression. These findings correlate with the high proportion of plasmablast-like TAB in human melanomas (Figure 1B). Based on these results, we defined a tumor-induced plasmablast-like dominated B cell population (TIPB) signature with the genes CD27, CD38, and PAX5.

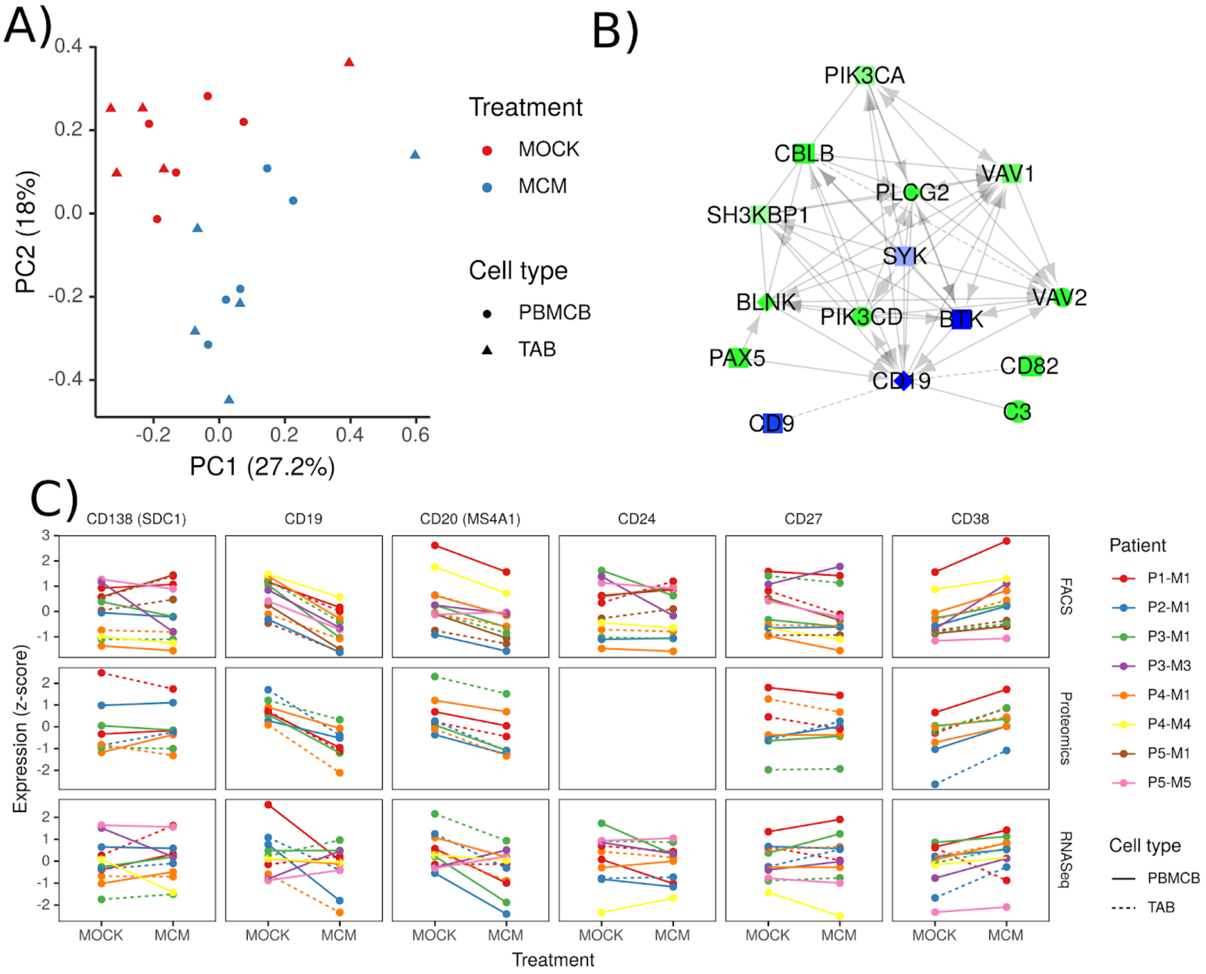

Figure 2: Melanoma conditioned medium induced distinct phenotypic changes in TAB. A) Principal component analysis of all samples based on the proteomics data comparing samples treated with melanoma conditioned medium (MCM) against control treated ones (MOCK). Peripheral blood derived B cells (PBMCB) and TAB are shown through different shapes. The percentage in the axis labels represents the variation explained by the component. Points represent one sample per patient and one sample where individual patient samples were pooled together. B) Interaction network of CD19 with the nearest neighbours depicting the gene abundance estimated by RNA-seq and proteomics. Green represents up-regulation, blue down-regulation, light colors indicate no relevant difference. Diamonds are genes only detected

in proteomics, rectangles genes only detected in RNA-seq, circles genes identified by both methods. For the actual data analysis, the network was constructed using the two nearest neighbours. C) Expression of key differentiation markers estimated by FACS (geo mean expression), proteomics and RNA-seq for melanoma conditioned medium (MCM) and control (MOCK) treated immortalized peripheral blood derived B cells (PBMCB) and TAB. All values are shown as z-scores.

Melanoma conditioned medium induces distinct functional signatures in TAB which are found *in vivo*

Cell surface signaling molecules, immuno-stimmulatory, and -suppressive cytokines are the most useful factors to distinguish TAB subsets with distinct functions. To profile TIPB on a functional level, we manually extracted six key immunological functional gene sets from the MCM-induced, enriched CD19 interaction network (Figure 2B, see Methods). To reduce the bias invariably introduced by our small sample size, we additionally extracted highly correlating genes from the TCGA skin cutaneous melanoma dataset to create six signatures based on existing literature. We explicitly chose a literature-backed strategy to avoid generation of purely data-driven signatures of unclear and arguable biological significance. In line with the pathway analysis (increased signalling via NFKB), these key upregulated gene sets included *activation-associated genes* (CD69, CD72, CFLAR, FGFR1, SELPLG, CD86), *co-stimulatory genes* (ICAM3, TNFRSF13C, CD40, CD72, C3, CD80, CD86, CD27, CD28, ICOS, TNFRSF9, CD40LG, ARHGDIB), and *pro-inflammatory genes* (TNF, IL12B, IL18, LTA, TNFAIP2, C3, HCK). In addition, *immune checkpoint-associated genes* (CD274, PDCD1LG2, TNFRSF14/HVEM, LGALS9, BTLA, LAG3, HAVCR2/TIM-3, ADORA2A), *immunosuppressive*

genes (IL10, TGFB1), as well as *B cell exhaustion-associated genes* (PDCD1, FCRL4, SIGLEC6, CD22) were upregulated.

We used scRNA-seq data by Sade-Feldman *et al.* on immune-checkpoint blockade and response in human melanoma[29] to validate these functional signatures *in vivo*. TAB clustered into distinct groups which we correlated with known developmental B cell phenotypes (Figure 3A). Overall, all B cell phenotypes expressed comparable levels of our functional signatures (Figure 3B) but with distinct differences on the single gene level (Supplementary Table 2). Plasmablast-like and memory-like B cells expressed substantial levels of immunosuppressive cytokines such as TGFB1 and IL-10 (Figure 3C). We could additionally validate the expression of the latter through multiplex immunostaining in plasmablast-like B cells (Figure 3D). Remarkably, TAB also expressed numerous chemokines including T (reg) cell, and macrophage chemoattractants CCL3, CCL3L1, CCL4, CCL5, CCL28 and CXCL16[30–33] in plasmablast-like B cells (Figure 3C). Together, these results indicate that TIPB are able to regulate inflammation and shape the cellular composition of the human melanoma TME.

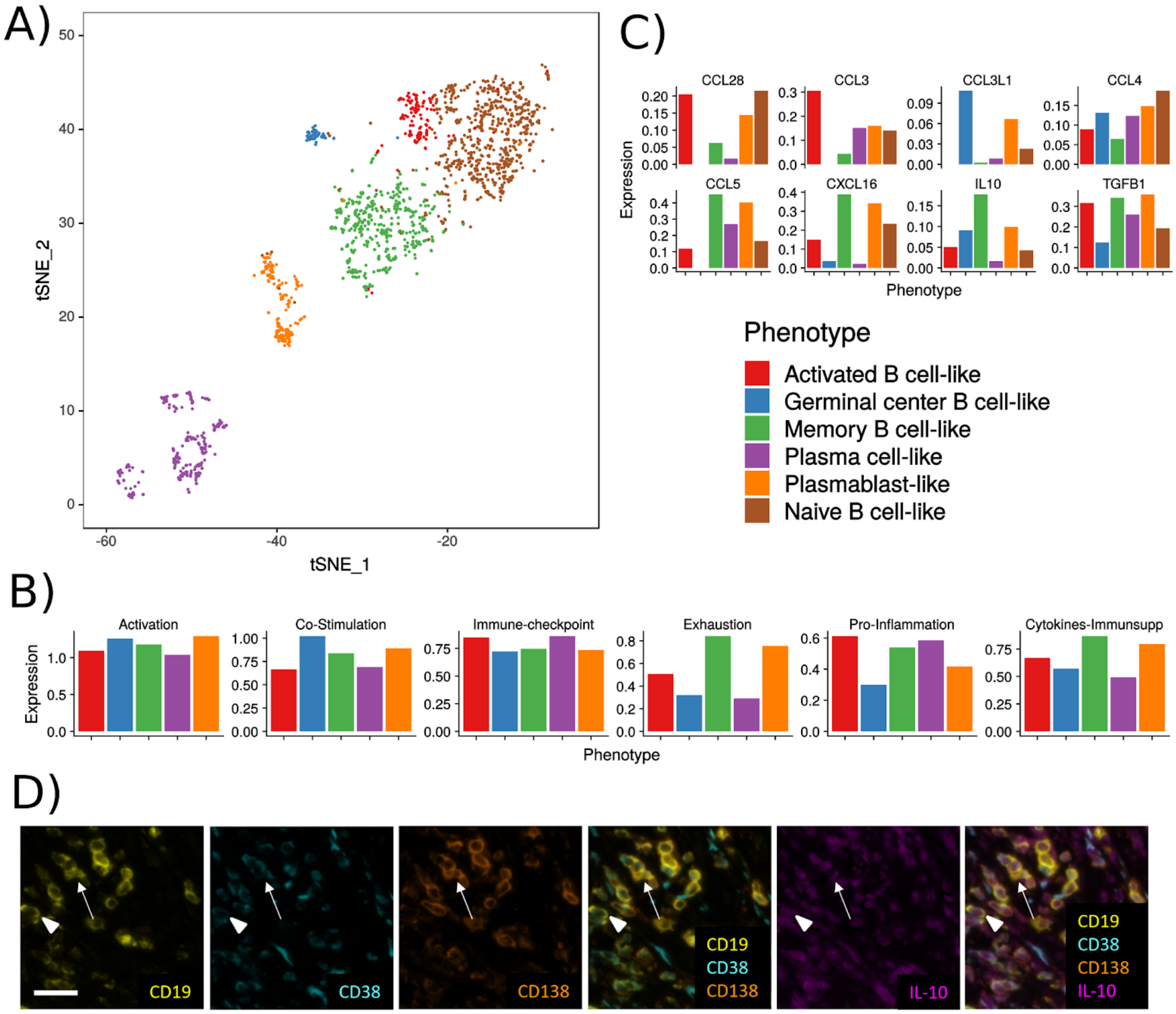

Figure 3: scRNA-seq data from human melanoma verifies the observed functional phenotypes *in vivo*. A) T distributed stochastic neighbor embedding representation of all B cells with their respective phenotype annotations from the Sade-Feldman *et al.* dataset. B) ssGSEA estimated expression of the functional signatures based on the average gene expression per B cell phenotype. C) Average expression of specific chemokines and immunosuppressive cytokines per B cell phenotype. D) Confirmation of IL-10 expression in plasma cell-like (arrow) and plasmablast-like (arrowhead) TAB by quadruple marker immunostaining. Scale bar represents 20 μm.

TIPB correlate with the predicted functional signatures, CD8+ T cells, macrophages, and overall survival

Next, we tested whether the TIPB signature correlates with the identified functional signatures in independent melanoma cohorts and evaluated its effect on clinical outcome in the TCGA skin cutaneous melanoma cohort. All functional signatures (except for the *immunosuppressive genes,* Spearman correlation 0.49, BH adjusted p < 0.01) showed a strong linear correlation with our TIPB signature (Spearman correlation >= 0.78, BH adjusted p < 0.01, Supplementary Figure 3A). Our TIPB signature was also significantly correlated with the expression of CD8A (Spearman correlation 0.87, p < 0.01, Supplementary Figure 3B) as well as with the abundance of $CD8^+$ T cells (Spearman correlation 0.82, p < 0.01, Supplementary Figure 3C) and macrophages (Spearman correlation 0.71, p < 0.01) as estimated by xCell[34]. Established signatures describing the inflammatory TME and possible response to anti-PD-1 therapy (tumor inflammatory score[35], interferon gamma[36], T-cell exhaustion[37], and T-cell effector[38] signatures) additionally showed a high linear correlation with our TIPB signature (Spearman correlation >= 0.85, BH adjusted p < 0.01, Supplementary Figure 3D). Finally, high expression (above median) of our TIPB signature was correlated with longer overall survival in the TCGA cohort[39] (Figure 4A). These results show, that the TIPB and the predicted functional signatures, are highly associated with T cell abundance and inflammation, and, importantly, are linked with patient outcome.

Anti-PD-1 therapy frequently leads to an increase in B cell numbers which should enhance our functional signatures. We used the transcriptomics data by Riaz *et al.* containing (partially matched) 51 pre-anti-PD-1 therapy and 58 post-anti-PD-1 therapy samples[40]. In this independent cohort, all signatures with exception of the immunosuppressive genes (Spearman

correlation = 0.6, BH adjusted $p < 0.01$) showed a strong linear correlation with the TIPB signature (Spearman correlation >= 0.77, BH adjusted $p < 0.01$, Supplementary Figure 4A). Again, the TIPB signature correlated with CD8A expression (Spearman correlation 0.9, BH adjusted $p < 0.01$, Supplementary Figure 4B), estimated $CD8^+$ T cell (Spearman correlation 0.76, $p < 0.01$, Supplementary Figure 4C) and macrophages (Spearman correlation 0.68, $p < 0.01$) abundance. We also observed a consistent up-regulation of all signatures during anti-PD-1 therapy (one-sided t-test BH adjusted $p <= 0.03$, df = 100-107, t = -2.03 - -2.77, immunosuppressive genes BH adjusted p = 0.06, df = 96.33, t = -1.58, exhaustion BH adjusted p = 0.09, df = 106.7, t = -1.35, Figure 4B). Interestingly, high expression of our TIPB signature before therapy (top 25% versus lower 25%) predicted overall survival (Likelihood ratio test p = 0.06, Figure 4C). While the TIPB signature did not correlate with clinical response (Supplementary Figure 5) in this dataset, scRNA-seq showed plasmablast-like and naive-like B cell frequencies in pre-therapy tumor samples to be significantly higher in patients responding to ICB therapy (two-sided Wilcoxon rank sum test, BH adjusted p-value = 0.04, Figure 4D).

In order to assess the functional impact of TIPB on T cells we performed a surrogate co-culture assay with MCM-induced B cells and NF-κB-reporter Jurkat T cells *in vitro*. There, MCM-induced B cells significantly increased the effect (NF-κB promoter activity) of PD-1 blockade on PD-1-expressing Jurkat T cells (two-sided t-test, BH adjusted p 0.02 - 0.06, see Methods, Figure 4E). Additionally, MCM increased B cell viability (Supplementary Figure 6). Together, these functional data support the clinical importance of the identified TIPB population.

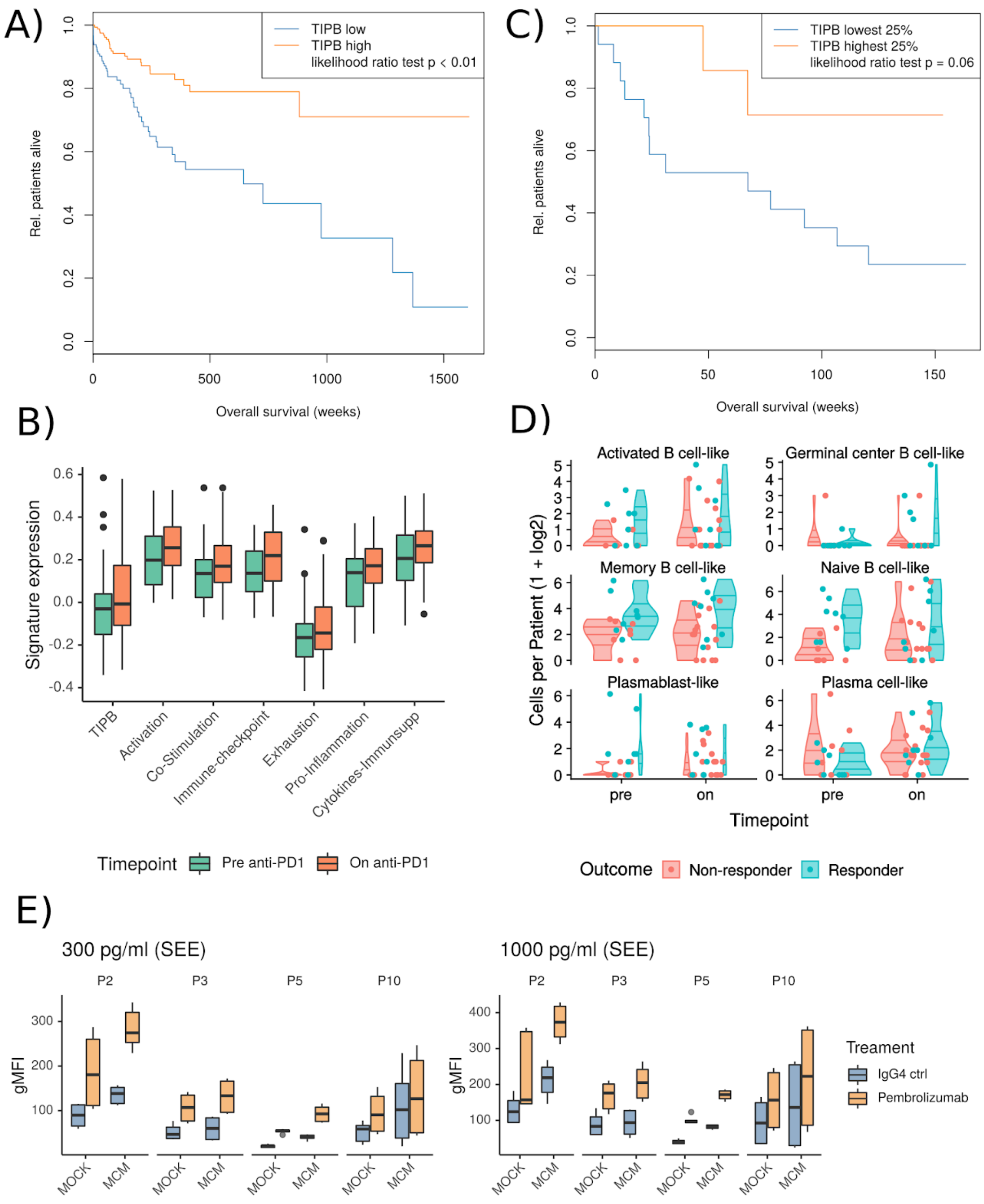
A)
TIPB low
TIPB high
likelihood ratio test p < 0.01
Rel. patients alive
Overall survival (weeks)
C)
TIPB lowest 25%
TIPB highest 25%
likelihood ratio test p = 0.06
Rel. patients alive
Overall survival (weeks)
B)
Signature expression
TIPB
Activation
Co-Stimulation
Immune-checkpoint
Exhaustion
Pro-Inflammation
Cytokines-Immunsupp
Timepoint
Pre anti-PD1
On anti-PD1
D)
Activated B cell-like
Germinal center B cell-like
Memory B cell-like
Naive B cell-like
Plasmablast-like
Plasma cell-like
Cells per Patient (1 + log2)
pre
on
Timepoint
Outcome
Non-responder
Responder
E)
300 pg/ml (SEE)
1000 pg/ml (SEE)
P2
P3
P5
P10
gMFI
MOCK
MCM
Treament
IgG4 ctrl
Pembrolizumab

Figure 4: TIPB predict improved survival of melanoma patients. A) Survival analysis of patients expressing high or low levels of the TIPB signature separated by the median expression in the TCGA melanoma cohort. B) Expression of the TIPB signature and all functional signatures before and on anti-PD-1 therapy as estimated in the Riaz *et al.* dataset. C) Survival analysis based on the TIPB signature in the pre-treatment samples comparing top 25% expressing samples against the lower 25% samples in the Riaz *et al.* dataset. D) Frequencies of B cell phenotypes (logarithmic scale) before and on ICB therapy in the scRNA-seq dataset from Sade-Feldman *et al.* separated by response. Plots represent the relative frequencies, lines represent the 25%, 50%, and 75% quantiles. E) NF-κB promoter activity of PD-1-expressing Jurkat T cells co-cultured with control and MCM-conditioned B cells (“MOCK” and “MCM”). Activation was measured by immunofluorescence (geo mean immunofluorescence intensity, gMFI) after stimulation with 300 pg/ml (left panel) and 1 ng/ml (right panel) *Staphylococcus* Enterotoxin E (SEE).

Loss of TAB reduces inflammation in the tumor microenvironment

We evaluated the loss of TAB in a cohort of patients with metastatic melanoma treated with anti-CD20 antibodies[13,40] (see Methods, Supplementary Figure 1). The dataset consists of 9 patients with pre- and on-anti-CD20 therapy samples (therapeutic setting) and 2 patients with pre- and on-therapy samples, where the metastases developed *de-novo* in B cell depleted patients on therapy[41] (adjuvant setting, Supplementary Table 5). Out of these 11 patients, matched pre- and on-therapy samples of 6 patients could be characterised using whole tissue RNA-seq. Principal component analysis showed no systematic difference between the two patient groups (Figure 5A).

Next to the expected down-regulation of CD19 and CD20 (MS4A1), all patients showed a consistent, significant down-regulation of CD8A on anti-CD20 therapy (BH adjusted $p = 0.02$, Supplementary Table 3). This was linked to a consistent reduction of $CD8^+$ T-cells (one-sided, paired t-test, BH adjusted $p = 0.07$, $t = 1.74$, $df = 5$) and macrophages (one-sided, paired t-test, BH adjusted $p = 0.07$, $t = 2.17$, $df = 5$, Figure 5B). A signature consisting of CD4 and FOXP3 to approximate the abundance of $FOXP3^+$ T cells also decreased significantly (one-sided, paired t-test $p = 0.05$, $df = 5$, $t = 2.03$, Figure 5B).

Anti-CD20 therapy caused a significant downregulation of our TIPB signature and all our associated functional signatures (one sided, paired t-test, BH adjusted p-value 0.01 - 0.04, t = 2.3 - 4.8, df = 5, Supplementary Figure 7A). Published signatures describing the inflammatory TME and possible response to anti-PD-1 therapy (tumor inflammatory score[35], interferon gamma[36], T-cell exhaustion[37], and T-cell effector[38] signatures) highly correlated with our TIPB signature (Spearman correlation 0.7 - 0.9, BH adjusted $p < 0.01$, Supplementary Figure 7B) and showed a significant decrease on anti-CD20 therapy (one-sided, paired t-test, BH adjusted $p = 0.01$, $df = 5$, $t = 3.11 - 5.46$, Figure 5C).

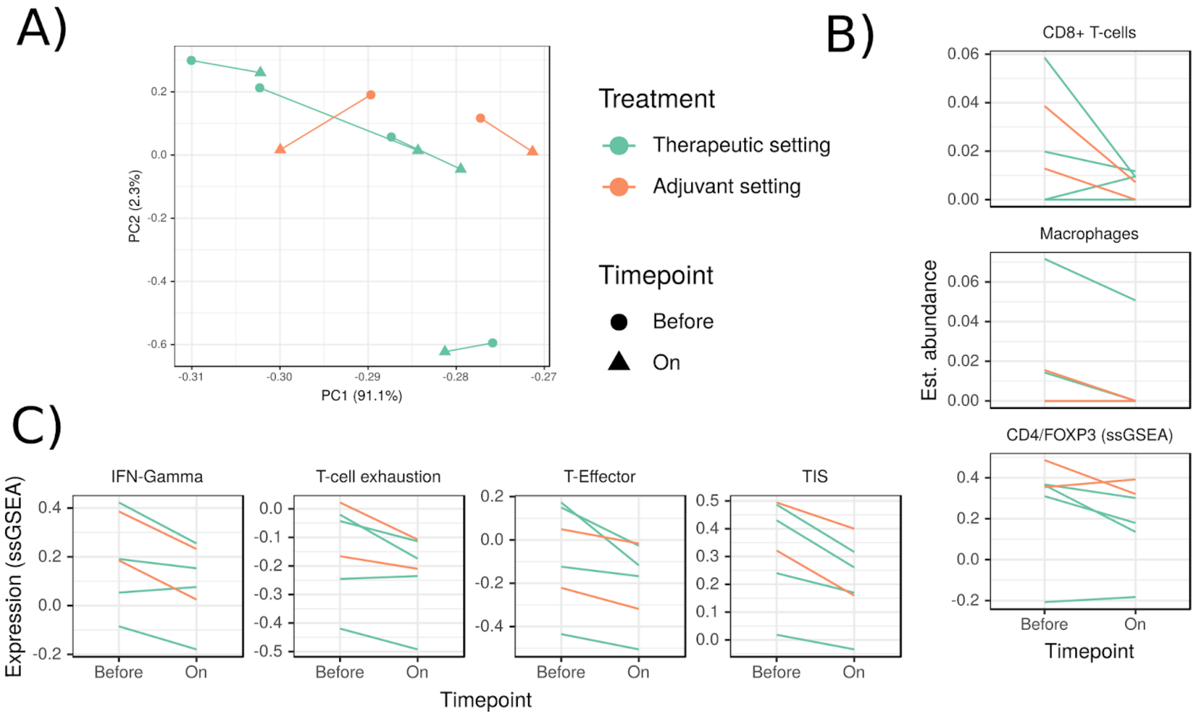

Figure 5: Reduction of TIPB through anti-CD20 therapy reduces tumor inflammation and CD8$^+$ T cell numbers. A) Principal component analysis of RNA-seq data from melanoma samples before and on anti-CD20 therapy. On-therapy samples consist of metastases affected by anti-CD20 therapy (therapeutic setting, green lines) and of metastases that developed *de-novo* in B cell depleted patients (adjuvant setting, orange lines). Percentage numbers in axis labels represent the explained variation by each component. Lines link a patient's samples. B) xCell estimated abundance of cell types in tissue samples before and on anti-CD20 therapy. Abundance of CD4$^+$FOXP3$^+$ was estimated using ssGSEA since no comparable xCell signature exists. C) Expression of established inflammation (interferon (IFN) gamma, tumor inflammatory score (TIS) and T cell gene signatures before and on anti-CD20 therapy.

We further used 6 color multiplex immunostaining to characterise and quantify B and T cells from a total of 25 tumor samples from the patients of the therapeutic cohort (see Methods).

This confirmed that B cell depletion led to a marked reduction of both $CD8^+$ and $CD4^+$ T cells (one-sided Wilcoxon rank sum test, BH adjusted $p < 0.01$, 0.06 respectively) at the invasive tumor-stroma margin without significantly affecting total cell numbers (two-sided t-test $p = 0.9$, $t = -0.02$, $df = 52$, Figure 6A, Supplementary Figure 8A, B). Loss of B cells also coincided with a significant reduction in the total area of tertiary lymphoid structures (one sided paired t-test $p = 0.04$, $t = 2.5$, $df = 3$, Supplementary Figure 8C, D). This effect was stable over at least six months (Figure 6B, Supplementary Figure 8E). In one patient where samples were available over a timespan of close to two years, a short influx of T cells was observed around week 25, shortly prior to disease progression and termination of anti-CD20 therapy (Figure 6B). Upon termination of subsequent (poly-)chemotherapy (week 76, due to continued disease progression), $CD19^+$ B cells and T cells reoccurred. In on- and after-anti-CD20 therapy tumor samples where we were able to detect T cells, we did not observe an effect on tissue-resident, central and effector memory T cell composition (Supplementary Figure 9).

These data validate the essential role of TIPB to sustain tumor inflammation and recruit CD8+ T cells in human melanoma.

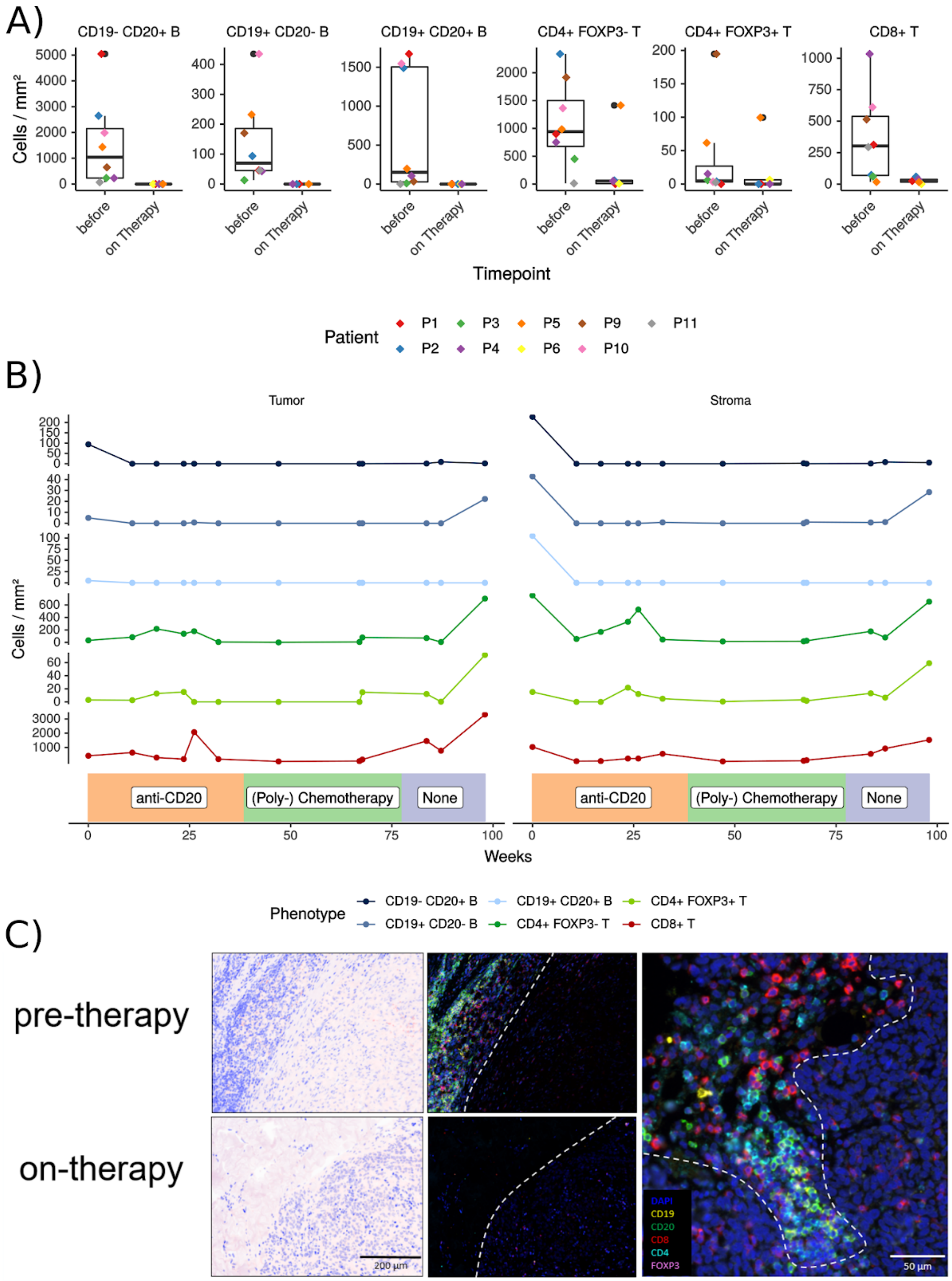
A)
CD19- CD20+ B
CD19+ CD20- B
CD19+ CD20+ B
CD4+ FOXP3- T
CD4+ FOXP3+ T
CD8+ T
Cells / mm²
before
on Therapy
Timepoint
Patient
P1
P2
P3
P4
P5
P6
P9
P10
P11
B)
Tumor
Stroma
Cells / mm²
anti-CD20
(Poly-) Chemotherapy
None
Weeks
Phenotype
CD19- CD20+ B
CD19+ CD20- B
CD19+ CD20+ B
CD4+ FOXP3- T
CD4+ FOXP3+ T
CD8+ T
C)
pre-therapy
on-therapy
200 µm
DAPI
CD19
CD20
CD8
CD4
FOXP3
50 µm

Figure 6: A) B and T cell numbers at the invasive tumor-stroma margin quantified using 6 color multiplex immunostaining in tumor samples from 9 patients obtained before and on therapy (at week 9±2). B) Longitudinal analysis of tumor samples obtained over nearly two years from patient 4. Sample regions were classified as invasive tumor-stroma margin ("Stroma") and intratumoral ("Tumor"). The lowest panel depicts the respective therapy at the given timepoints. C) Multiplex immunostaining for indicated antigens (right) of a representative patient-matched pair of melanoma samples before (top) and on (down) anti-CD20 therapy. Images were taken at the invasive tumor-stroma margin (left: pathology overviews), separated by a dashed line (middle: corresponding immunostaining overviews). Close up showing a dense B and T cell infiltrate at the invasive tumor-stroma margin (right). Note loss of B and T cell infiltrates, but not tumor cellularity on therapy (left and middle, down).

Discussion

The current focus in clinical tumor immunology centers on T cells, both in terms of treatment concepts and signatures for patient stratification. Existing data on the role of B cells is controversial with the prevailing concept of regulatory B cells that inhibit the inflammatory anti-tumor response. Here we show that TAB are essential to sustain the inflammatory TME through melanoma-induced subpopulations enriched for plasmablast-like cells. In our own and independent sc-RNA-seq data, these subpopulations simultaneously express pro- and anti-inflammatory factors pointing towards a more complex role of TAB.

The co-expression of immunostimulatory and -inhibitory cytokines and cell surface receptors characterizes TIPB as a rather unique B cell population. Melanoma-induced stimulatory signatures indicated antibody-independent functions of TIPB, such as antigen

presentation, T cell activation and cytokine production with the ability to promote local inflammation as recently shown in multiple sclerosis[42]. TIPB also expressed inhibitory receptors and ligands as well as IL-10 and TGFB1 as indicated by scRNA-seq and multiplex immunostaining data. In this respect TIPB resemble Breg, which directly and indirectly suppress proliferation, anti-tumor activity, and differentiation of several immune cells residing in the TME[43–48]. The expression of these inhibitory factors in TIPB is also in line with previous reports on human $CD27^{int}CD38^{+}CD138^{-}$ plasmablasts as a major $IL\text{-}10^{+}$ B cell population[45], the suppression of $IL\text{-}17A^{+}CD4^{+}$ T cells[49] in human colorectal cancers being highly enriched for $IL\text{-}10^{+}CD19^{lo}CD27^{hi}$ plasmablasts, and murine $IgA^{+}PD\text{-}L1^{+}IL\text{-}10^{+}$ plasmocytes that prevent $CD8^{+}$ T cell-mediated regression of hepatocellular carcinoma[7] and impede T cell-dependent chemotherapy of castration-resistant prostate cancer[4]. Our data expand these observations by pinpointing the additional expression of inhibitory ligands such as LGALS9/Galectin-9, and TNFRSF14/HVEM in TIPB. These ligands can stimulate their cognate inhibitory receptors HAVCR2/TIM-3 and BTLA, which are induced on T cells upon persistent antigen exposure and drive T cell dysfunction.

This seemingly contradictory role of TAB, with both pro- and anti-inflammatory roles is in-line with findings from other diseases. In murine models of autoimmune diseases such as experimental multiple sclerosis and systemic lupus erythematodes[50,51] B cell depletion therapy has opposing effects dependent on the stages of the disease. While B cell depletion in the early phase of disease exacerbates disease severity, it attenuates disease severity in the late phase of the disease. In line with these data, $CD20^{+}$ TAB in human oro- and hypopharynx cancer are associated with a favorable outcome in early disease but a negative outcome in advanced disease[52]. In addition, a favorable prognostic effect of $CD20^{+}$ TAB can be linked only to distinct histologic or molecular cancer subtypes, as shown for breast cancer (in ER-, basal, and HER2+,

but not triple negative subtypes[53,54]), ovarian cancer (in high-grade serous, but not other subtypes[55]) and mesothelioma (in the epithelioid, but not non-epithelioid subtype[56]).

Together with our observations, these data emphasize the importance of future context-specific TAB analyses in human cancer considering disease stages, molecular subtypes and therapies. The “tide model” describes expression of co-stimulatory and inhibitory signals as a dynamic system to fine-tune immune responses[57]. Additionally, the “immune set point model” highlights the often subtle contribution of intrinsic and extrinsic factors that modulate tumor inflammation and tip the balance between immunity and tolerance[58]. In human melanoma, even small variations within the release of tumor antigens/neoantigens, extrinsic or environmental factors, the secretion and consumption of cytokines and chemokines, distinct genetic subtypes as well as therapeutic agents can tip this balance. The well-known dichotomy of clinical responses to ICB could thus reflect the net outcome of all these drivers and thus the overall inflammatory status of the TME[58]. Similarly, pro-tumorigenic[13] as well as pro-immunogenic functions of TIPB can result from a disbalanced expression of inflammation-modulating factors and depend on the degree of inflammation in the respective TME.

The functional plasticity of TIPB emphasizes the importance of a careful evaluation of their functions in different tumor and host contextes to guide the development of TIPB-targeting strategies for novel cancer combination therapies.

Acknowledgements

This work was supported by the FWF-Austrian Science Fund (project P31127-B28 to SNW and SFB F4609 to WFP) and received funding from the European Research Council (ERC) under the European Union's Horizon 2020 research and innovation programme under grant

agreement No 788042. We thank Bjoern Wendik (Perkin Elmer) for support in tissue imaging and data acquisition of multiplex immunostainings, Barbara Sternitzky and Claudia Kokesch for technical support in processing tissue microarrays, the Biomedical Sequencing Facility at CeMM for assistance with next-generation sequencing, the MFPL Mass Spectrometry team for technical support with proteomics, and the VBCF for providing the mass spectrometry instrument pool. This work was supported in part by NIH grants P01 CA114046, P50 CA174523, U54 CA224070, DoD - PRCRP WX1XWH-16-1-0119 [CA150619], and the Dr. Miriam and Sheldon G. Adelson Medical Research Foundation to MHe.

Author contributions

Hypothesis & study protocol JG, GZ, MHe, RS, SNW **Clinical study & patient materials** JG, MMG, FR, PP, WFP, SNW **Histopathology** CW, MC, KG, HL, KDM, PP, SNW **FACS & functional assays** WB, MS, PS, KGM, SNW **Proteomics** JG, MMG, MHa, CW **RNA-seq** JG, TW, CW, TP, CB **Bioinformatics** JG **Manuscript** JG, SNW.

All authors contributed to the final version of the manuscript.

Competing interests

The authors declare no competing interests.

Correspondence and requests for materials should be addressed to stephan.wagner@meduniwien.ac.at.

Methods

Data availability

RNA-seq data were deposited in the ArrayExpress[59] database at EMBL-EBI (www.ebi.ac.uk/arrayexpress) under accession numbers E-MTAB-7472 (induction experiment) and E-MTAB-7473 (anti-CD20 study). The mass spectrometry proteomics data were deposited to the ProteomeXchange Consortium via the PRIDE partner repository[60] with the dataset identifier PXD011799 (induction experiment).

Patient-derived material

10 patients with metastatic melanoma were treated with the anti-CD20 antibody ofatumumab in a therapeutic setting[13,61](Clinical trials number NCT01376713). Baseline demographic and clinical characteristics of these patients as well as response data were given in ref[13], additional clinical data are shown in Supplementary Table 5). Patients had to have adequate biopsy material for *(i)* multiplex immunohistochemistry (IHC) staining and/or *(ii)* RNA-seq analysis from time points before initiation of therapy (week 0) and/or under anti-CD20 therapy (week 9±2, with additional later samples as indicated for some patients), when immunophenotyping of peripheral blood mononuclear cells showed a consistent loss of $CD19^+$ B lymphocytes (week 2 and later). From 9 patients formalin-fixed paraffin-embedded (FFPE) tumor biopsies could be subjected to multiplex IHC, RNA-seq data were successfully generated from matched samples of four patients. We also collected pre-therapy peripheral blood mononuclear cell (PBMC) samples for generation of EBV-immortalized peripheral B cells[13,61] as well as adequate pre-therapy tumor biopsy material for generation of *(i)* early passage melanoma cells[61,62] and *(ii)* autologous

EBV-immortalized TAB[13,61] from tumor single cell suspensions. From four patients all these samples were successfully established, from a further patient EBV-immortalized peripheral B cells as well as EBV-immortalized TAB could be established. To increase the number of patients with pre-therapy early passage melanoma cells and autologous EBV-immortalized peripheral B cells and TAB, we included one further patient who was considered for but did not undergo anti-CD20 therapy.

In addition, of two patients with metastatic melanoma who were treated with the anti-CD20 antibody rituximab in an adjuvant setting[41] (EudraCT number: 2007-005125-30), adequate tumor biopsy material for RNA-seq was available at two time points: before initiation of anti-CD20 therapy, when the tumor mass was completely resected, and after disease progression under therapy. Both studies were investigator-initiated clinical pilot trials. Anti-CD20 antibodies (Ofatumumab vs. Rituximab) were chosen based on availability. Samples were collected under local ethics committee-approved protocols (457-2007, 969-2010) with informed patient consent. Separate ethics committee-approval was obtained to make the derived proteomics and RNA-seq data publicly available (1555-2016). Clinical details are summarized in Supplementary Table 5.

Induction experiments with melanoma-conditioned medium (MCM)

Immediately after surgical resection, melanoma cells and TAB were collected by mechanical and enzymatic dissociation of metastatic melanoma tissue into single cells (tumor dissociation kit, gentleMACS Dissociator, both Miltenyi Biotec GmbH, Bergisch Gladbach, Germany) as described earlier by us[61,62]. Peripheral blood samples were collected in BD Vacutainer CPT cell preparation tubes and PBMC prepared according to the manufacturer’s instructions.

Melanoma cells grown from these single cell suspensions were frozen after passage 2 and stored in liquid nitrogen. Cell purity was determined by flow cytometric staining for melanoma-associated chondroitin sulfate proteoglycan (MCSP) on a FACS Calibur (Becton Dickinson, San Jose, CA). The percentage of MCSP-positive cells was >97%[62]. Sorting of TAB from single cell suspensions and peripheral B cells from PBMC was performed with Dynabeads CD19 Pan B (Life Technologies, Grand Island, NY) as described[61]. After detachment of beads and antibodies (DETACHaBEAD CD19 Kit, Thermo Fisher Scientific), flow cytometric analyses with a (PE)-conjugated CD19 antibody (Miltenyi Biotec) revealed a purity of collected B cell samples between 89.3 and 98.4%, respectively. Immortalization of TAB and peripheral B cells with EBV was performed on autologous irradiated (2× 30 Gy) PBMC as feeder cells essentially as described[61,63]. The purity of immortalized B cells was assessed as >96% in all samples by flow cytometry. A semi-quantitative comparative proteome of autologous freshly isolated vs. immortalized TAB has revealed significant differences predominantly in pathways related to cell cycle proliferation, apoptosis, and interferon response[61]. In addition, we have recently shown that immortalized TAB and peripheral blood-derived B cells can nicely recapitulate the induction of pro-tumorigenic/pro-inflammatory factors/cytokines in freshly isolated TAB and peripheral blood-derived B cells upon exposure to soluble factors from human melanoma cells[13,61] and, thus, can offer a consistent source of quality cells for such experiments.

To produce MCM, early passage (p3) human melanoma cells were grown in complete RPMI 1640 medium (Life Technologies) supplemented with 10% FCS, 100 IU penicillin G, and 100 mg ⁄ ml streptomycin at 37°C in a humidified atmosphere with 5% CO_2. At 80% confluence, the entire media was replaced with fresh medium. MCM was collected 48 hrs thereafter and sterile filtrated. Control medium was prepared the same way, but without melanoma cells. For induction experiments, $5x10^5$ human B cells were seeded in 24 well cell culture plates (Corning

Costar) and incubated in 1 ml MCM or control medium for 48 hrs. About 50% of media was substituted with fresh MCM after 24 hrs. B cells were then pelleted by centrifugation at 400*g* for 5 min at RT, snap-frozen and stored at −80 °C.

FACS analysis

Mock- or MCM-treated immortalized B cells or TAB were stained with the following antibodies or matched isotypes and analyzed on a FACS Aria III (BD): CD19 BV711 (clone SJ25C1), CD20 AF700 (clone 2H7), CD24 PE-CF594 (clone ML5), CD27 BV421 (clone M-T271), CD38 APC (clone HIT2), CD138 PE (clone MI15), IgD PE-Cy7 (clone IA6-2), IgG FITC (clone G18-145), IgM BV605 (clone G20-127) (all BD biosciences). Live/dead cell exclusion was performed by addition of 7-AAD (5µg/ml, Calbiochem) prior to acquisition of the samples. Data were analysed using FlowJo 10.4.2 (FlowJo LLC). The gating strategy is shown in the Supplementary Methods.

Jurkat reporter assay

Jurkat E6.1 NF-kB::eGFP and Jurkat E6.1 NF-kB::eGFP-PD-1 reporter T cells have been previously described in detail[64]. For functional assays, reporter cells ($5x10^4$/well) and mock treated or MCM-treated EBV immortalized B cells ($2x10^4$/well) were co-cultured in the presence of *Staphylococcus* Enterotoxin E (SEE, Toxin technology, Inc. Sarasota, FL; used at final concentrations of 300 pg/ml and 1 ng/ml) for 24 h. The PD-1 antibody pembrolizumab (KeytrudaTM, MSD SHARP & DOHME GmbH) or an isotype control antibody (Ultra-LEAF human IgG4 isotype control, Biolegend, San Diego, CA) were added at a final concentration of 10 microg/ml. Following 24 h of co-culture, reporter gene expression was assessed as described in detail[64]. Briefly, cells were harvested and stained using a CD19-APC antibody for exclusion of EBV immortalized B cells. Cells were analyzed using a FACS-CALIBUR flow

cytometer and FlowJo software (Franklin Lakes, NJ). Geometric mean of fluorescence intensity (gMFI) of viable reporter cells was used for further analysis.

Cell viability assay, cell numbers

Cell viability was assayed along[65]. Briefly, B cells were plated in 96 well plates in triplicates and cultured in control medium or MCM (see above) for 24 to 96 hrs. Cell viability was determined by cellular dehydrogenase activity using water soluble WST-8 [2-(2-methoxy-4-nitrophenyl)-3-(4-nitrophenyl)-5-(2,4-disulfophenyl)-2H-tetrazolium], monosodium salt according to manufacturer's instructions (Dojindo EU GmbH, Munich, Germany). 20 µl of WST-8 reagent were added to 200 µl of culture volume per well and incubated for 4 hrs at 37°C, 5% CO2. The absorbance was determined using a microplate reader (Bender MedSystems GmbH, Vienna, Austria) at 450 nm with 620 nm as a reference wavelength.

Cell numbers were determined by counting B cells plated in 96 well plates at equal cell numbers in triplicates and cultured in control medium or MCM (see above) for 24 to 96 hrs. Cells were counted on a FACSCalibur (BD, San Diego, CA).

Proteomics analysis

Cell lysis and protein digestion

All reagents were of analytical grade and obtained from SIGMA-Aldrich, unless specified otherwise. Cells were lysed in freshly prepared lysis buffer containing 100 mM Tris/HCL pH 7.6, 2 % sodium dodecyl sulfate (SDS), 1 mM sodium vanadate, 1 mM NaF, protease inhibitor (cOmplete^{Tm} EDTA-free) and phosphatase inhibitor (PhosSTOP^{Tm}) cocktail tablets (both Roche).

Cell extraction and DNA sharing was assisted by sonication and cell debris pelleted by centrifugation at 20.000 x g for 15 min at 20°C. The supernatant was collected and the total protein concentration determined using the BCA protein assay kit (Pierce Biotechnology).

Filter-aided sample preparation (FASP) was performed using Amicon Ultra Centrifugal 30 kDa molecular weight cutoff filters (Millipore) essentially according to the procedure described by[66]. Dithiothreitol (DTT) was added to a final concentration of 0.1 M and the samples heated at 99°C for 5 minutes. 200 µL of each protein extract was mixed with 3.8 mL of 8 M urea in 100 mM Tris-HCl, pH 8.5 (UA) in the filter unit and centrifuged at 4000 g for 30 min at 20°C to remove SDS. Any remaining SDS was exchanged by urea in a second washing step with 4 mL of UA. Free thiols were alkylated with 2 mL of 50 mM iodoacetamide for 30 min at RT. Afterwards, three washes with 3 mL of UA solution and then three washes with 3 mL of 50 mM triethylammonium bicarbonate (TEAB) were performed. Proteins were digested on filters with trypsin (1:50; Trypsin Gold, Promega) in 50 mM TEAB overnight at 37°C. Digested peptides were collected by centrifugation, acidified with trifluoroacetic acid (TFA), and desalted using Sep-Pak C18 SPE cartridges (50 mg, Waters Corporation) using 80 % acetonitrile containing 0.1 % TFA for the elution and evaporating the solvent in a vacuum centrifuge.

TMT 10plex-labeling of peptides

Isobaric labeling was performed using 10plex tandem mass tag (TMT) reagents (Thermo Fisher Scientific). 200 µg peptide digest per cell line was resuspended in 100 mM TEAB buffer and labeled with 0.8 mg TMT 10-plex™ reagents (Thermo Fisher Scientific) according to the manufacturer's protocol. After one hour incubation at room temperature, samples were quenched for 15 min with 8 µl 5 % hydroxylamine at room temperature. Labeling efficiency was determined running aliquots of the samples on 1h LC-MS/MS gradients and standard database

searches with TMT-tags configured as variable modifications. Corresponding TMT-labeled samples were pooled, acidified with TFA to a final concentration of 1 % TFA and concentrated via Sep-Pak C18 SPE cartridges (200 mg bed volume).

Off-line basic pH reversed-phase fractionation

Off-line high-pH reversed phase fractionations was performed essentially according to[67] with a few modifications. Peptides were separated on a Waters Xbridge BEH130 C18 3.5 µm 4.6 x 250 mm column on an Ultimate 3000 HPLC (Dionex, Thermo Fisher Scientific) operating at 0.8 ml/min. Buffer A consisted of H_2O and buffer B consisted of 90 % acetonitrile (MeCN), both adjusted to pH 10 with ammonium hydroxide. The gradient was set as follows: 1 % B to 30 % B in 50 min, 45 % B in 10 min and 70 % B in 5 min. Fractions were collected at minute intervals and reduced using a vacuum concentrator. Dried peptides were reconstituted in 2 % MeCN, 0.1 % TFA and pooled to a total number of 12 fractions using a concatenation strategy covering the whole gradient[67]. An aliquot of each fraction (5-10 µg) was used for analysis of the global proteome and residual samples were dried in a vacuum concentrator prior to TiO_2 phosphopeptide enrichment.

Phosphopeptide enrichment

Phosphopeptide enrichment was performed using a modified TiO_2 batch protocol. In short, titanium dioxide beads (5 µm; GL Sciences, Japan) were sequentially washed with 120 µl 50% methanol, 300 µl ddH_2O and 2x 300 µl binding solvent (1M glycolic acid, 70 % MeCN, 3 % TFA). In between, beads were spun down and the supernatant was discarded.

Dried peptides of each of the 12 fractions were individually resuspended in 150 µl binding solvent and incubated with the titanium dioxide beads at a bead to peptide ratio of 1:4 for 30 min at RT under continuous rotation. Bead-bound peptides were washed twice with binding solvent,

2x with washing solvent A (70 % MeCN, 3 % TFA) and 2x with washing solvent B (1 % MeCN, 0.1 % TFA). Phosphopeptides were eluted from the beads with 2x 150µl 0.3M NH_4OH. The eluates were acidified by addition of TFA to a final concentration of 2 % and desalted using C18 StageTips[68].

Liquid chromatography and tandem mass spectrometry

Global proteome and the phosphopeptide fractions were separated on an Ultimate 3000 RSLC nano-flow chromatography system using a pre-column for sample loading (PepMapAcclaim C18, 2 cm × 0.1 mm, 5 µm,) and a C18 analytical column (PepMapAcclaim C18, 50 cm × 0.75 mm, 2 µm, all Dionex, Thermo Fisher Scientific), applying a linear gradient over for 2 hours from 2 to 35% solvent B (80% acetonitrile, 0.1% formic acid; solvent A 0.1% formic acid) at a flow rate of 230 nl/min. Eluting peptides were analysed on an Orbitrap Fusion Lumos mass spectrometer equipped with EASY-Spray™ source (all Thermo Fisher Scientific), operated in a data-dependent acquisition mode with a cycle time of 3 s. FTMS1 spectra were recorded at a resolution of 120k, with an automated gain control (AGC) target of 200.000, and a max injection time of 50 ms. Precursors were filtered according to charge state (included charge states 2-6 z), and monoistopic peak assignment. Selected precursors were excluded from repeated fragmentation using a dynamic window (40 s, ± 10 ppm). The MS2 precursor were isolated with a quadrupole mass filter width of 1.2 m/z. For FTMS2, the Orbitrap was operated at 50k resolution, with an AGC target of 100.000 and a maximal injection time of 150 ms for global proteome samples ad 250 ms for phosphopeptide samples. Precursors were fragmented by high-energy collision dissociation (HCD) at a normalized collision energy (NCE) of 42 %.

Transcriptomics analysis

Library preparation and sequencing

The amount of total RNA was quantified using the Qubit Fluorometric Quantitation system (Life Technologies) and the RNA integrity number (RIN) was determined using the Experion Automated Electrophoresis System (Bio-Rad).

For the co-culture experiments the isolated RNA (1µg) was processed using the SENSE mRNA-Seq Library Prep Kit V2 (Lexogen; #SKU 001.96) according to the manufacturer's protocol. The libraries were sequenced on an Illumina HiSeq 2500 platform with 50 bp single-end reads to obtain on average 25 million reads per sample.

For the samples of the anti-CD20 study, RNA-seq libraries were prepared with the TruSeq Stranded mRNA LT sample preparation kit (Illumina) using both, Sciclone and Zephyr liquid handling robotics (PerkinElmer). Library concentrations were quantified with the Qubit Fluorometric Quantitation system (Life Technologies) and the size distribution was assessed using the Experion Automated Electrophoresis System (Bio-Rad). For sequencing, samples were diluted and pooled into NGS libraries in equimolar amounts. Expression profiling libraries were sequenced on Illumina HiSeq 3000/4000 instruments in 50-base-pair-single-end mode.

Base calls provided by the Illumina Real-Time Analysis (RTA) software were subsequently converted into BAM format (Illumina2bam) before de-multiplexing (BamIndexDecoder) into individual, sample-specific BAM files via Illumina2bam tools (1.17.3 https://github.com/wtsi-npg/illumina2bam).

Data analysis and statistical information

All statistical tests were performed using R version 3.5.1[69]. The complete R scripts including the processed input data for all datasets and plots shown in this manuscript are available as Jupyter notebooks in Supplementary File 1.

Proteomics

RAW files were converted to the MGF files using ProteoWizard's msConvert tool (version 3.0.9393) using vendor libraries[70]. Peak list files were searched using MSGF+[71] (version 10089) and X!Tandem[72] (version 2017.2.1.2). The precursor tolerance was set to 10 ppm, fragment tolerance to 0.01 Da for X!Tandem and machine type to QExactive with HCD fragmentation in MSGF+, and 1 missed cleavage was allowed. TMT tags and Carbamidomethylation of C were set as fixed modifications, Oxidation of M and Deamidation of N and Q, and Phosphorylation of S, T, and Y (TiO2 enriched samples only) as variable modifications. Searches were performed against the human SwissProt database (version 17-02), combined with sequences of common contaminants and reversed decoy sequences. Search results were filtered based on the target-decoy strategy at 0.01 FDR if both search engines identified the same sequences, diverging results were discarded, and spectra only identified by one search engines filtered at 0.001 FDR (search engine specific).

For quantitation all spectra with >30% interference based on total ion current were discarded. Isotope impurity correction and normalisation of intensity values was performed using the R Bioconductor package isobar[73] (version 1.26). Protein abundance was estimated using the R Bioconductor package MSnbase[74] (version 2.6.3) using the iPQF method and only peptides uniquely mapping to a single protein. Differential expression analysis was performed using the

R Bioconductor package limma[75] (version 3.35.3). The linear model included the MS run, patient, cell type (PBMCB vs. TAB) as factors next to the treatment group.

Transcriptomics

Bam files were converted to FASTQ format using the samtools package (https://github.com/samtools/samtools, version 0.1.19). The first 9 amino acids were removed using the fastx_trimmer tool (http://hannonlab.cshl.edu/fastx_toolkit, 0.0.14). Alignment was performed using the STAR aligner[76] (version 2.5.3a, 23104886) with the output set to read counts. Alignment was performed against the Ensembl human genome 38 (version 88). Differential expression was assessed using the Bioconductor R package edgeR[77] (version 3.22). Genes with less than 100 transcripts found in all samples were discarded prior to analysis. The linear model included the patient and cell type next to the treatment.

Pathway analysis

Pathway analysis was performed using the Camera algorithm[78] as implemented in limma against the Molecular Signatures Database[79] (MSIGDB, v6.1) hallmark[80] and C2 canonical pathways collection. The analysis was performed separately for proteomics and transcriptomics data and merged using the Cytoscape[81] Enrichment Map plugin[82] (version 3.1.0).

Cell type abundance in whole tissue samples was estimated using xCell[34] (R package version 1.1.0). Abundance of custom signatures was estimated using the ssGSEA approach[34,83] as implemented in the Bioconductor R package GSVA[84] (version 1.28.0).

Public datasets

To create our curated, key functional signatures we extracted highly correlating genes from the data sets from cutaneous melanoma samples of The Cancer Genome Atlas (TCGA)[39] using the

Bioconductor R Package geneRecommender (version 1.52). TCGA mRNA expression data were extracted using cBioPortal's R interface using the CGDS-R package[85] (version 1.2.5). Survival analysis was performed using the survival package (version 2.42-6, https://github.com/therneau/survival).

We used the data from Riaz *et al.* to evaluate the effects of anti-PD-1 therapy on our signatures[40]. The TPM normalized expression values were downloaded from GEO (GSE91061). Signature expression levels were estimated using the ssGSEA approach and cell type abundances estimated using xCell (see above).

scRNA-seq from Sade-Feldman *et al.*[29] was directly downloaded from GEO as TPM normalized expression values (GSE120575). The complete data analysis was performed in R using Seurat 2.3.4[86]. The data was normalized using the “LogNormalize” function with a scale factor of 10,000. Variable genes were detected using the “LogVMR” dispersion function and the “ExpMean” function to calculate means. The x.low.cutoff was set to 0.0125, x.high.cutoff to 3, and the y.cutoff to 0.5. Next, the data was scaled regressing out the patient identifier. Out of 100 calculated principal components, the first 35 were used for further analysis. Cells were clustered using Seurath’s graph-based clustering algorithm based on the principal components using a resolution of 0.6. Subsequent cell types were annotated based on canonical markers and all B cell-like cells retained. B cells were again clustered using a resolution of 0.9. Clusters were annotated based on the found markers (function FindMarkers, min.pct set to 0.25), as well as the expression of canonical B cell markers. Signature expression levels were estimated using the ssGSEA function (see above) based on the average gene expressions. The complete workflow can be found in Supplementary File 1 as a Jupyter notebook.

Histology

Whole tissue sections were obtained from human melanoma metastases almost exclusively derived from cutaneous and subcutaneous sites. All tumor samples were obtained with informed patients' consent and retrieved from the pathology files of the Medical University of Vienna (ethics vote: 405/2006). Diagnosis was based on the review of hematoxylin and eosin-stained sections from formalin-fixed paraffin-embedded (FFPE) blocks by two authors of this study (PP, SNW), respectively. In selected tumor samples, S-100, HMB45 or triple CSPG/β3 integrin/HMB45 melanoma marker immunostainings were done to identify tumor cells and to confirm the diagnosis..

Multiplex IHC staining

4 µm sections from full FFPE blocks that were used to generate the TMA or from control tonsil tissue were utilized for both, the initial establishment of staining conditions for each individual primary antibody (Ab) and the successive optimization of multiplex staining. In a first step, primary Abs against the following antigens were established as single stains initially on human tonsil tissue and thereafter on metastatic human melanoma tumors (for antibodies see Supplementary Table 4).

In a second step, multiplex immunostains were established essentially as described[87,88]. Random integration of sequential Abs within a multiplex panel may lead to imbalanced signals, incomplete staining through interference with previously applied tyramide signal amplification (TSA), disruption of epitopes, and removal of TSA fluorophores because of repetitive antigen-retrievals at high temperature[87,88]. Therefore, each Ab was tested individually for its optimal position in the sequence of multiplex staining to minimize interference with previous Ab-TSA complexes or by alteration of epitopes.

For multiplex immunostains, 4 µm sections were deparaffinized and antigen retrieval was performed in heated citrate buffer (pH 6.0) and/or Tris-EDTA buffer (pH 9) for 30 min. Thereafter, sections were fixed with 7.5% neutralized formaldehyde (SAV Liquid Production GmbH). Each section was subjected to 6 successive rounds of Ab staining, each consisting of protein blocking with 20% normal goat serum (Dako) in PBS, incubation with primary Abs, biotinylated anti-mouse/rabbit secondary antibodies and Streptavidin-HRP (Dako), followed by TSA visualization with fluorophores Opal 520, Opal 540, Opal 570, Opal 620, Opal 650, and Opal 690 (PerkinElmer) diluted in 1X Plus Amplification Diluent (PerkinElmer), Ab-TSA complex-“stripping” in heated citrate buffer (pH 6.0) and/or Tris-EDTA buffer (pH 9) for 30 min and fixation with 7.5% neutralized formaldehyde. Thereafter, nuclei were counterstained with DAPI (PerkinElmer) and sections mounted with PermaFluor fluorescence mounting medium (Thermo Fisher Scientific).

Respective stainings without primary antibodies were used as a negative control. Along with tissue arrays, serial sections of melanoma specimens and normal controls were stained to assess reproducibility. At equal Ab-concentrations, TSA-based visualization is expected to yield a higher number of positive cells as compared to conventional immunofluorescence. We therefore established TSA-based visualization of primary Abs on control tonsil tissue, the golden standard for lymphocyte antigen detection in pathology, and performed a comparison for each Ab to validated staining patterns in human tonsil (as to the Human Protein Atlas[89], available from www.proteinatlas.org). Thereafter, we balanced the signal through dilution of the primary Abs to obtain staining levels and cell frequencies comparable to conventional immunofluorescence staining. The dilution of the CD19 antibody was optimized to allowing for the detection of $CD19^{low}$ plasma cell-like cells as compared to patterns and frequencies obtained by $CD138^{+}$ pooled IgA/IgG^{+} stainings on human tonsil and melanoma[14,16]. In multiplex stainings,

single primary Ab stainings were run in parallel to control for false positive results through incomplete Ab-TSA complex-"stripping" and false negative results through antigen masking (by incubation with multiple primary Abs, "umbrella-effect"). Spillover effects were controlled for anti-CD20-Ab stainings on tonsil with different Opal fluorophores by signal detection in adjacent components/channels and thereafter for exposure time settings upon acquisition of multiplex-stained tissue sections.

Tissue imaging, spectral unmixing and phenotyping

Multiplexed slides were scanned on a Vectra Multispectral Imaging System version 2 (Perkin Elmer) as described[87,88]. Briefly, a spectral library from spectral peaks emitted by each fluorophore from single stained slides was created with the Advanced Image Analysis software (InForm 2.4, PerkinElmer) and used for spectral unmixing of multispectral images allowing for identification of all markers of interest. Autofluorescence was determined on an unstained representative study sample.

TAB subset phenotyping in TMA and whole tumor sections: cells were phenotyped as (i) CD19$^+$ CD20$^-$ CD38$^+$ CD138$^-$ plasmablast-like, CD19$^+$ CD20$^-$ CD138$^+$ plasma cell-like, CD19$^+$ CD20$^+$ CD38$^-$ CD138$^-$ memory B cell-like, CD20$^+$ CD38$^+$ CD138$^-$ CD5$^-$ germinal center B cell-like, CD19$^+$ CD20$^-$ CD38$^-$ CD138$^-$ CD27$^+$ activated B cell-like, CD20$^+$ CD19$^-$ CD138$^-$ CD5$^+$ transitional cell-like TAB and (ii) other cells. The staining protocol has been optimized for detection of CD19. Though CD19^{low} plasma cell-like cells could be detected at significant numbers, they may still be underrepresented in our staining data. The same may also be true for the detection of activated B cell-like cells, as expression of CD27 has been reported to be downregulated on TAB[90,91]. Human melanoma cells can express CD38[92] and we observed some cores with slight CD38 immunoreactivity of melanoma cells. In these cores tumor cell areas were excluded with the

“exclusion area tool” of InForm. After adaptive cell segmentation, a selection of around 25 representative original multispectral images was used to set cut-off values for each fluorophore/antibody staining. All phenotyping and subsequent quantifications were performed blinded to the sample identity.

TAB and T cell subset distribution in intra- and extratumoral stromal tumor areas: was performed in whole tumor sections using the automated tissue segmentation tool of the InForm software (v.2.4, PerkinElmer), which had been trained to recognize intratumor and extratumoral stromal areas based on tissue morphology including the presence and absence, respectively, of melanoma cells. Total cell counts were determined by DAPI$^+$ cell numbers referred to the total tumor area, 6 color multiplex immunostaining (CD20, CD19, CD8, CD4, FoxP3, DAPI) was used for the detection of CD20$^+$CD19$^+$, CD20$^+$CD19$^-$, and CD20$^-$CD19$^+$ TAB and CD8$^+$, CD4$^+$FoxP3$^-$, and CD4$^+$FoxP3$^+$ cells. Further T cell subsets were identified by an additional 7 color multiplex immunostaining and phenotyped acc. to[93,94] as CD8$^+$CD4$^-$CD69$^+$CD103$^+$ and CD8$^-$CD4$^+$CD69$^+$CD103$^-$ cells corresponding to a tissue-resident memory T cell phenotype (T_{RM}); CD8$^+$CD4$^-$CD45RO$^+$CD27$^+$ and CD8$^-$CD4$^+$CD45RO$^+$CD27$^+$ cells corresponding to a central memory T cell phenotype (T_{CM}); CD8$^+$CD4$^-$CD45RO$^+$CD27$^-$ and CD8$^-$CD4$^+$CD45RO$^+$CD27$^-$ cells corresponding to an effector memory T cell phenotype (T_{EM}); and other CD8$^+$CD4$^-$ or CD8$^-$CD4$^+$ T cells.

Tertiary lymphoid structures (TLS) were identified acc. to Silina et al.[95] by a further 7 color multiplex immunostaining primary follicle-like TLS were identified as CD4$^+$ and CD20$^+$ lymphocyte aggregates with CXCL13 and CD21 but not CD23 expression; immature secondary follicle-like TLS by additional CD23 expression; and mature secondary follicle-like TLS by accessory expression of Bcl6. In on-anti-CD20 therapy samples, TLS were identified along the same criteria, without the presence of CD20 lymphocyte aggregates. Trainable tissue

segmentation was performed using the automated tissue segmentation tool of the InForm software (v.2.4, PerkinElmer). TLS areas (as determined by pixels) were read in whole tumor tissue sections and referred to the total tumor area (as determined by pixels).

References

1. Ascierto, P. A. *et al.* Survival Outcomes in Patients With Previously Untreated BRAF Wild-Type Advanced Melanoma Treated With Nivolumab Therapy: Three-Year Follow-up of a Randomized Phase 3 Trial. *JAMA Oncol* (2018). doi:10.1001/jamaoncol.2018.4514
2. de Visser, K. E., Korets, L. V. & Coussens, L. M. De novo carcinogenesis promoted by chronic inflammation is B lymphocyte dependent. *Cancer Cell* **7**, 411–423 (2005).
3. Ammirante, M., Luo, J.-L., Grivennikov, S., Nedospasov, S. & Karin, M. B-cell-derived lymphotoxin promotes castration-resistant prostate cancer. *Nature* **464**, 302–305 (2010).
4. Shalapour, S. *et al.* Immunosuppressive plasma cells impede T-cell-dependent immunogenic chemotherapy. *Nature* **521**, 94–98 (2015).
5. Affara, N. I. *et al.* B cells regulate macrophage phenotype and response to chemotherapy in squamous carcinomas. *Cancer Cell* **25**, 809–821 (2014).
6. Gunderson, A. J. *et al.* Bruton Tyrosine Kinase-Dependent Immune Cell Cross-talk Drives Pancreas Cancer. *Cancer Discov.* **6**, 270–285 (2016).
7. Shalapour, S. *et al.* Inflammation-induced IgA+ cells dismantle anti-liver cancer immunity. *Nature* **551**, 340–345 (2017).
8. Mauri, C. & Menon, M. Human regulatory B cells in health and disease: therapeutic potential. *J. Clin. Invest.* **127**, 772–779 (2017).
9. Liu, J. *et al.* Aberrant frequency of IL-10-producing B cells and its association with Treg and MDSC cells in Non Small Cell Lung Carcinoma patients. *Hum. Immunol.* **77**, 84–89 (2016).
10. Zhou, X., Su, Y.-X., Lao, X.-M., Liang, Y.-J. & Liao, G.-Q. CD19(+)IL-10(+) regulatory B cells affect survival of tongue squamous cell carcinoma patients and induce resting CD4(+) T cells to CD4(+)Foxp3(+) regulatory T cells. *Oral Oncol.* **53**, 27–35 (2016).
11. Ishigami, E. *et al.* Coexistence of regulatory B cells and regulatory T cells in tumor-infiltrating lymphocyte aggregates is a prognostic factor in patients with breast cancer. *Breast Cancer* (2018). doi:10.1007/s12282-018-0910-4
12. Wei, X. *et al.* Regulatory B cells contribute to the impaired antitumor immunity in ovarian cancer patients. *Tumour Biol.* **37**, 6581–6588 (2016).
13. Somasundaram, R. *et al.* Tumor-associated B-cells induce tumor heterogeneity and therapy resistance. *Nat. Commun.* **8**, 607 (2017).
14. Erdag, G. *et al.* Immunotype and immunohistologic characteristics of tumor-infiltrating

immune cells are associated with clinical outcome in metastatic melanoma. *Cancer Res.* **72**, 1070–1080 (2012).
15. Ladányi, A. Prognostic and predictive significance of immune cells infiltrating cutaneous melanoma. *Pigment Cell Melanoma Res.* **28**, 490–500 (2015).
16. Bosisio, F. M. *et al.* Plasma cells in primary melanoma. Prognostic significance and possible role of IgA. *Mod. Pathol.* **29**, 347–358 (2016).
17. Fremd, C., Schuetz, F., Sohn, C., Beckhove, P. & Domschke, C. B cell-regulated immune responses in tumor models and cancer patients. *Oncoimmunology* **2**, e25443 (2013).
18. Hooijkaas, A. *et al.* Selective BRAF inhibition decreases tumor-resident lymphocyte frequencies in a mouse model of human melanoma. *Oncoimmunology* **1**, 609–617 (2012).
19. Sumimoto, H., Imabayashi, F., Iwata, T. & Kawakami, Y. The BRAF-MAPK signaling pathway is essential for cancer-immune evasion in human melanoma cells. *J. Exp. Med.* **203**, 1651–1656 (2006).
20. Wang, Z. *et al.* Tumor-derived IL-35 promotes tumor growth by enhancing myeloid cell accumulation and angiogenesis. *J. Immunol.* **190**, 2415–2423 (2013).
21. Huang, A. *et al.* Interleukin-35 on B cell and T cell induction and regulation. *J. Inflamm.* **14**, 16 (2017).
22. Rosser, E. C. *et al.* Regulatory B cells are induced by gut microbiota-driven interleukin-1β and interleukin-6 production. *Nat. Med.* **20**, 1334–1339 (2014).
23. Ritvo, P.-G. & Klatzmann, D. Interleukin-1 in the Response of Follicular Helper and Follicular Regulatory T Cells. *Front. Immunol.* **10**, 250 (2019).
24. Winkler, J. K., Schiller, M., Bender, C., Enk, A. H. & Hassel, J. C. Rituximab as a therapeutic option for patients with advanced melanoma. *Cancer Immunol. Immunother.* **67**, 917–924 (2018).
25. Hugo, W. *et al.* Non-genomic and Immune Evolution of Melanoma Acquiring MAPKi Resistance. *Cell* **162**, 1271–1285 (2015).
26. Bindea, G. *et al.* Spatiotemporal dynamics of intratumoral immune cells reveal the immune landscape in human cancer. *Immunity* **39**, 782–795 (2013).
27. Hoesel, B. & Schmid, J. A. The complexity of NF-κB signaling in inflammation and cancer. *Mol. Cancer* **12**, 86 (2013).
28. Wu, G. & Haw, R. Functional Interaction Network Construction and Analysis for Disease Discovery. *Methods Mol. Biol.* **1558**, 235–253 (2017).
29. Sade-Feldman, M. *et al.* Defining T Cell States Associated with Response to Checkpoint Immunotherapy in Melanoma. *Cell* **175**, 998–1013.e20 (2018).
30. de Oliveira, C. E. C. *et al.* CC chemokine receptor 5: the interface of host immunity and cancer. *Dis. Markers* **2014**, 126954 (2014).
31. Bystry, R. S., Aluvihare, V., Welch, K. A., Kallikourdis, M. & Betz, A. G. B cells and professional APCs recruit regulatory T cells via CCL4. *Nat. Immunol.* **2**, 1126–1132 (2001).
32. Facciabene, A. *et al.* Tumour hypoxia promotes tolerance and angiogenesis via CCL28 and T(reg) cells. *Nature* **475**, 226–230 (2011).
33. Günther, C., Carballido-Perrig, N., Kaesler, S., Carballido, J. M. & Biedermann, T. CXCL16 and CXCR6 are upregulated in psoriasis and mediate cutaneous recruitment of human CD8+ T cells. *J. Invest. Dermatol.* **132**, 626–634 (2012).

34. Aran, D., Hu, Z. & Butte, A. J. xCell: digitally portraying the tissue cellular heterogeneity landscape. *Genome Biol.* **18**, 220 (2017).
35. Danaher, P. *et al.* Pan-cancer adaptive immune resistance as defined by the Tumor Inflammation Signature (TIS): results from The Cancer Genome Atlas (TCGA). *J Immunother Cancer* **6**, 63 (2018).
36. Ayers, M. *et al.* IFN-γ-related mRNA profile predicts clinical response to PD-1 blockade. *J. Clin. Invest.* **127**, 2930–2940 (2017).
37. Elia, A. R., Caputo, S. & Bellone, M. Immune Checkpoint-Mediated Interactions Between Cancer and Immune Cells in Prostate Adenocarcinoma and Melanoma. *Front. Immunol.* **9**, 1786 (2018).
38. Bolen, C. R. *et al.* Mutation load and an effector T-cell gene signature may distinguish immunologically distinct and clinically relevant lymphoma subsets. *Blood Adv* **1**, 1884–1890 (2017).
39. Cancer Genome Atlas Network. Genomic Classification of Cutaneous Melanoma. *Cell* **161**, 1681–1696 (2015).
40. Riaz, N. *et al.* Tumor and Microenvironment Evolution during Immunotherapy with Nivolumab. *Cell* **171**, 934–949.e16 (2017).
41. Pinc, A. *et al.* Targeting CD20 in melanoma patients at high risk of disease recurrence. *Mol. Ther.* **20**, 1056–1062 (2012).
42. Li, R., Patterson, K. R. & Bar-Or, A. Reassessing B cell contributions in multiple sclerosis. *Nat. Immunol.* **19**, 696–707 (2018).
43. Kessel, A. *et al.* Human CD19(+)CD25(high) B regulatory cells suppress proliferation of CD4(+) T cells and enhance Foxp3 and CTLA-4 expression in T-regulatory cells. *Autoimmun. Rev.* **11**, 670–677 (2012).
44. Parekh, V. V. *et al.* B Cells Activated by Lipopolysaccharide, But Not By Anti-Ig and Anti-CD40 Antibody, Induce Anergy in CD8+ T Cells: Role of TGF- 1. *The Journal of Immunology* **170**, 5897–5911 (2003).
45. Matsumoto, M. *et al.* Interleukin-10-producing plasmablasts exert regulatory function in autoimmune inflammation. *Immunity* **41**, 1040–1051 (2014).
46. Sun, C.-M., Deriaud, E., Leclerc, C. & Lo-Man, R. Upon TLR9 signaling, CD5+ B cells control the IL-12-dependent Th1-priming capacity of neonatal DCs. *Immunity* **22**, 467–477 (2005).
47. Iwata, Y. *et al.* Characterization of a rare IL-10-competent B-cell subset in humans that parallels mouse regulatory B10 cells. *Blood* **117**, 530–541 (2011).
48. Biswas, S. K. & Mantovani, A. Macrophage plasticity and interaction with lymphocyte subsets: cancer as a paradigm. *Nat. Immunol.* **11**, 889–896 (2010).
49. Mao, H. *et al.* CD19CD27 Plasmablasts Suppress Harmful Th17 Inflammation Through Interleukin 10 Pathway in Colorectal Cancer. *DNA Cell Biol.* **36**, 870–877 (2017).
50. Matsushita, T., Yanaba, K., Bouaziz, J.-D., Fujimoto, M. & Tedder, T. F. Regulatory B cells inhibit EAE initiation in mice while other B cells promote disease progression. *J. Clin. Invest.* **118**, 3420–3430 (2008).
51. Haas, K. M. *et al.* Protective and pathogenic roles for B cells during systemic autoimmunity in NZB/W F1 mice. *J. Immunol.* **184**, 4789–4800 (2010).

52. Distel, L. V. *et al.* Tumour infiltrating lymphocytes in squamous cell carcinoma of the oro- and hypopharynx: prognostic impact may depend on type of treatment and stage of disease. *Oral Oncol.* **45**, e167–74 (2009).
53. Mahmoud, S. M. A. *et al.* The prognostic significance of B lymphocytes in invasive carcinoma of the breast. *Breast Cancer Res. Treat.* **132**, 545–553 (2012).
54. Song, I. H. *et al.* Predictive Value of Tertiary Lymphoid Structures Assessed by High Endothelial Venule Counts in the Neoadjuvant Setting of Triple-Negative Breast Cancer. *Cancer Res. Treat.* **49**, 399–407 (2017).
55. Milne, K. *et al.* Systematic analysis of immune infiltrates in high-grade serous ovarian cancer reveals CD20, FoxP3 and TIA-1 as positive prognostic factors. *PLoS One* **4**, e6412 (2009).
56. Chee, S. J. *et al.* Evaluating the effect of immune cells on the outcome of patients with mesothelioma. *Br. J. Cancer* **117**, 1341–1348 (2017).
57. Zhu, Y., Yao, S. & Chen, L. Cell surface signaling molecules in the control of immune responses: a tide model. *Immunity* **34**, 466–478 (2011).
58. Chen, D. S. & Mellman, I. Elements of cancer immunity and the cancer-immune set point. *Nature* **541**, 321–330 (2017).
59. Kolesnikov, N. *et al.* ArrayExpress update--simplifying data submissions. *Nucleic Acids Res.* **43**, D1113–6 (2015).
60. Perez-Riverol, Y. *et al.* The PRIDE database and related tools and resources in 2019: improving support for quantification data. *Nucleic Acids Res.* (2018). doi:10.1093/nar/gky1106
61. Maurer, M. *et al.* Comprehensive comparative and semiquantitative proteome of a very low number of native and matched epstein-barr-virus-transformed B lymphocytes infiltrating human melanoma. *J. Proteome Res.* **13**, 2830–2845 (2014).
62. Maurer, M. *et al.* Combining filter-aided sample preparation and pseudoshotgun technology to profile the proteome of a low number of early passage human melanoma cells. *J. Proteome Res.* **12**, 1040–1048 (2013).
63. Fraussen, J. *et al.* A novel method for making human monoclonal antibodies. *J. Autoimmun.* **35**, 130–134 (2010).
64. Jutz, S. *et al.* A cellular platform for the evaluation of immune checkpoint molecules. *Oncotarget* **8**, 64892–64906 (2017).
65. Jalili, A. *et al.* Dual suppression of the cyclin-dependent kinase inhibitors CDKN2C and CDKN1A in human melanoma. *J. Natl. Cancer Inst.* **104**, 1673–1679 (2012).
66. Wiśniewski, J. R., Zougman, A., Nagaraj, N. & Mann, M. Universal sample preparation method for proteome analysis. *Nat. Methods* **6**, 359–362 (2009).
67. Batth, T. S., Francavilla, C. & Olsen, J. V. Off-line high-pH reversed-phase fractionation for in-depth phosphoproteomics. *J. Proteome Res.* **13**, 6176–6186 (2014).
68. Rappsilber, J., Mann, M. & Ishihama, Y. Protocol for micro-purification, enrichment, pre-fractionation and storage of peptides for proteomics using StageTips. *Nat. Protoc.* **2**, 1896–1906 (2007).
69. R Core Team. R: A Language and Environment for Statistical Computing. (2018).
70. Adusumilli, R. & Mallick, P. Data Conversion with ProteoWizard msConvert. *Methods Mol.*

Biol. **1550**, 339–368 (2017).
71. Kim, S. & Pevzner, P. A. MS-GF+ makes progress towards a universal database search tool for proteomics. *Nat. Commun.* **5**, 5277 (2014).
72. MacLean, B., Eng, J. K., Beavis, R. C. & McIntosh, M. General framework for developing and evaluating database scoring algorithms using the TANDEM search engine. *Bioinformatics* **22**, 2830–2832 (2006).
73. Breitwieser, F. P. *et al.* General statistical modeling of data from protein relative expression isobaric tags. *J. Proteome Res.* **10**, 2758–2766 (2011).
74. Gatto, L. & Lilley, K. S. MSnbase-an R/Bioconductor package for isobaric tagged mass spectrometry data visualization, processing and quantitation. *Bioinformatics* **28**, 288–289 (2012).
75. Ritchie, M. E. *et al.* limma powers differential expression analyses for RNA-sequencing and microarray studies. *Nucleic Acids Res.* **43**, e47 (2015).
76. Dobin, A. *et al.* STAR: ultrafast universal RNA-seq aligner. *Bioinformatics* **29**, 15–21 (2013).
77. McCarthy, D. J., Chen, Y. & Smyth, G. K. Differential expression analysis of multifactor RNA-Seq experiments with respect to biological variation. *Nucleic Acids Res.* **40**, 4288–4297 (2012).
78. Wu, D. & Smyth, G. K. Camera: a competitive gene set test accounting for inter-gene correlation. *Nucleic Acids Res.* **40**, e133 (2012).
79. Liberzon, A. *et al.* Molecular signatures database (MSigDB) 3.0. *Bioinformatics* **27**, 1739–1740 (2011).
80. Liberzon, A. *et al.* The Molecular Signatures Database (MSigDB) hallmark gene set collection. *Cell Syst* **1**, 417–425 (2015).
81. Shannon, P. *et al.* Cytoscape: a software environment for integrated models of biomolecular interaction networks. *Genome Res.* **13**, 2498–2504 (2003).
82. Merico, D., Isserlin, R., Stueker, O., Emili, A. & Bader, G. D. Enrichment map: a network-based method for gene-set enrichment visualization and interpretation. *PLoS One* **5**, e13984 (2010).
83. Barbie, D. A. *et al.* Systematic RNA interference reveals that oncogenic KRAS-driven cancers require TBK1. *Nature* **462**, 108–112 (2009).
84. Hänzelmann, S., Castelo, R. & Guinney, J. GSVA: gene set variation analysis for microarray and RNA-seq data. *BMC Bioinformatics* **14**, 7 (2013).
85. Cerami, E. *et al.* The cBio cancer genomics portal: an open platform for exploring multidimensional cancer genomics data. *Cancer Discov.* **2**, 401–404 (2012).
86. Butler, A., Hoffman, P., Smibert, P., Papalexi, E. & Satija, R. Integrating single-cell transcriptomic data across different conditions, technologies, and species. *Nat. Biotechnol.* **36**, 411 (2018).
87. Carstens, J. L. *et al.* Spatial computation of intratumoral T cells correlates with survival of patients with pancreatic cancer. *Nat. Commun.* **8**, 15095 (2017).
88. Gorris, M. A. J. *et al.* Eight-Color Multiplex Immunohistochemistry for Simultaneous Detection of Multiple Immune Checkpoint Molecules within the Tumor Microenvironment. *J. Immunol.* **200**, 347–354 (2018).
89. Uhlén, M. *et al.* Proteomics. Tissue-based map of the human proteome. *Science* **347**,

1260419 (2015).
90. Hegde, P. S., Karanikas, V. & Evers, S. The Where, the When, and the How of Immune Monitoring for Cancer Immunotherapies in the Era of Checkpoint Inhibition. *Clin. Cancer Res.* **22**, 1865–1874 (2016).
91. Nielsen, J. S. *et al.* CD20+ tumor-infiltrating lymphocytes have an atypical CD27- memory phenotype and together with CD8+ T cells promote favorable prognosis in ovarian cancer. *Clin. Cancer Res.* **18**, 3281–3292 (2012).
92. Morandi, F. *et al.* A non-canonical adenosinergic pathway led by CD38 in human melanoma cells induces suppression of T cell proliferation. *Oncotarget* **6**, 25602–25618 (2015).
93. Shin, H. & Iwasaki, A. Tissue-resident memory T cells. *Immunol. Rev.* **255**, 165–181 (2013).
94. Schenkel, J. M. & Masopust, D. Tissue-resident memory T cells. *Immunity* **41**, 886–897 (2014).
95. Siliņa, K. *et al.* Germinal Centers Determine the Prognostic Relevance of Tertiary Lymphoid Structures and Are Impaired by Corticosteroids in Lung Squamous Cell Carcinoma. *Cancer Res.* **78**, 1308–1320 (2018).
