# Supplementary material for: B cells sustain inflammation and predict response to immune checkpoint blockade in human melanoma
Source: Nat Commun. 2019 Sep 13;10:4186. doi: 10.1038/s41467-019-12160-2 (PMC6744450; doi:10.1038/s41467-019-12160-2)
Supplement: Supplementary file 8 — Supplementary Software 1 [file 41467_2019_12160_MOESM8_ESM.zip › 193518_1_supp_0_psxk8f.pdf]

B cells sustain inflammation and predict response to immune checkpoint blockade in human melanoma - Supplementary Materials

Johannes Griss[1,2,*], Wolfgang Bauer[1], Christine Wagner[1], Margarita Maurer-Granofszky[1§], Martin Simon[1], Minyi Chen[1], Peter Steinberger[3], Katharina Grabmeier-Pfistershammer[1,3], Florian Roka[1], Thomas Penz[4], Christoph Bock[4,5], Gao Zhang[6,#], Meenhard Herlyn[6], Katharina Glatz[7], Heinz Läubli[8], Kirsten D Mertz[9], Peter Petzelbauer[1], Thomas Wiesner[1], Markus Hartl[10], Winfried F Pickl[11], Rajasekharan Somasundaram[6], Stephan N Wagner[1*]

1 Department of Dermatology, Medical University of Vienna, 1090 Vienna, Austria
2 EMBL-European Bioinformatics Institute, Wellcome Trust Genome Campus, CB10 1SD Hinxton, Cambridge, United Kingdom
3 Division of Immune Receptors and T-cell Activation, Institute of Immunology, Center for Pathophysiology, Infectiology and Immunology, Medical University of Vienna
4 CeMM Research Center for Molecular Medicine of the Austrian Academy of Sciences, 1090 Vienna, Austria
5 Department of Laboratory Medicine, Medical University of Vienna, 1090 Vienna, Austria
6 Molecular & Cellular Oncogenesis Program and Melanoma Research Center, The Wistar Institute, Philadelphia, PA 19104-4265, USA
7 Institute of Pathology, University Hospital Basel, Basel, Switzerland, 4031
8 Division of Medical Oncology, University Hospital Basel, Basel, Switzerland, 4031
9 Institute of Pathology, Cantonal Hospital Baselland, Liestal, Switzerland, 4410
10 Mass Spectrometry Facility, Max F. Perutz Laboratories (MFPL), University of Vienna, Vienna BioCenter (VBC), 1030 Vienna, Austria
11 Division of Cellular Immunology and Immunohematology, Institute of Immunology, Center for Pathophysiology, Infectiology and Immunology, Medical University of Vienna
# Present address: Department of Neurosurgery & The Preston Robert Tisch Brain Tumor Center, Duke University Medical Center, Durham, NC 27710, USA
§ Present address: Children's Cancer Research Institute, 1090 Vienna, Austria

* Corresponding Authors:

Johannes Griss, johannes.griss@meduniwien.ac.at, Tel: +43 1 40400 77020

Stephan N. Wagner, stephan.wagner@meduniwien.ac.at, Tel: +43 1 40400 77020

Table of Contents

Table of Contents 2

Supplementary Figures 3

Supplementary Figure 1 3
Supplementary Figure 2 4
Supplementary Figure 3 5
Supplementary Figure 4 6
Supplementary Figure 5 7
Supplementary Figure 6 8
Supplementary Figure 7 9
Supplementary Figure 8 10
Supplementary Figure 9 12

Supplementary Methods 13

FACS Gating Strategy 13

Supplementary Figures

Supplementary Figure 1

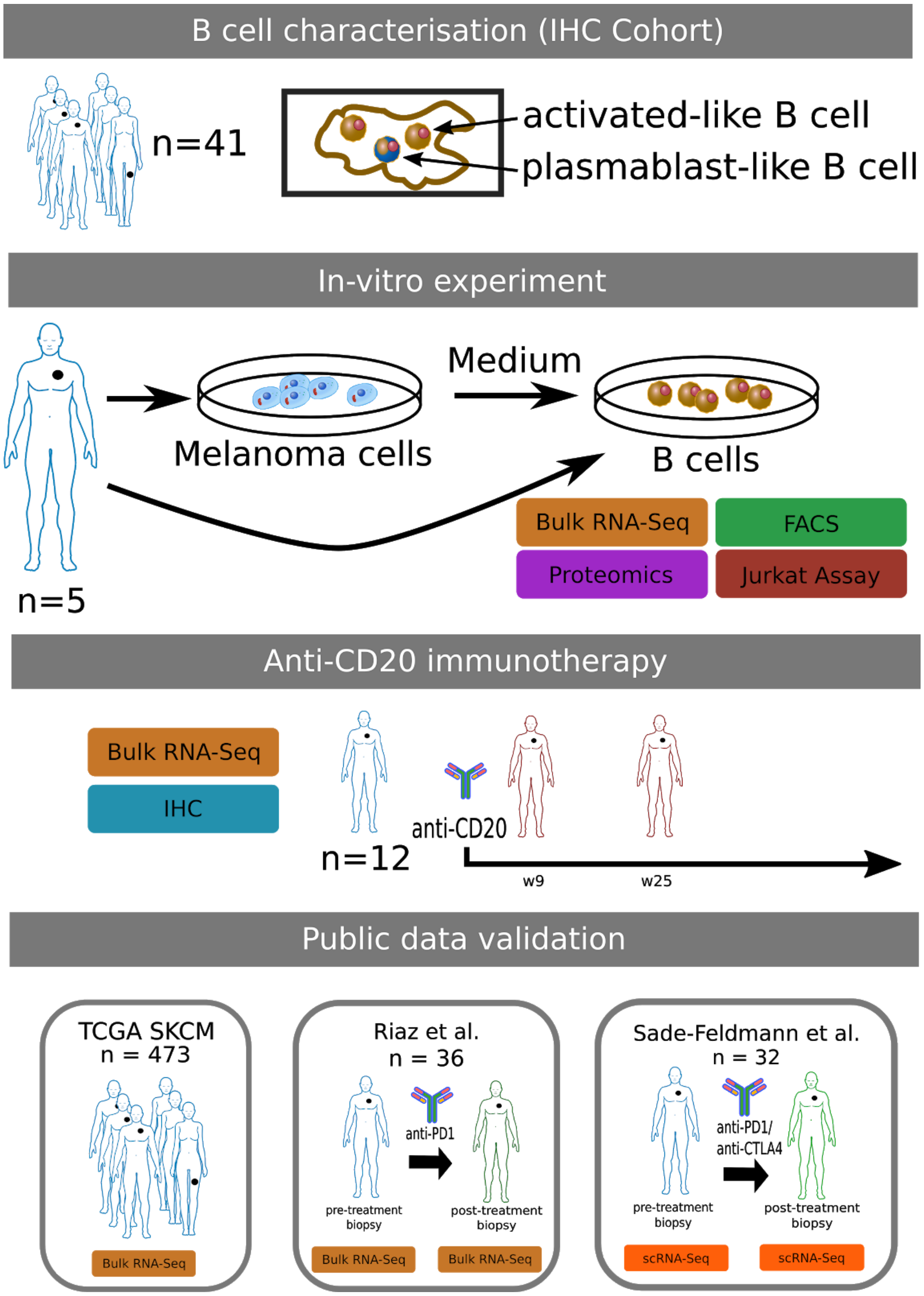

Patient cohorts evaluated in this study. Melanoma TAB phenotypes were characterised using whole tumor sections from 41 patients. B cell changes through soluble factors from melanoma cells were analysed in PBMC-and tumor-derived B cells from 5 patients and conditioned media from autologous melanoma cells. B cell-induced changes in the melanoma TME were analyzed in clinical samples from anti-CD20 immunotherapy trials performed by our group. Finally, all findings were validated using 3 public datasets: the TCGA skin cutaneous melanoma cohort, whole tissue RNA-seq data from melanoma patients pre- and on-anti-PD1 therapy and scRNA-seq data from melanoma lesions from two independent studies.

Supplementary Figure 2

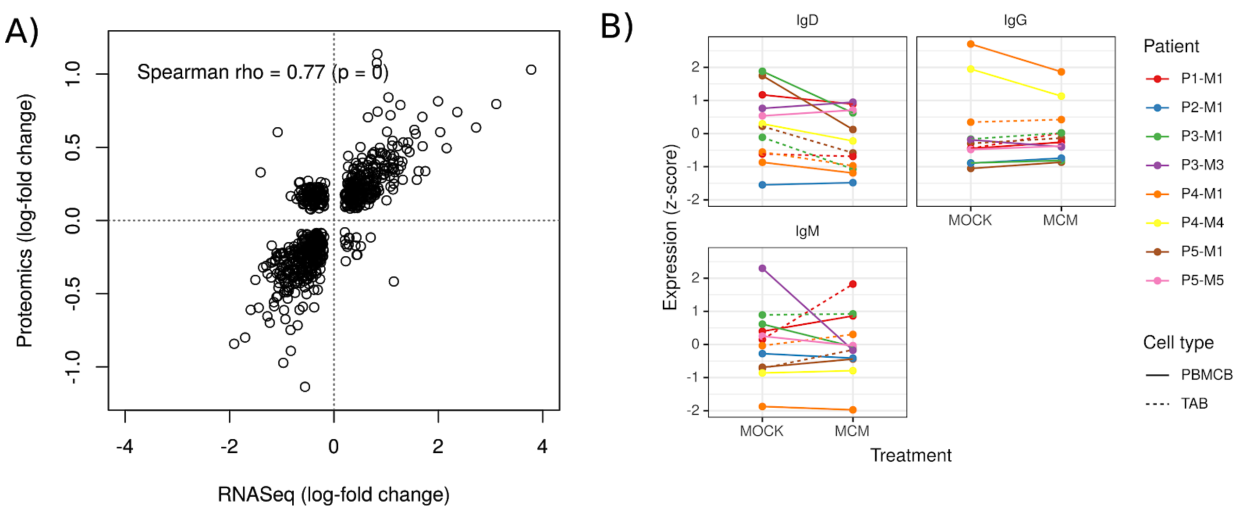

Induction experiments, significantly regulated gene/proteins and FACS data in B cells induced with melanoma conditioned medium. A) Fold changes (log2 transformed) of genes/proteins identified as significantly regulated showed a high correlation between proteomics and RNA-seq results. One group of proteins was consistently upregulated while the corresponding transcripts were downregulated (top-left corner). These all belong to proteins associated with metabolic pathways. **B)** Additional FACS-estimated expression levels of IgD, IgG, and IgM on peripheral blood B cells (PBMCB)- and tumour -derived B cells (TAB) by melanoma conditioned (MCM) and control (MOCK) medium.

Supplementary Figure 3

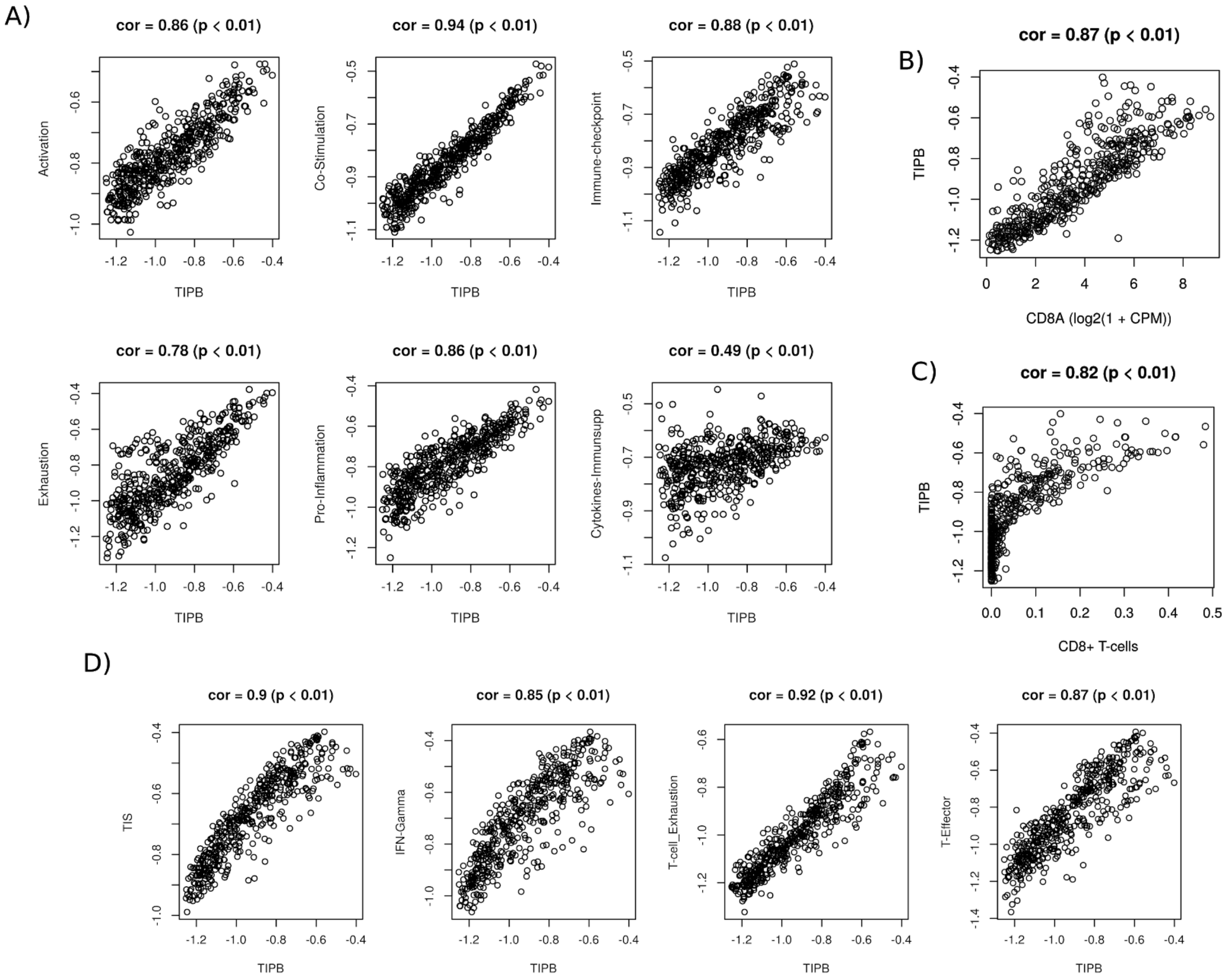

Validation of the TIPB and the functional signatures in the TCGA skin cutaneous melanoma cohort. A) Correlation of the functional signatures with the TIPB signature. **B)** Correlation of the TIPB signature with the expression of CD8A and **C)** with the xCell estimated abundance of $CD8^+$ T-cells. **D)** Correlation of the TIPB signature with established signatures describing the inflammation in the TME and T cell function and phenotype (tumor inflammatory score (TIS), interferon (IFN) gamma, T cell exhaustion, T cell effector (T-effector)) highly correlated with our TIPB signature. All correlation coefficients and p-values refer to the Spearman correlation coefficient.

Supplementary Figure 4

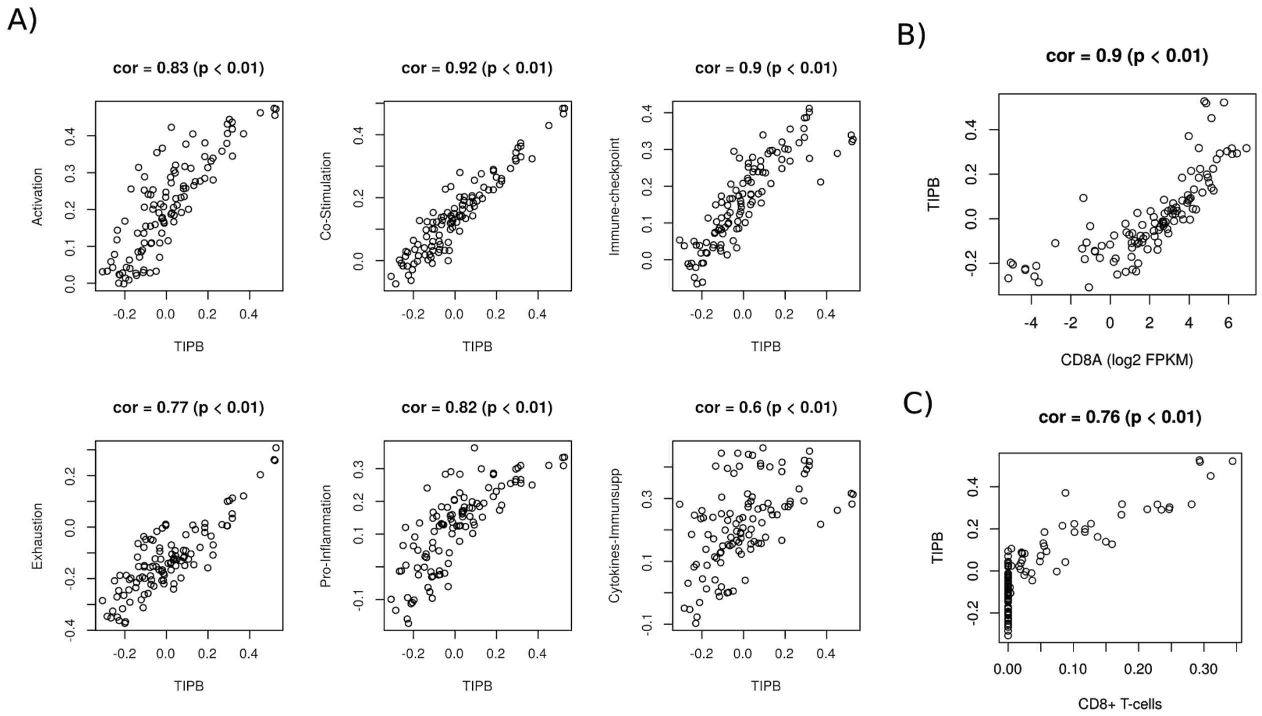

Validation of the TIPB and the functional signatures in a melanoma cohort treated with anti-PD1 (Riaz et al. dataset). A) Correlation of functional signatures as estimated by ssGSEA with the TIPB signature. **B)** Correlation of the TIPB signature with the expression of CD8A and **C)** the xCell estimated abundance of $CD8^{+}$ T cells.

Supplementary Figure 5

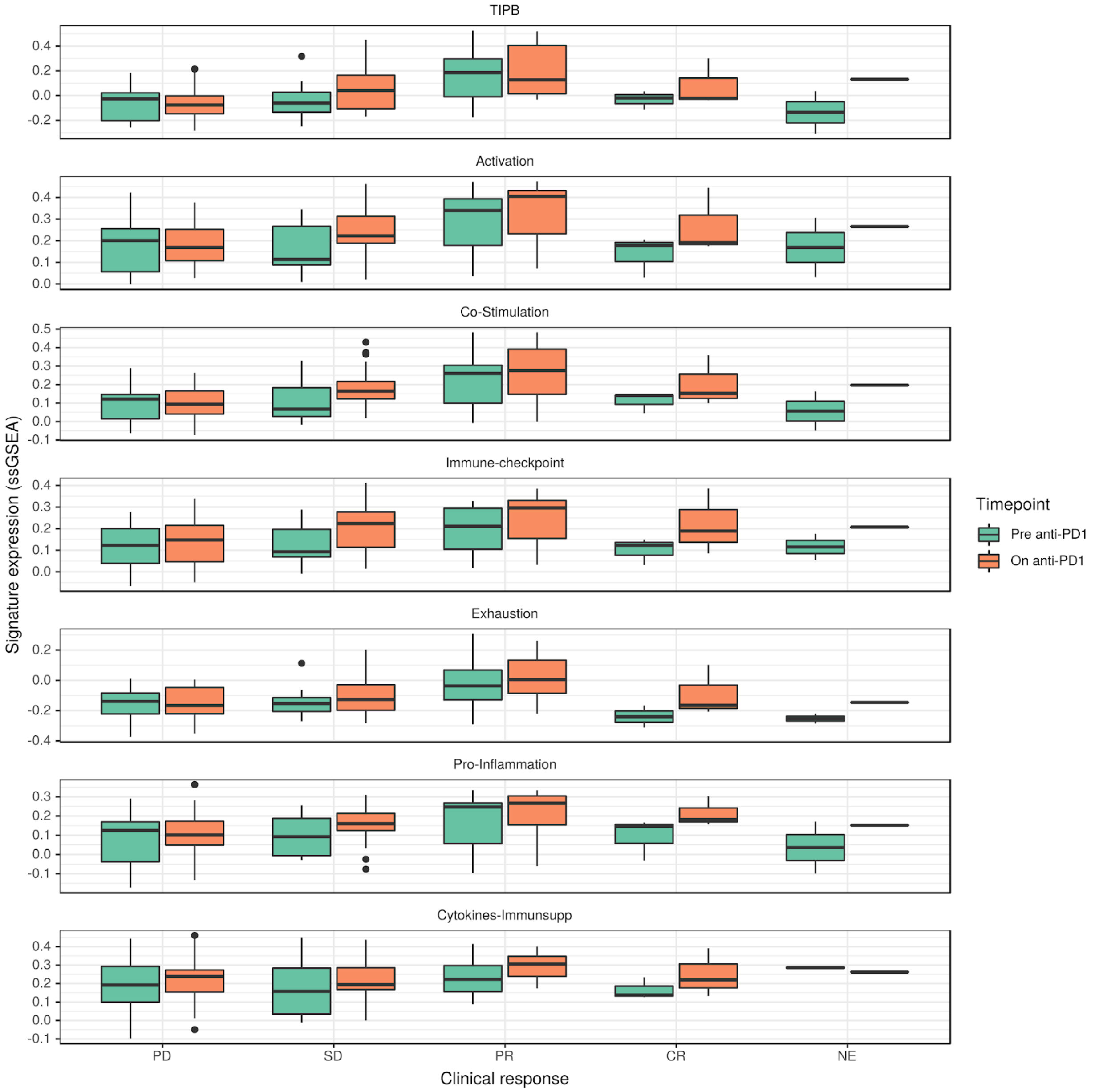

Expression of the TIPB signature and the functional signatures before and on anti-PD1 therapy versus clinical response (Riaz *et al.* dataset). (PD = progressive disease, SD = stable disease, PR = partial response, CR = complete response, NE = not evaluated)

Supplementary Figure 6

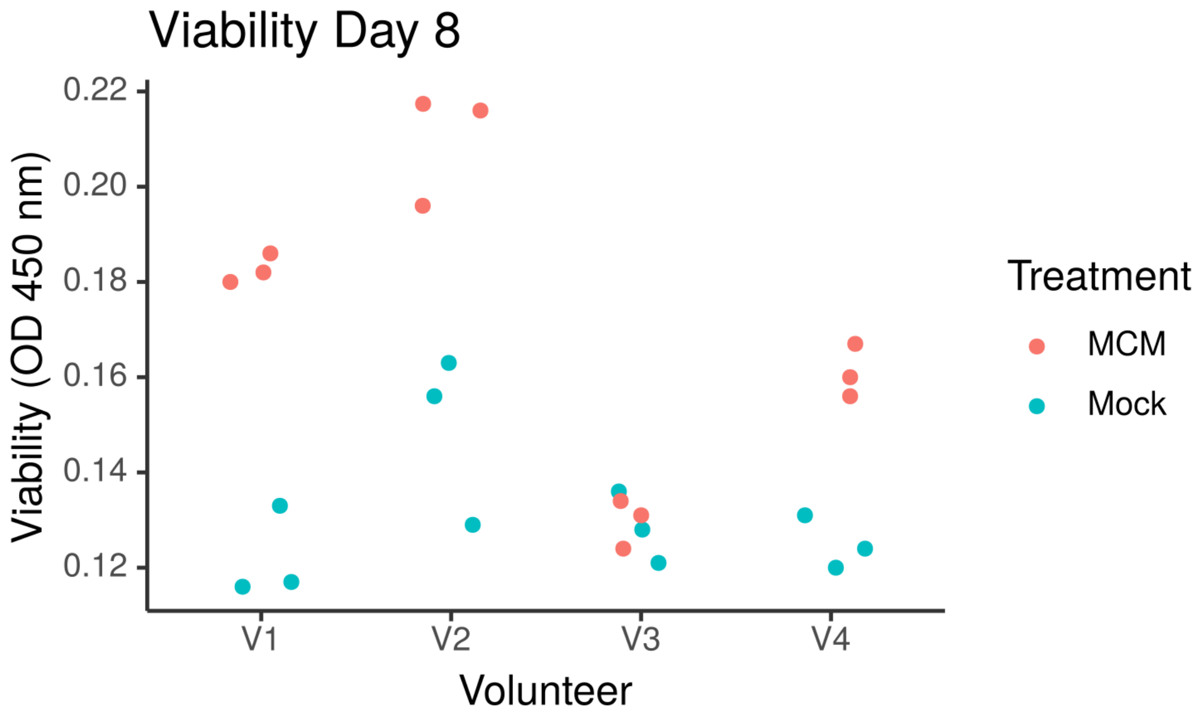

Viability of MCM and control medium conditioned B cells from four healthy volunteers. MCM led to a significant increase in B cell viability at day 8 (paired t-test, $p < 0.01$, $t = -4.8$, $df = 11$).

Supplementary Figure 7

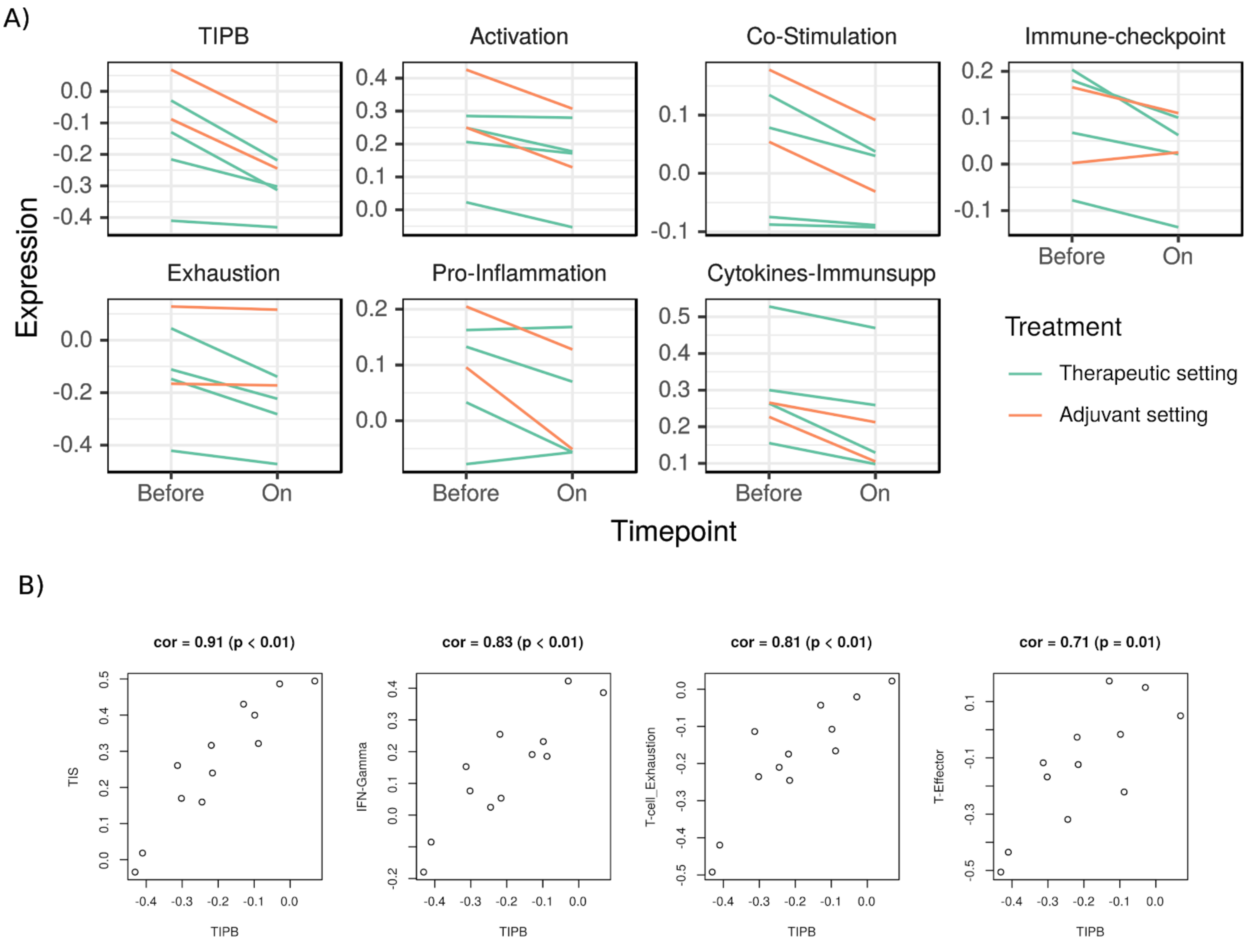

Validation of the TIPB and the functional signatures in the anti-CD20 clinical study samples. A) Estimated abundance (ssGSEA) of the TIPB signature and all functional signatures before and on anti-CD20 therapy. B) Correlation of established inflammation and T cell gene signatures with our TIPB signature.

Supplementary Figure 8

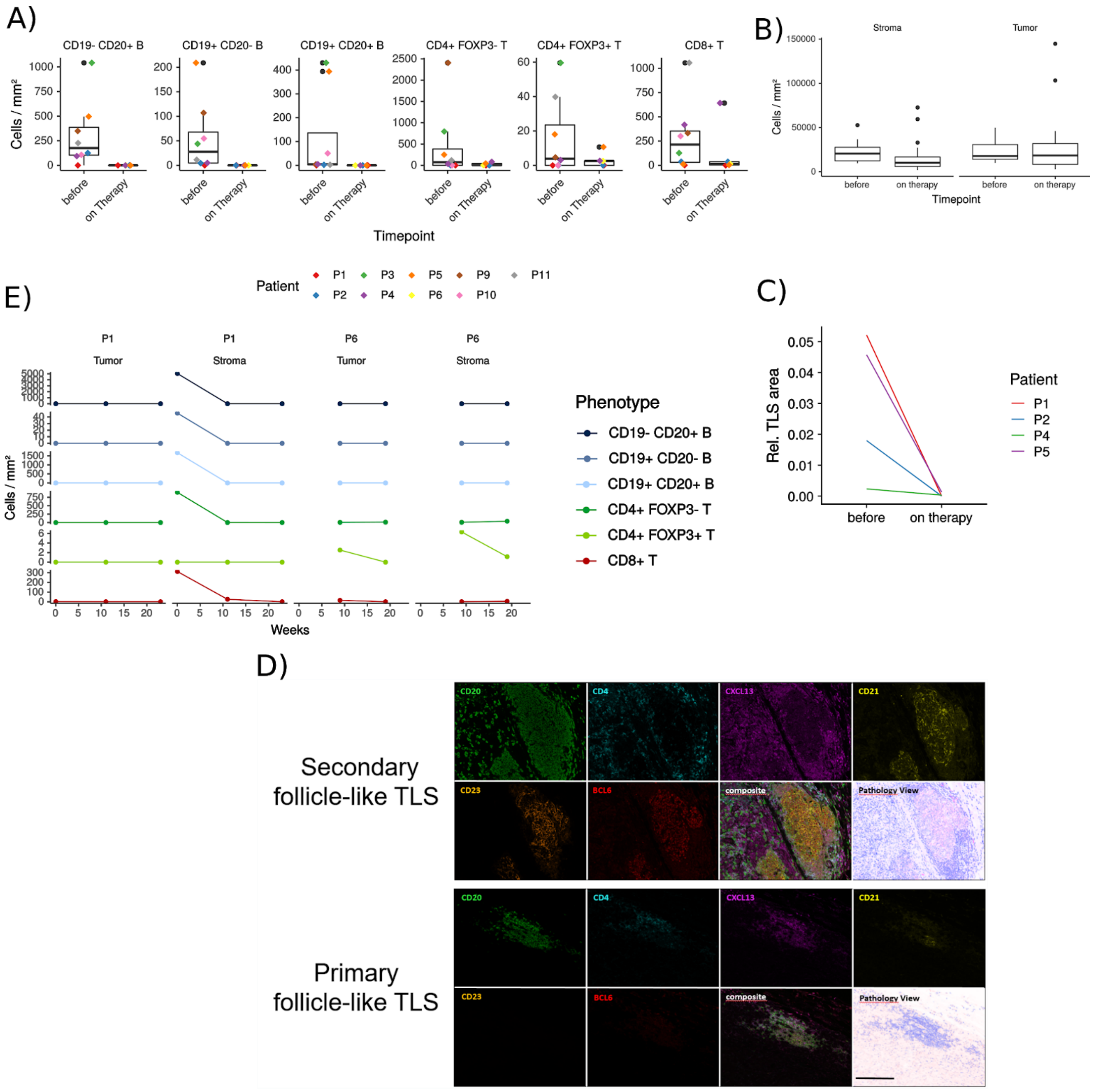

Quantification of immune cells using multiplex immunostainings. A) Intratumoral B and T cell numbers quantified using 6 color multiplex immunostaining in tumor samples from 9 patients obtained before and on therapy (at week 9±2). **B)** Total cell numbers as quantified by multiplex immunostainings of samples before and on therapy (at week 9±2). Cells were separately

quantified for the extratumoral stroma (“Stroma”) and intratumorally (“Tumor”). **C)** Relative area occupied by tertiary lymphoid structures (TLS) within metastases before and on anti-CD20 treatment as detected by 7 color multiplex immunostaining. **D)** Examples for both secondary mature and primary follicle-like TLS. Images for each of the individual markers and their composites are shown (for clarity without DAPI nuclear staining), together with the corresponding pathology view (respective bottom right). Note lack of for CD23 and Bcl6 immunoreactivity in primary follicle-like TLS. **E)** B and T cells quantified in two patients intratumorally (“Tumor”) and at the extratumoral stroma (“Stroma”) at two time points on therapy.

Supplementary Figure 9

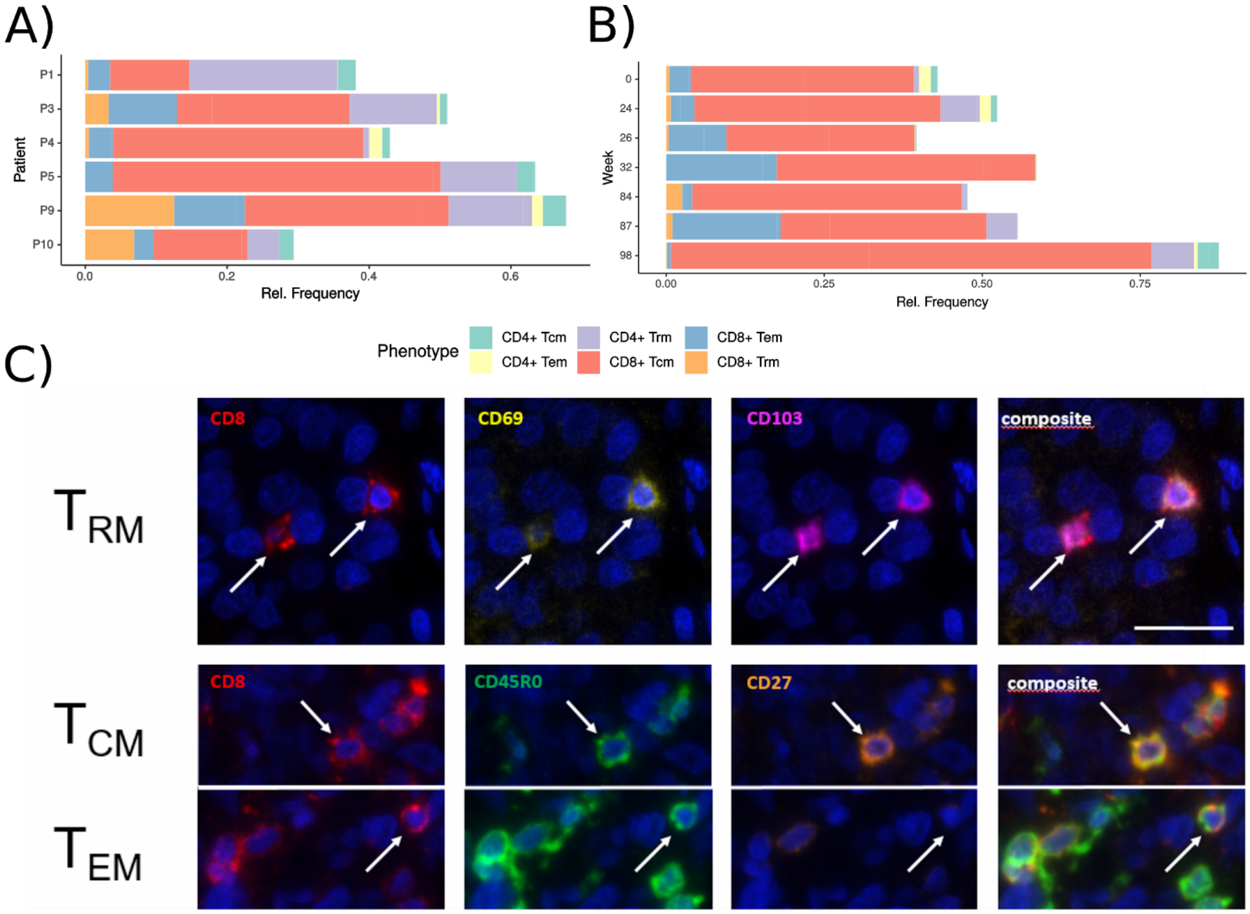

Quantification of T cell subtypes using multiplex immunostainings. A,B) Tissue-resident (T_{RM}), central (T_{CM}) and effector memory(T_{EM}) T cell subtypes quantified in samples from 6 patients before therapy (D) and a longitudinal analysis of tumor samples obtained over nearly two years (whenever a T cell infiltrate was present) in patient 4 (B). C) Examples for $CD8^+$ tissue-resident (T_{RM}), central (T_{CM}) and effector memory(T_{EM}) T cell subtypes from 7 color multiplex immunostaining. Composite images together with DAPI nuclear staining (right) and images for each of the individual markers used. Arrows depict representative cells. Scale bar represents 40µm.

Supplementary Methods

FACS Gating Strategy

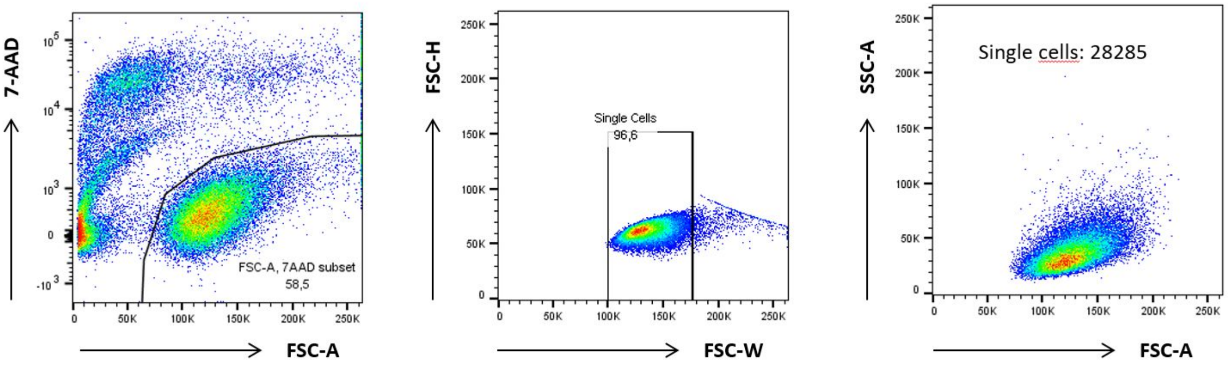

FACS staining of immortalized peripheral blood- and tumor-derived B cells: gating strategy for analysis of viable, single cells.

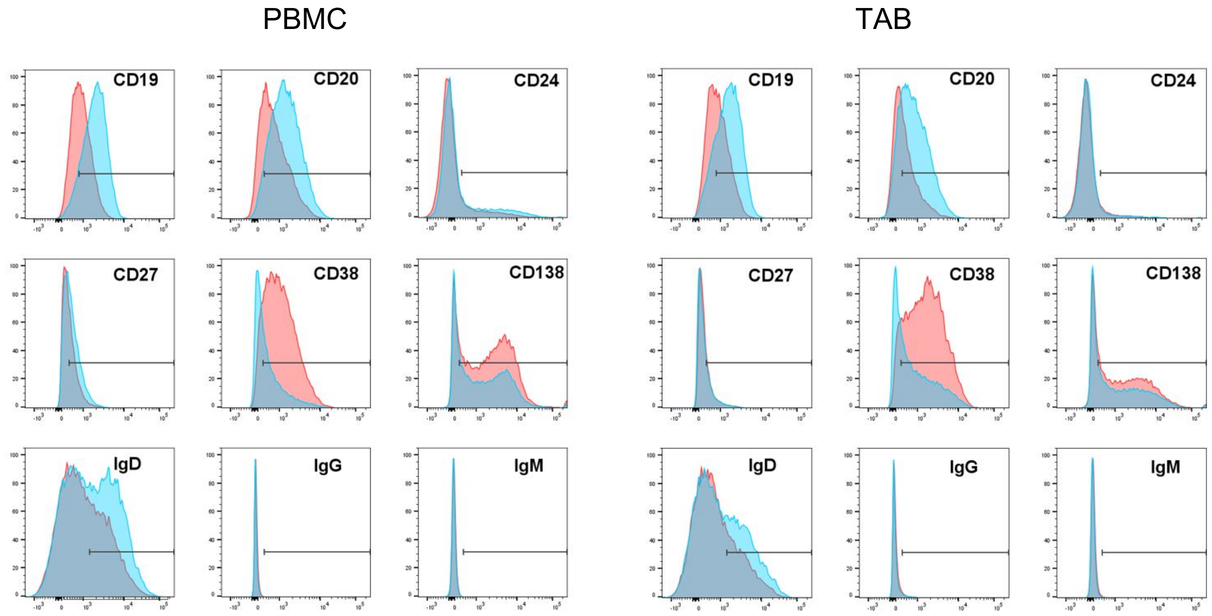

Representative FACS stainings of peripheral blood-derived immortalized B cells (PBMC) and

tumor-derived B cells (TAB) of one patient stimulated with control medium (blue histograms) or melanoma-conditioned medium (red histograms) for 48 hours. The gate represents the isotype defined positivity of the stainings.
