# Supplementary material for: B cells sustain inflammation and predict response to immune checkpoint blockade in human melanoma
Source: Nat Commun. 2019 Sep 13;10:4186. doi: 10.1038/s41467-019-12160-2 (PMC6744450; doi:10.1038/s41467-019-12160-2)
Supplement: Supplementary file 8 — Supplementary Software 1 [file 41467_2019_12160_MOESM8_ESM.zip › 193518_1_additional_review_material_0_pt1pcy.pdf]

nature research

Corresponding author(s): Johannes Griss (johannes.griss@meduniwien.ac.at)
Stephan N. Wagner (stephan.wagner@meduniwien.ac.at)

☒ Initial submission ☐ Revised version ☐ Final submission

Editorial Policy Checklist

This form is used to ensure compliance with Nature Research editorial policies related to research ethics and reproducibility. For further information, please see our Authors & Referees site. All relevant questions on the form must be answered.

ü Competing interests

Policy information about competing interests

Competing interests declaration

In the interest of transparency and to help readers form their own judgements of potential bias, Nature Research journals require authors to declare any competing financial and/or non-financial interest in relation to the work described in the submitted manuscript.

☒ No, I declare that the authors have no competing financial or non-financial interests as defined by Nature Research.

☐ Yes, I declare that the authors have a competing interest as defined by Nature Research

ü Data availability

Policy information about availability of data

Data availability statement

All manuscripts must include a data availability statement. This statement should provide the following information, where applicable:

- Accession codes, unique identifiers, or web links for publicly available datasets
- A list of figures that have associated raw data
- A description of any restrictions on data availability

☒ A full data availability statement is included in the manuscript.

Mandated accession codes (where applicable)

Confirm that all relevant data are deposited into a public repository and that accession codes are provided.

☒ All relevant accession codes are provided ☐ Accession codes will be available before publication ☐ No data with mandated deposition

ü Data presentation

Image integrity

☒ Confirm that all images comply with our image integrity policy.

Unprocessed data must be provided upon request. Please double-check figure assembly to ensure that all panels are accurate (e.g. all labels are correct, no inadvertent duplications have occurred during preparation, etc.).

Data distribution

Present data in a format that shows data distribution (dot-plots or box-and-whisker plots).
Define all box-plot elements (e.g. center line, median; box limits, upper and lower quartiles; whiskers, 1.5x interquartile range; points, outliers).
If using bar graphs, overlay the corresponding dot plots.

☒ Confirm that all data presentation meets these requirements and that individual data points are shown.

Specific policy considerations

Some types of research require additional policy disclosures. Please indicate whether these apply to your study. If you are not certain, please read the appropriate section before selecting a response.

Does not apply	Involved in the study	
☒	☐	Custom software or computer code
☒	☐	Macromolecular structural data
☒	☐	Research animals and/or animal-derived materials that require ethical approval
☐	☒	Human research participants
☐	☒	Clinical data

Human research participants

Policy information about studies involving human research participants

Ethical compliance

- [x] Confirm that you have complied with all relevant ethical regulations and that a statement affirming this is included in the manuscript.

Ethics committee

Confirm that the manuscript states the name(s) of the board and/or institution that:

- [x] Approved the study protocol -OR- [ ] Provided guidelines for study procedures (if protocol approval is not required)

Informed consent

- [x] Confirm that informed consent was obtained from all participants.

Identifiable images

For publication of identifiable images of research participants, confirm that consent to publish was obtained and is noted in the Methods.

Authors must ensure that consent meets the conditions set out in the Nature Research participant release form.

[ ] Yes [x] No identifiable images of human research participants

Clinical studies

Policy information about clinical studies

Clinical trial registration

- [x] Confirm that you have provided the trial registration number from ClinicalTrials.gov or an equivalent agency in the manuscript.

Phase 2 and 3 randomized controlled trials

Confirm that you have provided the CONSORT checklist with your submission.

[ ] Yes [ ] No [x] Not a phase 2/3 randomized controlled trial

Tumor marker prognostic studies

Did you follow the REMARK reporting guidelines?

[ ] Yes [ ] No [x] Not a tumor marker prognostic study

I certify that all the above information is complete and correct.

Typed signature Johannes Griss Date 22.11.2018

This checklist template is licensed under a Creative Commons Attribution 4.0 International License, which permits use, sharing, adaptation, distribution and reproduction in any medium or format, as long as you give appropriate credit to the original author(s) and the source, provide a link to the Creative Commons license, and indicate if changes were made. The images or other third party material in this article are included in the article's Creative Commons license, unless indicated otherwise in a credit line to the material. If material is not included in the article's Creative Commons license and your intended use is not permitted by statutory regulation or exceeds the permitted use, you will need to obtain permission directly from the copyright holder. To view a copy of this license, visit http://creativecommons.org/licenses/by/4.0/
