# Supplementary material for: B cells sustain inflammation and predict response to immune checkpoint blockade in human melanoma
Source: Nat Commun. 2019 Sep 13;10:4186. doi: 10.1038/s41467-019-12160-2 (PMC6744450; doi:10.1038/s41467-019-12160-2)
Supplement: Supplementary file 9 — Reporting Summary [file 41467_2019_12160_MOESM9_ESM.pdf]

nature research

Corresponding author(s): Johannes Griss (johannes.griss@meduniwien.ac.at), Stephan N Wagner (stephan.wagner@meduniwien.ac.at)

Reporting Summary

Nature Research wishes to improve the reproducibility of the work that we publish. This form provides structure for consistency and transparency in reporting. For further information on Nature Research policies, see Authors & Referees and the Editorial Policy Checklist.

Statistical parameters

When statistical analyses are reported, confirm that the following items are present in the relevant location (e.g. figure legend, table legend, main text, or Methods section).

n/a	Confirmed	
☐	☒	The exact sample size (*n*) for each experimental group/condition, given as a discrete number and unit of measurement
☐	☒	An indication of whether measurements were taken from distinct samples or whether the same sample was measured repeatedly
☐	☒	The statistical test(s) used AND whether they are one- or two-sided<br>*Only common tests should be described solely by name; describe more complex techniques in the Methods section.*
☐	☒	A description of all covariates tested
☐	☒	A description of any assumptions or corrections, such as tests of normality and adjustment for multiple comparisons
☐	☒	A full description of the statistics including central tendency (e.g. means) or other basic estimates (e.g. regression coefficient) AND variation (e.g. standard deviation) or associated estimates of uncertainty (e.g. confidence intervals)
☐	☒	For null hypothesis testing, the test statistic (e.g. *F*, *t*, *r*) with confidence intervals, effect sizes, degrees of freedom and *P* value noted<br>*Give P values as exact values whenever suitable.*
☐	☒	For Bayesian analysis, information on the choice of priors and Markov chain Monte Carlo settings
☐	☒	For hierarchical and complex designs, identification of the appropriate level for tests and full reporting of outcomes
☐	☒	Estimates of effect sizes (e.g. Cohen's *d*, Pearson's *r*), indicating how they were calculated
☐	☒	Clearly defined error bars<br>*State explicitly what error bars represent (e.g. SD, SE, CI)*

Our web collection on statistics for biologists may be useful.

Software and code

Policy information about availability of computer code

Data collection	FACS: FlowJo 10.4.2 (FlowJo LLC), Proteomics: ProteoWizard msConvert (version 3.0.9393), MSGF+ (version 10089), X!Tandem (version 2017.2.1.2), RNASeq: samtools (version 0.1.19), fastx_trimmer (0.0.14), STAR (2.5.3a), Histology/IHC: InForm (2.4, PerkinElmer)
Data analysis	All analysis were performed using R (3.5.1) and the pathway analysis using Cytoscape (3.5.1). The complete R scripts were submitted as supplementary file. Key R packages used: edgeR (3.22), Cytoscape EnrichmentMap plugin (3.1.0), xCell (1.1.0), GSVA (1.28.0), geneRecommender (1.52), survival (2.42-6), Rtsne (0.15), isobar (1.26), MSnbase (2.6.3), limma (3.35.3).

For manuscripts utilizing custom algorithms or software that are central to the research but not yet described in published literature, software must be made available to editors/reviewers upon request. We strongly encourage code deposition in a community repository (e.g. GitHub). See the Nature Research guidelines for submitting code & software for further information.

Data

Policy information about availability of data

All manuscripts must include a data availability statement. This statement should provide the following information, where applicable:

- Accession codes, unique identifiers, or web links for publicly available datasets
- A list of figures that have associated raw data
- A description of any restrictions on data availability

RNA-seq data have been deposited in the ArrayExpress database at EMBL-EBI (www.ebi.ac.uk/arrayexpress) under accession numbers E-MTAB-7472 (induction experiment) and E-MTAB-7473 (anti-CD20 study). The mass spectrometry proteomics data have been deposited to the ProteomeXchange Consortium via the PRIDE partner repository with the dataset identifier PXD011799 (induction experiment).

Field-specific reporting

Please select the best fit for your research. If you are not sure, read the appropriate sections before making your selection.

☒ Life sciences ☐ Behavioural & social sciences ☐ Ecological, evolutionary & environmental sciences

For a reference copy of the document with all sections, see nature.com/authors/policies/ReportingSummary-flat.pdf

Life sciences study design

All studies must disclose on these points even when the disclosure is negative.

Sample size	All available samples that were of sufficient quality for RNASeq analysis were used for this study. This resulted in 4 matched pairs from the Ofatumumab study and 2 matched pairs from the Rituximab study. Additionally, all available samples histological samples were used to the IHC-based quantitative analysis.
Data exclusions	No data was excluded from the analysis.
Replication	Only biological replicates were used. Due to the limited amount of material available, no technical replicates were performed.
Randomization	In the two pilot studies, treatment effects of anti-CD20 antibodies were studied. All included patients received the respective treatment and no randomization was performed as per the study design.
Blinding	Since both studies only contained one treatment group, no blinding was performed.

Reporting for specific materials, systems and methods

Materials & experimental systems

n/a	Involved in the study
☒	☐ Unique biological materials
☐	☒ Antibodies
☒	☐ Eukaryotic cell lines
☒	☐ Palaeontology
☒	☐ Animals and other organisms
☐	☒ Human research participants

Methods

n/a	Involved in the study
☒	☐ ChIP-seq
☐	☒ Flow cytometry
☒	☐ MRI-based neuroimaging

Antibodies

Antibodies used

FACS Aria III (BD): CD19 BV711 (clone SJ25C1, catalogue number 563036), CD20 AF700 (clone 2H7, 560631), CD24 PE-CF594 (clone ML5, 562405), CD27 BV421 (clone M-T271, 562513), CD38 APC (clone HIT2, 555462), CD138 PE (clone MI15, 552026), IgD PE-Cy7 (clone IA6-2, 561314), IgG FITC (clone G18-145, 555786), IgM BV605 (clone G20-127, 562977) (all BD biosciences).

Histology: CD19 (Rabbit monoclonal, clone EPR5906, 1:500, Abcam, catalogue number ab134114), CD20 (Mouse monoclonal, clone L26 , 1:2000, Dako, M0755), CD27 (Rabbit monoclonal, clone EPR8569, 1:500, Abcam, ab131254), CD38 (Mouse monoclonal, clone AT13/5, 1:1350, Dako, F7101), CD138 (mouse monoclonal, clone MI15, 1:1350, Agilent, M7228), CD5 (Mouse monoclonal, clone 4C7, 1:600, Novocastra, NCL-L-CD5-4C7), IL-10 (Rabbit polyclonal IgG, 1:800, Proteintech, 20850-1-AP), CD8 (Mouse monoclonal, clone C8/144B, 1:900, Biocare Medical, ACI 3160C), FoxP3 (Rabbit monoclonal, clone D2W8E, 1:400, Cell Signaling Technology, 98377S), CD4 (Mouse monoclonal, clone 4B12, 1:500, Dako, M7310), CD69 (Rabbit polyclonal, HPA050525, 1:100, Sigma-Aldrich, HPA050525), CD103 (Rabbit monoclonal, clone EPR4166(2), 1:4000, Abcam, ab129202), CD45RO (Mouse monoclonal, clone UCHL1, 1:2500, Dako, M0742), CXCL13 (Rabbit polyclonal IgG, 1:1800, Proteintech, 10927-1-AP), CD21 (Rabbit polyclonal IgG, 1:3600, Proteintech, 24374-1-AP), CD23 (Rabbit monoclonal, clone SP23, 1:900, Novus Biologicals, NB120-16702), Bcl6 (Mouse monoclonal, clone 1E6B1, 1:9000, Proteintech, 66340-1-Ig).

Validation

IHC Validation:

Respective stainings without primary antibodies were used as a negative control. Along with tissue arrays, serial sections of melanoma specimens and normal controls were stained to assess reproducibility. At equal Ab-concentrations, TSA-based visualization is expected to yield a higher number of positive cells as compared to conventional immunofluorescence. We therefore established TSA-based visualization of primary Abs on control tonsil tissue, the golden standard for lymphocyte antigen detection in pathology, and performed a comparison for each Ab to validated staining patterns in human tonsil (as to the Human Protein Atlas, available from www.proteinatlas.org). Thereafter, we balanced the signal through dilution of the primary Abs to obtain staining levels and cell frequencies comparable to conventional immunofluorescence staining. The dilution of the CD19 antibody was optimized to allowing for the detection of CD19low plasma cell-like cells as compared to patterns and frequencies obtained by CD138+ pooled IgA/IgG+ stainings on human tonsil and melanoma. In multiplex stainings, single primary Ab stainings were run in parallel to control for false positive results through incomplete Ab-TSA complex-"stripping" and false negative results through antigen masking (by incubation with multiple primary Abs, "umbrella-effect"). Spillover effects were controlled for anti-CD20-Ab stainings on tonsil with different Opal fluorophores by signal detection in adjacent components/channels and thereafter for exposure time settings upon acquisition of multiplex-stained tissue sections.

Human research participants

Policy information about studies involving human research participants

Population characteristics

Characteristics are summarized in Supp Table 5 (including prior therapies).

Recruitment

10 patients with metastatic melanoma were treated with the anti-CD20 antibody ofatumumab in a therapeutic setting13,59 (Clinical trials number ClinicalTrials.gov NCT01376713). Baseline demographic and clinical characteristics of these patients as well as response data were given in ref13, additional clinical data are shown in Supplementary Table 1). Patients had to have adequate biopsy material for (i) multiplex immunohistochemistry (IHC) staining and/or (ii) RNA-seq analysis from time points before initiation of therapy (week 0) and/or under anti-CD20 therapy (week 9±2, with additional later samples as indicated for some patients), when immunophenotyping of peripheral blood mononuclear cells showed a consistent loss of CD19+ B lymphocytes (week 2 and later). From 9 patients formalin-fixed paraffin-embedded (FFPE) tumor biopsies could be subjected to multiplex IHC, RNA-seq data were successfully generated from matched samples of four patients. We also collected pre-therapy peripheral blood mononuclear cell (PBMC) samples for generation of EBV-immortalized peripheral B cells13,59 as well as adequate pre-therapy tumor biopsy material for generation of (i) early passage melanoma cells59,60 and (ii) autologous EBV-immortalized TAB13,59 from tumor single cell suspensions. From four patients all these samples were successfully established, from a further patient EBV-immortalized peripheral B cells as well as EBV-immortalized TAB could be established. To increase the number of patients with pre-therapy early passage melanoma cells and autologous EBV-immortalized peripheral B cells and TAB, we included one further patient who was considered for but did not undergo anti-CD20 therapy.

In addition, of two patients with metastatic melanoma who were treated with the anti-CD20 antibody rituximab in an adjuvant setting41 (EudraCT number: 2007-005125-30), adequate tumor biopsy material for RNA-seq was available at two time points: before initiation of anti-CD20 therapy, when the tumor mass was completely resected, and after disease progression under therapy. Both studies were investigator-initiated clinical pilot trials. Anti-CD20 antibodies (Ofatumumab vs. Rituximab) were chosen based on availability. Samples were collected under local ethics committee-approved protocols (ethical review board of the Medical University of Vienna, votes 457-2007, 969-2010) with informed patient consent. Separate ethics committee-approval was obtained to make the derived proteomics and RNA-seq data publicly available (ethical review board of the Medical University of Vienna, vote 1555-2016). Clinical details are summarized in Supplementary Table 1.

Flow Cytometry

Plots

Confirm that:

- [x] The axis labels state the marker and fluorochrome used (e.g. CD4-FITC).
- [x] The axis scales are clearly visible. Include numbers along axes only for bottom left plot of group (a 'group' is an analysis of identical markers).
- [x] All plots are contour plots with outliers or pseudocolor plots.
- [x] A numerical value for number of cells or percentage (with statistics) is provided.

Methodology

Sample preparation	FACS analysis was performed on peripheral and tumor invading human B cells
Instrument	FACS Aria III (BD bioscience)
Software	FlowJo 10.4.2 (FlowJo LLC)
Cell population abundance	FACS analysis was only performed on cultivated immortalized cells.
Gating strategy	Gating strategy is shown in Supplementary Methods

- [x] Tick this box to confirm that a figure exemplifying the gating strategy is provided in the Supplementary Information.
