# Supplementary material for: B cells sustain inflammation and predict response to immune checkpoint blockade in human melanoma
Source: Nat Commun. 2019 Sep 13;10:4186. doi: 10.1038/s41467-019-12160-2 (PMC6744450; doi:10.1038/s41467-019-12160-2)
Supplement: Supplementary file 8 — Supplementary Software 1 [file 41467_2019_12160_MOESM8_ESM.zip › 193518_1_other_0_psz9cz.zip!/Whole tissue signature validation.html]

Whole tissue signature validation


# Signature Validation Analysis¶

This Jupyter notebook contains the code used to validate the functional tumour-associated B-cell (TAB) signatures in public datasets.

## Signatures¶

The signatures describe A) a plasmablast-like TAB phenotype and B) various functional signatures found induced in TAB through melanoma-conditioned medium.

In [1]:

```
library(ggplot2)
library(RColorBrewer)
library(reshape2)

org.options <- options()

my_theme_bw <- theme_bw() +
    theme(strip.background = element_blank(),
          panel.border = element_rect(colour = "black"))

# add some convenience functions
setPlotSize <- function(height, width) {
    options(repr.plot.height=height, repr.plot.width = width)
}

resetPlotSize <- function() {
    options(org.options)
}

# if set to true, SVG plots are created in the "plots" subdirectory
createPlots <- F

if (!file.exists("plots")) {
    message("Created plots directory")
    dir.create("plots")
}

if (!file.exists("cache")) {
    message("Created cache directory")
    dir.create("cache")
}
```

In [2]:

```
signatures <- list(
    "TIPB" = c("CD38", "CD27", "PAX5"),
    # Functional signatures
    "Activation" = c("CD69", "SELPLG", "CD72", "CFLAR", "FGFR1", "CD86"),
    "Co-Stimulation" = c("ICAM3", "TNFRSF13C", "CD40", "CD72", "C3",
                         "CD80", "CD86", "CD27", "CD28", "ICOS", "TNFRSF9",
                         "CD40LG", "ARHGDIB" ),
    "Immune-checkpoint" = c("CD274", "PDCD1LG2", "TNFRSF14", "LGALS9",
                            "BTLA", "LAG3", "HAVCR2", "ADORA2A"),
    "Exhaustion" = c("PDCD1", "FCRL4", "SIGLEC6", "CD22"),
    "Pro-Inflammation" = c("TNF", "IL12B", "IL18", "LTA", "TNFAIP2", "C3", "HCK"),
    "Cytokines-Immunsupp" = c("IL10", "TGFB1"))
```

# TCGA-SKCM cohort¶

This analysis evaluates the expression of our signatures with the samples found in the TCGA melanoma cohort.

## Download data¶

**Note**: This cell is only run once and the resulting read counts and clinical information stored in the files `mel.sample.[..].rds` and `mel.clinical.indexed.rds`. For the paper, the data was retrieved in October 2018.

In [ ]:

```
library(SummarizedExperiment)
library(TCGAbiolinks)

# download raw counts
query <- GDCquery(project = c("TCGA-SKCM"),
                  data.category = "Transcriptome Profiling",
                  legacy = FALSE,
                  workflow.type = "HTSeq - Counts"
)
query.result <- data.frame(getResults(query))
saveRDS(query.result, file = "cache/mel.sample.counts.rds")

# download FPKM normalized
query <- GDCquery(project = c("TCGA-SKCM"),
                  data.category = "Transcriptome Profiling",
                  legacy = FALSE,
                  workflow.type = "HTSeq - Counts"
)
query.result <- data.frame(getResults(query))
saveRDS(query.result, file = "cache/mel.sample.fpkm")

# get the clinical information
clinical <- GDCquery_clinic(project = "TCGA-SKCM", type = "clinical")
saveRDS(clinical, file = "cache/mel.clinical.indexed.rds")
```

Download the actual count expression values:

In [ ]:

```
library(TCGAbiolinks)
library(SummarizedExperiment)
library(edgeR)

# download RNASeq data
query <- GDCquery(
    project = "TCGA-SKCM",
    data.category = "Transcriptome Profiling",
    data.type = "Gene Expression Quantification",
    workflow.type = "HTSeq - Counts"
)

GDCdownload(query)

mel.counts <- GDCprepare(query)

counts <- assay(mel.counts)
pat.data <- colData(mel.counts)
gene.data <- rowData(mel.counts)

# TODO: Adapt this to the melanoma samples
pat.group <- factor(colData(mel.rnaseq)$definition)

mel.dge <- DGEList(counts = counts, 
                    samples = pat.data, 
                    genes = gene.data, 
                    group = pat.group)

mel.dge <- calcNormFactors(mel.dge)
design <- model.matrix(~ pat.group)
mel.dge <- estimateDisp(mel.dge, design)

saveRDS(mel.dge, file = "cache/mel.dge.rds")
```

Download the FPKM data:

In [ ]:

```
library(TCGAbiolinks)
library(SummarizedExperiment)
library(edgeR)

# download RNASeq data
query <- GDCquery(
    project = "TCGA-SKCM",
    data.category = "Transcriptome Profiling",
    data.type = "Gene Expression Quantification",
    workflow.type = "HTSeq - FPKM"
)

GDCdownload(query)

mel.fpkm <- GDCprepare(query)

fpkm <- assay(mel.fpkm)

saveRDS(fpkm, file = "cache/mel.fpkm.rds")
```

## Load data¶

Change the rownames to Gene identifiers. For duplicate entries, keep the one with the highest overall expression:

In [3]:

```
# change to gene
mel.dge <- readRDS("cache/mel.dge.rds")

gene.counts <- rowSums(mel.dge$counts)
mel.dge <- mel.dge[order(gene.counts, decreasing = T), ]
mel.dge <- mel.dge[!duplicated(mel.dge$genes$external_gene_name) & !is.na(mel.dge$genes$external_gene_name), ]
rownames(mel.dge) <- mel.dge$genes$external_gene_name

head(mel.dge$genes)
```

```
Loading required package: edgeR
Loading required package: limma
```

|  | ensembl\_gene\_id | external\_gene\_name | original\_ensembl\_gene\_id |
| --- | --- | --- | --- |
| PMEL | ENSG00000185664 | PMEL | ENSG00000185664.13 |
| ACTB | ENSG00000075624 | ACTB | ENSG00000075624.12 |
| GAPDH | ENSG00000111640 | GAPDH | ENSG00000111640.13 |
| ACTG1 | ENSG00000184009 | ACTG1 | ENSG00000184009.8 |
| EEF1A1 | ENSG00000156508 | EEF1A1 | ENSG00000156508.16 |
| IGHG1 | ENSG00000211896 | IGHG1 | ENSG00000211896.5 |

Filter out any genes that were observed in less than 30% of the samples with at least 10 reads:

In [4]:

```
min.counts <- 10
min.rel.patients <- 0.3

mel.rel.in.patients <- rowSums(mel.dge$counts >= min.counts) / ncol(mel.dge)
mel.dge.filtered <- mel.dge[mel.rel.in.patients >= 0.3, ]

message("Removed ", nrow(mel.dge) - nrow(mel.dge.filtered), 
        " genes observed in less than ", min.rel.patients, 
        " of the patients with ", min.counts, " read counts")
message(nrow(mel.dge.filtered), " genes retained")
```

```
Removed 34332 genes observed in less than 0.3 of the patients with 10 read counts
20899 genes retained
```

Repeat filtering and changing to gene identifiers for FPKM data:

In [5]:

```
mel.fpkm <- readRDS("cache/mel.fpkm.rds")

# match the mel.fpkm genes to the mel.cpm list
gene.mappings <- as.character(mel.dge.filtered$genes$external_gene_name)
names(gene.mappings) <- mel.dge.filtered$genes$ensembl_gene_id

mel.fpkm <- data.frame(mel.fpkm)
mel.fpkm$gene <- gene.mappings[rownames(mel.fpkm)]
mel.fpkm <- mel.fpkm[!is.na(mel.fpkm$gene),]
rownames(mel.fpkm) <- mel.fpkm$gene
mel.fpkm$gene <- NULL
rm(gene.mappings)

# use the same sorting of samples and genes
colnames(mel.fpkm) <- gsub("\\.", "-", colnames(mel.fpkm))
mel.fpkm <- mel.fpkm[rownames(mel.dge), colnames(mel.dge)]
```

## Quantify signature expression¶

Use the `ssGSEA` algorithm to estimate the expression of our signatures within the samples:

In [6]:

```
library(GSVA)

mel.sig <- gsva(expr = as.matrix(mel.fpkm), signatures, kcdf="Poisson", method="ssgsea")
saveRDS(mel.sig, file = "cache/mel.sig.rds")
```

```
Warning message in .local(expr, gset.idx.list, ...):
“34148 genes with constant expression values throuhgout the samples.”
```

```
Estimating ssGSEA scores for 7 gene sets.
  |                                                                      |   0%Using parallel with 4 cores
  |======================================================================| 100%
```

## Correlate functional signatures with phenotype¶

For the TCGA dataset this is not very informative since the signatures were created by testing whether the genes correlated in the TCGA cohort

In [9]:

```
mel.sig <- readRDS("cache/mel.sig.rds")

setPlotSize(5.5,7)

if (createPlots) {
    svg("plots/Supplementary_Figure_4B-TCGA_sig_corr.svg", width=7, height=5.5)
}
par(mfrow=c(2,3))
for (sig.index in 2:nrow(mel.sig)) {
    for (ref.sig in c(1)) {
        # get the correlation
        cor.res <- cor.test(mel.sig[ref.sig, ], mel.sig[sig.index,],
                           method = "spearman", alternative = "two.sided")

        if (round(cor.res$p.value, 2) < 0.01) {
            p.text <- " (p < 0.01)"
        } else {
            p.text <- paste0(" (p = ", round(cor.res$p.value, 3), ")")
        }

        plot(mel.sig[ref.sig, ], mel.sig[sig.index,],
            xlab = rownames(mel.sig)[ref.sig],
            ylab = rownames(mel.sig)[sig.index],
            main = paste0("cor = ", round(cor.res$estimate, 2), 
                          p.text))
    }
}
dev.off()

resetPlotSize()
```

**null device:** 1

Compare signatures against the expression of **CD8A**:

In [10]:

```
# sanity check
par(mfrow=c(1,1))
ref.expr <- log2(1 + cpm(mel.dge)["CD8A",])

setPlotSize(4,4)
if (createPlots) {
    svg("plots/Supplementary_Figure_4C-TCGA_sig_cd8a.svg", width=4, height=4)
}
for (sig.index in 1) {
    # get the correlation
    cor.res <- cor.test(ref.expr, mel.sig[sig.index,],
                       method = "pearson", alternative = "two.sided")
    
    if (round(cor.res$p.value, 2) < 0.01) {
        p.text <- " (p < 0.01)"
    } else {
        p.text <- paste0(" (p = ", round(cor.res$p.value, 3), ")")
    }
    
    plot(ref.expr, mel.sig[sig.index,],
        xlab = "CD8A (log2(1 + CPM))",
        ylab = rownames(mel.sig)[sig.index],
        main = paste0("cor = ", round(cor.res$estimate, 2), 
                      p.text))
}
dev.off()

resetPlotSize()
```

**null device:** 1

# Correlate published signatures¶

In [9]:

```
library(GSVA)

published.sig <- list(
    # TIS: 10.1186/s40425-018-0367-1
    "TIS" = c("PSMB10", "HLA-DQA1", "HLA-DRB1", "CMKLR1", "HLA-E", "NKG7", "CD8A", "CCL5", "CXCL9", "CD27", "CXCR6", "IDO1", "STAT1", "TIGIT", "LAG3", "CD274", "PDCD1LG2", "CD276"),
    # Taken from doi.org/10.1172/JCI91190
    "IFN-Gamma" = c("IFNG", "STAT1", "CCR5", "CXCL9", "CXCL10", "CXCL11", "IDO1", "PRF1", "GZMA", "MHCII", "HLA-DRA"),
    # T-cell exhaustion: Immunity 2007; 27: 670–684, doi: 10.3389/fimmu.2018.01786 
    "T-cell_Exhaustion" = c("HAVCR2", "CD38", "CD101", "CD244", "TIGIT"),
    # 10.1182/bloodadvances.2016000786.
    "T-Effector" = c("GZMA", "GZMB", "PRF1", "IFNG", "EOMES", "CD8A")
)

mel.pubslished.sig.expr <- gsva(expr = as.matrix(mel.fpkm), published.sig, kcdf="Poisson", method="ssgsea")
saveRDS(mel.pubslished.sig.expr, file = "cache/mel.pub.sig.rds")
```

```
Warning message in .local(expr, gset.idx.list, ...):
“34148 genes with constant expression values throuhgout the samples.”
```

```
Estimating ssGSEA scores for 4 gene sets.
  |                                                                      |   0%Using parallel with 4 cores
  |======================================================================| 100%
```

In [11]:

```
mel.pubslished.sig.expr <- readRDS("cache/mel.pub.sig.rds")

setPlotSize(3,10)
if (createPlots) {
    svg("plots/Supplementary_Figure_4E-TCGA_pub_sig.svg", width=10, height=3)
}
par(mfrow=c(1,4))
for (sig.index in 1:nrow(mel.pubslished.sig.expr)) {
    # get the correlation
    cor.res <- cor.test(mel.sig["TIPB", ], mel.pubslished.sig.expr[sig.index,],
                       method = "pearson", alternative = "two.sided")
    
    if (round(cor.res$p.value, 2) < 0.01) {
        p.text <- " (p < 0.01)"
    } else {
        p.text <- paste0(" (p = ", round(cor.res$p.value, 3), ")")
    }
    
    plot(mel.sig["TIPB", ], mel.pubslished.sig.expr[sig.index,],
        xlab = "TIPB",
        ylab = rownames(mel.pubslished.sig.expr)[sig.index],
        main = paste0("cor = ", round(cor.res$estimate, 2), 
                      p.text))
}
dev.off()

resetPlotSize()
```

**null device:** 1

## Compare signatures against cell types¶

Estimate cell types using xCell. The pre-compiled xCell results were downloaded from "http://xcell.ucsf.edu/" on 03.10.2018

In [ ]:

```
library(xCell)

# run xCell
mel.xcell <- xCellAnalysis(mel.fpkm, rnaseq = T)
saveRDS(mel.xcell, file = "cache/mel.xcell.rds")
```

In [12]:

```
mel.xcell <- readRDS("cache/mel.xcell.rds")

# create a function to compare all signatures against a cell type
compareSigWithXcell <- function(xcell.type, lab.name) {
    for (sig.index in 3) {
        # get the correlation
        cor.res <- cor.test(mel.xcell[xcell.type,], mel.sig[sig.index,],
                           method = "spearman", alternative = "two.sided")
        
        if (round(cor.res$p.value, 2) < 0.01) {
            p.text <- " (p < 0.01)"
        } else {
            p.text <- paste0(" (p = ", round(cor.res$p.value, 3), ")")
        }

        plot(mel.xcell[xcell.type,], mel.sig[sig.index,],
            xlab = lab.name,
            ylab = rownames(mel.sig)[sig.index],
            main = paste0("cor = ", round(cor.res$estimate, 2), 
                          p.text))
    }
}
```

Compare against **CD8+ T-cells**:

In [13]:

```
# sanity check
setPlotSize(4,4)
if (createPlots) {
    svg("plots/Supplementary_Figure_4D-TCGA_sig_cd8_tcell.svg", width=4, height=4)
}
par(mfrow=c(1,1))
compareSigWithXcell(xcell.type = "CD8+ T-cells", lab.name = "CD8+ T-cells")

dev.off()
resetPlotSize()
```

```
Warning message in cor.test.default(mel.xcell[xcell.type, ], mel.sig[sig.index, :
“Cannot compute exact p-value with ties”
```

**null device:** 1

In [14]:

```
cell.types <- c("CD8+ T-cells", "Macrophages", "Monocytes")

setPlotSize(height = 4, width = 12)
par(mfrow=c(1,3))
for (cell.type in cell.types) {
    compareSigWithXcell(xcell.type = cell.type, lab.name = cell.type)
}
resetPlotSize()
```

```
Warning message in cor.test.default(mel.xcell[xcell.type, ], mel.sig[sig.index, :
“Cannot compute exact p-value with ties”Warning message in cor.test.default(mel.xcell[xcell.type, ], mel.sig[sig.index, :
“Cannot compute exact p-value with ties”Warning message in cor.test.default(mel.xcell[xcell.type, ], mel.sig[sig.index, :
“Cannot compute exact p-value with ties”
```

## Compare signatures against survival¶

In [15]:

```
library(survival)
mel.surv <- Surv(mel.dge$samples$days_to_last_follow_up / 7, mel.dge$samples$vital_status == "dead")

setPlotSize(7,7)

cols <- brewer.pal(8, "Set1")

par(mfrow=c(1,1))
if (createPlots) {
    svg("plots/Figure_4A.svg", width = 6, height = 6)
}
for (sig.name in rownames(mel.sig)[3]) {
    # group the samples into high and low
    sample.groups <- factor(mel.sig[sig.name,] > median(mel.sig[sig.name,]),
                           levels = c(F,T),
                           labels = paste0(sig.name, c(" low", " high")))
    
    stat.res <- summary(coxph(mel.surv ~ sample.groups))
    p.val <- stat.res[["logtest"]][["pvalue"]]
    
    plot(survfit(mel.surv ~ sample.groups), col = cols[c(2,5)], xlab = "Overall survival (weeks)",
        ylab = "Rel. patients alive")
    
    if (round(p.val, 2) < 0.01) {
        p.text <- "likelihood ratio test p < 0.01"
    } else {
        p.text <- paste0("likelihood ratio test p = ", round(p.val, 2))
    }
    legend("topright", legend = c(levels(sample.groups), p.text), lty = 1,
                                  col = c(cols[c(2,5)], "white"))
}
dev.off()
```

**null device:** 1

# Riaz et al.¶

GSE91061: Riaz N, Havel JJ, Makarov V, Desrichard A et al. Tumor and Microenvironment Evolution during Immunotherapy with Nivolumab. Cell 2017 Nov 2;171(4):934-949.e16. PMID: 29033130

- 58 On-treatment and 51 Pre-treatment
- FPKM and RAW available (pre-processed against hg19)

The files were downloaded directly from GEO

## Load data¶

In [16]:

```
# FPKM
riaz.fpkm <- read.csv("data/GSE91061_BMS038109Sample.hg19KnownGene.fpkm.csv.gz", row.names = 1)

# RAW Counts
riaz.raw <- read.csv("data/GSE91061_BMS038109Sample.hg19KnownGene.raw.csv.gz", row.names = 1)

# Clinical
riaz.clinical <- read.csv("data/GSE91061_Clinical.t.csv", sep = "\t")
riaz.clinical$Time.to.Death..weeks. <- as.numeric(gsub(",", ".", riaz.clinical$Time.to.Death..weeks.))

# Add patient data to prepare for easy merging
patient.data <- data.frame(
    sample.id = colnames(riaz.fpkm),
    Patient = gsub("_.*", "", colnames(riaz.fpkm)),
    timepoint = gsub("[^_]*_([^_]*)_.*", "\\1", colnames(riaz.fpkm))
)

riaz.clinical <- merge(riaz.clinical, patient.data, by.x = "Patient", by.y = "Patient", all.y = T)
rownames(riaz.clinical) <- riaz.clinical$sample.id

# ensure everything is sorted correctly
if (!all(colnames(riaz.fpkm) %in% rownames(riaz.clinical))) {
    stop("Missing clinical information for riaz.fpkm")
}
riaz.fpkm <- riaz.fpkm[, rownames(riaz.clinical)]

if (!all(colnames(riaz.raw) %in% rownames(riaz.clinical))) {
    stop("Missing clinical annotation for riaz.raw")
}
riaz.raw <- riaz.raw[, rownames(riaz.clinical)]
```

Change to gene name as identifier:

In [17]:

```
# function to change to Gene name
toGeneName <- function(data) {
    invisible(library(org.Hs.eg.db))
    
    data$Gene <- mapIds(org.Hs.eg.db,
                       keys = rownames(data),
                       column = "SYMBOL",
                       keytype = "ENTREZID",
                       multiVals = "first")

    data <- data[order(rowSums(data[1:(ncol(data) - 1)]), decreasing = T), ]
    data <- data[!duplicated(data$Gene) & !is.na(data$Gene), ]

    rownames(data) <- data$Gene
    data$Gene <- NULL
    
    return(data)
}

# change to gene name
riaz.fpkm.gene <- toGeneName(riaz.fpkm)
riaz.raw.gene <- toGeneName(riaz.raw)
```

```
Loading required package: AnnotationDbi
Loading required package: stats4
Loading required package: BiocGenerics
Loading required package: parallel

Attaching package: ‘BiocGenerics’

The following objects are masked from ‘package:parallel’:

    clusterApply, clusterApplyLB, clusterCall, clusterEvalQ,
    clusterExport, clusterMap, parApply, parCapply, parLapply,
    parLapplyLB, parRapply, parSapply, parSapplyLB

The following object is masked from ‘package:limma’:

    plotMA

The following objects are masked from ‘package:stats’:

    IQR, mad, sd, var, xtabs

The following objects are masked from ‘package:base’:

    anyDuplicated, append, as.data.frame, basename, cbind, colMeans,
    colnames, colSums, dirname, do.call, duplicated, eval, evalq,
    Filter, Find, get, grep, grepl, intersect, is.unsorted, lapply,
    lengths, Map, mapply, match, mget, order, paste, pmax, pmax.int,
    pmin, pmin.int, Position, rank, rbind, Reduce, rowMeans, rownames,
    rowSums, sapply, setdiff, sort, table, tapply, union, unique,
    unsplit, which, which.max, which.min

Loading required package: Biobase
Welcome to Bioconductor

    Vignettes contain introductory material; view with
    'browseVignettes()'. To cite Bioconductor, see
    'citation("Biobase")', and for packages 'citation("pkgname")'.

Loading required package: IRanges
Loading required package: S4Vectors

Attaching package: ‘S4Vectors’

The following object is masked from ‘package:xts’:

    first

The following object is masked from ‘package:base’:

    expand.grid


'select()' returned 1:1 mapping between keys and columns
'select()' returned 1:1 mapping between keys and columns
```

## Quantify signature expression¶

In [18]:

```
library(GSVA)

riaz.sig <- gsva(expr = as.matrix(log2(1 + riaz.fpkm.gene)), signatures, kcdf="Gaussian", method="ssgsea")
saveRDS(riaz.sig, file = "cache/riaz.sig.rds")
```

```
Warning message in .local(expr, gset.idx.list, ...):
“19 genes with constant expression values throuhgout the samples.”
```

```
Estimating ssGSEA scores for 7 gene sets.
  |                                                                      |   0%Using parallel with 4 cores
  |======================================================================| 100%
```

## Correlate signatures with phenotype¶

In [19]:

```
riaz.sig <- readRDS("cache/riaz.sig.rds")

setPlotSize(5.5,7)
if (createPlots) {
    svg("plots/Supplementary_Figure_5A-riaz_sig_corr.svg", width=7, height=5.5)
}
par(mfrow=c(2,3))
for (sig.index in 2:nrow(riaz.sig)) {
    # get the correlation
    cor.res <- cor.test(riaz.sig["TIPB", ], riaz.sig[sig.index,],
                       method = "spearman")
    
    if (round(cor.res$p.value, 2) < 0.01) {
        p.text <- " (p < 0.01)"
    } else {
        p.text <- paste0(" (p < ", round(cor.res$p.value, 3), ")")
    }
    
    plot(riaz.sig["TIPB", ], riaz.sig[sig.index,],
        xlab = "TIPB",
        ylab = rownames(riaz.sig)[sig.index],
        main = paste0("cor = ", round(cor.res$estimate, 2), 
                      p.text))
}
dev.off()
resetPlotSize()
```

**null device:** 1

Correlate against expression of CD8A:

In [20]:

```
riaz.sig <- readRDS("cache/riaz.sig.rds")

setPlotSize(4,4)
if (createPlots) {
    svg("plots/Supplementary_Figure_5B-riaz_cd8a.svg", width=4, height=4)
}
par(mfrow=c(1,1))
for (sig.index in 3) {
    # get the correlation
    cor.res <- cor.test(as.numeric(log2(riaz.fpkm.gene["CD8A",])), riaz.sig[sig.index,],
                       method = "spearman")
    
    if (round(cor.res$p.value, 3) < 0.01) {
        p.lab <- " (p < 0.01)"
    } else {
       p.lab <- paste0(" (p = ", round(cor.res$p.value, 2), ")")
    }
    
    plot(as.numeric(log2(riaz.fpkm.gene["CD8A",])), riaz.sig[sig.index,],
        xlab = "CD8A (log2 FPKM)",
        ylab = rownames(riaz.sig)[sig.index],
        main = paste0("cor = ", round(cor.res$estimate, 2), p.lab))
}
dev.off()
resetPlotSize()
```

**null device:** 1

## Signatures vs. treatment¶

### Treatment timepoint¶

In [25]:

```
library(ggplot2)
library(reshape2)

riaz.sig <- readRDS("cache/riaz.sig.rds")

plot.data <- melt(riaz.sig, varnames = c("signature", "sample.id"))
plot.data <- merge(plot.data, riaz.clinical, by.x = "sample.id", by.y = "sample.id")

plot.data$timepoint <- factor(plot.data$timepoint, levels = c("Pre", "On"), labels = c("Pre anti-PD1", "On anti-PD1"))

setPlotSize(4,7)

plot.obj <- ggplot(plot.data, aes(x = signature, fill = timepoint, y = value)) +
    geom_boxplot() +
    my_theme_bw +
    labs(x = NULL, y = "Signature expression", fill = "Timepoint") +
    scale_fill_brewer(palette = "Set2") +
    theme(axis.text.x = element_text(angle = 45, hjust = 1))

print(plot.obj)

svg("plots/Figure_4C_sig_expr_pd1.svg", width = 6, height = 3)
print(plot.obj)
dev.off()

p.vals <- do.call("rbind", by(plot.data, plot.data$signature, function(x) {
    pre <- x[x[,"timepoint"] == "Pre anti-PD1", "value"]
    on <- x[x[,"timepoint"] == "On anti-PD1", "value"]
    t.res <- t.test(pre, on, paired = F, alternative = "less")
    
    data.frame(
        p = t.res$p.value,
        df = t.res$parameter["df"],
        t = t.res$statistic["t"]
    )
}))

p.vals$adj.p <- p.adjust(p.vals$p, "BH")
p.vals <- round(p.vals, 2)

p.vals
```

**png:** 2

|  | p | df | t | adj.p |
| --- | --- | --- | --- | --- |
| TIPB | 0.02 | 107.00 | -2.03 | 0.03 |
| Activation | 0.02 | 105.02 | -2.05 | 0.03 |
| Co-Stimulation | 0.01 | 106.66 | -2.29 | 0.03 |
| Immune-checkpoint | 0.00 | 106.56 | -2.77 | 0.02 |
| Exhaustion | 0.09 | 106.70 | -1.35 | 0.09 |
| Pro-Inflammation | 0.01 | 100.14 | -2.27 | 0.03 |
| Cytokines-Immunsupp | 0.06 | 96.33 | -1.58 | 0.07 |

### Treatment response¶

In [26]:

```
plot.data$Response <- factor(plot.data$Response, levels = c("PD", "SD", "PR", "CR", "NE"))

setPlotSize(10, 10)

plot.obj <- ggplot(plot.data[!is.na(plot.data$Response),], aes(x = Response, y = value, fill = timepoint)) +
    geom_boxplot() +
    facet_wrap(~ signature, ncol = 1, scales = "free_y") +
    scale_fill_brewer(palette = "Set2") +
    my_theme_bw +
    labs(y = "Signature expression (ssGSEA)", fill = "Timepoint", x = "Clinical response")

print(plot.obj)

svg("plots/Supplementary_Figure_6-riaz_sig_clin_out.svg", width = 10, height = 10)
print(plot.obj)
dev.off()
```

**png:** 2

## Compare signatures against survival¶

In [27]:

```
library(survival)

riaz.surv <- Surv(riaz.clinical$Time.to.Death..weeks., riaz.clinical$Dead.Alive..Dead...True. == 1)

setPlotSize(7,7)

cols <- brewer.pal(8, "Set1")

par(mfrow=c(1,1))

plotSurvival <- function(sample.groups, timepoint) {
    samples.to.use <- riaz.clinical$timepoint == timepoint &
                        grepl("25%", sample.groups)
    
    sample.groups <- sample.groups[samples.to.use]
    sample.groups <- droplevels(sample.groups)
    
    stat.res <- summary(coxph(riaz.surv[samples.to.use] ~ sample.groups))
    p.val <- stat.res[["logtest"]][["pvalue"]]
    p.text <- paste0("likelihood ratio test p = ", round(p.val, 2))
    
    plot(survfit(riaz.surv[samples.to.use] ~ sample.groups), lty = 1, 
         col = cols[c(2,5)],
        ylab = "Rel. patients alive",
        xlab = "Overall survival (weeks)")
    legend("topright", legend = c(levels(sample.groups), p.text),
          lty = 1,
          col = c(cols[c(2,5)], "white"))
}

par(mfrow=c(1,1))
if (createPlots) {
    svg("plots/Figure_4B_surv_riaz.svg", width = 6, height = 6)
}
for (sig.name in rownames(riaz.sig)[3]) {
    # create four groups of samples
    sample.groups <- factor(riaz.sig[sig.name,] > median(riaz.sig[sig.name,]),
                         levels = c(F, T), labels = c("low", "high"))
    
    sig.expr <- riaz.sig[sig.name,]
    sample.groups <- cut(sig.expr,
                        breaks = c(-Inf, quantile(sig.expr, 0.25), median(sig.expr), quantile(sig.expr, 0.75), Inf),
                        labels = c("TIPB lowest 25%", "med-low", "med-high", "TIPB highest 25%"))
    # split based on pre and on samples
    plotSurvival(sample.groups, "Pre") 
    #plotSurvival(sample.groups, "On") 
}
dev.off()
```

**null device:** 1

## Compare against estimated cell types¶

In [51]:

```
library(xCell)

# run xCell
riaz.xcell <- xCellAnalysis(riaz.fpkm.gene, rnaseq = T)
saveRDS(riaz.xcell, file = "cache/riaz.xcell.rds")
```

```
[1] "Num. of genes: 10650"
Estimating ssGSEA scores for 489 gene sets.
  |                                                                      |   0%Using parallel with 4 cores
  |======================================================================| 100%
```

In [29]:

```
riaz.xcell <- readRDS("cache/riaz.xcell.rds")

setPlotSize(4,4)
par(mfrow=c(1,1))

ref.expr <- as.numeric( riaz.xcell["CD8+ T-cells", ] )

if (createPlots) {
    svg("plots/Supplementary_Figure_5C-riaz_cd8_T_cells.svg", width=4, height = 4)
}
for (sig.index in 1) {
    # get the correlation
    cor.res <- cor.test(ref.expr, riaz.sig[sig.index,],
                       method = "spearman")
    
    if (round(cor.res$p.value, 3) < 0.01) {
        p.lab <- " (p < 0.01)"
    } else {
       p.lab <- paste0(" (p = ", round(cor.res$p.value, 2), ")")
    }
    
    plot(ref.expr, riaz.sig[sig.index,],
        xlab = "CD8+ T-cells",
        ylab = rownames(mel.sig)[sig.index],
        main = paste0("cor = ", round(cor.res$estimate, 2), 
                      p.lab))
}
dev.off()

resetPlotSize()
```

```
Warning message in cor.test.default(ref.expr, riaz.sig[sig.index, ], method = "spearman"):
“Cannot compute exact p-value with ties”
```

**null device:** 1

In [30]:

```
setPlotSize(4,4)
par(mfrow=c(1,1))

ref.expr <- as.numeric( riaz.xcell["Macrophages", ] )

for (sig.index in 1) {
    # get the correlation
    cor.res <- cor.test(ref.expr, riaz.sig[sig.index,],
                       method = "spearman")
    
    if (round(cor.res$p.value, 3) < 0.01) {
        p.lab <- " (p < 0.01)"
    } else {
       p.lab <- paste0(" (p = ", round(cor.res$p.value, 2), ")")
    }
    
    plot(ref.expr, riaz.sig[sig.index,],
        xlab = "Macrophages",
        ylab = rownames(mel.sig)[sig.index],
        main = paste0("cor = ", round(cor.res$estimate, 2), 
                      p.lab))
}

resetPlotSize()
```

```
Warning message in cor.test.default(ref.expr, riaz.sig[sig.index, ], method = "spearman"):
“Cannot compute exact p-value with ties”
```

### Compare against single gene expression¶

In [31]:

```
ref.expr <- as.numeric( log2(1 + riaz.fpkm.gene["CD8A",]) )

for (sig.index in 1) {
    # get the correlation
    cor.res <- cor.test(ref.expr, riaz.sig[sig.index,],
                       method = "pearson", alternative = "two.sided")
    
    plot(ref.expr, riaz.sig[sig.index,],
        xlab = "CD8A (1 + log2(FPKM))",
        ylab = rownames(mel.sig)[sig.index],
        main = paste0("cor = ", round(cor.res$estimate, 2), 
                      " (p = ", round(cor.res$p.value, 3), ")"))
}
```

# Package versions¶

In [32]:

```
sessionInfo()
```

```
R version 3.5.1 (2018-07-02)
Platform: x86_64-pc-linux-gnu (64-bit)
Running under: Ubuntu 18.04.1 LTS

Matrix products: default
BLAS: /usr/lib/x86_64-linux-gnu/blas/libblas.so.3.7.1
LAPACK: /usr/lib/x86_64-linux-gnu/lapack/liblapack.so.3.7.1

locale:
 [1] LC_CTYPE=en_US.UTF-8       LC_NUMERIC=C              
 [3] LC_TIME=de_AT.UTF-8        LC_COLLATE=en_US.UTF-8    
 [5] LC_MONETARY=de_AT.UTF-8    LC_MESSAGES=en_US.UTF-8   
 [7] LC_PAPER=de_AT.UTF-8       LC_NAME=C                 
 [9] LC_ADDRESS=C               LC_TELEPHONE=C            
[11] LC_MEASUREMENT=de_AT.UTF-8 LC_IDENTIFICATION=C       

attached base packages:
[1] parallel  stats4    stats     graphics  grDevices utils     datasets 
[8] methods   base     

other attached packages:
 [1] org.Hs.eg.db_3.6.0         AnnotationDbi_1.42.1      
 [3] IRanges_2.14.12            S4Vectors_0.18.3          
 [5] Biobase_2.40.0             BiocGenerics_0.26.0       
 [7] survival_2.42-6            PerformanceAnalytics_1.5.2
 [9] xts_0.11-2                 zoo_1.8-4                 
[11] GSVA_1.28.0                edgeR_3.22.5              
[13] limma_3.36.5               reshape2_1.4.3            
[15] RColorBrewer_1.1-2         ggplot2_3.1.0             

loaded via a namespace (and not attached):
 [1] bit64_0.9-7          jsonlite_1.5         splines_3.5.1       
 [4] shiny_1.1.0          assertthat_0.2.0     getPass_0.2-2       
 [7] blob_1.1.1           pillar_1.3.0         RSQLite_2.1.1       
[10] lattice_0.20-35      glue_1.3.0           quadprog_1.5-5      
[13] uuid_0.1-2           digest_0.6.18        promises_1.0.1      
[16] colorspace_1.3-2     Matrix_1.2-14        htmltools_0.3.6     
[19] httpuv_1.4.5         plyr_1.8.4           GSEABase_1.42.0     
[22] XML_3.98-1.16        pkgconfig_2.0.2      purrr_0.2.5         
[25] xtable_1.8-3         scales_1.0.0         later_0.7.5         
[28] tibble_1.4.2         annotate_1.58.0      withr_2.1.2         
[31] repr_0.15.0          lazyeval_0.2.1       magrittr_1.5        
[34] crayon_1.3.4         mime_0.6             memoise_1.1.0       
[37] evaluate_0.12        graph_1.58.2         tools_3.5.1         
[40] stringr_1.3.1        munsell_0.5.0        locfit_1.5-9.1      
[43] bindrcpp_0.2.2       compiler_3.5.1       rlang_0.3.0.1       
[46] grid_3.5.1           RCurl_1.95-4.11      pbdZMQ_0.3-3        
[49] IRkernel_0.8.12.9000 labeling_0.3         bitops_1.0-6        
[52] base64enc_0.1-3      gtable_0.2.0         DBI_1.0.0           
[55] R6_2.3.0             dplyr_0.7.7          bit_1.1-14          
[58] bindr_0.1.1          shinythemes_1.1.1    stringi_1.2.4       
[61] IRdisplay_0.6        Rcpp_0.12.19         geneplotter_1.58.0  
[64] tidyselect_0.2.5
```
