# Supplementary material for: B cells sustain inflammation and predict response to immune checkpoint blockade in human melanoma
Source: Nat Commun. 2019 Sep 13;10:4186. doi: 10.1038/s41467-019-12160-2 (PMC6744450; doi:10.1038/s41467-019-12160-2)
Supplement: Supplementary file 8 — Supplementary Software 1 [file 41467_2019_12160_MOESM8_ESM.zip › 193518_1_other_0_psz9cz.zip!/TAB Induction by MCM.html]

TAB Induction by MCM


# Introduction¶

This notebook contains the analysis of the RNASeq, Proteomics, and Phospho-Proteomics data from experiments where tumor infiltrating/associated B-cells (TIBC/TAB) and peripheral B-cells (PBMC) from 4 patients were exposed to melanoma cell conditioned (MCM) or control (MOCK) medium.

In [1]:

```
# create plot style and save general options
library(ggplot2)
library(reshape2)
my_theme_bw <- theme_bw() +
    theme(strip.background = element_blank(),
          panel.border = element_rect(colour = "black"))

my_theme <- function(base_size = 12, base_family = "", flip_axis = F) {
    flip_axis_theme <- theme(axis.text.x = element_text(angle = 45, hjust = 1))
    
    res <- theme_bw(base_size=base_size, base_family=base_family) %+replace% 
    theme(strip.background = element_blank(),
          panel.grid.major = element_blank(),
          panel.grid.minor = element_blank(),
          panel.border = element_blank(), 
          axis.line = element_line(colour = "#333333")
    )
    
    if (flip_axis) {
        res <- res + flip_axis_theme
    }
    
    return(res)
}

org.options <- options()

# add some convenience functions
setPlotSize <- function(height, width) {
    options(repr.plot.height=height, repr.plot.width = width)
}

resetPlotSize <- function() {
    options(org.options)
}

# if set to true, SVG plots are created in the "plots" subdirectory
createPlots <- F

if (!file.exists("plots")) {
    message("Created plots directory")
    dir.create("plots")
}

if (!file.exists("cache")) {
    message("Created cache directory")
    dir.create("cache")
}
```

```
Registered S3 methods overwritten by 'ggplot2':
  method         from 
  [.quosures     rlang
  c.quosures     rlang
  print.quosures rlang
```

# Load data¶

## Load RNASeq data¶

Load the raw read counts (as estimated by STAR) from the rds file and add the metadata:

In [2]:

```
# ---- load the read counts of the first batch -------------------------------
rnaseq.raw <- readRDS("data/mcm_induction_rnaseq_star.rds")

# Load secon batch data (sequenced by a different facility)
rnaseq.rev.raw <- readRDS("data/mcm_induction_rnaseq_star_second.rds")
# remove the first sample - it's a blank
rnaseq.rev.raw <- rnaseq.rev.raw[, 2:ncol(rnaseq.rev.raw)]

# ---- load the sample data --------------------------------
# org data
id_mapping <- read.table("data/mcm_induction_rnaseq_mapping.csv", header = T, sep = "\t", stringsAsFactors = F)
id_mapping$Id <- gsub("/", "-", id_mapping$Id)
id_mapping$patient <- gsub(" .*", "", id_mapping$Sample)
id_mapping$cell_type <- gsub("[^ ]* ([^ ]*).*", "\\1", id_mapping$Sample)
id_mapping$treatment <- gsub(".* ", "", id_mapping$Sample)

# indicate that these were all incubated with MCM from P1
id_mapping$patient <- paste0(id_mapping$patient, "-M1")

rownames(id_mapping) <- id_mapping$Id
id_mapping <- id_mapping[, !colnames(id_mapping) %in% c("Sample", "Id")]

# second data
# these B cells were incubated with autologous MCM
patient_id <- gsub(".*_pt_([123])_.*", "P\\1-M\\1", colnames(rnaseq.rev.raw))
cell_type <- ifelse(grepl("_PBMC_", colnames(rnaseq.rev.raw)), "PBMCB", "TAB")
treatment <- ifelse(grepl("_MCM_", colnames(rnaseq.rev.raw)), "MCM", "MOCK")

# fix the patient ids to match the previous experiment
patient_id_mapping <- c("1" = "5", "2" = "4")
for (exp2_id in names(patient_id_mapping)) {
    patient_id <- gsub(exp2_id, patient_id_mapping[exp2_id], patient_id)
}

rev.sample.data <- data.frame(
    patient = patient_id,
    cell_type = cell_type,
    treatment = treatment,
    row.names = paste0(patient_id, "_", cell_type, "_", treatment)
)

# ---- merge the read counts -------------------------------------------
all_genes <- unique(c(rownames(rnaseq.raw), rownames(rnaseq.rev.raw)))

missing_genes_org <- all_genes[!all_genes %in% rownames(rnaseq.raw)]
rnaseq.raw[missing_genes_org, ] <- 0

missing_genes_rev <- all_genes[!all_genes %in% rownames(rnaseq.rev.raw)]
rnaseq.rev.raw[missing_genes_rev, ] <- 0

merged_reads <- cbind(rnaseq.raw[all_genes, ], rnaseq.rev.raw[all_genes, ])

write.table(merged_reads, file = "plots/mcm_induction-read_counts.tsv", col.names = NA, row.names = T, quote = F, sep = "\t")
```

In [3]:

```
# ---- add gene mappings ----------------------------------------------
library(org.Hs.eg.db)

annotations <- data.frame(row.names = rownames(merged_reads), stringsAsFactors = F)
annotations$gene <- mapIds(org.Hs.eg.db,
                           keys = rownames(merged_reads),
                           column = "SYMBOL",
                           keytype = "ENSEMBL",
                           multiVals = "first")
annotations$uniprot <- mapIds(org.Hs.eg.db,
                              keys = rownames(merged_reads),
                              column = "UNIPROT",
                              keytype = "ENSEMBL",
                              multiVals = "first")
annotations$name <- mapIds(org.Hs.eg.db,
                           keys = rownames(merged_reads),
                           column = "GENENAME",
                           keytype = "ENSEMBL",
                           multiVals = "first")
annotations$entrez <- mapIds(org.Hs.eg.db,
                           keys = rownames(merged_reads),
                           column = "ENTREZID",
                           keytype = "ENSEMBL",
                           multiVals = "first")

# ---- create the EList object --------------------------------------------
library(edgeR)
rnaseq.dge <- DGEList(counts = merged_reads, 
                      samples = rbind(id_mapping[colnames(rnaseq.raw), ], rev.sample.data), 
                      genes = annotations[rownames(merged_reads), ], 
                      group = c(id_mapping[colnames(rnaseq.raw), "treatment"], rev.sample.data$treatment))

# change to "nicer" sample names
rnaseq.dge$samples$org_samples <- colnames(rnaseq.dge)
colnames(rnaseq.dge) <- paste0(rnaseq.dge$samples$patient, "_", rnaseq.dge$samples$cell_type, "_", rnaseq.dge$samples$treatment)

# use MOCK as reference
rnaseq.dge$samples$treatment <- relevel(factor(rnaseq.dge$samples$treatment), "MOCK")
```

```
Loading required package: AnnotationDbi
Loading required package: stats4
Loading required package: BiocGenerics
Loading required package: parallel

Attaching package: ‘BiocGenerics’

The following objects are masked from ‘package:parallel’:

    clusterApply, clusterApplyLB, clusterCall, clusterEvalQ,
    clusterExport, clusterMap, parApply, parCapply, parLapply,
    parLapplyLB, parRapply, parSapply, parSapplyLB

The following objects are masked from ‘package:stats’:

    IQR, mad, sd, var, xtabs

The following objects are masked from ‘package:base’:

    anyDuplicated, append, as.data.frame, basename, cbind, colMeans,
    colnames, colSums, dirname, do.call, duplicated, eval, evalq,
    Filter, Find, get, grep, grepl, intersect, is.unsorted, lapply,
    lengths, Map, mapply, match, mget, order, paste, pmax, pmax.int,
    pmin, pmin.int, Position, rank, rbind, Reduce, rowMeans, rownames,
    rowSums, sapply, setdiff, sort, table, tapply, union, unique,
    unsplit, which, which.max, which.min

Loading required package: Biobase
Welcome to Bioconductor

    Vignettes contain introductory material; view with
    'browseVignettes()'. To cite Bioconductor, see
    'citation("Biobase")', and for packages 'citation("pkgname")'.

Loading required package: IRanges
Loading required package: S4Vectors

Attaching package: ‘S4Vectors’

The following object is masked from ‘package:base’:

    expand.grid


'select()' returned 1:many mapping between keys and columns
'select()' returned 1:many mapping between keys and columns
'select()' returned 1:many mapping between keys and columns
'select()' returned 1:many mapping between keys and columns
Loading required package: limma

Attaching package: ‘limma’

The following object is masked from ‘package:BiocGenerics’:

    plotMA
```

In [4]:

```
rnaseq.dge$samples
```

|  | group | lib.size | norm.factors | patient | cell\_type | treatment | org\_samples |
| --- | --- | --- | --- | --- | --- | --- | --- |
| P3-M1\_TAB\_MOCK | MOCK | 29907534 | 1 | P3-M1 | TAB | MOCK | 195-13 |
| P3-M1\_TAB\_MCM | MCM | 26397322 | 1 | P3-M1 | TAB | MCM | 195-14 |
| P4-M1\_PBMCB\_MOCK | MOCK | 18194834 | 1 | P4-M1 | PBMCB | MOCK | 195-19 |
| P4-M1\_PBMCB\_MCM | MCM | 24282215 | 1 | P4-M1 | PBMCB | MCM | 195-20 |
| P1-M1\_TAB\_native | native | 40280314 | 1 | P1-M1 | TAB | native | 196-2 |
| P3-M1\_PBMCB\_MOCK | MOCK | 22628117 | 1 | P3-M1 | PBMCB | MOCK | 197-11 |
| P3-M1\_PBMCB\_MCM | MCM | 23319849 | 1 | P3-M1 | PBMCB | MCM | 197-12 |
| P4-M1\_TAB\_MOCK | MOCK | 22266757 | 1 | P4-M1 | TAB | MOCK | 197-13 |
| P4-M1\_TAB\_MCM | MCM | 27595533 | 1 | P4-M1 | TAB | MCM | 197-14 |
| P1-M1\_TAB\_MOCK | MOCK | 113200728 | 1 | P1-M1 | TAB | MOCK | 197-1 |
| P1-M1\_TAB\_MCM | MCM | 25830746 | 1 | P1-M1 | TAB | MCM | 197-2 |
| P1-M1\_PBMCB\_MOCK | MOCK | 15521345 | 1 | P1-M1 | PBMCB | MOCK | 197-3 |
| P1-M1\_PBMCB\_MCM | MCM | 31446259 | 1 | P1-M1 | PBMCB | MCM | 197-4 |
| P2-M1\_TAB\_MOCK | MOCK | 24470913 | 1 | P2-M1 | TAB | MOCK | 197-5 |
| P2-M1\_TAB\_MCM | MCM | 27055478 | 1 | P2-M1 | TAB | MCM | 197-6 |
| P2-M1\_PBMCB\_MOCK | MOCK | 23998487 | 1 | P2-M1 | PBMCB | MOCK | 197-7 |
| P2-M1\_PBMCB\_MCM | MCM | 25929798 | 1 | P2-M1 | PBMCB | MCM | 197-8 |
| P2-M1\_PBMCB\_native | native | 30789329 | 1 | P2-M1 | PBMCB | native | 198-2 |
| P3-M1\_PBMCB\_native | native | 31545937 | 1 | P3-M1 | PBMCB | native | 198-3 |
| P1-M1\_PBMCB\_native | native | 26803779 | 1 | P1-M1 | PBMCB | native | 198-4 |
| P5-M5\_PBMCB\_MCM | 1 | 65196998 | 1 | P5-M5 | PBMCB | MCM | BSF\_0595\_H52VWBBXY\_6\_pt\_1\_PBMC\_B\_MCM\_S51256 |
| P4-M4\_PBMCB\_MCM | 1 | 72590831 | 1 | P4-M4 | PBMCB | MCM | BSF\_0595\_H52VWBBXY\_6\_pt\_2\_PBMC\_B\_MCM\_S51254 |
| P4-M4\_PBMCB\_MOCK | 2 | 81669517 | 1 | P4-M4 | PBMCB | MOCK | BSF\_0595\_H52VWBBXY\_6\_pt\_2\_PBMC\_B\_mock\_S51255 |
| P3-M3\_PBMCB\_MCM | 1 | 81764931 | 1 | P3-M3 | PBMCB | MCM | BSF\_0595\_H52VWBBXY\_6\_pt\_3\_PBMC\_B\_MCM\_S51253 |
| P3-M3\_PBMCB\_MOCK | 2 | 73244673 | 1 | P3-M3 | PBMCB | MOCK | BSF\_0595\_H52VWBBXY\_6\_pt\_3\_PBMC\_B\_mock\_S51252 |
| P5-M5\_PBMCB\_MOCK | 2 | 99435521 | 1 | P5-M5 | PBMCB | MOCK | BSF\_0599\_H52WMBBXY\_8\_pt\_1\_PBMC\_B\_mock\_S51290 |

Display a PCA across all samples:

In [5]:

```
pca.fit <- prcomp(cpm(rnaseq.dge, log = T, prior.count = 2), center = T)
plot.data <- data.frame(pca.fit$rotation)

plot.data <- merge(plot.data, rnaseq.dge$samples, by.x = 0, by.y = 0)

setPlotSize(3,5)
ggplot(plot.data, aes(x = PC1, y = PC2, color = cell_type, shape = treatment)) +
    geom_point() +
    scale_color_brewer(palette = "Set1") +
    labs(color = "Cell type", shape = "Treatment")

resetPlotSize()
```

The PCA analysis clearly shows that the main difference is caused by the immortalization (native vs. rest). Additionally, there is no clear separation between PBMC and TIBC.

Remove all "native" samples, change to Gene identifier and filter based on transcript abundance:

In [6]:

```
# remove native samples
rnaseq.dge <- rnaseq.dge[, rnaseq.dge$samples$group != "native"]
rnaseq.dge$samples$group <- droplevels(rnaseq.dge$samples$group)
rnaseq.dge$samples$treatment <- droplevels(rnaseq.dge$samples$treatment)

# remove counts of unmapped reads
rnaseq.dge <- rnaseq.dge[grep("^N_", rownames(rnaseq.dge)) * -1, ]

# only keep highest counts for duplicated genes
message("Loaded ", nrow(rnaseq.dge), " genes")

sort_order <- order(rowSums(rnaseq.dge$counts), decreasing=TRUE)
rnaseq.dge <- rnaseq.dge[sort_order, ]
is_duplicated <- duplicated(rnaseq.dge$genes$gene)
rnaseq.dge <- rnaseq.dge[!is_duplicated & !is.na(rnaseq.dge$genes$gene), ]

rownames(rnaseq.dge) <- rnaseq.dge$genes$gene

message(nrow(rnaseq.dge), " genes after change to gene name")

# only keep highly expressed genes
total.expression <- rowSums(rnaseq.dge$counts)
rnaseq.dge <- rnaseq.dge[total.expression >= 100, , keep.lib.sizes = F]

message(nrow(rnaseq.dge), " with >= 100 transcripts")
```

```
Loaded 59073 genes
25907 genes after change to gene name
14705 with >= 100 transcripts
```

## Load proteomics data¶

The data is already normalized by channel (on the PSM level) and all spectra with >70% interference were removed.

In [7]:

```
library(MSnbase)
library(limma)
library(methods)
library(utils)
library(stats)

annotateProteins <- function(accessions) {
    library(org.Hs.eg.db)
    
    annotations <- data.frame(uniprot = accessions, stringsAsFactors = F)
    
    annotations$RefSeqId <- mapIds(org.Hs.eg.db,
                                     keys = accessions,
                                     column = "ENTREZID",
                                     keytype = "UNIPROT",
                                     multiVals = "first")
    
    annotations$Gene <- mapIds(org.Hs.eg.db,
                                     keys = accessions,
                                     column = "SYMBOL",
                                     keytype = "UNIPROT",
                                     multiVals = "first")
    
    annotations$Name <- mapIds(org.Hs.eg.db,
                                     keys = accessions,
                                     column = "GENENAME",
                                     keytype = "UNIPROT",
                                     multiVals = "first")
    
    return(annotations)
}

# Load the PSM-level quant data into an Msnbase msnset object
loadAsMsnSet <- function(filename) {
    # data is already normalised based on channel = sample and run
    # all spectra with >70% interference are removed
    message("Loading data...")
    merged.data <- read.table(filename, header = T, sep = "\t", 
                              stringsAsFactors = F, quote = "", 
                              comment.char = "")
    
    message(nrow(merged.data), " PSMs loaded.")
    
    # only use phospho ids if it's a phospho experiment
    if (length(grep("TiO2", filename)) == 1) {
        merged.data <- merged.data[grep("-79\\.", merged.data$modif), ]
        message(nrow(merged.data), " phospho PSMs loaded.")
    }
    
    # ---- load and clean the data ----
    # remove any ambiguous ids
    is.duplicate <- duplicated(merged.data$spectrum)
    merged.data <- merged.data[!merged.data$spectrum %in% merged.data$spectrum[is.duplicate], ]
    
    # extract the quant data
    quant.data <- merged.data[, grep("^X.*_ions", colnames(merged.data))]
    rownames(quant.data) <- merged.data$spectrum
    
    # extract the id data
    id.data <- merged.data[, grep("^X", colnames(merged.data)) * -1]
    rownames(id.data) <- id.data$spectrum
    id.data$spectrum <- NULL
    
    # sample metadata
    sample.data <- data.frame(
        row.names = c("X126", "X127N", "X127C", "X128N", "X128C", "X129N", "X129C", "X130N", "X130C", "X131N"),
        patient.id = rep(c("P1", "P2", "P3", "P4", "all"), each = 2),
        condition = rep(c("MOCK", "MCM"), times = 5)
    )
    
    if (length(grep("TMT2", filename)) == 1) {
        sample.data$cell.type <- "PBMCB"
    } else {
        sample.data$cell.type <- "TAB"
    }
    
    elist_raw <- new("EListRaw", list(E = quant.data))
    elist_raw$genes <- id.data[, c("accession", "peptide", "modif", "charge", "theo.mass", "exp.mass", "parent.intens", "retention.time", "search.engine", "score.msgf", "is.decoy", "file")]
    elist_raw$targets <- sample.data
    
    rm(merged.data, is.duplicate, quant.data, id.data, filename)
    
    # ---- Convert to MSnbase ----
    expr.data <- elist_raw$E
    colnames(expr.data) <- gsub("_ions", "", colnames(expr.data))
    expr.data <- as.matrix(expr.data)
    
    msnset <- new("MSnSet",
                  qual = data.frame(),
                  exprs = expr.data,
                  featureData = new("AnnotatedDataFrame", data = elist_raw$genes),
                  phenoData = new("AnnotatedDataFrame", data = elist_raw$targets),
                  annotation = "No annotation")
    
    # fix some colnames in the fData
    fvarLabels(msnset)[which(fvarLabels(msnset) == "modif")] <- "modifications"
    fvarLabels(msnset)[which(fvarLabels(msnset) == "exp.mass")] <- "mass_to_charge"
    fvarLabels(msnset)[which(fvarLabels(msnset) == "score.msgf")] <- "search_engine_score"
    fvarLabels(msnset)[which(fvarLabels(msnset) == "peptide")] <- "sequence"
    
    return(msnset)
}

# use iPQF to combine PSM-level quantification on the protein level
doIPQFQuant <- function(msnset) {
    # remove all spectra with missing values
    has.missing.values <- (rowSums(is.na(exprs(msnset))) > 0 | rowSums(exprs(msnset) == 0)) > 0    
    msnset <- filterNA(msnset,0.0)
    
    # use iPQF to get protein-level quantitation
    allProts <- combineFeatures(msnset, fun="iPQF",
                                groupBy=fData(msnset)$accession, 
                                verbose=F)
    
    # log2 transform
    exprs(allProts) <- log2(exprs(allProts))
    
    # add protein annotations
    annotations <- annotateProteins(rownames(allProts))
    rownames(annotations) <- annotations$uniprot
    fData(allProts) <- annotations[rownames(allProts), ]
    
    # add sample annotations
    pData(allProts) <- pData(msnset)[colnames(allProts), ]
    pData(allProts)$condition <- factor(pData(allProts)$condition, levels = c("MOCK", "MCM"), labels = c("MOCK", "MCM"))
    pData(allProts)$patient.id <- gsub("([A-Z])([A-Z])", "\\1-\\2", pData(allProts)$patient.id)
    
    pData(allProts)$sample_name <- paste(pData(allProts)$patient.id, 
                                         pData(allProts)$condition, 
                                         pData(allProts)$cell.type)
    
    return(allProts)
}

# ---- Load TMT1 and TMT2 data ----
filenames <- c("data/TMT2_Global.quant.tsv.gz", "data/TMT1_Global.quant.tsv.gz")
msnset.pbmcb <- loadAsMsnSet(filenames[1])
msnset.tibc <- loadAsMsnSet(filenames[2])

prots.pbmcb <- doIPQFQuant(msnset.pbmcb)
prots.tibc <- doIPQFQuant(msnset.tibc)

sampleNames(prots.pbmcb) <- pData(prots.pbmcb)$sample_name
sampleNames(prots.tibc) <- pData(prots.tibc)$sample_name

# merge protein quants
merged.exp <- merge(exprs(prots.pbmcb), exprs(prots.tibc), 
                    all.x = T, all.y = T, 
                    by.x = 0, by.y = 0)
rownames(merged.exp) <- merged.exp$Row.names
merged.exp$Row.names <- NULL

sample.data <- data.frame(
    row.names = colnames(merged.exp),
    patient.id = gsub(" .*", "", colnames(merged.exp)),
    condition = gsub("[^ ]* ([^ ]*) .*", "\\1", colnames(merged.exp)),
    cell.type = gsub(".* ", "", colnames(merged.exp))
)

# indicate that these were all incubated with MCM from patient 1
sample.data$patient.id <- paste0(sample.data$patient.id, "-M1")

# remove all missing values
merged.exp.complete <- merged.exp[rowSums(is.na(merged.exp)) == 0, ]

# create a limma object again
protein.data <- annotateProteins(rownames(merged.exp.complete))
rownames(protein.data) <- protein.data$uniprot

merged.elist <- new("EList", list(E = merged.exp.complete, samples = sample.data, genes = protein.data))
saveRDS(merged.elist, file = "cache/mcm_induction_proteomics.rds")
```

```
Loading required package: mzR
Loading required package: Rcpp
Loading required package: BiocParallel
Loading required package: ProtGenerics

This is MSnbase version 2.6.4 
  Visit https://lgatto.github.io/MSnbase/ to get started.


Attaching package: ‘MSnbase’

The following object is masked from ‘package:stats’:

    smooth

The following object is masked from ‘package:base’:

    trimws

Loading data...
118090 PSMs loaded.
Loading data...
144752 PSMs loaded.
The following 1489 proteins are only supported by 1 or 2 peptide spectra,
hence, protein quantification is not reliable and can only be calculated
by the 'mean' in these cases, corresponding protein accessions are:
  A0A075B6H9, A0A0B4J1Y9, A0A0B4J2F0, A0A0C4DH25, A0AVI4, A0PJW6, A0PK00, A2A288, A2RUC4, A4D1U4, A6NED2, A6NFY4, A6NI79, A6NKT7, A8MSI8, A8MW92, A9QM74, ALDOA_RABIT, B7ZAP0, O00142, O00165, O00189, O00244, O00257, O00401, O00463, O00478, O00481, O00534, O00555, O00584, O00623, O00626, O00748, O14492, O14521, O14523, O14524, O14593, O14639, O14641, O14681, O14686, O14733, O14788, O14862, O14907, O14986, O15037, O15040, O15050, O15078, O15116, O15120, O15151, O15155, O15243, O15318, O15360, O15417, O15519, O15550, O15554, O43167, O43189, O43278, O43298, O43303, O43310, O43325, O43379, O43422, O43462, O43463, O43513, O43521, O43557, O43567, O43586, O43683, O43715, O43734, O43736, O43761, O43766, O43822, O43826, O43924, O43933, O43934, O60262, O60291, O60294, O60331, O60361, O60427, O60496, O60499, O60512, O60551, O60583, O60613, O60637, O60658, O60669, O60671, O60674, O60682, O60725, O60831, O60861, O60907, O60921, O75037, O75140, O75143, O75157, O75182, O75298, O75319, O75379, O75387, O75420, O75486, O75528, O75529, O75575, O75600, O75665, O75817, O75864, O75884, O75896, O75907, O75928, O75953, O76080, O94761, O94782, O94806, O94817, O94876, O94886, O94964, O94967, O94985, O95049, O95136, O95178, O95197, O95229, O95235, O95258, O95267, O95273, O95297, O95342, O95396, O95429, O95445, O95562, O95568, O95718, O95807, O95835, O95873, O95900, O95926, O95977, O96013, O96018, P00156, P00519, P00568, P01619, P01700, P01701, P01817, P01860, P02538, P02647, P02686, P02749, P02760, P03886, P03923, P04003, P04114, P04350, P06213, P06241, P06312, P07099, P07205, P08519, P09038, P09211, P09238, P09488, P09603, P0C1Z6, P0C7X5, P0C870, P0CG12, P0CG13, P0CJ78, P0DJ07, P10114, P10147, P10244, P10276, P10301, P10643, P10914, P11498, P12074, P13196, P13598, P14151, P14543, P14621, P14649, P14784, P15018, P15104, P15260, P15289, P15621, P15735, P15822, P15848, P15923, P15924, P16260, P17029, P17252, P17405, P17483, P17535, P17544, P17927, P17947, P18084, P18433, P19419, P19634, P19793, P20292, P20336, P20674, P21266, P21675, P21926, P22415, P22830, P23025, P23443, P24001, P24557, P24588, P24863, P25208, P26374, P26951, P27487, P27815, P28289, P28749, P29374, P29992, P30291, P31431, P31785, P31947, P33897, P34059, P34931, P35609, P35790, P36402, P36954, P37275, P38398, P38936, P40425, P41273, P41440, P41743, P42331, P42336, P42338, P42685, P42771, P42773, P42898, P45844, P46019, P46092, P46108, P47813, P47974, P48551, P48668, P49069, P49116, P49336, P49356, P49419, P49448, P49641, P49674, P49747, P49759, P49760, P50151, P50539, P50542, P51451, P51452, P51606, P51617, P51687, P51688, P51797, P51808, P51957, P52435, P52657, P52740, P52758, P52926, P53370, P53677, P53778, P54253, P54652, P55042, P55061, P55082, P55198, P55201, P55211, P55854, P56181, P56199, P56211, P58557, P61165, P61218, P61457, P61599, P61960, P62253, P62324, P62491, P62714, P62875, P62877, P63098, P63172, P63272, P68402, P78317, P78381, P78410, P78536, P78537, P78539, P78543, P78549, P78560, P80748, P81877, P82921, P83369, P83876, P84022, P98170, P98173, Q00526, Q01151, Q01196, Q01459, Q01658, Q01664, Q01850, Q01970, Q02080, Q02413, Q02447, Q02487, Q03013, Q03111, Q03135, Q03154, Q03468, Q04695, Q04756, Q04941, Q05397, Q05469, Q06033, Q06413, Q07912, Q08116, Q08334, Q08426, Q08999, Q09019, Q0GE19, Q0IIM8, Q0P6H9, Q0VDG4, Q0ZGT2, Q12772, Q12778, Q12894, Q12962, Q12968, Q12974, Q12983, Q13017, Q13094, Q13129, Q13227, Q13233, Q13395, Q13415, Q13541, Q13569, Q13608, Q13614, Q13615, Q13636, Q13772, Q13825, Q13901, Q13950, Q13952, Q14055, Q14106, Q14119, Q14142, Q14147, Q14149, Q14191, Q14195, Q14207, Q14242, Q14493, Q14520, Q14568, Q14586, Q14596, Q14624, Q14644, Q14686, Q14849, Q14894, Q14993, Q14999, Q14CB8, Q14CZ7, Q14DG7, Q15032, Q15035, Q15051, Q15059, Q15063, Q15072, Q15155, Q15417, Q15526, Q15544, Q15649, Q15654, Q15669, Q15742, Q15773, Q15776, Q15782, Q15788, Q15797, Q15831, Q15853, Q16520, Q16560, Q16563, Q16611, Q16625, Q16626, Q16633, Q16670, Q16762, Q16774, Q16842, Q16864, Q17R31, Q17RS7, Q2KHM9, Q2KJY2, Q2NKJ3, Q2PPJ7, Q2TAA5, Q2TAK8, Q2TBE0, Q2VPB7, Q30134, Q30154, Q32NB8, Q32P51, Q330K2, Q3KR37, Q3KRA6, Q3MIX3, Q3SXM5, Q49B96, Q4G0I0, Q4G148, Q4KWH8, Q4LDG9, Q4U2R6, Q53FZ2, Q53GT1, Q53HC5, Q53S33, Q562F6, Q562R1, Q56NI9, Q58FG1, Q5BJF6, Q5BJH2, Q5D1E8, Q5EBM0, Q5EG05, Q5FWE3, Q5HYI7, Q5JPI3, Q5JPI9, Q5JS37, Q5JSJ4, Q5JU69, Q5JV73, Q5M7Z0, Q5MNZ9, Q5PRF9, Q5PSV4, Q5QJ74, Q5QP82, Q5SNV9, Q5SQI0, Q5SVS4, Q5SW96, Q5SXM2, Q5T0D9, Q5T1C6, Q5T3J3, Q5T3U5, Q5T4F4, Q5T5P2, Q5T5X7, Q5T749, Q5T7V8, Q5T9C2, Q5T9S5, Q5TA50, Q5TAX3, Q5TC82, Q5TGY3, Q5TGZ0, Q5THK1, Q5U3C3, Q5U649, Q5VST6, Q5VSY0, Q5VT06, Q5VU43, Q5VUG0, Q5VV52, Q5VVQ6, Q5VYV7, Q5XPI4, Q5XUX0, Q629K1, Q63HQ0, Q66K89, Q66LE6, Q68CP4, Q68CZ6, Q68DH5, Q68DK7, Q69YL0, Q6AI08, Q6DD87, Q6DKK2, Q6EEV4, Q6GMV3, Q6GPH4, Q6ICB0, Q6ICG6, Q6ICH7, Q6IEG0, Q6IPR1, Q6IPR3, Q6IQ22, Q6IQ32, Q6KCM7, Q6MZP7, Q6N063, Q6N069, Q6N075, Q6NT76, Q6NTF9, Q6NUK4, Q6NW29, Q6NXT4, Q6NXT6, Q6P0N0, Q6P161, Q6P1R3, Q6P1X5, Q6P3S1, Q6P499, Q6P4I2, Q6P589, Q6P9G4, Q6PCB7, Q6PCB8, Q6PFW1, Q6PI47, Q6PI98, Q6PID6, Q6PIY7, Q6PJ61, Q6PJW8, Q6PK18, Q6Q0C0, Q6QNY0, Q6RW13, Q6SPF0, Q6UVJ0, Q6UW56, Q6UWD8, Q6UWF3, Q6UWJ1, Q6UWP2, Q6UWU4, Q6UX65, Q6UX98, Q6V1X1, Q6VEQ5, Q6W2J9, Q6ZN55, Q6ZNC8, Q6ZNW5, Q6ZPD9, Q6ZRQ5, Q6ZSJ8, Q6ZSS7, Q6ZT21, Q6ZTQ3, Q6ZU65, Q6ZUT9, Q6ZVM7, Q709F0, Q70CQ3, Q70HW3, Q70IA6, Q70Z53, Q71RG4, Q7L1I2, Q7L211, Q7L2Z9, Q7L311, Q7L3B6, Q7L4E1, Q7L590, Q7L5Y1, Q7L7X3, Q7L8J4, Q7L8W6, Q7LBR1, Q7RTP0, Q7RTP6, Q7RTS9, Q7RTU4, Q7Z3B0, Q7Z3B3, Q7Z3E2, Q7Z3T8, Q7Z406, Q7Z412, Q7Z4F1, Q7Z4H8, Q7Z589, Q7Z5L2, Q7Z5P9, Q7Z5W3, Q7Z624, Q7Z6B0, Q7Z6M2, Q7Z6V5, Q7Z769, Q7Z7A4, Q7Z7F0, Q7Z7F7, Q7Z7L7, Q7Z7N9, Q86SQ9, Q86SX3, Q86T24, Q86TJ2, Q86TP1, Q86TS9, Q86U06, Q86U70, Q86UA6, Q86UD5, Q86UL3, Q86UU0, Q86UW6, Q86V85, Q86V88, Q86VP1, Q86VX9, Q86VZ5, Q86W34, Q86W74, Q86WI3, Q86WP2, Q86WQ0, Q86WV6, Q86WW8, Q86XN7, Q86XP1, Q86XR7, Q86Y37, Q86Y79, Q86YN1, Q86YS3, Q86YV5, Q86YZ3, Q8IU68, Q8IUC6, Q8IUW3, Q8IUX1, Q8IUZ5, Q8IVB5, Q8IVH2, Q8IVL5, Q8IW41, Q8IWB1, Q8IWB9, Q8IWD4, Q8IWE2, Q8IWE4, Q8IWR1, Q8IWT0, Q8IWT3, Q8IWV1, Q8IWX5, Q8IWY8, Q8IWY9, Q8IX15, Q8IX90, Q8IXM6, Q8IY26, Q8IY34, Q8IY57, Q8IYB1, Q8IYJ3, Q8IYL3, Q8IYS1, Q8IYT2, Q8IYW5, Q8IZ52, Q8IZA0, Q8IZF4, Q8IZQ5, Q8N0S6, Q8N0V3, Q8N0Y2, Q8N0Y7, Q8N128, Q8N137, Q8N138, Q8N142, Q8N257, Q8N2H4, Q8N2U9, Q8N2W9, Q8N302, Q8N3C7, Q8N3Z3, Q8N465, Q8N531, Q8N565, Q8N5C7, Q8N5D0, Q8N5F7, Q8N5G0, Q8N5I4, Q8N5L8, Q8N5U6, Q8N612, Q8N6L1, Q8N6S5, Q8N8N7, Q8N8Q8, Q8N8R5, Q8N8V2, Q8N954, Q8N9A8, Q8N9B5, Q8N9L9, Q8N9M5, Q8N9N5, Q8N9Q2, Q8N9V3, Q8NB14, Q8NB15, Q8NBI2, Q8NBI6, Q8NBJ9, Q8NBM8, Q8NBN3, Q8NBP0, Q8NBT0, Q8NBZ0, Q8NC42, Q8NC96, Q8NCE0, Q8NCQ2, Q8ND71, Q8ND83, Q8NDB2, Q8NDC0, Q8NDF8, Q8NDH3, Q8NDV3, Q8NDV7, Q8NDX6, Q8NDZ4, Q8NEN9, Q8NEP3, Q8NEZ2, Q8NEZ4, Q8NF64, Q8NFA0, Q8NFZ0, Q8NFZ5, Q8NGA1, Q8NHG7, Q8NHM5, Q8NHS3, Q8NHZ8, Q8NI22, Q8NI38, Q8TA86, Q8TAD4, Q8TAG9, Q8TAV0, Q8TAV4, Q8TB22, Q8TB52, Q8TB96, Q8TBE0, Q8TBF5, Q8TBZ3, Q8TBZ6, Q8TCT7, Q8TCT8, Q8TCY9, Q8TCZ2, Q8TD08, Q8TD46, Q8TDH9, Q8TDP1, Q8TDR2, Q8TDS5, Q8TDW0, Q8TE02, Q8TE04, Q8TE82, Q8TEA7, Q8TEB9, Q8TEH3, Q8TEK3, Q8TEL6, Q8TET4, Q8TF40, Q8WU10, Q8WUB8, Q8WUH1, Q8WUM9, Q8WUR7, Q8WUU4, Q8WUU5, Q8WUX2, Q8WUY8, Q8WUZ0, Q8WV44, Q8WV60, Q8WV74, Q8WV93, Q8WVD3, Q8WVI0, Q8WVK2, Q8WVP7, Q8WW01, Q8WW22, Q8WW59, Q8WWP7, Q8WWW0, Q8WX93, Q8WXD5, Q8WXW3, Q8WY36, Q8WYB5, Q8WYL5, Q8WYN0, Q8WYQ5, Q8WZA1, Q92508, Q92521, Q92562, Q92581, Q92622, Q92623, Q92624, Q92625, Q92674, Q92686, Q92802, Q92830, Q92902, Q92934, Q92968, Q92993, Q92994, Q93015, Q93075, Q969E8, Q969H6, Q969J2, Q969K3, Q969M7, Q969Q4, Q969Q6, Q969S0, Q969T4, Q969V5, Q969X0, Q969X1, Q969Y2, Q96A57, Q96AB3, Q96AE7, Q96AH0, Q96AN5, Q96AT1, Q96AT9, Q96AW1, Q96B02, Q96B23, Q96B45, Q96B96, Q96BD0, Q96BD5, Q96BD8, Q96BI1, Q96BI3, Q96BN2, Q96BP2, Q96BQ5, Q96BS2, Q96BT7, Q96BW1, Q96BY9, Q96BZ9, Q96C90, Q96CD2, Q96CJ1, Q96CN4, Q96CP7, Q96CQ1, Q96CS2, Q96CW6, Q96D31, Q96D70, Q96DC7, Q96DE5, Q96DX8, Q96E22, Q96EA4, Q96EC8, Q96ES7, Q96EU7, Q96EW2, Q96EX1, Q96EX3, Q96EY5, Q96F25, Q96F44, Q96FN4, Q96GE9, Q96GV9, Q96GY0, Q96H22, Q96H78, Q96HE9, Q96HH6, Q96HH9, Q96HJ9, Q96HR3, Q96HR8, Q96HV5, Q96I23, Q96I36, Q96I45, Q96IG2, Q96IQ9, Q96IR7, Q96IW7, Q96IY1, Q96J42, Q96J88, Q96JN8, Q96K58, Q96L93, Q96LC7, Q96LI5, Q96LL9, Q96LR5, Q96LT4, Q96LW4, Q96LW7, Q96LZ7, Q96MC6, Q96MD2, Q96MF7, Q96MG8, Q96MX3, Q96N11, Q96N46, Q96N64, Q96NB3, Q96NL6, Q96NT0, Q96NW4, Q96PC3, Q96PE7, Q96PM5, Q96PV7, Q96Q07, Q96Q27, Q96Q42, Q96Q45, Q96Q83, Q96Q89, Q96QD8, Q96QE3, Q96QF0, Q96QT6, Q96RD7, Q96RD9, Q96RJ3, Q96RK0, Q96RT8, Q96S94, Q96SL4, Q96SN7, Q96SN8, Q96SW2, Q96T21, Q99081, Q99418, Q99471, Q99487, Q99523, Q99541, Q99551, Q99595, Q99611, Q99640, Q99689, Q99717, Q99732, Q99735, Q99828, Q99871, Q99941, Q9BPY3, Q9BPZ3, Q9BQ04, Q9BQ13, Q9BQ15, Q9BQ49, Q9BQA5, Q9BQC6, Q9BQD3, Q9BQE9, Q9BQF6, Q9BQQ3, Q9BR77, Q9BRA0, Q9BRF8, Q9BRG2, Q9BRL6, Q9BRQ0, Q9BRT3, Q9BRV3, Q9BRV8, Q9BS16, Q9BSA9, Q9BSH5, Q9BSI4, Q9BT43, Q9BT73, Q9BT88, Q9BTL3, Q9BTX3, Q9BU79, Q9BU89, Q9BUB5, Q9BUE0, Q9BUH6, Q9BUK0, Q9BUV8, Q9BV10, Q9BV19, Q9BV35, Q9BV57, Q9BV94, Q9BVA0, Q9BVC4, Q9BVI0, Q9BVJ7, Q9BVK8, Q9BW66, Q9BW72, Q9BWC9, Q9BWG4, Q9BWT1, Q9BWT3, Q9BWU1, Q9BWW9, Q9BX67, Q9BXJ8, Q9BY08, Q9BY12, Q9BY41, Q9BY67, Q9BYI3, Q9BYT3, Q9BZ67, Q9BZA5, Q9BZE2, Q9BZG1, Q9BZP6, Q9BZW5, Q9C086, Q9C0B0, Q9C0C7, Q9C0D3, Q9C0F1, Q9C0H2, Q9C0I1, Q9C0K1, Q9GZN1, Q9GZN8, Q9GZP1, Q9GZT4, Q9GZU7, Q9GZY4, Q9H013, Q9H019, Q9H063, Q9H0C5, Q9H0F7, Q9H0N0, Q9H0R1, Q9H0R4, Q9H0V1, Q9H0X4, Q9H171, Q9H1A3, Q9H1C7, Q9H1D9, Q9H1J1, Q9H1K0, Q9H1N7, Q9H1X3, Q9H237, Q9H2D1, Q9H2F5, Q9H2G4, Q9H2K0, Q9H2P9, Q9H2V7, Q9H3C7, Q9H3D4, Q9H3H1, Q9H3Q1, Q9H3R0, Q9H3S4, Q9H3U5, Q9H400, Q9H410, Q9H469, Q9H4B0, Q9H4F1, Q9H4G4, Q9H4K7, Q9H4P4, Q9H5I1, Q9H5U6, Q9H6F2, Q9H6K1, Q9H6Q4, Q9H6R7, Q9H6U6, Q9H6X4, Q9H714, Q9H7B4, Q9H7C9, Q9H7H0, Q9H7M9, Q9H813, Q9H825, Q9H869, Q9H8H3, Q9H8K7, Q9H8P0, Q9H8W3, Q9H930, Q9H944, Q9H967, Q9H9A7, Q9H9G7, Q9H9H4, Q9H9L4, Q9H9S4, Q9H9Y4, Q9HA38, Q9HA72, Q9HA82, Q9HA92, Q9HAB3, Q9HAT2, Q9HAW4, Q9HBE5, Q9HBH9, Q9HBY0, Q9HC62, Q9HCG7, Q9HCI7, Q9HCM4, Q9HCN4, Q9HCU8, Q9HD47, Q9HD67, Q9NP31, Q9NP50, Q9NP80, Q9NPA8, Q9NPF0, Q9NPF2, Q9NPG3, Q9NPH0, Q9NPJ6, Q9NPR9, Q9NQ48, Q9NQ89, Q9NQA3, Q9NQC1, Q9NQG6, Q9NQG7, Q9NQQ7, Q9NQV6, Q9NR77, Q9NRA0, Q9NRB3, Q9NRC8, Q9NRF2, Q9NRG4, Q9NRP4, Q9NRQ5, Q9NRR3, Q9NRS6, Q9NRX6, Q9NRY2, Q9NRY6, Q9NS23, Q9NS73, Q9NS84, Q9NS93, Q9NSG2, Q9NSK0, Q9NSP4, Q9NSY1, Q9NTG7, Q9NTM9, Q9NU19, Q9NUA8, Q9NUG6, Q9NUI1, Q9NUK0, Q9NUM3, Q9NUP9, Q9NUS5, Q9NVA4, Q9NVE5, Q9NVN3, Q9NVV0, Q9NVV5, Q9NWH2, Q9NWS6, Q9NWT8, Q9NX00, Q9NX07, Q9NX08, Q9NX18, Q9NX38, Q9NX61, Q9NX70, Q9NX76, Q9NXC2, Q9NXF7, Q9NXJ5, Q9NXK8, Q9NXL6, Q9NXV2, Q9NXW9, Q9NY26, Q9NYA4, Q9NYB9, Q9NYJ8, Q9NYR9, Q9NYS0, Q9NYV6, Q9NZ09, Q9NZI8, Q9NZJ0, Q9NZM4, Q9NZN3, Q9P021, Q9P0B6, Q9P0J6, Q9P0M6, Q9P0S2, Q9P0U1, Q9P0W2, Q9P1U1, Q9P1W9, Q9P215, Q9P243, Q9P260, Q9P299, Q9P2E5, Q9P2G1, Q9P2H5, Q9P2M7, Q9P2S5, Q9UBC1, Q9UBP4, Q9UBQ6, Q9UBT6, Q9UBU6, Q9UBV7, Q9UBZ4, Q9UDR5, Q9UGC7, Q9UGY1, Q9UH03, Q9UHA3, Q9UHC7, Q9UHK0, Q9UHP7, Q9UHR6, Q9UHV7, Q9UI95, Q9UIK4, Q9UIW2, Q9UJC3, Q9UJJ7, Q9UJJ9, Q9UJU3, Q9UK23, Q9UK39, Q9UK45, Q9UK58, Q9UK73, Q9UK97, Q9UK99, Q9UKS7, Q9UKT4, Q9UKY1, Q9UL33, Q9UL45, Q9UL54, Q9ULE0, Q9ULG6, Q9ULH7, Q9ULI3, Q9ULJ3, Q9ULJ6, Q9ULM3, Q9ULP9, Q9ULR0, Q9ULT0, Q9UM13, Q9UM22, Q9UM82, Q9UMN6, Q9UMR2, Q9UMW8, Q9UMX0, Q9UNK9, Q9UNW9, Q9UPQ8, Q9UPT9, Q9UPV0, Q9UPY5, Q9UPY8, Q9UQ88, Q9UQL6, Q9Y244, Q9Y248, Q9Y252, Q9Y256, Q9Y259, Q9Y289, Q9Y2A7, Q9Y2B1, Q9Y2C9, Q9Y2E8, Q9Y2G2, Q9Y2G5, Q9Y2I8, Q9Y2K6, Q9Y2R2, Q9Y2S0, Q9Y2V2, Q9Y2W6, Q9Y2Y0, Q9Y2Y4, Q9Y2Y9, Q9Y2Z9, Q9Y303, Q9Y3B8, Q9Y3C0, Q9Y3C4, Q9Y3C7, Q9Y3D0, Q9Y3D2, Q9Y3E0, Q9Y3E7, Q9Y3P4, Q9Y3T6, Q9Y421, Q9Y483, Q9Y484, Q9Y4E1, Q9Y4E5, Q9Y4F3, Q9Y4G2, Q9Y4G8, Q9Y4K3, Q9Y576, Q9Y587, Q9Y5J5, Q9Y5J9, Q9Y5N5, Q9Y5N6, Q9Y5S2, Q9Y5T4, Q9Y5W7, Q9Y5Z7, Q9Y616, Q9Y620, Q9Y657, Q9Y672, Q9Y6A9, Q9Y6B7, Q9Y6G5, Q9Y6I8, Q9Y6J0, REF_HEVBR
'select()' returned 1:many mapping between keys and columns
'select()' returned 1:many mapping between keys and columns
'select()' returned 1:many mapping between keys and columns
The following 1593 proteins are only supported by 1 or 2 peptide spectra,
hence, protein quantification is not reliable and can only be calculated
by the 'mean' in these cases, corresponding protein accessions are:
  A0A075B6I0, A0A0A0MS15, A0A0B4J1Y9, A0A0C4DH34, A0AVF1, A0JLT2, A0PJW6, A0PK00, A1L157, A1L188, A2RTX5, A2RU54, A2VEC9, A5A3E0, A5PLL7, A6H8Y1, A6NCL7, A6NFY4, A6NFY7, A6NHT5, A6NJG2, A6NK58, A6NKF1, A8MSI8, A8MW92, A9YTQ3, B7ZAP0, CASB_BOVIN, O00160, O00244, O00257, O00291, O00311, O00391, O00507, O00560, O00626, O00628, O00716, O14490, O14521, O14602, O14607, O14678, O14681, O14713, O14763, O14788, O14795, O14817, O14862, O14867, O14904, O14907, O14983, O14986, O15015, O15078, O15120, O15121, O15151, O15156, O15169, O15182, O15264, O15304, O15318, O15360, O15392, O15417, O15431, O15440, O15534, O15552, O15554, O43187, O43189, O43310, O43325, O43426, O43463, O43502, O43504, O43513, O43567, O43677, O43715, O43734, O43739, O43759, O43766, O43791, O43826, O43829, O43854, O43903, O43909, O43924, O43933, O43934, O60268, O60291, O60336, O60361, O60486, O60496, O60499, O60512, O60513, O60583, O60637, O60658, O60674, O60682, O60704, O60825, O60831, O60858, O60888, O60906, O60907, O60921, O75054, O75063, O75071, O75081, O75140, O75144, O75157, O75167, O75317, O75363, O75379, O75387, O75394, O75398, O75438, O75460, O75486, O75564, O75570, O75575, O75582, O75600, O75626, O75716, O75808, O75817, O75864, O75865, O75896, O75907, O75912, O75953, O76080, O76095, O94761, O94817, O94864, O94876, O94885, O94887, O94889, O94921, O94952, O94964, O94967, O94985, O95059, O95136, O95166, O95178, O95197, O95199, O95208, O95258, O95267, O95297, O95302, O95399, O95402, O95415, O95427, O95429, O95445, O95450, O95476, O95498, O95568, O95644, O95807, O95825, O95835, O95977, O96018, P00156, P00395, P00414, P00568, P00734, P01033, P01034, P01116, P01130, P01619, P01701, P01705, P01782, P01893, P02538, P02647, P02749, P02760, P03923, P04003, P04114, P04156, P04220, P04222, P04229, P04259, P04278, P04350, P04637, P04921, P05106, P05412, P05543, P05546, P06737, P07205, P07477, P07478, P07585, P07942, P08048, P08519, P08603, P08697, P09017, P09038, P09211, P09237, P09455, P09486, P09912, P0C1Z6, P0C7T5, P0C7X5, P0CG13, P0DJ07, P10071, P10074, P10109, P10244, P10253, P10276, P10301, P10415, P10620, P10914, P11474, P11831, P12074, P12109, P12259, P13056, P13196, P13497, P13521, P13686, P13762, P13929, P14151, P14543, P14621, P14784, P14923, P15018, P15151, P15289, P15735, P15822, P15924, P16455, P17010, P17029, P17036, P17483, P17813, P17947, P18084, P18846, P19419, P19532, P19634, P19793, P20333, P20930, P21266, P21589, P21854, P21926, P22415, P23025, P23434, P23469, P23508, P23511, P23634, P24278, P24588, P24863, P25054, P25208, P26374, P26951, P28328, P29120, P30291, P30307, P30501, P30711, P31431, P31629, P31641, P31947, P33552, P33897, P34931, P34947, P35222, P36402, P36956, P38435, P38936, P39060, P39880, P40337, P41162, P41217, P41440, P42685, P45844, P45877, P45984, P46527, P47224, P48060, P48061, P48378, P48551, P48995, P49069, P49407, P49459, P49674, P49760, P49789, P49795, P49810, P50053, P50135, P50219, P51157, P51606, P51684, P51688, P51790, P51884, P51957, P52655, P52657, P52739, P52740, P52758, P52823, P52926, P53671, P53778, P53803, P53804, P53814, P54852, P55055, P55061, P55211, P55347, P55789, P55854, P56181, P56270, P56282, P56557, P57086, P57087, P57729, P57775, P58557, P59780, P60006, P60059, P60604, P61073, P61165, P61218, P61599, P62068, P62253, P62699, P62714, P62877, P63027, P78312, P78317, P78381, P78537, P78560, P79522, P80404, P81605, P81877, P83369, P84022, P84243, P98170, Q01196, Q01459, Q01664, Q01850, Q02318, Q02413, Q02539, Q02930, Q03111, Q03135, Q03154, Q03167, Q03468, Q05469, Q05682, Q05BQ5, Q06609, Q07000, Q07011, Q07352, Q07889, Q07890, Q07912, Q08116, Q08117, Q08334, Q08AD1, Q09327, Q0GE19, Q0P6D6, Q0P6H9, Q0VDG4, Q12766, Q12767, Q12772, Q12774, Q12815, Q12887, Q12894, Q12913, Q12962, Q12974, Q12983, Q13127, Q13158, Q13227, Q13277, Q13286, Q13515, Q13569, Q13608, Q13637, Q13641, Q13794, Q13825, Q14055, Q14116, Q14119, Q14164, Q14242, Q14324, Q14410, Q14442, Q14469, Q14520, Q14568, Q14624, Q14644, Q14680, Q14686, Q14687, Q14693, Q14849, Q14865, Q14872, Q14894, Q14964, Q149N8, Q14CB8, Q14CZ0, Q15116, Q15131, Q15155, Q15170, Q15334, Q15468, Q15545, Q15649, Q15746, Q15788, Q15797, Q15836, Q15907, Q16513, Q16520, Q16533, Q16560, Q16584, Q16594, Q16595, Q16626, Q16635, Q16842, Q16864, Q17R31, Q17R98, Q17RN3, Q17RS7, Q2KHM9, Q2KHR2, Q2KHR3, Q2LD37, Q2NKJ3, Q2PZI1, Q2T9J0, Q2TAA2, Q2TAA5, Q2TAK8, Q2V2M9, Q2YD98, Q32M88, Q32P51, Q330K2, Q3B7J2, Q3B820, Q3KQZ1, Q3KRA6, Q3KRA9, Q3L8U1, Q3MII6, Q49AG3, Q49AM3, Q49B96, Q4G0X4, Q4KWH8, Q4VC44, Q502W6, Q53F39, Q53FV1, Q53FZ2, Q53GT1, Q53HV7, Q53RE8, Q53S33, Q53TN4, Q58FG1, Q5BJF6, Q5BKX5, Q5D1E8, Q5FWE3, Q5FWF5, Q5H8A4, Q5HYI8, Q5JPI9, Q5JS54, Q5JSJ4, Q5JU69, Q5JV73, Q5M7Z0, Q5MNZ6, Q5PRF9, Q5QJ74, Q5QP82, Q5SNV9, Q5SQI0, Q5SR56, Q5SXM2, Q5SXM8, Q5T0D9, Q5T3F8, Q5T3J3, Q5T3U5, Q5T5C0, Q5T6S3, Q5T749, Q5T750, Q5T7M9, Q5T7V8, Q5T9C2, Q5T9S5, Q5TA50, Q5TC82, Q5TEU4, Q5TF39, Q5TF58, Q5TGY1, Q5TGY3, Q5TGZ0, Q5U3C3, Q5VST6, Q5VSY0, Q5VVQ6, Q5VWC8, Q5VWJ9, Q5VYV7, Q5VZI3, Q5W0U4, Q5W111, Q5XKK7, Q5XPI4, Q5XUX0, Q5XUX1, Q63HK3, Q63HQ0, Q66K64, Q66K79, Q66K89, Q68DH5, Q69YI7, Q69YU5, Q6AI08, Q6AZZ1, Q6DCA0, Q6DD87, Q6DN14, Q6EEV6, Q6GPH4, Q6GPH6, Q6IC98, Q6ICH7, Q6ICL3, Q6IE81, Q6IPR3, Q6IQ22, Q6MZP7, Q6N021, Q6N043, Q6N063, Q6NSI4, Q6NT16, Q6NTE8, Q6NTF9, Q6NUK4, Q6NUS6, Q6NW29, Q6NWY9, Q6NXT4, Q6NXT6, Q6P1M9, Q6P1X5, Q6P3S6, Q6P4F2, Q6P4F7, Q6P4I2, Q6P9G4, Q6P9H5, Q6PCB7, Q6PH81, Q6PHR2, Q6PI47, Q6PI98, Q6PID8, Q6PII3, Q6PIW4, Q6PJ61, Q6PJW8, Q6Q0C0, Q6R327, Q6RI45, Q6RW13, Q6UB98, Q6UB99, Q6UUV7, Q6UW56, Q6UWD8, Q6UWJ1, Q6UWP2, Q6UWS5, Q6UWU4, Q6UWZ7, Q6UX07, Q6UX98, Q6V0I7, Q6ZMG9, Q6ZN18, Q6ZN66, Q6ZNC8, Q6ZNW5, Q6ZPD9, Q6ZS17, Q6ZS86, Q6ZSS7, Q6ZT21, Q6ZU65, Q6ZUT9, Q6ZUX7, Q6ZVK8, Q6ZXV5, Q709F0, Q70CQ3, Q70HW3, Q71RG4, Q71UM5, Q76I76, Q7KYR7, Q7L0J3, Q7L590, Q7L7X3, Q7L8J4, Q7LFL8, Q7RTN6, Q7RTR2, Q7RTU4, Q7Z2K8, Q7Z309, Q7Z3B0, Q7Z3B3, Q7Z3D4, Q7Z3K6, Q7Z3Q1, Q7Z3T8, Q7Z3V4, Q7Z412, Q7Z4F1, Q7Z4H8, Q7Z4L5, Q7Z4V0, Q7Z5U6, Q7Z5W3, Q7Z614, Q7Z6B0, Q7Z6J0, Q7Z6J9, Q7Z6K3, Q7Z6V5, Q7Z736, Q7Z769, Q7Z7B0, Q7Z7C8, Q7Z7K0, Q86SK9, Q86SQ7, Q86T96, Q86TJ2, Q86TL2, Q86TS9, Q86TW2, Q86U06, Q86UA6, Q86UD5, Q86UT8, Q86UU0, Q86UW6, Q86UX6, Q86UY6, Q86VI3, Q86VP1, Q86VQ1, Q86VV8, Q86VX9, Q86VZ1, Q86VZ5, Q86W34, Q86W74, Q86WI3, Q86WP2, Q86WQ0, Q86WV6, Q86WW8, Q86WZ6, Q86X40, Q86X52, Q86XK3, Q86XR7, Q86XS8, Q86Y37, Q86Y91, Q86YC3, Q86YS3, Q8IU60, Q8IU68, Q8IUW3, Q8IV08, Q8IV20, Q8IVB4, Q8IVB5, Q8IVF5, Q8IVQ6, Q8IVV7, Q8IW41, Q8IWE5, Q8IWL3, Q8IWP9, Q8IWT6, Q8IWU2, Q8IWV1, Q8IX18, Q8IX90, Q8IXJ6, Q8IXK0, Q8IXQ3, Q8IXQ5, Q8IXZ2, Q8IY22, Q8IY34, Q8IY92, Q8IYH5, Q8IYL2, Q8IYL3, Q8IYN0, Q8IYS0, Q8IZ52, Q8IZD2, Q8IZF4, Q8IZM8, Q8IZN3, Q8N0S6, Q8N0Y2, Q8N0Y7, Q8N0Z8, Q8N129, Q8N138, Q8N1B3, Q8N1G1, Q8N1N4, Q8N257, Q8N2A8, Q8N2I9, Q8N2Z9, Q8N300, Q8N335, Q8N370, Q8N3C7, Q8N3J3, Q8N3J5, Q8N413, Q8N474, Q8N4H5, Q8N4N3, Q8N4P3, Q8N4S0, Q8N531, Q8N554, Q8N5C7, Q8N5G0, Q8N5I2, Q8N5L8, Q8N5U6, Q8N5Y8, Q8N612, Q8N680, Q8N6L1, Q8N755, Q8N7R7, Q8N8D1, Q8N8E1, Q8N8N7, Q8N8Q9, Q8N8R3, Q8N8V2, Q8N960, Q8N961, Q8N9A8, Q8N9N5, Q8N9Q2, Q8NA29, Q8NAF0, Q8NB14, Q8NB15, Q8NB37, Q8NB50, Q8NB78, Q8NB91, Q8NBI2, Q8NBK3, Q8NBM8, Q8NBT0, Q8NBZ0, Q8NC42, Q8NC96, Q8NCC3, Q8NCD3, Q8NCE0, Q8NCG7, Q8NCQ2, Q8ND25, Q8ND83, Q8NDB2, Q8NDC0, Q8NDV3, Q8NDX6, Q8NE00, Q8NEM7, Q8NEV4, Q8NEZ3, Q8NFV4, Q8NFZ0, Q8NFZ5, Q8NG08, Q8NG11, Q8NG68, Q8NHS3, Q8NHY2, Q8NI22, Q8NI37, Q8NI38, Q8NI60, Q8TAG9, Q8TAP6, Q8TAU3, Q8TAV0, Q8TB96, Q8TBF4, Q8TBK2, Q8TBK6, Q8TBR7, Q8TBZ3, Q8TC44, Q8TCT7, Q8TCT8, Q8TCY9, Q8TCZ2, Q8TD08, Q8TD22, Q8TD26, Q8TD57, Q8TDG2, Q8TDN4, Q8TDR0, Q8TDY2, Q8TE04, Q8TED4, Q8TEK3, Q8TEQ8, Q8TEV9, Q8TEY7, Q8TF40, Q8TF50, Q8TF76, Q8WTW4, Q8WUB8, Q8WUH1, Q8WUM9, Q8WUN7, Q8WUR7, Q8WUU4, Q8WUX2, Q8WUY3, Q8WUY8, Q8WUY9, Q8WUZ0, Q8WV19, Q8WV74, Q8WV93, Q8WVD3, Q8WVE0, Q8WVK7, Q8WVN8, Q8WVR3, Q8WVX3, Q8WWZ7, Q8WX93, Q8WXW3, Q8WXX7, Q8WY91, Q8WYB5, Q8WYH8, Q8WYK2, Q8WYL5, Q8WYQ3, Q8WZ64, Q8WZA1, Q92478, Q92539, Q92546, Q92551, Q92562, Q92569, Q92624, Q92686, Q92783, Q92802, Q92823, Q92830, Q92870, Q92871, Q92902, Q92934, Q92963, Q92966, Q92968, Q92993, Q93075, Q93091, Q93096, Q95460, Q969E2, Q969E8, Q969H0, Q969K3, Q969M3, Q969M7, Q969Q6, Q969R5, Q969R8, Q969T4, Q969T7, Q969T9, Q969W1, Q969X1, Q969Y2, Q96A19, Q96A25, Q96A29, Q96A46, Q96A54, Q96AB6, Q96AD5, Q96AH0, Q96AP0, Q96AT9, Q96AW1, Q96B01, Q96B45, Q96B70, Q96B96, Q96BI1, Q96BI3, Q96BN2, Q96BP2, Q96BQ5, Q96BS2, Q96BT3, Q96BU1, Q96BY9, Q96BZ4, Q96BZ9, Q96C00, Q96C55, Q96C90, Q96CF2, Q96CJ1, Q96CN4, Q96CP7, Q96CQ1, Q96CW6, Q96D31, Q96DF8, Q96DT6, Q96DX7, Q96E16, Q96EC8, Q96ED9, Q96EK4, Q96EM0, Q96EQ0, Q96EU7, Q96EW2, Q96EX3, Q96EY5, Q96F25, Q96FV2, Q96G28, Q96GE9, Q96GR4, Q96GY3, Q96GZ6, Q96H22, Q96H55, Q96H78, Q96H96, Q96HH6, Q96HI0, Q96HJ9, Q96HN2, Q96HP0, Q96HQ2, Q96HR8, Q96HU1, Q96I23, Q96I34, Q96I45, Q96IG2, Q96IK0, Q96IL0, Q96IM9, Q96IW7, Q96IY1, Q96IZ5, Q96J88, Q96JC9, Q96JT2, Q96JY6, Q96K12, Q96K19, Q96K80, Q96KC2, Q96KF7, Q96L93, Q96L94, Q96LA8, Q96LC7, Q96LC9, Q96LL9, Q96LT7, Q96LU5, Q96LW4, Q96MD2, Q96MD7, Q96MF7, Q96MT8, Q96N11, Q96N21, Q96N64, Q96ND0, Q96NL6, Q96NM4, Q96NR8, Q96NT0, Q96P53, Q96PE7, Q96PM9, Q96PU5, Q96PV7, Q96Q07, Q96Q42, Q96Q45, Q96Q80, Q96QF0, Q96QT6, Q96RJ3, Q96RK0, Q96RR1, Q96S19, Q96S53, Q96S99, Q96SL4, Q96SN7, Q96SW2, Q99081, Q99471, Q99496, Q99519, Q99523, Q99541, Q99551, Q99572, Q99583, Q99607, Q99622, Q99640, Q99732, Q99735, Q99757, Q99766, Q99941, Q99942, Q9BPU6, Q9BPX7, Q9BPZ3, Q9BPZ7, Q9BQ04, Q9BQ15, Q9BQ24, Q9BQ49, Q9BQ69, Q9BQB6, Q9BQD3, Q9BQD7, Q9BQE9, Q9BQG2, Q9BQP9, Q9BR77, Q9BRL6, Q9BRN9, Q9BRP1, Q9BRQ0, Q9BRV3, Q9BRX9, Q9BS16, Q9BSA4, Q9BSA9, Q9BSE5, Q9BST9, Q9BT43, Q9BT73, Q9BTE1, Q9BTF0, Q9BTK6, Q9BTM9, Q9BTX3, Q9BU02, Q9BU64, Q9BU79, Q9BU89, Q9BUB4, Q9BUB5, Q9BUE0, Q9BUE6, Q9BUK0, Q9BUL5, Q9BUM1, Q9BUV8, Q9BV10, Q9BV57, Q9BV94, Q9BVA1, Q9BVL4, Q9BVM2, Q9BVM4, Q9BVT8, Q9BVV6, Q9BW66, Q9BWG4, Q9BWM7, Q9BWW4, Q9BX69, Q9BX70, Q9BXH1, Q9BXK1, Q9BXM9, Q9BXS4, Q9BXT2, Q9BXV9, Q9BY08, Q9BY12, Q9BY41, Q9BY76, Q9BY84, Q9BY89, Q9BYE7, Q9BYN0, Q9BZK3, Q9BZP6, Q9BZW5, Q9C086, Q9C0B9, Q9C0C4, Q9C0D5, Q9C0D7, Q9GZN8, Q9GZP1, Q9GZU3, Q9GZX7, Q9H013, Q9H0N5, Q9H0R3, Q9H0U9, Q9H0X4, Q9H116, Q9H165, Q9H171, Q9H1A3, Q9H1B5, Q9H1C7, Q9H1J1, Q9H1K0, Q9H1N7, Q9H1P3, Q9H1Z4, Q9H237, Q9H2D6, Q9H2F5, Q9H2G4, Q9H2G9, Q9H2L5, Q9H2X8, Q9H2Y9, Q9H3C7, Q9H3D4, Q9H3H1, Q9H3J6, Q9H3L0, Q9H3M7, Q9H3R2, Q9H3S4, Q9H3S5, Q9H3U5, Q9H425, Q9H469, Q9H477, Q9H4B7, Q9H4P4, Q9H4Z2, Q9H582, Q9H5H4, Q9H5K3, Q9H5U6, Q9H649, Q9H6F2, Q9H6P5, Q9H6R6, Q9H6R7, Q9H6S3, Q9H6X4, Q9H714, Q9H777, Q9H7C9, Q9H7H0, Q9H7M6, Q9H7M9, Q9H7X7, Q9H813, Q9H825, Q9H840, Q9H892, Q9H8E8, Q9H8G1, Q9H8K7, Q9H8M7, Q9H8P0, Q9H8W3, Q9H930, Q9H977, Q9H9F9, Q9H9H4, Q9H9L4, Q9HA47, Q9HAT2, Q9HAV0, Q9HAW0, Q9HB09, Q9HB20, Q9HBH9, Q9HBK9, Q9HBM6, Q9HC44, Q9HC52, Q9HC62, Q9HCE9, Q9HCL2, Q9HCM1, Q9HCM4, Q9HCN4, Q9HCU9, Q9HD47, Q9HD67, Q9NP87, Q9NPA5, Q9NPA8, Q9NPD8, Q9NPE2, Q9NPF2, Q9NPI8, Q9NPY3, Q9NQ89, Q9NQ92, Q9NQA3, Q9NQG1, Q9NQG7, Q9NQL2, Q9NQR1, Q9NQV6, Q9NR77, Q9NR82, Q9NR96, Q9NR99, Q9NRA0, Q9NRC8, Q9NRF2, Q9NRF9, Q9NRG0, Q9NRG4, Q9NRN9, Q9NRP4, Q9NRQ5, Q9NRR3, Q9NRR8, Q9NRV9, Q9NRX6, Q9NRY2, Q9NRY6, Q9NS73, Q9NSD4, Q9NSG2, Q9NSY2, Q9NTG7, Q9NTN3, Q9NTW7, Q9NTX7, Q9NU23, Q9NUA8, Q9NUJ3, Q9NUM3, Q9NUN5, Q9NUP1, Q9NUS5, Q9NVA4, Q9NVC3, Q9NVE5, Q9NVK5, Q9NVN3, Q9NVR0, Q9NVR5, Q9NVV5, Q9NVW2, Q9NW81, Q9NWR8, Q9NWS6, Q9NWS9, Q9NWX5, Q9NX05, Q9NX08, Q9NX18, Q9NX38, Q9NX61, Q9NXA8, Q9NXD2, Q9NXF7, Q9NXL6, Q9NXV2, Q9NXW9, Q9NY97, Q9NYA4, Q9NYB9, Q9NYG2, Q9NYJ1, Q9NYJ8, Q9NYP9, Q9NYR9, Q9NYS0, Q9NZ71, Q9NZC7, Q9NZM4, Q9NZN3, Q9P003, Q9P021, Q9P0M6, Q9P0N9, Q9P0P8, Q9P0S2, Q9P0T7, Q9P0U1, Q9P0V9, Q9P0W2, Q9P1F3, Q9P1U1, Q9P1W9, Q9P1Y5, Q9P209, Q9P215, Q9P243, Q9P2B4, Q9P2G9, Q9P2H5, Q9P2J3, Q9P2M7, Q9P2N6, Q9P2P5, Q9P2P6, Q9P2Q2, Q9P2S5, Q9P2X3, Q9UBC1, Q9UBH6, Q9UBI4, Q9UBI6, Q9UBJ2, Q9UBU6, Q9UBV7, Q9UBZ4, Q9UDR5, Q9UF56, Q9UGK3, Q9UGL1, Q9UH92, Q9UHA4, Q9UHB7, Q9UHC1, Q9UHC7, Q9UHH9, Q9UHJ3, Q9UHJ9, Q9UHP7, Q9UHW5, Q9UI95, Q9UIA0, Q9UIE0, Q9UIF8, Q9UIL8, Q9UIW2, Q9UJJ7, Q9UJJ9, Q9UJT0, Q9UK10, Q9UK97, Q9UKF7, Q9UKJ1, Q9UKL3, Q9UKT4, Q9UKV0, Q9UL01, Q9UL33, Q9ULC3, Q9ULE6, Q9ULH7, Q9ULJ6, Q9ULL1, Q9UM13, Q9UM22, Q9UMW8, Q9UNW9, Q9UNX3, Q9UPG8, Q9UPM9, Q9UPS8, Q9UPT6, Q9UPW0, Q9UQ16, Q9UQ88, Q9Y248, Q9Y252, Q9Y253, Q9Y256, Q9Y2D2, Q9Y2D4, Q9Y2F5, Q9Y2I8, Q9Y2K2, Q9Y2K6, Q9Y2M5, Q9Y2N7, Q9Y2R2, Q9Y2S0, Q9Y2S2, Q9Y2U5, Q9Y2U9, Q9Y2V2, Q9Y2Y0, Q9Y2Y1, Q9Y2Y9, Q9Y365, Q9Y3A0, Q9Y3B6, Q9Y3B8, Q9Y3C5, Q9Y3C7, Q9Y3D5, Q9Y3E0, Q9Y3E2, Q9Y3E7, Q9Y3P4, Q9Y3R5, Q9Y4C2, Q9Y4C5, Q9Y4G2, Q9Y4G8, Q9Y4K0, Q9Y4U1, Q9Y4X0, Q9Y535, Q9Y548, Q9Y576, Q9Y584, Q9Y592, Q9Y5J5, Q9Y5L3, Q9Y5N5, Q9Y5N6, Q9Y5T4, Q9Y5W7, Q9Y5W8, Q9Y5Z7, Q9Y605, Q9Y616, Q9Y623, Q9Y651, Q9Y680, Q9Y689, Q9Y692, Q9Y6A1, Q9Y6B6, Q9Y6I8, Q9Y6M4, Q9Y6N7, Q9Y6T7, REF_HEVBR
'select()' returned 1:many mapping between keys and columns
'select()' returned 1:many mapping between keys and columns
'select()' returned 1:many mapping between keys and columns
'select()' returned 1:many mapping between keys and columns
'select()' returned 1:many mapping between keys and columns
'select()' returned 1:many mapping between keys and columns
```

Create nicer sample labels:

In [8]:

```
proteomics.el <- readRDS("cache/mcm_induction_proteomics.rds")

# add nicer sample names
colnames(proteomics.el) <- paste0(proteomics.el$samples$patient.id, "_",
                                  proteomics.el$samples$cell.type, "_",
                                  proteomics.el$samples$condition)
rownames(proteomics.el$samples) <- colnames(proteomics.el)

# set "MOCK" as reference
proteomics.el$samples$condition <- relevel(proteomics.el$samples$condition, "MOCK")

# also change to Gene
message("Values for ", nrow(proteomics.el), " proteins")

total.expr <- rowSums(proteomics.el$E, na.rm = T)
proteomics.el <- proteomics.el[order(total.expr, decreasing = T), ]
proteomics.el <- proteomics.el[!is.na(proteomics.el$genes$Gene) & !duplicated(proteomics.el$genes$Gene), ]
rownames(proteomics.el) <- proteomics.el$genes$Gene

message(nrow(proteomics.el), " proteins after change to Gene identifier")
```

```
Values for 6456 proteins
6399 proteins after change to Gene identifier
```

Perform a basic PCA:

In [9]:

```
exprs <- proteomics.el$E
# remove missing proteins
exprs <- exprs[rowSums(is.na(exprs)) == 0, ]
proteomics.pca <- prcomp(exprs)
proteomics.pca.sum <- summary(proteomics.pca)

plot.data <- data.frame(proteomics.pca$rotation)
plot.data <- merge(plot.data, proteomics.el$samples, by.x = 0, by.y = 0)
plot.data$condition <- relevel(plot.data$condition, "MOCK")

plot.xlab <- paste0("PC1 (", round(proteomics.pca.sum[["importance"]]["Proportion of Variance", "PC1"] * 100, 1), "%)")
plot.ylab <- paste0("PC2 (", round(proteomics.pca.sum[["importance"]]["Proportion of Variance", "PC2"] * 100, 1), "%)")

setPlotSize(3,5)
ggplot(plot.data, aes(x = PC1, y = PC2, color = patient.id, shape = cell.type)) +
    geom_point() +
    scale_color_brewer(palette = "Set1") +
    labs(color = "Patient", shape = "Cell type", x = plot.xlab, y = plot.ylab) +
    my_theme_bw

plot.obj <- ggplot(plot.data, aes(x = PC1, y = PC2, color = condition, shape = cell.type)) +
    geom_point() +
    scale_color_brewer(palette = "Set1") +
    labs(color = "Treatment", shape = "Cell type", x = plot.xlab, y = plot.ylab) +
    my_theme()

plot.obj

# save the svg
svg("plots/Figure_2A-Proteomics_PCA.svg", width=4, height=3)
print(plot.obj)
dev.off()

resetPlotSize()
```

**png:** 2

Additional components:

In [10]:

```
plot.xlab <- paste0("PC3 (", round(proteomics.pca.sum[["importance"]]["Proportion of Variance", "PC3"] * 100, 1), "%)")
plot.ylab <- paste0("PC4 (", round(proteomics.pca.sum[["importance"]]["Proportion of Variance", "PC4"] * 100, 1), "%)")

plot.obj <- ggplot(plot.data, aes(x = PC3, y = PC4, color = condition, shape = cell.type)) +
    geom_point() +
    scale_color_brewer(palette = "Set1") +
    labs(color = "Treatment", shape = "Cell type", x = plot.xlab, y = plot.ylab) +
    my_theme_bw

plot.obj
```

Most importantly, there was again no difference between the two cell types (shapes). For the proteomics setup this is especially important since the two cell types were analysed in two separate MS/MS runs.

In the proteomics data, the treatment effect is considerably more distinct than in the RNASeq data.

## Phospho-proteomics data¶

In [11]:

```
fixPtmNames <- function(ptm.strings) {
    # remove PTMs we don't care about - TMT tag and carbamidomethylation
    ptm.strings <- gsub("[0-9]+-229.162932;?", "", ptm.strings)
    ptm.strings <- gsub("[0-9]+-229.1629;?", "", ptm.strings)
    ptm.strings <- gsub("[0-9]+-57.021463735;?", "", ptm.strings)
    ptm.strings <- gsub("[0-9]+-57.022;?", "", ptm.strings)
    ptm.strings <- gsub(";$", "", ptm.strings)
    ptm.strings <- gsub(";", ",", ptm.strings)
    ptm.strings <- gsub("#", ";", ptm.strings)
    
    # fix PTM definitions
    #MOD:00696
    ptm.strings <- gsub("([0-9]+)-79.96633092500001", "MOD:00696:\\1", ptm.strings)
    ptm.strings <- gsub("([0-9]+)-79.9663", "MOD:00696:\\1", ptm.strings)
    
    # MOD:00400
    ptm.strings <- gsub("([0-9]+)-0.9840155950000007", "MOD:00400:\\1", ptm.strings)
    ptm.strings <- gsub("([0-9]+)-0.984", "MOD:00400:\\1", ptm.strings)
    
    # MOD:00425
    ptm.strings <- gsub("([0-9]+)-15.99491463", "MOD:00425:\\1", ptm.strings)
    ptm.strings <- gsub("([0-9]+)-15.9949", "MOD:00425:\\1", ptm.strings)
    
    return(ptm.strings)
}

doIPQFPhosphoQuant <- function(msnset, min.psms = 2) {
    has.missing.values <- (rowSums(is.na(exprs(msnset))) > 0 | rowSums(exprs(msnset) == 0)) > 0    
    msnset <- filterNA(msnset,0.0)
    
    # create a unique sequence tag
    fData(msnset)$sequence_mod <- paste0(fData(msnset)$sequence, "#", fData(msnset)$modifications)
    fData(msnset)$sequence_mod <- fixPtmNames(fData(msnset)$sequence_mod)
    
    # get the min psms
    if (min.psms > 1) {
        num.psms <- table(fData(msnset)$sequence_mod)
        peptides.to.remove <- names(num.psms[num.psms < min.psms])
        msnset <- msnset[!fData(msnset)$sequence_mod %in% peptides.to.remove]
    }
    
    allProts <- combineFeatures(msnset, fun="iPQF",
                                groupBy=fData(msnset)$sequence_mod, 
                                verbose=F)
    
    exprs(allProts) <- log2(exprs(allProts))
    
    # add sample annotations
    pData(allProts) <- pData(msnset)[colnames(allProts), ]
    pData(allProts)$condition <- factor(pData(allProts)$condition, levels = c("MOCK", "MCM"), labels = c("MOCK", "MCM"))
    pData(allProts)$patient.id <- gsub("([A-Z])([A-Z])", "\\1-\\2", pData(allProts)$patient.id)
    
    pData(allProts)$sample_name <- paste(pData(allProts)$patient.id, pData(allProts)$condition, pData(allProts)$cell.type)
    
    return(allProts)
}

msnset.pbmcb <- loadAsMsnSet("data/TMT1_TiO2.quant.tsv.gz")
msnset.tibc <- loadAsMsnSet("data/TMT2_TiO2.quant.tsv.gz")

prots.pbmcb <- doIPQFPhosphoQuant(msnset.pbmcb)
prots.tibc <- doIPQFPhosphoQuant(msnset.tibc)

sampleNames(prots.pbmcb) <- pData(prots.pbmcb)$sample_name
sampleNames(prots.tibc) <- pData(prots.tibc)$sample_name

# merge protein quants
merged.exp <- merge(exprs(prots.pbmcb), exprs(prots.tibc), all.x = T, all.y = T, by.x = 0, by.y = 0)
rownames(merged.exp) <- merged.exp$Row.names
merged.exp$Row.names <- NULL

sample.data <- data.frame(
    row.names = colnames(merged.exp),
    patient.id = gsub(" .*", "", colnames(merged.exp)),
    condition = gsub("[^ ]* ([^ ]*) .*", "\\1", colnames(merged.exp)),
    cell.type = gsub(".* ", "", colnames(merged.exp))
)

# remove all missing values
merged.exp.complete <- merged.exp[rowSums(is.na(merged.exp)) == 0, ]

# find the protein data
protein.accs <- ifelse(is.na(fData(prots.pbmcb)[rownames(merged.exp.complete), "accession"]), 
                          fData(prots.tibc)[rownames(merged.exp.complete), "accession"], 
                          fData(prots.pbmcb)[rownames(merged.exp.complete), "accession"])

# create a limma object again
protein.data <- annotateProteins(protein.accs)
rownames(protein.data) <- rownames(merged.exp.complete)

merged.phospho.elist <- new("EList", list(E = merged.exp.complete, samples = sample.data, genes = protein.data))

saveRDS(merged.phospho.elist, file = "cache/mcm_induction_proteomics_phospho.rds")
```

```
Loading data...
32272 PSMs loaded.
24549 phospho PSMs loaded.
Loading data...
19306 PSMs loaded.
18675 phospho PSMs loaded.
The following 1248 proteins are only supported by 1 or 2 peptide spectra,
hence, protein quantification is not reliable and can only be calculated
by the 'mean' in these cases, corresponding protein accessions are:
  Q6SPF0, Q96A47, P49006, Q9H4A6, Q14978, O75694, Q8IYH5, P59923, Q86U86, O00116, O75367, Q14669, Q5VSL9, Q8IYB3, Q5TCZ1, O15127, Q5BKY9, Q92538, P61927, P49768, Q9UFC0, O43290, P55196, Q96C19, Q01543, Q9Y388, Q99704, Q96S94, Q5T1R4, Q9NW97, O75569, P29966, Q15651, P27824, Q9UGU0, Q9UK58, Q15459, Q92925, Q9NYF8, Q9P2E9, Q9NZB2, P61158, Q86UE8, Q8NE71, Q9BUA3, Q9NTI5, P46063, Q9Y6D6, P18858, P51608, Q5W0B1, P21333, O95365, Q9NX00, Q9Y320, P16989, A2RRP1, Q08J23, Q6IQ22, Q9H6H4, O60832, Q9H6F5, O60784, Q14692, Q9UQ35, P21580, Q969V6, Q8IZL8, Q92619, O95456, Q9Y4C1, O75122, O00161, Q14676, Q8NFG4, Q96P48, Q8N1F8, Q9UBM7, Q49A26, Q8N3X1, Q15155, P18887, Q96BT3, P11831, O75592, Q9Y5Y0, P04637, Q07912, O14545, Q6NYC8, Q9Y314, Q8NF50, Q9BSQ5, Q9HB90, Q9H4L5, Q86VR2, O95295, Q15776, Q2TB10, Q8N3D4, O75396, Q9BXI6, Q9H211, Q15642, O75387, Q13233, P55198, Q76L83, Q15345, Q9UJZ1, Q7RTS1, Q13200, Q9GZR7, Q7Z6Z7, Q5T200, Q93008, O94979, O75376, Q969S3, P46013, Q8WUI4, Q15276, O14647, Q96MG7, Q13627, Q8IWY8, Q63HK3, Q9BRG2, Q7L7X3, Q9NSI8, Q9NRA8, Q8IY26, Q9Y3P8, Q96BY6, Q92785, Q15022, Q8N5C6, O94826, Q9C0C2, P46379, Q9BSF8, Q9UI10, Q9UIF9, Q96JN0, Q5VUA4, O94804, Q9BZ23, Q9Y485, O43182, P02545, Q9UHB6, Q7Z6E9, Q13614, Q9ULD9, Q9NSU2, O43665, O14646, Q13428, Q66PJ3, Q71RC2, Q8IWE5, Q8NFH5, Q8N1G2, P46821, Q66K14, Q63HN8, P29692, Q9H2Y7, O60927, Q9HC35, P20749, Q96GM8, Q14258, Q9Y520, Q13586, Q9Y2K6, Q8TB61, O75037, O00512, Q7L2J0, Q14493, P02765, P49761, Q5VZL5, P06748, P31751, Q7Z4V5, Q96RR4, Q147X3, Q9H116, Q5T7V8, Q9BPX3, Q9P289, Q86X95, Q5JSZ5, Q5VWN6, Q9H9J4, P08651, Q9UQ49, Q04726, Q00341, Q2VPK5, Q9UKM9, Q09666, Q9H1E3, Q9UPQ9, Q6ZRS2, P23396, Q5JRA6, O43237, Q9Y6G9, Q13105, Q58WW2, Q7L4I2, Q96JH7, Q9NWH9, Q96RL7, Q14157, P06241, Q6UN15, Q9UIG0, Q9Y6M7, Q14699, Q14498, P05023, Q8WU79, Q7Z2K8, Q96T58, Q5T3I0, P51957, Q9UG56, P21127, Q6P158, Q9NR82, Q76I76, Q9UBW7, Q8TAQ2, Q8NEY8, Q9ULL5, Q9BQG0, Q14005, P58004, Q9HCD5, Q9NZM3, O94762, Q9H8M2, Q15154, Q6WCQ1, Q7L1V2, Q9BRD0, Q96RS0, Q8NHG8, Q8IWS0, P49796, O43303, P52948, Q5UIP0, Q6WKZ4, P54198, Q9Y6X9, Q9BXL7, Q9HD20, Q6P2E9, Q9UGP8, P46108, Q9NQX3, Q9BWT3, Q5THK1, O15164, Q86WB0, Q9UIF8, Q9UPU7, P23497, Q3V6T2, Q9H4L7, Q13153, Q9H4A3, Q8N3F8, O00267, Q15185, Q9Y230, P61978, Q05519, Q7Z417, Q9BVJ6, O95466, Q9Y3T9, P26358, Q15545, Q0IIM8, P26038, Q3KQU3, Q5T011, Q9NUA8, Q9GZY6, P30281, O75674, Q86YV5, P15391, Q15596, Q12888, Q96KC8, Q13620, P78312, P54725, P05114, Q9Y2L9, Q9P2R6, O75643, Q86X53, Q13263, Q9NTJ3, Q99442, O00567, Q4G0J3, Q9Y2X3, Q92797, Q9BW71, P16615, O15400, Q5VT25, Q92609, Q9Y3X0, Q9BZQ8, P13796, Q15007, A6NC98, O75351, Q08170, Q86YC2, Q9BVS4, P85037, Q13098, Q5T8P6, Q9Y5W9, P12270, Q9ULJ3, Q8IXJ6, O60292, O75179, O60216, P08238, Q8WWM7, P30419, O00767, P29374, Q13610, Q9NRL2, Q15021, P26599, Q5JSH3, O95453, P53999, P18583, O43765, P25054, Q9UBW5, Q562F6, Q02446, Q8TCJ2, Q13043, Q12834, Q8TEQ6, A0AVK6, Q5T5X7, Q9UPN3, O95218, Q92974, Q9BWG6, Q9H0W8, Q9P260, Q92615, O95402, P08575, Q96LT9, Q6PKG0, Q9H3S1, Q8WYQ5, Q9Y2W1, Q96GY3, Q9NPI1, Q9UKL0, Q53GS9, Q9UMN6, Q9GZY8, Q14687, O75151, Q8TEH3, O95232, P07900, O43719, O14976, Q9ULU4, O15042, Q15057, Q9GZR1, Q9H307, Q6IBW4, O43148, Q15424, Q9UPS8, P80723, O15355, P21359, O75475, Q9UPN6, O43491, Q9UGN4, Q9H772, P46100, Q5THJ4, Q99729, O43166, P07814, Q5C9Z4, P05455, Q9UHX1, P28715, Q01085, Q8N108, Q9H6X4, Q96ST2, Q9BZI7, Q76FK4, Q8N3C0, Q96Q05, Q6IQ26, Q9H2U1, P62070, Q9Y383, Q03164, Q8TEW0, Q14C86, Q6ZS17, Q8WU90, Q9HAW4, Q8TDD1, Q969W3, Q8IY92, P16455, P35579, Q15435, Q13136, Q5SRE5, Q96EZ8, Q96RJ3, Q2M1Z3, P08567, Q8WVB6, O43395, P35611, P15056, Q4KMQ1, Q9C0B5, P42331, O75533, Q15477, Q9UQE7, Q9NSI2, Q8IY57, Q6PD62, Q9H330, Q7LBC6, P22626, Q9Y5S9, Q9NXC5, Q6VY07, O15056, Q92574, Q06587, P08621, Q07065, Q9UK61, Q8N1G0, O14497, P10321, Q29963, Q96JM3, Q14739, Q14195, Q16584, Q15003, P30414, Q8NAA4, Q5JTH9, Q00613, Q92622, Q8TB72, Q9Y5Q9, Q9Y6J0, Q9BQC3, P27448, Q13342, Q8NFC6, Q8NEM2, Q641Q2, O15173, O96028, P04075, P09972, Q06413, Q8IV63, O75821, P11912, Q7L8J4, Q5TZA2, Q7Z2Z1, Q9UKT9, P98175, Q99081, P51858, P49736, O00499, P51587, Q9BYV8, P48634, P35269, Q9Y2V2, Q3YEC7, Q6ZTN6, Q9UKJ3, Q8IY81, Q8N1G1, P46087, P43243, Q9P107, Q7RTP6, Q86VM9, O14920, Q9Y2D5, P49815, Q13242, Q6R327, Q9HCG8, Q15056, Q13459, Q9UN37, Q9H5I1, Q9H6A9, Q9H5J8, P36956, Q9BSJ6, Q12982, Q15742, Q96QE2, O00562, Q9H4L4, Q8WYP5, Q8NEL9, Q6F5E8, P10398, Q14684, Q12906, Q13112, Q9NUL3, O43516, Q969E4, Q8WUZ0, Q6NZY4, Q8WUB8, O75427, Q9P2G1, Q13535, P28066, Q9UKV3, Q12872, P22102, O15264, Q69YN4, P21554, P13051, Q5T4S7, Q9HC44, Q01082, P17987, Q8N4S0, Q9P2K3, Q9HB19, Q7Z4S6, Q6PH81, P52597, O95400, Q8N9Q2, P49354, P02794, P27105, Q12959, P13984, Q8WWQ0, P29590, Q9H1B7, Q9NPI6, Q66K74, P27816, O94874, Q9ULX3, Q92922, Q5JTD0, O60318, Q86UP2, O76021, Q68E01, Q15629, Q96ST3, Q9NR30, P23193, Q8WVC0, Q12789, Q9NZC9, O95104, O75909, P16333, Q9ULX6, A7E2V4, Q14573, O60930, Q13442, Q8WWN9, Q7Z569, Q6ULP2, Q09161, Q9Y5J1, Q9Y5T5, Q9H3N1, Q9UDY2, P19338, O60841, O00418, Q92766, Q92556, Q15773, Q08211, Q9NQS7, Q9NZN5, P14866, Q9H792, Q14103, Q9UHW9, O43432, Q32MZ4, Q5VT06, Q8TB03, Q16533, Q15831, Q8NI27, Q9HAN9, P62263, Q7Z5L9, Q9NR09, P61006, Q9BXB4, Q9Y2Z0, Q14004, P51532, Q12968, Q9C0C7, Q9UKS7, Q86W56, Q86X27, Q8N6T7, Q9P035, Q5VT52, Q9Y6X4, Q96T23, P34910, P12931, Q9UPT6, Q08999, Q9UNE7, P24941, Q9UDT6, Q96T37, P17152, P19634, O00178, Q53QZ3, O15037, P19878, Q8IZD2, Q16666, P33241, Q9H8G2, Q16181, Q9UGY1, P51531, O95772, Q52LR7, Q96QC0, P35251, P12956, Q9C0C9, O00193, Q9UM11, Q659C4, Q16512, O00472, P14859, Q5TB80, Q13111, Q53EP0, Q96CV9, P49792, Q96K21, Q86U90, O60934, Q56NI9, Q14247, O43149, P49841, Q00839, Q15061, P49674, Q5H9R7, Q9NS56, Q2KHR2, Q9UKI8, Q8N5Y2, Q06546, Q7Z3K3, Q9BRR9, Q9UHI6, Q16514, Q8TAD8, Q7Z3B3, Q8TEK3, P15822, O95251, Q8NEJ9, Q15366, Q9UPR0, Q8NG08, O75152, P07437, P50991, Q99698, Q7Z401, P14618, Q13641, Q8WXI9, P49916, O43583, Q9BXJ9, Q7Z5K2, O15014, O95391, Q9ULM3, P11717, Q8NHV4, O43896, Q15019, Q9ULV3, P52272, Q9BYW2, Q12873, Q9Y4W2, Q14847, P22059, Q13547, Q92930, O60885, Q13895, P37275, Q9H0D6, Q13303, Q13045, Q8N5G2, P07910, Q9NS91, Q8TC07, P15336, Q03188, P67809, Q9H6Z4, Q96GX5, P37802, Q6PJT7, Q9NU22, Q5VYK3, Q08379, Q92604, Q6PJG2, P42575, Q14694, Q6VMQ6, P06239, Q13033, P53621, P54252, Q9UBB4, Q9NUQ3, Q7Z3F1, Q6KC79, Q8N5F7, P20591, P09651, Q9NS87, Q86YS7, O75717, Q8N1I0, Q9Y608, O43399, Q9UPP1, O75554, Q9Y4F9, P51812, Q8N6T3, Q9Y6Y0, Q8IWI9, Q7Z460, Q9NYV4, Q15648, Q8TEB1, P11836, Q8TEQ0, O00505, Q15393, Q9H2X6, P54727, Q13501, O43566, Q5F1R6, Q8WUA7, Q96FS4, P53778, P18433, Q9C0H2, O94806, Q3MII6, O60341, Q9Y570, Q9NQA3, P13994, Q13206, Q8TBE7, P49848, Q9BTC0, Q96G46, P54259, P49756, Q8WUQ7, O15446, Q12830, O75400, Q96QE3, Q8IVH2, Q9UPA5, Q9Y3P9, Q9BRZ2, Q86YZ3, P16150, O00425, O00555, Q8NHZ8, Q14161, Q63ZY6, Q92576, Q9Y2K2, Q9H4Z3, Q96AY2, Q96EU6, P11940, Q86U42, P42167, P42166, Q9UBN7, Q9UKE5, Q13191, Q8NDX1, Q9UQR1, Q9NQW6, P05771, O15360, Q8TDB6, Q9BTA9, Q86V81, Q9UNL2, Q14644, P27987, P33991, Q8NDI1, Q96RU3, Q14160, O15169, Q14966, Q9H3D4, Q99728, P42694, Q8TBB5, Q9UHB9, O43684, Q9BVG4, O14492, Q9UH62, Q86YI8, Q8IXQ3, Q9NS68, P13612, Q9Y2H0, O75382, Q1KMD3, P36915, Q9NPF5, Q7Z3C6, Q9H7N4, Q14207, O15318, P51114, P50747, Q6ZN18, O43847, Q8IYB1, Q5TEJ8, Q5VYV7, Q15696, Q9BX95, Q9UK76, Q9H7L9, Q96I25, P40222, Q96Q45, Q9BQF6, Q9NUP7, Q8N6F7, O15085, Q86W92, Q13427, Q8N5H7, Q9H992, Q86V48, Q15311, Q8N8A6, Q9UGN5, Q96NY7, P22087, P54132, Q15014, Q8N2F6, Q9H7J1, Q14980, Q9Y4H4, Q14202, Q9UKV8, Q12912, Q8TEA8, Q99575, P27707, P29375, Q7Z2W4, Q9Y2Y9, Q96FJ0, Q8IZM8, Q9BY44, P38159, Q5J8M3, Q13131, O15234, O75153, P52701, P43007, Q9H270, Q9NSY1, P62316, Q9Y6K1, Q92625, P40818, O14578, Q86UE4, Q01813, O75494, Q27J81, Q92835, Q13144, Q04837, P38936, O43314, O00257, Q9BUJ2, Q13247, Q92804, Q15005, Q9Y618, O14686, Q53EL6, Q96EA4, P57737, Q92685, Q5VTR2, A0JNW5, Q5HYJ3, Q07666, Q96MU7, P17812, Q99459, P16949, O75665, Q8N2M8, Q8TF01, Q8NAP3, Q96GS4, Q86VQ1, B2RTY4, Q6DN90, Q9UJ70, Q9H410, P46019, Q02223, O43353, Q8NBR6, O43318, O95544, Q13185, Q8NG31, Q9H3Z4, O75420, Q13009, O95999, P31321, Q96Q15, P06744, O75534, Q5JVS0, P55265, Q9Y4E8, Q7Z2K6, P10075, Q9BTE7, Q92466, Q96FZ2, Q15036, C9JLW8, Q9H400, P33993, Q5FWF5, Q9UQR0, Q13129, Q92918, Q8WVT3, Q96CX2, O96018, Q96N67, Q15637, Q68EM7, Q53EZ4, Q9P275, P25098, Q96F86, Q96IQ9, Q9H981, Q9NUQ6, O60667, Q16186, Q9Y2X7, O43933, Q5VTL8, O95155, O95817, P23588, Q9BYG3, Q05D32, P17275, Q5JTV8, O94913, Q13523, Q9P1Y6, Q9NYF3, Q7Z3E2, Q7Z5R6, Q9UGP4, O00571, Q02543, Q9UIQ6, O95429, P51991, Q3KR37, Q13435, Q5T1M5, Q6ZW49, Q9BXK5, Q5SW79, O15226, O75081, Q96EP1, Q0VDF9, Q9BTU6, Q13283, Q07960, Q9NYL2, Q15287, P50552, Q8N684, Q92608, O75175, Q96K76, Q2TBE0, Q9P265, O60861, Q8WV28, O95394, Q6GQQ9, O14874, Q86TB9, Q99607, Q9H1A4, P04049, Q9NR55, Q53GL0, Q9UM82, Q9Y5K6, Q8N6S5, P42696, Q96CB8, P82094, Q9UKA4, Q01804, Q9BZE4, Q9UPZ3, Q8IWB9, P16220, O95747, P30622, Q6PGQ7, Q96P50, P40425, Q9H6Y2, Q6UB98, O75925, Q96PY6, P51116, P17544, Q96B36, Q9Y4D8, Q8NHM5, O43513, O15015, Q8NEZ4, O75132, Q9Y6N7, P09132, Q9Y572, Q9UBZ9, Q8N554, P06400, Q8ND30, Q9BSJ8, O14639, Q9NW82, O60674, P49757, Q96EV2, Q9NZN8, Q96T60, Q9Y6Q9, P28290, A1A4S6, Q9H4E7, Q14839, P15880, Q9UQL6, P78344, O43379, P35580, Q96MH2, Q5M775, P09086, Q96JC9, P51003, Q15652, P49585, P04920, Q86YV0, P49840, Q13439, Q99618, Q9P0U3, Q13029, O60293, O60343, P42356, Q13188, Q99640, P10809, P78362, Q6BDS2, O76094, O94915, O95674, P49321, Q8TD19, P41250, Q07955, Q6JBY9, O15258, O43823, Q12802, Q16625, Q07866, Q06210, Q96E39, Q9BU76, O14979, Q9NZ63, Q9NP87, Q6Y7W6, Q8N122, Q01831, Q9NV56, Q3L8U1, Q15365, Q8NI35, Q96RL1, Q9Y606, Q9BXS6, P41229, Q92945, Q9Y4F1, P04150, P46527, Q6N021, O60716, Q9BTE3, Q96J02, Q9Y2K1, O95793, Q9HB58, Q9Y3L3, Q02880, O95671, Q02040, Q8N573, P32519, Q9H5H4, Q6P5R6, Q04323, Q68DH5, Q15468, P24534, Q9H1Z4, Q01130, Q86TI0, P29350, Q9P2N5, P19525, Q9UJU6, Q9NX63, Q92614, P24928, Q8IXT5, Q9UH99
The following 826 proteins are only supported by 1 or 2 peptide spectra,
hence, protein quantification is not reliable and can only be calculated
by the 'mean' in these cases, corresponding protein accessions are:
  Q96A47, Q1KMD3, Q9H4A6, Q9H7N4, Q86U86, O75367, Q9BUU2, Q04637, Q8IYB3, Q5TCZ1, O95819, Q92538, Q92766, P49768, Q9UFC0, P49327, P06576, Q96C19, O15027, Q99704, Q9NW97, Q9P1Y6, P29966, Q15651, Q9NU22, Q6WCQ1, Q9NYF8, Q9NZB2, Q9P2E9, Q9NTI5, Q14566, P18858, P51608, P13747, P38432, A2RRP1, Q08J23, P13639, P20700, O60784, P21580, Q6ZRS2, Q9Y4C1, Q9Y6X4, Q8NFG4, Q8N684, Q8N1F8, P24588, Q14247, Q49A26, Q8N3X1, Q9NYJ8, Q96GM8, P51114, Q9Y5Y0, Q9BSQ5, Q9HB90, Q6KC79, Q9H6F5, Q14699, Q8N3D4, Q5TAQ9, P13051, Q9Y2U8, O94964, Q13200, O94979, P46013, Q9NYM9, Q8WUI4, Q15276, Q14669, Q13627, Q13523, Q86X29, Q14258, P27708, Q7L7X3, Q9NSI8, Q92618, Q6ZSS7, Q6NXT1, O00555, Q96BY6, Q8TEP8, Q7KZI7, Q12778, Q15022, Q14677, P46379, P41273, Q8N5I9, Q9HC35, Q9UIF9, Q5VUA4, Q9UQC2, O43182, P02545, Q16513, Q9UHB6, Q9NPI6, O14646, Q14005, Q13428, Q8N1G2, Q66K14, P29692, Q9H2Y7, P33991, Q9BX63, P16104, P50552, Q7Z4H7, Q66K74, Q8TB61, Q9Y2W1, Q96HI0, Q96HB5, O75909, Q92900, P02765, Q7Z4V5, Q9HBH9, Q13077, Q9P289, Q86X95, Q5VWN6, P53621, Q9UPP1, Q9BTA9, Q9BVG9, Q04726, O14981, Q00341, Q09666, Q5JRA6, Q9UPN3, Q96KC8, Q9Y6G9, Q7L4I2, Q9H4Z2, Q9NWH9, P06241, P38159, Q8NI27, Q9UIG0, Q86VQ1, Q8N488, Q9Y6M7, Q92576, Q8TEM1, Q8WU79, Q96T58, P51957, Q86W92, P21127, Q9BW71, P41227, Q76I76, Q96EY5, Q9BUJ2, Q96JM3, Q9HB58, Q9ULL5, P58004, Q9HCD5, Q9NZM3, Q9H8M2, Q15154, Q14676, Q9BRD0, Q9H9J4, Q96D46, P51532, O43303, P52948, Q53EL6, Q9UKV3, Q9NQX3, O15164, Q8WW12, Q86WB0, Q9UPU7, Q8NEY8, P23497, Q13153, Q9H4A3, Q05519, P55196, Q01201, Q9Y3T9, P55010, Q15545, O14523, Q3KQU3, Q9Y4C8, P17029, Q86YV5, Q96JZ2, Q15596, Q9Y5B6, P78312, P54725, Q86UE4, P34932, Q12789, Q9UKA4, Q96NY7, Q9NTJ3, Q9Y2X3, Q9NR30, O00567, P36915, P11836, Q9Y3X0, P49006, Q96EI5, Q15007, Q08170, Q13098, Q5T8P6, Q9Y5W9, Q9NZJ5, Q15788, O75179, Q96C57, Q13610, P29375, P49750, Q8WYB5, Q5UIP0, Q9BQG0, Q96B01, P28715, P18583, Q9UGU0, Q05655, Q8N264, Q02446, Q12834, Q9UN37, Q8TEQ6, Q9Y2L9, Q5T5X7, O95218, Q92974, P42858, Q9H0W8, Q9P260, P54105, O95402, Q15652, P55081, Q6PKG0, Q15643, Q8NCN4, P08651, Q9Y388, O95232, O00418, P35659, O60832, O15042, Q8NEZ4, Q15057, Q01804, Q96RU3, Q15424, Q96G01, Q13557, Q5T6F2, P80723, Q9Y6I9, P19634, O75475, Q9UPN6, P19338, O60292, P07814, Q8N4S9, Q5JSH3, Q5H9R7, Q5C9Z4, Q9H6X4, O94804, Q969V6, Q9H2U1, P62070, Q9NYZ3, O75962, Q969W3, Q8IZL8, Q15435, Q9Y4E8, P17861, Q8WVB6, P35611, Q5JTH9, P42331, O75533, Q6PD62, Q9UQ35, Q6WKZ4, Q96RK0, Q01082, P22626, P21291, Q9Y5S9, Q14188, Q14814, Q9NXC5, P20290, Q92574, P08621, Q07065, P30419, Q9UK61, P10321, O43432, P01893, Q00613, P07948, Q9BQC3, P27448, O15534, Q8NFC6, P09972, Q96HA1, Q63HN8, P49736, Q7Z6Z7, O00499, Q9BYV8, Q96ST2, Q96MG7, Q15942, Q9P107, O14497, Q86VM9, O14920, P49815, Q86VR2, P54259, Q6R327, P48651, Q8NEL9, O43491, Q9Y6D5, Q13112, Q99661, Q9P275, Q8IV04, O43516, Q8WUZ0, Q6NZY4, P52594, Q8WUB8, Q9NPR2, Q9BZF1, Q69YN4, Q15648, O95671, Q9Y546, Q9P2K8, Q9NWW5, P17987, Q9UPT8, Q8N4C6, Q9C0C2, Q9NRN7, Q96KQ7, Q8IYL3, O95400, P49354, Q16539, Q9H7U1, P13984, Q8WWQ0, O14647, Q02880, P29590, Q9H1B7, Q9H1E3, Q15910, Q9UI08, Q9C0C9, Q5SSJ5, P51858, Q96ST3, P23193, P17096, Q8WVC0, P16070, Q9Y2K7, A7E2V4, O60841, P48634, Q13442, Q13185, Q92993, Q6P6C2, O95071, Q5T200, Q9NZT2, Q92733, Q92556, Q08211, P14866, Q9H792, Q14103, Q12888, Q9UBQ5, Q32MZ4, Q16204, O14545, O60293, P35269, Q7Z5L9, Q9NR09, P61006, Q12968, Q9C0C7, Q12774, Q9H7P9, Q96T23, P34910, Q08999, Q8TEU7, Q5SW79, Q9Y4B6, Q9C086, Q09472, P08238, Q8IZP0, Q53QZ3, O15037, O60231, P51531, Q8IU68, O75448, Q01105, Q659C4, Q16512, O00472, P14859, P36507, Q9GZR1, Q01581, Q9Y3Q8, Q13404, Q9Y520, O43149, P49841, C9JI98, P49674, Q13574, Q2KHR2, Q9UKI8, Q06546, Q7Z3K3, Q13905, Q9UHI6, Q6NYC8, Q8NEJ9, Q15366, Q9UPR0, Q8N4S0, Q8NE71, P07437, Q13586, P21796, Q8WXI9, P49916, Q7Z5K2, O60333, O95391, Q9ULM3, Q8NHV4, Q9BYW2, Q9Y4W2, P22059, Q9BWU0, Q92930, O43818, P37275, Q9UBB9, Q9Y2H6, Q99543, Q8N5G2, Q66PJ3, Q5VYV7, P07910, Q96HR8, Q8TC07, P15336, Q03188, Q92922, Q86YC2, Q9P2D1, Q6PJT7, Q9BX95, Q6PJG2, Q9NY27, O95466, P06239, Q5U623, Q9Y5T5, Q7Z417, Q5T1M5, Q8N5F7, Q8NF50, Q86YS7, P08575, Q9Y608, Q6ZU80, Q9H0D6, Q9P2R6, P50750, Q7Z460, P20020, O75592, Q9NSU2, Q8TEQ0, Q9H2P0, O00505, Q16625, P52292, P67809, Q8NB46, Q96L91, Q01518, Q96QC0, P53778, Q9Y4G8, Q9H147, P18433, Q9C0H2, Q9NVM6, Q9ULV3, P33241, Q9Y570, Q96T37, P13994, O43166, Q99590, Q86X53, Q8TB36, Q96G46, Q8N6T7, Q8WUQ7, Q96QE3, Q9Y3P9, Q8WWM7, Q8TDD1, P27824, Q63ZY6, Q96AY2, P27815, P42166, Q9UBN7, Q9UKE5, Q13191, O14976, O75362, Q13107, Q5T5C0, Q86UP2, P05771, Q9UMN6, P10398, Q969T4, P46100, O43572, Q14966, O14686, Q8IYH5, P42694, Q9Y2D5, P32249, Q6ZSZ5, Q7Z628, Q9BZ23, Q9Y6J0, Q9NRG0, P16383, Q9NZ63, Q9H307, Q6UN15, Q7Z333, Q9H7D7, Q6ZNJ1, Q99698, Q15696, Q9UK76, Q9BU76, Q9H7L9, Q8TAD8, Q96I25, Q8IYB7, P57682, Q12906, Q96Q45, Q09161, Q6JBY9, P49796, Q92608, Q86TB9, Q13459, Q8TEH3, Q8N8A6, Q9NV88, Q14157, Q86X27, Q15014, Q5VT06, Q9H6Z4, O00178, Q9NRL2, P35658, Q8ND76, Q14739, Q14004, O14745, Q8N111, Q13620, Q6GYQ0, Q2M2I8, Q96JG6, P27707, Q7Z2W4, Q96FJ0, Q9NS87, Q9BY44, Q13247, Q13131, Q99442, Q8TBB5, O75153, Q5JTV8, Q9Y2R2, Q9BVJ6, Q14207, Q5VZK9, Q12986, P40818, O14578, Q9NP64, Q9BXB4, Q13263, Q96A65, P38936, O00257, Q9UPN7, Q96FS4, Q96MU7, P17812, O14974, Q86V48, Q8NDX1, O60885, Q8NAP3, Q13563, P12931, P35236, Q6DN90, O43566, Q9H410, Q14191, Q13111, Q13813, Q96N67, Q2M1Z3, Q13206, Q9UPN4, Q147X3, Q9BRL6, Q96PY6, P18754, Q5FWF5, Q13129, Q96CX2, Q92615, Q8ND56, Q6VN20, O75122, O95251, Q9Y2X7, O43933, Q5VTL8, O95155, Q4G0J3, Q9P2T1, Q8WWI1, P23588, Q9ULW0, Q9GZU8, P51991, O95503, Q68EM7, Q13435, Q8N7B6, P32248, Q6ZW49, Q8N201, P49023, Q96BY7, O75131, O15226, O75081, Q5SQI0, Q07960, O75427, Q8WYL5, Q9UPQ9, Q2PPJ7, Q15149, O75175, Q9H8G2, Q8N3S3, Q96K76, Q15032, Q9NVR2, Q99700, Q6P995, Q9BXL7, O15234, Q53GL0, Q9Y5K6, P42696, Q96CB8, P00492, P43243, Q99685, Q01433, P17544, Q16666, P51809, O43513, Q9ULJ3, Q9Y5P4, Q15637, O60669, Q9H089, Q9UDT6, Q9Y285, P05455, P18887, Q8IY57, Q9H501, Q9BPX3, O15400, Q8ND30, Q8TAQ2, Q05209, Q9H4E7, P15880, P11388, P78344, O43186, Q9UKM9, Q9ULT8, Q6ULP2, Q96CV9, Q9NRY4, Q5M775, Q8IWC1, Q13469, P49585, P04920, Q86YV0, Q9NYA4, Q13439, Q99618, O60934, Q99640, Q16555, Q9BTU6, Q9HD15, Q8TD19, Q96LR5, O95674, Q07955, O43823, Q96E39, O43719, Q13310, P10809, O94761, P52701, Q13427, Q9UBH6, O14737, P11532, Q9UGN5, Q8WZ73, Q96RL1, P50851, Q9UKY7, P40222, O60566, P04150, Q76FK4, Q9BYG3, Q9UJX2, O95793, Q5JSP0, Q5VZL5, Q2LD37, Q6AI39, Q02040, Q8IXM2, Q96C90, P32519, Q9UN86, Q9NWZ5, Q6P5R6, Q68DH5, P49792, Q15361, Q9GZY6, Q15021, Q9NX63, Q92614, P24928, Q9HCG8, P42680
'select()' returned 1:many mapping between keys and columns
'select()' returned 1:many mapping between keys and columns
'select()' returned 1:many mapping between keys and columns
```

In [12]:

```
head(merged.exp.complete)
```

|  | P1 MOCK TAB | P1 MCM TAB | P2 MOCK TAB | P2 MCM TAB | P3 MOCK TAB | P3 MCM TAB | P4 MOCK TAB | P4 MCM TAB | all MOCK TAB | all MCM TAB | P1 MOCK PBMCB | P1 MCM PBMCB | P2 MOCK PBMCB | P2 MCM PBMCB | P3 MOCK PBMCB | P3 MCM PBMCB | P4 MOCK PBMCB | P4 MCM PBMCB | all MOCK PBMCB | all MCM PBMCB |
| --- | --- | --- | --- | --- | --- | --- | --- | --- | --- | --- | --- | --- | --- | --- | --- | --- | --- | --- | --- | --- |
| AAAAGLGHPASPGGSEDGPPGSEEEDAAR;MOD:00696:11 | -3.189996 | -3.310649 | -3.291647 | -3.247577 | -3.373987 | -3.389355 | -3.440618 | -3.386714 | -3.354630 | -3.253330 | -3.255346 | -3.302503 | -3.394234 | -3.328847 | -3.383582 | -3.245505 | -3.289506 | -3.339847 | -3.424943 | -3.266948 |
| AAAAGLGHPASPGGSEDGPPGSEEEDAAR;MOD:00696:11,MOD:00696:15 | -3.333761 | -3.236725 | -3.260791 | -3.199894 | -3.405461 | -3.296208 | -3.498287 | -3.421945 | -3.299274 | -3.292990 | -3.247255 | -3.226669 | -3.392989 | -3.370331 | -3.432972 | -3.402722 | -3.332143 | -3.221197 | -3.316559 | -3.294446 |
| AAAAGLGHPASPGGSEDGPPGSEEEDAAR;MOD:00696:11,MOD:00696:15,MOD:00696:22 | -3.384471 | -3.308603 | -3.282938 | -3.277347 | -3.312964 | -3.275861 | -3.404325 | -3.358213 | -3.331447 | -3.289666 | -3.218630 | -3.197564 | -3.414800 | -3.422803 | -3.378703 | -3.357616 | -3.287456 | -3.394653 | -3.303287 | -3.264483 |
| AAAGSPRSPGPLPGAR;MOD:00696:5,MOD:00696:8 | -3.598677 | -3.515455 | -2.741501 | -2.773794 | -4.234705 | -4.193610 | -3.207020 | -2.841275 | -3.335149 | -3.651504 | -3.126377 | -3.014671 | -3.605889 | -3.438229 | -3.587995 | -3.211325 | -3.417647 | -3.502627 | -3.314216 | -3.134290 |
| AAATPESQEPQAK;MOD:00696:7 | -3.263208 | -3.172013 | -3.674968 | -3.733727 | -3.051952 | -2.814715 | -3.452647 | -3.479216 | -3.556972 | -3.282788 | -3.406341 | -3.079812 | -3.537622 | -3.234091 | -3.655277 | -3.488505 | -3.323115 | -3.013821 | -3.445493 | -3.170881 |
| AAEEQGDDQDSEK;MOD:00696:11 | -3.563011 | -3.093479 | -3.479177 | -2.531309 | -3.780714 | -3.466817 | -3.613729 | -3.200595 | -3.635946 | -3.300877 | -3.677561 | -3.076639 | -3.541433 | -3.311868 | -3.595263 | -3.110334 | -3.382357 | -3.139196 | -3.514140 | -3.043776 |

# Differential expression analysis¶

## RNASeq¶

In [13]:

```
# create the design
rnaseq.design <- model.matrix(~ rnaseq.dge$samples$treatment + rnaseq.dge$samples$patient + rnaseq.dge$samples$cell_type)
colnames(rnaseq.design)[2] <- "treatment"

# re-compute library size
rnaseq.dge$samples$lib.size <- colSums(rnaseq.dge$counts)

# normalize
rnaseq.dge <- calcNormFactors(rnaseq.dge, "TMM")

# estimate dispersion
rnaseq.dge <- estimateDisp(rnaseq.dge, rnaseq.design)

# fit and get the DE genes for treatment
rnaseq.fit <- glmFit(rnaseq.dge, rnaseq.design)
rnaseq.treatment <- glmLRT(rnaseq.fit, coef = "treatment")

rnaseq.sig <- topTags(rnaseq.treatment, n=Inf, p = 0.05)$table
rnaseq.all <- topTags(rnaseq.treatment, n = Inf, p = 1)$table
```

20 top regulated genes:

In [14]:

```
rnaseq.sig[1:20,]
```

|  | gene | uniprot | name | entrez | logFC | logCPM | LR | PValue | FDR |
| --- | --- | --- | --- | --- | --- | --- | --- | --- | --- |
| CYB5R2 | CYB5R2 | A8K237 | cytochrome b5 reductase 2 | 51700 | 1.2373258 | 5.2270899 | 97.68605 | 4.902630e-23 | 7.209317e-19 |
| TVP23A | TVP23A | A6NH52 | trans-golgi network vesicle protein 23 homolog A | 780776 | 1.8455671 | 3.0975751 | 95.19845 | 1.722247e-22 | 1.266282e-18 |
| ACKR3 | ACKR3 | P25106 | atypical chemokine receptor 3 | 57007 | 2.7148705 | 5.3166526 | 92.85795 | 5.618569e-22 | 2.754035e-18 |
| CD22 | CD22 | P20273 | CD22 molecule | 933 | 1.2183852 | 9.6092232 | 86.91514 | 1.132787e-20 | 4.164407e-17 |
| NFKB2 | NFKB2 | Q00653 | nuclear factor kappa B subunit 2 | 4791 | 1.1095606 | 8.2577186 | 84.96313 | 3.039809e-20 | 8.940080e-17 |
| SPTY2D1 | SPTY2D1 | Q68D10 | SPT2 chromatin protein domain containing 1 | 144108 | -0.8573408 | 6.0412507 | 84.46195 | 3.916800e-20 | 9.599423e-17 |
| POLE3 | POLE3 | A0A024R829 | DNA polymerase epsilon 3, accessory subunit | 54107 | -1.0307820 | 6.7231878 | 83.81788 | 5.425200e-20 | 1.139680e-16 |
| ZG16B | ZG16B | G8H6I3 | zymogen granule protein 16B | 124220 | 2.5764240 | 1.7560417 | 83.08916 | 7.843469e-20 | 1.441728e-16 |
| CHAC2 | CHAC2 | Q8WUX2 | ChaC cation transport regulator homolog 2 | 494143 | -1.0776733 | 3.7677900 | 82.17085 | 1.248174e-19 | 2.039377e-16 |
| NFKBIE | NFKBIE | A0A024RD24 | NFKB inhibitor epsilon | 4794 | 1.0057043 | 6.5207275 | 81.91282 | 1.422235e-19 | 2.091396e-16 |
| CAMK1D | CAMK1D | Q5SQQ7 | calcium/calmodulin dependent protein kinase ID | 57118 | 0.8194934 | 6.5078298 | 81.70933 | 1.576472e-19 | 2.107456e-16 |
| TRIP13 | TRIP13 | Q15645 | thyroid hormone receptor interactor 13 | 9319 | -1.2506421 | 5.4770033 | 80.99940 | 2.257862e-19 | 2.766821e-16 |
| CDC6 | CDC6 | A0A024R1S2 | cell division cycle 6 | 990 | -1.1771185 | 5.9852642 | 80.57745 | 2.795311e-19 | 3.161927e-16 |
| SELENOI | SELENOI | Q9C0D9 | selenoprotein I | 85465 | -0.6920195 | 6.3338883 | 76.75096 | 1.939338e-18 | 1.947783e-15 |
| CTSB | CTSB | Q5HYG5 | cathepsin B | 1508 | 1.0827821 | 6.2309038 | 76.70315 | 1.986858e-18 | 1.947783e-15 |
| DUSP22 | DUSP22 | Q9NRW4 | dual specificity phosphatase 22 | 56940 | 0.7995448 | 7.5107511 | 76.01360 | 2.817182e-18 | 2.589167e-15 |
| ZWINT | ZWINT | O95229 | ZW10 interacting kinetochore protein | 11130 | -1.2132295 | 6.0926681 | 74.93171 | 4.872804e-18 | 4.214975e-15 |
| FBXO5 | FBXO5 | Q9UKT4 | F-box protein 5 | 26271 | -1.0819130 | 5.5068664 | 74.22197 | 6.980889e-18 | 5.436747e-15 |
| MCM10 | MCM10 | Q7L590 | minichromosome maintenance 10 replication initiation factor | 55388 | -1.1811441 | 5.4562528 | 74.20962 | 7.024698e-18 | 5.436747e-15 |
| SHE | SHE | Q5VZ18 | Src homology 2 domain containing E | 126669 | 2.9585513 | 0.7710592 | 72.80277 | 1.432755e-17 | 1.053433e-14 |

## Proteomics¶

Differential expression analysis for the proteomics data is performed using `limma`.

Normalization was already performed prior to loading the data.

In [15]:

```
# create the experimental design
prot.design <- model.matrix(~ 0 + proteomics.el$samples$condition +
                         proteomics.el$samples$cell.type +
                         proteomics.el$samples$patient.id)

colnames(prot.design) <- gsub("[^A-Z]*([A-Z])", "\\1", colnames(prot.design))

contrast.matrix<-makeContrasts(contrasts="MCM-MOCK", levels=prot.design)

# fit a linear model and find differentially expressed proteins
prot.fit <- lmFit(proteomics.el, prot.design)
prot.contr <- contrasts.fit(prot.fit, contrast.matrix)
prot.bayes <- eBayes(prot.contr)

prot.sig <- topTable(prot.bayes, p = 0.05, n = Inf)
prot.all <- topTable(prot.bayes, p = 1, n = Inf)
```

Top 20 regulated proteins:

In [16]:

```
prot.sig[1:20,]
```

|  | uniprot | RefSeqId | Gene | Name | logFC | AveExpr | t | P.Value | adj.P.Val | B |
| --- | --- | --- | --- | --- | --- | --- | --- | --- | --- | --- |
| HIC1 | Q14526 | 3090 | HIC1 | HIC ZBTB transcriptional repressor 1 | 0.4937650 | -3.346909 | 14.437652 | 1.497320e-10 | 9.581350e-07 | 13.946874 |
| PACS1 | Q6VY07 | 55690 | PACS1 | phosphofurin acidic cluster sorting protein 1 | 0.2981411 | -3.330347 | 13.319758 | 4.911532e-10 | 1.571445e-06 | 12.935973 |
| SERPINE2 | P07093 | 5270 | SERPINE2 | serpin family E member 2 | 1.7950301 | -3.648968 | 12.320355 | 1.529058e-09 | 3.261480e-06 | 11.943993 |
| LY75 | O60449 | 4065 | LY75 | lymphocyte antigen 75 | 0.4610657 | -3.349367 | 10.511011 | 1.468121e-08 | 2.335192e-05 | 9.903659 |
| SRGN | P10124 | 5552 | SRGN | serglycin | 1.0758634 | -3.436961 | 10.348736 | 1.824654e-08 | 2.335192e-05 | 9.703552 |
| CASP7 | P55210 | 840 | CASP7 | caspase 7 | 0.5018522 | -3.347932 | 9.507717 | 5.876056e-08 | 6.266814e-05 | 8.616646 |
| BUB1 | O43683 | 699 | BUB1 | BUB1 mitotic checkpoint serine/threonine kinase | -0.4754083 | -3.345551 | -9.356517 | 7.308819e-08 | 6.681305e-05 | 8.412030 |
| ENDOD1 | O94919 | 23052 | ENDOD1 | endonuclease domain containing 1 | 0.4510115 | -3.348848 | 8.919236 | 1.393618e-07 | 1.021383e-04 | 7.803708 |
| LZTFL1 | Q9NQ48 | 54585 | LZTFL1 | leucine zipper transcription factor like 1 | 0.7954250 | -3.401921 | 8.818107 | 1.623005e-07 | 1.021383e-04 | 7.659445 |
| TGFBI | Q15582 | 7045 | TGFBI | transforming growth factor beta induced | 1.9031343 | -3.775602 | 8.806177 | 1.652574e-07 | 1.021383e-04 | 7.642336 |
| KPNA2 | P52292 | 3838 | KPNA2 | karyopherin subunit alpha 2 | -0.7477266 | -3.382343 | -8.766230 | 1.755776e-07 | 1.021383e-04 | 7.584909 |
| EDRF1 | Q3B7T1 | 26098 | EDRF1 | erythroid differentiation regulatory factor 1 | -0.4939674 | -3.347583 | -8.705489 | 1.925873e-07 | 1.026972e-04 | 7.497177 |
| YTHDF2 | Q9Y5A9 | 51441 | YTHDF2 | YTH N6-methyladenosine RNA binding protein 2 | -0.2504660 | -3.328601 | -8.550070 | 2.444830e-07 | 1.138037e-04 | 7.270418 |
| TOP2B | Q02880 | 7155 | TOP2B | DNA topoisomerase II beta | 0.3141782 | -3.332768 | 8.538262 | 2.489846e-07 | 1.138037e-04 | 7.253056 |
| SACM1L | Q9NTJ5 | 22908 | SACM1L | SAC1 like phosphatidylinositide phosphatase | 0.2662144 | -3.329345 | 8.480249 | 2.724012e-07 | 1.162064e-04 | 7.167474 |
| SLC25A1 | P53007 | 6576 | SLC25A1 | solute carrier family 25 member 1 | 0.2624973 | -3.331345 | 8.302766 | 3.595349e-07 | 1.437915e-04 | 6.902754 |
| TNC | P24821 | 3371 | TNC | tenascin C | 1.3742473 | -3.552465 | 8.234100 | 4.007041e-07 | 1.508297e-04 | 6.799159 |
| FLNB | O75369 | 2317 | FLNB | filamin B | -0.4166404 | -3.348002 | -8.176343 | 4.391608e-07 | 1.561217e-04 | 6.711509 |
| FLNA | P21333 | 2316 | FLNA | filamin A | -1.1360368 | -3.485893 | -8.091970 | 5.024447e-07 | 1.692181e-04 | 6.582622 |
| CDC20 | Q12834 | 991 | CDC20 | cell division cycle 20 | -0.5958105 | -3.362719 | -7.979627 | 6.019199e-07 | 1.848159e-04 | 6.409436 |

## Phospho-proteomics¶

In [17]:

```
phospho.el <- readRDS("cache/mcm_induction_proteomics_phospho.rds")

# ---- run limma ----
phospho.el$samples$condition <- relevel(factor(phospho.el$samples$condition), "MOCK")
design <- model.matrix(~ 0 + condition + patient.id + cell.type, data = phospho.el$samples)
colnames(design) <- gsub("^[^A-Z]*", "", colnames(design))


contrast.matrix<-makeContrasts(contrasts="MCM-MOCK", levels=design)

# fit a linear model and find differentially expressed proteins
phospho.fit <- lmFit(phospho.el, design)
phospho.contr <- contrasts.fit(phospho.fit, contrast.matrix)
phospho.bayes <- eBayes(phospho.contr)

phospho.sig <- topTable(phospho.bayes, n = Inf, p.value = 0.05)
```

In [18]:

```
head(phospho.sig)
```

|  | uniprot | RefSeqId | Gene | Name | logFC | AveExpr | t | P.Value | adj.P.Val | B |
| --- | --- | --- | --- | --- | --- | --- | --- | --- | --- | --- |
| ENQPENSQTPTKK;MOD:00696:11 | Q12834 | 991 | CDC20 | cell division cycle 20 | -1.3250365 | -3.510316 | -11.120513 | 5.513921e-09 | 9.908516e-06 | 10.885842 |
| LYNLSGNGSPLADSK;MOD:00400:7,MOD:00696:9 | Q53HL2 | 55143 | CDCA8 | cell division cycle associated 8 | -0.8543886 | -3.396912 | -10.085727 | 2.214741e-08 | 1.757622e-05 | 9.580331 |
| STEDLSPQK;MOD:00696:6 | Q96RT1 | 55914 | ERBIN | erbb2 interacting protein | -0.6453918 | -3.363111 | -9.885521 | 2.934260e-08 | 1.757622e-05 | 9.313590 |
| GGDAAVSPGGPPLGLAPPPR;MOD:00696:7 | Q14526 | 3090 | HIC1 | HIC ZBTB transcriptional repressor 1 | 0.9774377 | -3.420111 | 9.326814 | 6.580436e-08 | 2.849769e-05 | 8.543423 |
| ENQPENSQTPTKK;MOD:00696:9 | Q12834 | 991 | CDC20 | cell division cycle 20 | -0.9007158 | -3.427691 | -9.152651 | 8.524108e-08 | 2.849769e-05 | 8.295344 |
| SLGLSLSGGDQEDAGR;MOD:00696:7 | Q9UJ70 | 55577 | NAGK | N-acetylglucosamine kinase | 0.5364677 | -3.351057 | 9.079331 | 9.515087e-08 | 2.849769e-05 | 8.189740 |

Highlight proteins that were not found differentially expressed in the global proteomics and RNA-seq data but did show significantly different expression in their phosphorylated states:

In [19]:

```
phospho.sig[phospho.sig$Gene %in% c("PRKCB", "NKAP"), ]
```

|  | uniprot | RefSeqId | Gene | Name | logFC | AveExpr | t | P.Value | adj.P.Val | B |
| --- | --- | --- | --- | --- | --- | --- | --- | --- | --- | --- |
| NPEPDSDEHTPVEDEEPK;MOD:00696:6,MOD:00696:10 | Q8N5F7 | 79576 | NKAP | NFKB activating protein | 0.3665158 | -3.338488 | 6.101252 | 1.464369e-05 | 0.0004385786 | 3.2692745 |
| QPVELTPTDK;MOD:00696:8 | P05771 | 5579 | PRKCB | protein kinase C beta | 0.5704816 | -3.415820 | 3.986434 | 1.042988e-03 | 0.0075574571 | -0.9413995 |
| QPVELTPTDK;MOD:00696:6 | P05771 | 5579 | PRKCB | protein kinase C beta | 0.3928942 | -3.380943 | 3.160558 | 5.998992e-03 | 0.0259139160 | -2.6410029 |

## Repeat for difference between TAB and PBMCB¶

In support for the PCA, test for differentially expressed proteins between TAB and PBMCB

In [20]:

```
celltypes.prot.bayes <- eBayes(prot.fit)
celltypes.prot.sig <- topTable(celltypes.prot.bayes, coef = "TAB", p = 0.05, n = Inf)
nrow(celltypes.prot.sig)
```

0

# Compare RNASeq and Proteomics¶

In [21]:

```
all.prot <- unique(c(prot.sig$Gene, rnaseq.sig$gene))
all.prot <- all.prot[!is.na(all.prot)]
prot.found <- data.frame(
    row.names = all.prot,
    Proteomics = all.prot %in% prot.sig$Gene,
    RNASeq = all.prot %in% rnaseq.sig$gene
)

setPlotSize(5,5)
vennDiagram(vennCounts(prot.found))

shared.prot <- merge(prot.sig, rnaseq.sig, by.x = "Gene", by.y = "gene", all.x = F, all.y = F, suffixes = c(".prot", ".rna"))

cor.result <- cor.test(shared.prot$logFC.prot, shared.prot$logFC.rna, method = "spearman")

createPlots <- T
if (createPlots) {
    svg("plots/Supplementary_Figure_3B-prot_rna_corr.svg", width=5, height=5)
}
plot(shared.prot$logFC.rna, shared.prot$logFC.prot,
    xlim = c(-4, 4),
    ylim = c(-1.2, 1.2),
    xlab = "RNASeq (log-fold change)",
    ylab = "Proteomics (log-fold change)")
text(-4, 1, paste0("Spearman rho = ", round(cor.result[["estimate"]], 2), " (p = ", round(cor.result[["p.value"]], 4), ")"), pos=4)
abline(v = 0, col = "grey30", lty = 3)
abline(h = 0, col = "grey30", lty = 3)

dev.off()

resetPlotSize()
```

**png:** 2

In [22]:

```
rnaseq.all["MS4A1",]
prot.all[prot.all$Gene == "MS4A1" & !is.na(prot.all$Gene),]
```

|  | gene | uniprot | name | entrez | logFC | logCPM | LR | PValue | FDR |
| --- | --- | --- | --- | --- | --- | --- | --- | --- | --- |
| MS4A1 | MS4A1 | A0A024R507 | membrane spanning 4-domains A1 | 931 | -0.8142047 | 9.851281 | 5.636131 | 0.01759399 | 0.05145577 |

|  | uniprot | RefSeqId | Gene | Name | logFC | AveExpr | t | P.Value | adj.P.Val | B |
| --- | --- | --- | --- | --- | --- | --- | --- | --- | --- | --- |
| MS4A1 | P11836 | 931 | MS4A1 | membrane spanning 4-domains A1 | -0.3299744 | -3.366323 | -2.462929 | 0.02559595 | 0.08419436 | -3.925702 |

In [23]:

```
test_gene <- "PAX5"
rnaseq.all[test_gene,]
prot.all[prot.all$Gene == test_gene & !is.na(prot.all$Gene),]
```

|  | gene | uniprot | name | entrez | logFC | logCPM | LR | PValue | FDR |
| --- | --- | --- | --- | --- | --- | --- | --- | --- | --- |
| PAX5 | PAX5 | Q02548 | paired box 5 | 5079 | 0.4753438 | 8.940964 | 15.06654 | 0.000103787 | 0.0007911809 |

|  | uniprot | RefSeqId | Gene | Name | logFC | AveExpr | t | P.Value | adj.P.Val | B |
| --- | --- | --- | --- | --- | --- | --- | --- | --- | --- | --- |
| PAX5 | Q02548 | 5079 | PAX5 | paired box 5 | 0.1409227 | -3.328703 | 2.440358 | 0.02677647 | 0.08688775 | -3.967312 |

In [24]:

```
# CD69, CD80, CD30 (TNFRSF8), and CD137 (4-1BB/TNFRSF9)
nfkb_genes <- c("CD69", "CD80", "TNFRSF8", "TNFRSF9", "CD30", "TNFAIP2")
shared.prot[shared.prot$Gene %in% nfkb_genes, ]

rnaseq.sig[nfkb_genes, ]
prot.sig[prot.sig$Gene %in% nfkb_genes, ]
```

|  | Gene | uniprot.prot | RefSeqId | Name | logFC.prot | AveExpr | t | P.Value | adj.P.Val | B | uniprot.rna | name | entrez | logFC.rna | logCPM | LR | PValue | FDR |
| --- | --- | --- | --- | --- | --- | --- | --- | --- | --- | --- | --- | --- | --- | --- | --- | --- | --- | --- |
| 96 | CD69 | Q07108 | 969 | CD69 molecule | 0.7046565 | -3.419194 | 3.887112 | 0.0013245586 | 0.011547480 | -1.0994935 | Q07108 | CD69 molecule | 969 | 0.6508333 | 5.002735 | 12.46840 | 4.138939e-04 | 2.453168e-03 |
| 98 | CD80 | P33681 | 941 | CD80 molecule | 0.2885618 | -3.342227 | 3.067742 | 0.0074089378 | 0.035738128 | -2.7603505 | A0N0P2 | CD80 molecule | 941 | 1.1486172 | 7.342943 | 23.05586 | 1.573626e-06 | 2.388046e-05 |
| 666 | TNFAIP2 | Q03169 | 7127 | TNF alpha induced protein 2 | 0.5913617 | -3.373330 | 4.908859 | 0.0001605736 | 0.003302248 | 0.9650257 | Q03169 | TNF alpha induced protein 2 | 7127 | 1.5653892 | 6.504461 | 35.12936 | 3.085137e-09 | 1.269259e-07 |
| 670 | TNFRSF8 | P28908 | 943 | TNF receptor superfamily member 8 | 0.2313926 | -3.343165 | 3.764099 | 0.0017153573 | 0.013703585 | -1.3508794 | A5D8T4 | TNF receptor superfamily member 8 | 943 | 1.0822797 | 7.370087 | 55.39135 | 9.877033e-14 | 2.045659e-11 |

|  | gene | uniprot | name | entrez | logFC | logCPM | LR | PValue | FDR |
| --- | --- | --- | --- | --- | --- | --- | --- | --- | --- |
| CD69 | CD69 | Q07108 | CD69 molecule | 969 | 0.6508333 | 5.002735 | 12.46840 | 4.138939e-04 | 2.453168e-03 |
| CD80 | CD80 | A0N0P2 | CD80 molecule | 941 | 1.1486172 | 7.342943 | 23.05586 | 1.573626e-06 | 2.388046e-05 |
| TNFRSF8 | TNFRSF8 | A5D8T4 | TNF receptor superfamily member 8 | 943 | 1.0822797 | 7.370087 | 55.39135 | 9.877033e-14 | 2.045659e-11 |
| TNFRSF9 | TNFRSF9 | Q07011 | TNF receptor superfamily member 9 | 3604 | 1.5650770 | 3.956294 | 24.25431 | 8.441812e-07 | 1.425222e-05 |
| NA | NA | NA | NA | NA | NA | NA | NA | NA | NA |
| TNFAIP2 | TNFAIP2 | Q03169 | TNF alpha induced protein 2 | 7127 | 1.5653892 | 6.504461 | 35.12936 | 3.085137e-09 | 1.269259e-07 |

|  | uniprot | RefSeqId | Gene | Name | logFC | AveExpr | t | P.Value | adj.P.Val | B |
| --- | --- | --- | --- | --- | --- | --- | --- | --- | --- | --- |
| TNFAIP2 | Q03169 | 7127 | TNFAIP2 | TNF alpha induced protein 2 | 0.5913617 | -3.373330 | 4.908859 | 0.0001605736 | 0.003302248 | 0.9650257 |
| CD69 | Q07108 | 969 | CD69 | CD69 molecule | 0.7046565 | -3.419194 | 3.887112 | 0.0013245586 | 0.011547480 | -1.0994935 |
| TNFRSF8 | P28908 | 943 | TNFRSF8 | TNF receptor superfamily member 8 | 0.2313926 | -3.343165 | 3.764099 | 0.0017153573 | 0.013703585 | -1.3508794 |
| CD80 | P33681 | 941 | CD80 | CD80 molecule | 0.2885618 | -3.342227 | 3.067742 | 0.0074089378 | 0.035738128 | -2.7603505 |

# Pathway analysis¶

The pathway analysis is performed using `camera` against the MSIGDB Hallmark and Canonical pathways (C2 CP) collection version 6.1.

Only pathways with a size between 15 and 300 genes are kept.

In [25]:

```
# Prepare pathways

# Function to read GMT files
read.gmt <- function (file) 
{
    if (!grepl("\\.gmt$", file)[1]) {
        stop("Pathway information must be a .gmt file")
    }
    geneSetDB = readLines(file)
    geneSetDB = strsplit(geneSetDB, "\t")
    names(geneSetDB) = sapply(geneSetDB, "[", 1)
    geneSetDB = lapply(geneSetDB, "[", -1:-2)
    geneSetDB = lapply(geneSetDB, function(x) {
        x[which(x != "")]
    })
    return(geneSetDB)
}

# Load the pathways
pathways <- c(read.gmt("data/h.all.v6.1.entrez.gmt"), read.gmt("data/c2.cp.v6.1.entrez.gmt"))

message("Loaded ", length(pathways), " pathways")

# Filter based on size
min.pathway.size <- 15
max.pathway.size <- 300

pathways.size <- lapply(pathways, length)
pathways.to.remove <- pathways.size < min.pathway.size | pathways.size > max.pathway.size

pathways <- pathways[!pathways.to.remove]

message("Kept ", length(pathways), " with >= 15 and <= 300 gene")
```

```
Loaded 1379 pathways
Kept 1119 with >= 15 and <= 300 gene
```

Run the pathway analysis on the RNASeq and Proteomics data:

In [26]:

```
camera.rna <- camera(voom(rnaseq.dge), ids2indices(pathways, rnaseq.dge$genes$entrez), rnaseq.design, contrast = "treatment")
camera.prot <- camera(proteomics.el, ids2indices(pathways, proteomics.el$genes$RefSeqId), prot.design, contrast = contrast.matrix[,1])

# merge the pathway results
camera.merged <- merge(camera.rna, camera.prot, by.x = 0, by.y = 0, all.x = T, all.y = T,
                       suffixes = c(".rna", ".prot"))

rownames(camera.merged) <- camera.merged$Row.names
camera.merged$Row.names <- NULL

camera.merged <- camera.merged[order(camera.merged$FDR.rna), ]
```

## Up-regulated pathways¶

First, simply display up-regulated pathways:

In [27]:

```
camera.up <- camera.merged[camera.merged$Direction.rna == "Up", ]
camera.up[1:20,]
```

|  | NGenes.rna | Direction.rna | PValue.rna | FDR.rna | NGenes.prot | Direction.prot | PValue.prot | FDR.prot |
| --- | --- | --- | --- | --- | --- | --- | --- | --- |
| HALLMARK\_TNFA\_SIGNALING\_VIA\_NFKB | 188 | Up | 4.745504e-09 | 1.397426e-07 | 64 | Up | 1.965979e-05 | 0.0005416272 |
| KEGG\_GRAFT\_VERSUS\_HOST\_DISEASE | 29 | Up | 1.057024e-07 | 2.274634e-06 | 20 | Up | 8.998655e-03 | 0.0718588226 |
| KEGG\_AUTOIMMUNE\_THYROID\_DISEASE | 27 | Up | 5.104540e-07 | 8.399970e-06 | 21 | Up | 5.252986e-03 | 0.0482399178 |
| HALLMARK\_INTERFERON\_GAMMA\_RESPONSE | 188 | Up | 5.552549e-06 | 6.680970e-05 | 126 | Up | 5.669712e-04 | 0.0091882692 |
| SIG\_CD40PATHWAYMAP | 33 | Up | 6.880552e-06 | 8.078336e-05 | 24 | Up | 2.390800e-01 | 0.5905304988 |
| KEGG\_TYPE\_I\_DIABETES\_MELLITUS | 34 | Up | 7.862595e-06 | 8.977800e-05 | 21 | Up | 8.661084e-03 | 0.0701802539 |
| KEGG\_ALLOGRAFT\_REJECTION | 29 | Up | 1.005796e-05 | 1.082198e-04 | 21 | Up | 5.252986e-03 | 0.0482399178 |
| HALLMARK\_INTERFERON\_ALPHA\_RESPONSE | 96 | Up | 1.194633e-05 | 1.231637e-04 | 72 | Up | 5.954042e-03 | 0.0529141460 |
| REACTOME\_INTERFERON\_GAMMA\_SIGNALING | 54 | Up | 1.709571e-05 | 1.649147e-04 | 41 | Up | 8.205793e-03 | 0.0679908568 |
| KEGG\_CELL\_ADHESION\_MOLECULES\_CAMS | 92 | Up | 4.917929e-05 | 4.402530e-04 | 46 | Up | 3.531014e-02 | 0.1945588832 |
| PID\_CD40\_PATHWAY | 28 | Up | 5.634533e-05 | 5.004002e-04 | 19 | Up | 1.398703e-01 | 0.4317564462 |
| REACTOME\_INTERFERON\_ALPHA\_BETA\_SIGNALING | 50 | Up | 2.415996e-04 | 1.890559e-03 | 36 | Up | 2.562159e-02 | 0.1559944203 |
| HALLMARK\_INFLAMMATORY\_RESPONSE | 164 | Up | 3.580489e-04 | 2.699602e-03 | 54 | Up | 7.634765e-02 | 0.3139369650 |
| BIOCARTA\_CD40\_PATHWAY | 14 | Up | 4.849967e-04 | 3.547133e-03 | 10 | Up | 5.033207e-01 | 0.8205755802 |
| KEGG\_LEISHMANIA\_INFECTION | 63 | Up | 5.031368e-04 | 3.632323e-03 | 45 | Up | 5.113366e-01 | 0.8214358182 |
| KEGG\_ASTHMA | 17 | Up | 8.004586e-04 | 5.633416e-03 | 14 | Up | 5.927531e-02 | 0.2708672770 |
| KEGG\_INTESTINAL\_IMMUNE\_NETWORK\_FOR\_IGA\_PRODUCTION | 39 | Up | 9.117791e-04 | 6.376755e-03 | 23 | Up | 2.008761e-01 | 0.5372948553 |
| ST\_GAQ\_PATHWAY | 26 | Up | 9.554952e-04 | 6.640989e-03 | 16 | Up | 7.567418e-01 | 0.9164059717 |
| ST\_GA13\_PATHWAY | 33 | Up | 1.264346e-03 | 8.371619e-03 | 22 | Up | 8.292398e-01 | 0.9370216423 |
| BIOCARTA\_IL22BP\_PATHWAY | 13 | Up | 1.593517e-03 | 1.042775e-02 | 10 | Up | 2.817788e-01 | 0.6364609848 |

For the top-20 up-regulated pathways based on the RNASeq results, regulation is the same for the proteomics and the RNASeq data.

## Down-regulated pathways¶

In [28]:

```
camera.down <- camera.merged[camera.merged$Direction.rna == "Down", ]
camera.down[1:20,]
```

|  | NGenes.rna | Direction.rna | PValue.rna | FDR.rna | NGenes.prot | Direction.prot | PValue.prot | FDR.prot |
| --- | --- | --- | --- | --- | --- | --- | --- | --- |
| HALLMARK\_E2F\_TARGETS | 200 | Down | 4.074203e-27 | 4.559033e-24 | 166 | Down | 4.409899e-11 | 6.074635e-09 |
| HALLMARK\_G2M\_CHECKPOINT | 197 | Down | 3.456393e-20 | 1.933852e-17 | 148 | Down | 1.213750e-09 | 1.215957e-07 |
| HALLMARK\_MYC\_TARGETS\_V1 | 200 | Down | 3.994096e-19 | 1.117348e-16 | 192 | Down | 3.160798e-04 | 5.528888e-03 |
| REACTOME\_DNA\_REPLICATION | 185 | Down | 3.474210e-19 | 1.117348e-16 | 155 | Down | 2.107152e-07 | 1.161041e-05 |
| REACTOME\_MITOTIC\_M\_M\_G1\_PHASES | 166 | Down | 6.403242e-18 | 1.433046e-15 | 139 | Down | 3.466086e-06 | 1.123420e-04 |
| REACTOME\_PROCESSING\_OF\_CAPPED\_INTRON\_CONTAINING\_PRE\_MRNA | 135 | Down | 2.455341e-16 | 4.579211e-14 | 129 | Down | 1.250015e-01 | 4.087585e-01 |
| REACTOME\_MRNA\_SPLICING | 106 | Down | 7.574435e-15 | 1.210828e-12 | 101 | Down | 1.061793e-02 | 8.182488e-02 |
| REACTOME\_MRNA\_PROCESSING | 152 | Down | 1.022672e-13 | 1.430462e-11 | 142 | Down | 2.818448e-01 | 6.364610e-01 |
| REACTOME\_G1\_S\_TRANSITION | 104 | Down | 1.279304e-13 | 1.590602e-11 | 87 | Down | 2.735824e-05 | 7.178281e-04 |
| KEGG\_SPLICEOSOME | 126 | Down | 1.613995e-13 | 1.641873e-11 | 115 | Down | 2.734386e-03 | 3.074789e-02 |
| REACTOME\_MITOTIC\_PROMETAPHASE | 85 | Down | 1.551943e-13 | 1.641873e-11 | 71 | Down | 9.058829e-07 | 3.442355e-05 |
| REACTOME\_SYNTHESIS\_OF\_DNA | 89 | Down | 3.904873e-13 | 3.641294e-11 | 80 | Down | 3.549913e-04 | 6.112506e-03 |
| REACTOME\_S\_PHASE | 105 | Down | 5.090648e-13 | 4.381873e-11 | 89 | Down | 9.528431e-05 | 1.909151e-03 |
| REACTOME\_TCA\_CYCLE\_AND\_RESPIRATORY\_ELECTRON\_TRANSPORT | 126 | Down | 8.496436e-13 | 6.791080e-11 | 110 | Up | 2.889914e-20 | 1.592343e-17 |
| REACTOME\_M\_G1\_TRANSITION | 77 | Down | 1.724119e-12 | 1.286192e-10 | 65 | Down | 1.214386e-02 | 9.103765e-02 |
| REACTOME\_MITOTIC\_G1\_G1\_S\_PHASES | 128 | Down | 1.852972e-12 | 1.295922e-10 | 102 | Down | 3.996756e-05 | 9.106535e-04 |
| REACTOME\_CELL\_CYCLE\_CHECKPOINTS | 111 | Down | 2.359168e-12 | 1.552888e-10 | 93 | Down | 1.706225e-03 | 2.265373e-02 |
| REACTOME\_G2\_M\_CHECKPOINTS | 41 | Down | 2.666151e-12 | 1.657457e-10 | 35 | Down | 1.429496e-04 | 2.670007e-03 |
| REACTOME\_RESPIRATORY\_ELECTRON\_TRANSPORT\_ATP\_SYNTHESIS\_BY\_CHEMIOSMOTIC\_COUPLING\_AND\_HEAT\_PRODUCTION\_BY\_UNCOUPLING\_PROTEINS\_ | 92 | Down | 3.133338e-12 | 1.845371e-10 | 79 | Up | 3.262981e-21 | 3.595805e-18 |
| REACTOME\_MRNA\_SPLICING\_MINOR\_PATHWAY | 41 | Down | 5.622060e-12 | 3.145543e-10 | 38 | Down | 1.871906e-02 | 1.234045e-01 |

## Analysis using EnrichmentMap¶

The pathway results were further analyzed using the Cytoscape `EnrichmentMap` plugin. The following code formats the pathway results according to the plugin's format. One individual file is created for RNASeq and proteomics data. This allows one to load the two as separate datasets into EnrichmentMap and visualize them next to each other.

In [29]:

```
# create output for EnrichmentMap
enr.prot <- camera.prot
enr.prot$ID <- rownames(enr.prot)
enr.prot$Description <- rownames(enr.prot)
enr.prot <- enr.prot[, c("ID", "Description", "PValue", "FDR", "Direction")]
write.table(enr.prot, file = "plots/camera_prot.txt", quote = F, sep = "\t", row.names = F)

enr.rna <- camera.rna
enr.rna$ID <- rownames(enr.rna)
enr.rna$Description <- rownames(enr.rna)
enr.rna <- enr.rna[, c("ID", "Description", "PValue", "FDR", "Direction")]
write.table(enr.rna, file = "plots/camera_rna.txt", quote = F, sep = "\t", row.names = F)
```

The two files together with the `h.all_c2.cp_v6.1.gmt` holding all pathways were loaded into `EnrichmentMap` version .... as `Generic/gProfiler` datasets. All settings were left at their default. No filtering based on p-value was performed. Thereby, the p-value is directly visualized in EnrichmentMap.

# FACS Analysis¶

## Load data¶

In [30]:

```
zScore <- function(values) {
    return( (values - mean(values)) / sd(values))
}

facs.data <- read.csv("data/mcm_induction_facs_geo_mean.csv", 
                      sep = "\t", blank.lines.skip = T, 
                      dec = ",", stringsAsFactors = F)

facs.data <- facs.data[!is.na(facs.data[, 2]), ]

# normalize the values 
for (nCol in 2:ncol(facs.data)) {
    facs.data[, nCol] <- zScore(facs.data[, nCol])
}

facs.plot.data <- melt(facs.data, id.vars = 1, variable.name = "gene", value.name = "Expression")
facs.plot.data <- data.frame(facs.plot.data, stringsAsFactors = F)
pattern <- "[^_]*_([^_ ]*)[ _]([^ ]*) ([^_]*)_.*"
# highlight in the patient id that these were all run with MCM from patient 1
facs.plot.data$patient.id <- gsub(pattern, "\\1-M1", facs.plot.data$X)
facs.plot.data$cell.type <- gsub(pattern, "\\2", facs.plot.data$X)
facs.plot.data$treatment <- gsub(pattern, "\\3", facs.plot.data$X)
facs.plot.data$gene <- as.character(facs.plot.data$gene)
#facs.plot.data$gene[facs.plot.data$gene == "CD20"] <- "MS4A1"
#facs.plot.data$gene[facs.plot.data$gene == "CD138"] <- "SDC1"
facs.plot.data$cell.type[facs.plot.data$cell.type == "PBMC"] <- "PBMCB"
facs.plot.data$line_group <- paste0(facs.plot.data$patient.id, facs.plot.data$cell.type)

facs.plot.data$treatment <- factor(facs.plot.data$treatment, levels = c("Mock", "MCM"), labels = c("MOCK", "MCM"))

# add the data from the second batch
facs.data <- read.csv("data/mcm_induction_facs_geo_mean_second.csv", dec = ",")

# normalize the values 
for (nCol in 2:ncol(facs.data)) {
    facs.data[, nCol] <- zScore(facs.data[, nCol])
}

facs.plot.data.new <- melt(facs.data, id.vars = 1, variable.name = "gene", value.name = "Expression")
facs.plot.data.new <- data.frame(facs.plot.data.new, stringsAsFactors = F)
pattern <- "P([^_]*)_([^_]*)_([^_]*)"
# here, autologous MCM was used in all experiments
facs.plot.data.new$patient.id <- gsub(pattern, "P\\1-M\\1", facs.plot.data.new$X)
# not true for P2
facs.plot.data.new$patient.id[grep("P2", facs.plot.data.new$patient.id)] <- "P2-M1"
facs.plot.data.new$cell.type <- gsub(pattern, "\\2", facs.plot.data.new$X)
facs.plot.data.new$treatment <- gsub(pattern, "\\3", facs.plot.data.new$X)
facs.plot.data.new$gene <- as.character(facs.plot.data.new$gene)
facs.plot.data.new$cell.type[facs.plot.data.new$cell.type == "PBMC"] <- "PBMCB"
facs.plot.data.new$line_group <- paste0(facs.plot.data.new$patient.id, facs.plot.data.new$cell.type)
```

In [31]:

```
facs.plot.data <- rbind(facs.plot.data, facs.plot.data.new)
facs.plot.data$cell.type[facs.plot.data$cell.type == "TIBC"] <- "TAB"
```

In [32]:

```
table(facs.plot.data$gene)
```

```
CD138  CD19  CD20  CD24  CD27  CD38   IgD   IgG   IgM 
   24    24    24    24    24    24    24    24    24
```

## Plot FACS data¶

In [33]:

```
# limit to relevant genes
relevant.genes <- c("CD20", "CD19", "CD24", "CD27", "CD38", "CD138")
org.plot.data <- facs.plot.data
facs.plot.data <- facs.plot.data[facs.plot.data$gene %in% relevant.genes, ]
facs.plot.data$gene <- factor(facs.plot.data$gene, levels = relevant.genes,
                         labels = c("CD20 (MS4A1)", "CD19", "CD24", "CD27", "CD38", "CD138 (SDC1)"))

plot.obj <- ggplot(facs.plot.data, aes(x = treatment, y = Expression, color = patient.id, group = line_group, linetype = cell.type)) +
    facet_wrap( ~ gene, ncol = 2) +
    geom_point() +
    geom_line() +
    scale_color_brewer(palette = "Set1") +
    labs(x = "Treatment", y = "Expression (z-score)", color = "Patient", linetype = "Cell type") +
    my_theme_bw

setPlotSize(4,4)
plot.obj
resetPlotSize()

# create plot for other genes
# limit to relevant genes
relevant.genes <- c("CD20", "CD19", "CD24", "CD27", "CD38", "CD138")
supp.plot.data <- org.plot.data[!org.plot.data$gene %in% relevant.genes, ]

plot.obj <- ggplot(supp.plot.data, aes(x = treatment, y = Expression, color = patient.id, group = line_group, linetype = cell.type)) +
    facet_wrap( ~ gene, ncol = 2) +
    geom_point() +
    geom_line() +
    scale_color_brewer(palette = "Set1") +
    labs(x = "Treatment", y = "Expression (z-score)", color = "Patient", linetype = "Cell type") +
    my_theme_bw

setPlotSize(4,5)
plot.obj
resetPlotSize()

svg("plots/Supplementary_Figure_3C-FACS_AB.svg", width = 5, height = 4)
print(plot.obj)
dev.off()
```

**png:** 2

In [34]:

```
plot.obj <- ggplot(facs.plot.data[facs.plot.data$gene == "CD20 (MS4A1)",], aes(x = treatment, y = Expression, color = patient.id, group = line_group, linetype = cell.type)) +
    geom_point() +
    geom_line() +
    scale_color_brewer(palette = "Set1") +
    labs(x = "Treatment", y = "Expression (z-score)", color = "Patient", linetype = "Cell type") +
    my_theme_bw +
    labs(title = "CD20")
setPlotSize(3,4)
plot.obj
```

## Combined FACS, RNA-seq, and Proteomics¶

Create a combined plot of expression values:

In [35]:

```
# adapt the relevent gene names
relevant.genes <- c("MS4A1", "CD19", "CD24", "CD27", "CD38", "SDC1")
```

Add the proteomics expression values:

In [36]:

```
prot.expr <- proteomics.el$E[proteomics.el$genes$Gene %in% relevant.genes, ]

# zscore normalizse
prot.expr <- data.frame(t(apply(prot.expr, 1, zScore)))

prot.expr <- prot.expr[, 
                       grepl("MOCK", colnames(prot.expr)) | 
                       grepl("MCM", colnames(prot.expr))]
prot.expr$Gene <- rownames(prot.expr)

# fix the patient ids
colnames(prot.expr) <- gsub("\\.", "-", colnames(prot.expr))

prot.expr <- melt(prot.expr, id.vars = c("Gene"), variable.name = "X", 
                  value.name = "Expression")

prot.expr$method <- "Proteomics"

pattern <- "([^ _]*)[ _]([^ _]*)[ _](.*)"
prot.expr$patient.id <- gsub(pattern, "\\1", prot.expr$X)
prot.expr$cell.type <- gsub(pattern, "\\2", prot.expr$X)
prot.expr$treatment <- gsub(pattern, "\\3", prot.expr$X)
prot.expr <- prot.expr[!grepl("all", prot.expr$patient.id), ]

head(prot.expr)
```

| Gene | X | Expression | method | patient.id | cell.type | treatment |
| --- | --- | --- | --- | --- | --- | --- |
| CD19 | P1-M1\_PBMCB\_MOCK | 0.6571349 | Proteomics | P1-M1 | PBMCB | MOCK |
| CD27 | P1-M1\_PBMCB\_MOCK | 1.7995860 | Proteomics | P1-M1 | PBMCB | MOCK |
| SDC1 | P1-M1\_PBMCB\_MOCK | -0.3305744 | Proteomics | P1-M1 | PBMCB | MOCK |
| CD38 | P1-M1\_PBMCB\_MOCK | 0.6558121 | Proteomics | P1-M1 | PBMCB | MOCK |
| MS4A1 | P1-M1\_PBMCB\_MOCK | 0.6883212 | Proteomics | P1-M1 | PBMCB | MOCK |
| CD19 | P1-M1\_PBMCB\_MCM | -0.9567879 | Proteomics | P1-M1 | PBMCB | MCM |

Add the RNA-seq expression values:

In [37]:

```
rna.expr <- cpm(rnaseq.dge, log = T, prior.count = 2)[rnaseq.dge$genes$gene %in% relevant.genes,]

# zScore normalize
rna.expr <- data.frame(t(apply(rna.expr, 1, zScore)))

rna.expr$Gene <- rownames(rna.expr)
rna.expr <- melt(rna.expr, id.vars = c("Gene"), variable.name = "X",
               value.name = "Expression")

rna.expr$method <- "RNASeq"
rna.expr$X <- gsub("\\.", "-", rna.expr$X)
rna.expr$patient.id <- gsub(pattern, "\\1", rna.expr$X)
rna.expr$cell.type <- gsub(pattern, "\\2", rna.expr$X)
rna.expr$treatment <- gsub(pattern, "\\3", rna.expr$X)

head(rna.expr)
```

| Gene | X | Expression | method | patient.id | cell.type | treatment |
| --- | --- | --- | --- | --- | --- | --- |
| MS4A1 | P3-M1\_TAB\_MOCK | 2.16056156 | RNASeq | P3-M1 | TAB | MOCK |
| CD27 | P3-M1\_TAB\_MOCK | -0.88848476 | RNASeq | P3-M1 | TAB | MOCK |
| CD19 | P3-M1\_TAB\_MOCK | 0.23649868 | RNASeq | P3-M1 | TAB | MOCK |
| CD38 | P3-M1\_TAB\_MOCK | -0.07847775 | RNASeq | P3-M1 | TAB | MOCK |
| SDC1 | P3-M1\_TAB\_MOCK | -1.74230559 | RNASeq | P3-M1 | TAB | MOCK |
| CD24 | P3-M1\_TAB\_MOCK | 0.91264147 | RNASeq | P3-M1 | TAB | MOCK |

Combine all data types:

In [38]:

```
combined.expr <- rbind(prot.expr, rna.expr)

# fix some gene labels
combined.expr$Gene[combined.expr$Gene == "MS4A1"] <- "CD20 (MS4A1)"
combined.expr$Gene[combined.expr$Gene == "SDC1"] <- "CD138 (SDC1)"

# add the FACS data
facs.plot.data$method <- "FACS"
facs.plot.data$Gene <- facs.plot.data$gene

combined.expr <- rbind(combined.expr, facs.plot.data[, c("Gene", "X", "Expression", "method", "patient.id", "cell.type", "treatment")])
combined.expr$line_group <- paste0(combined.expr$patient.id, combined.expr$cell.type)

combined.expr <- combined.expr[combined.expr$patient.id != "all", ]
combined.expr$treatment <- factor(combined.expr$treatment, levels=c("MOCK", "MCM"))
```

In [39]:

```
plot.obj <- ggplot(combined.expr, aes(x = treatment, y = Expression, color = patient.id, group = line_group, linetype = cell.type)) +
    facet_grid(method ~ Gene, scales = "free_y") +
    geom_point() +
    geom_line() +
    scale_color_brewer(palette = "Set1") +
    labs(x = "Treatment", y = "Expression (z-score)", color = "Patient", linetype = "Cell type") +
    my_theme_bw +
    theme(panel.grid = element_blank())

#combined.expr
setPlotSize(4,10)
plot.obj

svg("plots/Figure_2C_combined_FACS_Proteomic_RNASeq_expr.svg", width = 10, height = 4)
print(plot.obj)
dev.off()
#prot.expr <- merge(prot.expr, proteomics.el$samples, by.x = "variable", by.y = 0)
#colnames(prot.expr) <- c("X", )
```

**png:** 2

## Check MS4A1 (CD20)¶

In [40]:

```
ggplot(combined.expr[combined.expr$Gene == "CD20 (MS4A1)",], aes(x = treatment, y = Expression, color = patient.id, group = line_group, linetype = cell.type)) +
    facet_wrap( ~ method, scales = "free_y") +
    geom_point() +
    geom_line() +
    scale_color_brewer(palette = "Set1") +
    labs(x = "Treatment", y = "Expression (z-score)", color = "Patient", linetype = "Cell type") +
    my_theme_bw +
    labs(title = "CD20 (MS4A1)")
```

# Supplementary tables¶

In [41]:

```
library(xlsx)

# RNASeq results
write.xlsx(rnaseq.sig, sheetName = "RNASeq", file = "plots/Supplementary_Table_1-MCM_Induction.xlsx")

# Proteomics results
write.xlsx(prot.sig, sheetName = "Proteomics", file = "plots/Supplementary_Table_1-MCM_Induction.xlsx", append = T)

# Phospho result
write.xlsx(phospho.sig, sheetName = "Phospho-proteomics", file = "plots/Supplementary_Table_1-MCM_Induction.xlsx", append = T)

# Pathway results
write.xlsx(camera.merged, sheetName = "Pathway analysis (Camera)", file = "plots/Supplementary_Table_1-MCM_Induction.xlsx", append = T)
```

# Used packages¶

In [42]:

```
sessionInfo()
```

```
R version 3.6.0 (2019-04-26)
Platform: x86_64-pc-linux-gnu (64-bit)
Running under: Ubuntu 18.04.2 LTS

Matrix products: default
BLAS:   /usr/lib/x86_64-linux-gnu/blas/libblas.so.3.7.1
LAPACK: /usr/lib/x86_64-linux-gnu/lapack/liblapack.so.3.7.1

locale:
 [1] LC_CTYPE=en_US.UTF-8          LC_NUMERIC=C                 
 [3] LC_TIME=de_AT.UTF-8           LC_COLLATE=en_US.UTF-8       
 [5] LC_MONETARY=de_AT.UTF-8       LC_MESSAGES=en_US.UTF-8      
 [7] LC_PAPER=de_AT.UTF-8          LC_NAME=de_AT.UTF-8          
 [9] LC_ADDRESS=de_AT.UTF-8        LC_TELEPHONE=de_AT.UTF-8     
[11] LC_MEASUREMENT=de_AT.UTF-8    LC_IDENTIFICATION=de_AT.UTF-8

attached base packages:
[1] parallel  stats4    stats     graphics  grDevices utils     datasets 
[8] methods   base     

other attached packages:
 [1] xlsx_0.6.1           MSnbase_2.6.4        ProtGenerics_1.12.0 
 [4] BiocParallel_1.14.2  mzR_2.14.0           Rcpp_1.0.0          
 [7] edgeR_3.22.5         limma_3.36.5         org.Hs.eg.db_3.6.0  
[10] AnnotationDbi_1.42.1 IRanges_2.14.12      S4Vectors_0.18.3    
[13] Biobase_2.40.0       BiocGenerics_0.26.0  reshape2_1.4.3      
[16] ggplot2_3.1.0       

loaded via a namespace (and not attached):
 [1] vsn_3.48.1            splines_3.6.0         foreach_1.4.4        
 [4] bit64_0.9-7           jsonlite_1.6          assertthat_0.2.0     
 [7] getPass_0.2-2         affy_1.58.0           xlsxjars_0.6.1       
[10] blob_1.1.1            impute_1.54.0         pillar_1.3.1         
[13] RSQLite_2.1.1         lattice_0.20-38       glue_1.3.0           
[16] uuid_0.1-2            digest_0.6.18         RColorBrewer_1.1-2   
[19] colorspace_1.4-0      htmltools_0.3.6       preprocessCore_1.42.0
[22] plyr_1.8.4            MALDIquant_1.18       XML_3.98-1.16        
[25] pkgconfig_2.0.2       zlibbioc_1.26.0       purrr_0.2.5          
[28] scales_1.0.0          affyio_1.50.0         tibble_2.0.1         
[31] withr_2.1.2           repr_0.19.1           lazyeval_0.2.1       
[34] magrittr_1.5          crayon_1.3.4          memoise_1.1.0        
[37] evaluate_0.12         doParallel_1.0.14     MASS_7.3-51.1        
[40] BiocInstaller_1.30.0  tools_3.6.0           stringr_1.3.1        
[43] munsell_0.5.0         locfit_1.5-9.1        bindrcpp_0.2.2       
[46] pcaMethods_1.72.0     compiler_3.6.0        mzID_1.18.0          
[49] rlang_0.3.1           grid_3.6.0            iterators_1.0.10     
[52] pbdZMQ_0.3-3          IRkernel_0.8.15       base64enc_0.1-3      
[55] labeling_0.3          gtable_0.2.0          codetools_0.2-16     
[58] DBI_1.0.0             R6_2.3.0              dplyr_0.7.8          
[61] bit_1.1-14            bindr_0.1.1           rJava_0.9-10         
[64] stringi_1.2.4         IRdisplay_0.7.0       tidyselect_0.2.5
```
