# Supplementary material for: B cells sustain inflammation and predict response to immune checkpoint blockade in human melanoma
Source: Nat Commun. 2019 Sep 13;10:4186. doi: 10.1038/s41467-019-12160-2 (PMC6744450; doi:10.1038/s41467-019-12160-2)
Supplement: Supplementary file 8 — Supplementary Software 1 [file 41467_2019_12160_MOESM8_ESM.zip › 193518_1_other_0_psz9cz.zip!/Sade-Feldman - Seurat analysis.html]

Sade-Feldman - Seurat analysis


## Introduction¶

This Notebook contains the complete R code used to analyze the scRNASeq dataset published by Sade-Feldman *et al.*. The data was directly downlaoded from GEO (GSE120575).

In [ ]:

```
# This follows: https://satijalab.org/seurat/pbmc3k_tutorial.html
suppressPackageStartupMessages(library(Seurat))
library(ggplot2)

org.options <- options

# add some convenience functions
setPlotSize <- function(height, width) {
    options(repr.plot.height=height, repr.plot.width = width)
}

resetPlotSize <- function() {
    options(org.options)
}

my_theme_bw <- theme_bw() +
    theme(strip.background = element_blank(),
          panel.border = element_rect(colour = "black"),
          panel.grid.major = element_blank(), panel.grid.minor = element_blank())
```

## Load the data¶

In [8]:

```
# get the epxression data
exprs.data <- read.csv("GSE120575_Sade_Feldman_melanoma_single_cells_TPM_GEO.txt", sep = "\t")
sample.ids <- colnames(exprs.data)[2:ncol(exprs.data)]
exprs.data[, ncol(exprs.data)] <- NULL
colnames(exprs.data) <- sample.ids
rm(sample.ids)

# load the sample metadata
sample.data <- read.csv("GSE120575_patient_ID_single_cells.txt", sep = "\t")
rownames(sample.data) <- sample.data[, "title"]

# remove unused columns
sample.data <- sample.data[, 1:12]
colnames(sample.data) <- gsub("characteristics\\.\\.", "", colnames(sample.data))
sample.data$timepoint <- "pre"
sample.data$timepoint[grep("Post_", sample.data$patinet.ID..Pre.baseline..Post..on.treatment.)] <- "on"
sample.data$patient <- gsub(".*_", "", sample.data$patinet.ID..Pre.baseline..Post..on.treatment.)

# fix some differences in the sample ids
rownames(sample.data) <- gsub("-", ".", rownames(sample.data))

# sanity check to make sure that all samples have corresponding annotations
if (!all(colnames(exprs.data) %in% rownames(sample.data))) {
    stop("Missing sample annotations")
}

# order the sample metadata like the columns in the epxression matrix
sample.data <- sample.data[colnames(exprs.data), ]

# create the Seurat object
sade <- CreateSeuratObject(raw.data = exprs.data, 
                           min.cells = 10, 
                           min.genes = 200, 
                           project = "Sade-Feldman",
                           names.delim = " ",
                           meta.data = sample.data)
```

## Normalize¶

In [ ]:

```
# normalize
sade <- NormalizeData(object = sade, normalization.method = "LogNormalize", scale.factor = 10000)

# find the variable genes
sade <- FindVariableGenes(object = sade, mean.function = ExpMean, dispersion.function = LogVMR, 
                          x.low.cutoff = 0.0125, x.high.cutoff = 3, y.cutoff = 0.5)

# correct for confounders
sade <- ScaleData(sade, vars.to.regress = c("patient"), do.par = T, num.cores = 4)

saveRDS(sade, file = "sade_scaled.rds")
```

In [ ]:

```
sade <- readRDS("sade_scaled.rds")

# perform the PCA
sade <- RunPCA(sade, pc.genes = sade@var.genes, do.print = FALSE, 
               pcs.print = 1:5, genes.print = 5, pcs.compute = 100)

saveRDS(sade, file = "sade_pca.rds")
```

## PC Analysis¶

Explore the PCA analysis, determine which genes are most important to every PC and how many PCs to use for the further clustering analysis

In [ ]:

```
sade <- readRDS("sade_pca.rds")
```

In [ ]:

```
setPlotSize(5,5)
PCHeatmap(sade, pc.use = c(30:40), cells.use = 500, do.balanced = TRUE, label.columns = FALSE, use.full = FALSE)
```

In [ ]:

```
setPlotSize(3,3)
PCElbowPlot(object = sade, num.pc = 100)
```

Base on this analysis, we used the **top 35 PCs** for the further analysis.

## Cluster cells¶

Clustering was also tested with a resolution of 0.3 but this joined several visually distinct clusters in the tSNE plot.

In [ ]:

```
sade <- FindClusters(object = sade, reduction.type = "pca", dims.use = 1:35, 
                     resolution = 0.6, print.output = 0, save.SNN = TRUE, force.recalc = F)
```

In [ ]:

```
sade <- RunTSNE(object = sade, dims.use = 1:35, do.fast = TRUE, perplexity = 20, check_duplicates = F)
```

In [ ]:

```
saveRDS(sade, file = "sade_clusters_06.rds")
```

### Visualize new clustering¶

In [ ]:

```
setPlotSize(6,8)

# add a group to sepearte pre- and post- as well
sade@meta.data$time_response <- paste0(sade@meta.data$response, "-", sade@meta.data$timepoint)

p1 <- TSNEPlot(sade, do.label = T, pt.size = 0.25, do.return = T)

p1
```

### Get cluster markers¶

Note: Cell type markers were downloaded from https://panglaodb.se/markers.html?cell\_type=%27all\_cells%27#

This complete list of markers was used to get a first estimate of a cluster's predominant cell type.

In [ ]:

```
annotateClusterMarkers <- function(cluster.markers, n.jobs = 10) {
    # create the cell marker list
    cell.markers <- read.csv("cell_type_markers.tsv", sep = "\t")
    # limit to human markers
    cell.markers <- cell.markers[grep("Hs", cell.markers$species), ]
    # create a list
    marker.list <- by(cell.markers$official.gene.symbol, cell.markers$cell.type, function(x) as.character(x))
    
    # process this in parallel
    library(parallel)

    shared.res <- list()

    # compare marker list with cluster genes
    for (cluster.id in 1:length(cluster.markers)) {
        top.genes <- rownames(cluster.markers[[cluster.id]])[1:100]

        cl <- makeCluster(10, "FORK")
        # get the shared genes per cell type
        shared.genes <- parSapply(cl, marker.list, function(marker.genes) sum(top.genes %in% marker.genes))
        stopCluster(cl)

        shared.genes <- shared.genes[shared.genes > 0]
        shared.genes <- sort(shared.genes, decreasing = T)

        shared.res[[cluster.id]] <- shared.genes
    }
                                  
    return(shared.res)
}
```

In [ ]:

```
sade <- readRDS("sade_clusters_06.rds")
```

In [ ]:

```
# process this in parallel - for some reason this failed
library(parallel)
cl <- makeCluster(5, "FORK") # 10 jobs require ~ 100 GB memory

clusters.to.process <- 0:19

cluster.markers.06 <- parLapply(cl, clusters.to.process, function(cluster.id) {
    message("Processing cluster ", cluster.id)
    markers <- FindMarkers(object = sade, ident.1 = cluster.id, min.pct = 0.25)
    return(markers)
})

stopCluster(cl)

names(cluster.markers.06) <- as.character(clusters.to.process)

saveRDS(cluster.markers.06, file = "sade_cluster-markers_0.6.rds")
```

In [ ]:

```
cluster.markers.06 <- readRDS("sade_cluster-markers_0.6.rds")
```

In [9]:

```
cluster.annot <- annotateClusterMarkers(cluster.markers.06)
```

Cluster ids were set based on the most commonly identified, plausible cell type based on the cluster annotations. This was done in a very crude fashion since the only main goal was to select possible B cells.

In [10]:

```
cluster.ids.06 <- c("CD8+ T cells", "T memory", "T helper CD4/FOXP3+", "T memory", "B cells", 
                    "Neutrophils", "NK cells", "Epithelial cells", "NK cells", "Macrophages", 
                    "T helper", "T memory", "Plasma cells", "PDCs", "Epithelial cells", 
                    "T memory", "B cells", "NK cells", "NK cells", "B cells")

sade@ident <- plyr::mapvalues(x = sade@ident, from = 0:19, 
                              to = cluster.ids.06)
```

In [11]:

```
setPlotSize(8,10)
TSNEPlot(object = sade, do.label = TRUE, pt.size = 0.5)
```

## Classify B cell subtypes¶

In [12]:

```
b.cells <- SubsetData(sade, ident.use = c("B cells", "Plasma cells"))
saveRDS(b.cells, file = "sade_bcells.rds")
```

### Re-cluster B cells (0.9)¶

In [13]:

```
b.cells <- readRDS("sade_bcells.rds")
```

In [14]:

```
b.cells <- FindClusters(object = b.cells, reduction.type = "pca", dims.use = 1:35, 
                     resolution = 0.9, print.output = 0, save.SNN = TRUE, force.recalc = T)
```

In [15]:

```
saveRDS(b.cells, file = "sade_bcell_clusters_0.9.rds")
```

In [16]:

```
setPlotSize(4,6)
TSNEPlot(b.cells, do.label = T, pt.size = 0.25, do.return = T)
```

### B cell cluster markers¶

In [18]:

```
library(parallel)
cl <- makeCluster(10, "FORK")

clusters.to.process <- 0:7

b.cluster.markers <- parLapply(cl, clusters.to.process, function(cluster.id) {
    markers <- FindMarkers(object = b.cells, ident.1 = cluster.id, min.pct = 0.25)
    return(markers)
})

names(b.cluster.markers) <- as.character(clusters.to.process)

stopCluster(cl)
```

In [19]:

```
saveRDS(b.cluster.markers, file = "sade_bcell_markers.rds")
```

Save markers as XLSX file:

In [20]:

```
b.cells <- readRDS("sade_bcell_clusters_0.9.rds")
b.cluster.markers <- readRDS("sade_bcell_markers.rds")
```

In [21]:

```
library(xlsx)
suppressPackageStartupMessages(library("org.Hs.eg.db"))

annotateGenes <- function(genes) {
    names <- mapIds(org.Hs.eg.db, 
                    keys=genes, 
                    keytype="SYMBOL", 
                    column="GENENAME", 
                    multiVals=function(x) paste0(x, collapse = ", "))
    alias <- mapIds(org.Hs.eg.db, 
                    keys=genes, 
                    keytype="SYMBOL", 
                    column="ALIAS", 
                    multiVals=function(x) paste0(x, collapse = ", "))
                           
    return(data.frame(name = names, alias = alias))
}
```

Export cluster specific markers as Excel file:

In [22]:

```
if (file.exists("sade_bcell_clusters.xlsx")) file.remove("sade_bcell_clusters.xlsx")
names(b.cluster.markers) <- as.character(0:7)
for (i in names(b.cluster.markers)) {
    markers <- head(b.cluster.markers[[i]], 40)
    markers <- cbind(markers, annotateGenes(rownames(markers)))
    
    down <- markers[markers$avg_logFC < 0,]
    up <- markers[markers$avg_logFC > 0,]
    
    if (nrow(down) > 0) {
        write.xlsx(down, sheetName=paste0("Cluster ", i, " (down)"), 
                   file = "sade_bcell_clusters.xlsx",
                  col.names = T, row.names = T, append = T)
    }
    if (nrow(up) > 0) {
        write.xlsx(up, sheetName=paste0("Cluster ", i, " (up)"), 
               file = "sade_bcell_clusters.xlsx",
              col.names = T, row.names = T, append = T)
    }
}
```

TRUE

```
'select()' returned 1:1 mapping between keys and columns
'select()' returned 1:many mapping between keys and columns
'select()' returned 1:1 mapping between keys and columns
'select()' returned 1:many mapping between keys and columns
'select()' returned 1:1 mapping between keys and columns
'select()' returned 1:many mapping between keys and columns
'select()' returned 1:1 mapping between keys and columns
'select()' returned 1:many mapping between keys and columns
'select()' returned 1:1 mapping between keys and columns
'select()' returned 1:many mapping between keys and columns
'select()' returned 1:1 mapping between keys and columns
'select()' returned 1:many mapping between keys and columns
'select()' returned 1:1 mapping between keys and columns
'select()' returned 1:many mapping between keys and columns
'select()' returned 1:1 mapping between keys and columns
'select()' returned 1:many mapping between keys and columns
```

### B cell marker expression¶

In [23]:

```
avg.expression <- AverageExpression(b.cells)
```

```
Finished averaging RNA for cluster 0
Finished averaging RNA for cluster 1
Finished averaging RNA for cluster 2
Finished averaging RNA for cluster 3
Finished averaging RNA for cluster 4
Finished averaging RNA for cluster 5
Finished averaging RNA for cluster 6
Finished averaging RNA for cluster 7
```

In [24]:

```
head(avg.expression)
```

|  | 0 | 1 | 2 | 3 | 4 | 5 | 6 | 7 |
| --- | --- | --- | --- | --- | --- | --- | --- | --- |
| TSPAN6 | 0.0000000 | 0.01417492 | 0.0000000 | 0.000000000 | 0.000000000 | 0.0000000 | 0.000000000 | 0.08922645 |
| DPM1 | 0.6798456 | 0.62714959 | 0.5558179 | 0.979417337 | 0.429884864 | 0.4123429 | 0.739409263 | 1.03619204 |
| SCYL3 | 0.1696566 | 0.25313961 | 0.3202305 | 0.117131227 | 0.383304160 | 0.3581673 | 0.147255459 | 0.30827857 |
| C1orf112 | 0.1572946 | 0.14230613 | 0.1895298 | 0.034309126 | 0.397329462 | 0.1943541 | 0.090105882 | 0.17596434 |
| FGR | 0.5002608 | 0.40621298 | 0.7810506 | 0.093274294 | 1.677707523 | 0.4900431 | 0.006207763 | 0.05928087 |
| CFH | 0.0000000 | 0.03102399 | 0.0000000 | 0.002847371 | 0.001576972 | 0.0000000 | 0.001867765 | 0.04738667 |

In [25]:

```
library(reshape2)
avg.expression$gene <- rownames(avg.expression)
gene.plot.data <- melt(avg.expression, id.var = c("gene"), variable.name = "Cell.type", value.name = "Expression")

plotGenes <- function(genes) {
    available.genes <- genes[genes %in% gene.plot.data$gene]
    
    plot.obj <- ggplot(gene.plot.data[gene.plot.data$gene %in% available.genes,], 
                       aes(x = Cell.type, y = Expression, fill = gene)) +
        geom_bar(stat = "identity", position = "dodge") +
        theme(axis.text.x = element_text(angle = 45, hjust = 1))
    
    if (length(genes) <= 8) {
        plot.obj <- plot.obj + scale_fill_brewer(palette = "Set2")
    }
    
    return(plot.obj)
}
```

In [26]:

```
b.cell.markers <- c("BCL6", "MZB1", "CD5", "CD38", 
                    "FCER2", "SDC1","MME", "CD74",
                   "XBP1", "PRDM1", "CD83", "TGFB1", "CD27", "IRF4", "TNFRSF8", 
                    "CD69", "FLT3", "IL2RA", "MS4A1", "CD22", "SELL", "TNFRSF17",
                   "IL4R", "IGKV3D-15")

b.cell.names <- c("BCL6", "MZB1", "CD5", "CD38", 
                    "FCER2", "CD138 (SDC1)","MME", "CD74",
                   "XBP1", "PRDM1 (BLIMP1)", "CD83", "TGFB1", "CD27", "IRF4", "TNFRSF8 (CD30)", "CD69", "FLT3 (CD135)", "IL2RA (CD25)",
                    "CD20 (MS4A1)", "CD22", "SELL (CD62L)", "TNFRSF17", "IL4R", "IGKV3D-15")

plots <- list()

for (i in 1:length(b.cell.markers)) {
    plots[[i]] <- plotGenes(b.cell.markers[i]) + 
        labs(title=b.cell.names[i]) +
        guides(fill=FALSE)
}

plots[["ncol"]] <- 3

setPlotSize(12,10)
do.call("plot_grid", plots)
```

In [27]:

```
setPlotSize(2,10)
p1 <- plotGenes("IGHG1")
p2 <- plotGenes("IGHG2")
p3 <- plotGenes("IGHG3")
plot_grid(p1, p2, p3, ncol = 3)
```

### Cluster annotations¶

- Cluster 0: memory B cells (CD135+, OK)
- Cluster 1: naive B cell (non class switched)
- Cluster 2: naive B cell (non class switched)
- Cluster 3: Plasma cells (SDC1+)
- Cluster 4: Plasmablasten (IgD, CD20, BCL6, CD30, FCER2, OK)
- Cluster 5: act B cells (CD30, CD135, TGFB1, BCL6, IgG Kappa++)
- Cluster 6: Plasma cellls (SDC1+)
- Cluster 7: Germincal center B cells (OK)

In [ ]:

```
b.cluster.ids <- c("Memory B cell-like", "Naive B cell-like", "Naive B cell-like", 
                   "Plasma cell-like", "Plasmablast-like", "Activated B cell-like", 
                   "Plasma cell-like", "Germinal center B cell-like")

b.cells <- StashIdent(b.cells, "BCellClusterIds_09")
b.cells@ident <- plyr::mapvalues(x = b.cells@ident, from = 0:7, 
                              to = b.cluster.ids)

b.cells@ident <- factor(b.cells@ident, levels = c("Activated B cell-like", "Germinal center B cell-like",
                                                 "Memory B cell-like", "Plasma cell-like",
                                                 "Plasmablast-like", "Naive B cell-like"))
```

In [64]:

```
# match the colors to the histo plot (Figure 1)
library(RColorBrewer)
cell.colors <- brewer.pal(8, "Set1")[c(1:5,7)]
```

In [65]:

```
library(RColorBrewer)

setPlotSize(5,5)
p <- TSNEPlot(b.cells, do.label = T, pt.size = 0.25, do.return = T, no.legend = T, 
              colors.use = cell.colors) +
        lims(y = c(0, 50), x = c(-60, -5))

svg("Figure_3A-sade_b_cell_phenotypes.svg", width = 5, height = 5)
print(p)
dev.off()

p
```

```
Warning message:
“Removed 1 rows containing missing values (geom_point).”
```

**png:** 2

```
Warning message:
“Removed 1 rows containing missing values (geom_point).”
```

## Signature expression¶

In [66]:

```
# repeat to take new annotations into account
avg.expression <- AverageExpression(b.cells)
```

```
Finished averaging RNA for cluster Activated B cell-like
Finished averaging RNA for cluster Germinal center B cell-like
Finished averaging RNA for cluster Memory B cell-like
Finished averaging RNA for cluster Plasma cell-like
Finished averaging RNA for cluster Plasmablast-like
Finished averaging RNA for cluster Naive B cell-like
```

### Supplementary Table 2¶

In [67]:

```
library(xlsx)
write.xlsx(avg.expression, file = "Supplementary_Table_2-Avg_Gene_Expression_Phenotypes.xlsx", sheetName = "Avg Expression")
```

In [86]:

```
avg.expression$gene <- rownames(avg.expression)
gene.plot.data <- melt(avg.expression, id.var = c("gene"), variable.name = "Cell.type", value.name = "Expression")
```

In [33]:

```
signatures <- list(
    "Activation" = c("CD69", "SELPLG", "CD72", "CFLAR", "FGFR1", "CD86"),
    "Co-Stimulation" = c("ICAM3", "TNFRSF13C", "CD40", "CD72", "C3",
                         "CD80", "CD86", "CD27", "CD28", "ICOS", "TNFRSF9",
                         "CD40LG", "ARHGDIB" ),
    "Immune-checkpoint" = c("CD274", "PDCD1LG2", "TNFRSF14", "LGALS9",
                            "BTLA", "LAG3", "HAVCR2", "ADORA2A"),
    "Exhaustion" = c("PDCD1", "FCRL4", "SIGLEC6", "CD22"),
    "Pro-Inflammation" = c("TNF", "IL12B", "IL18", "LTA", "TNFAIP2", "C3", "HCK"),
    "Cytokines-Immunsupp" = c("IL10", "TGFB1"))
```

In [74]:

```
library(GSVA)
sig.expr <- gsva(as.matrix(avg.expression[, 1:ncol(avg.expression)-1]), signatures, method="ssgsea", kcdf="Poisson")
```

```
Warning message in .local(expr, gset.idx.list, ...):
“4178genes with constant expression values throuhgout the samples.”
```

```
Estimating ssGSEA scores for 6 gene sets.
  |                                                                      |   0%Using parallel with 40 cores
  |======================================================================| 100%
```

### Figure 3B¶

In [75]:

```
sig.plot.data <- melt(sig.expr, varnames = c("Signature", "Cell.type"), value.name = "Expression")

setPlotSize(2,14)
p1 <- ggplot(sig.plot.data, aes(x = Cell.type, y = Expression, fill = Cell.type)) +
    geom_bar(stat = "identity", position = "dodge") +
    facet_wrap(~ Signature, scales = "free_y", ncol = 6) +
    scale_fill_manual(values = cell.colors) +
    theme(axis.text.x=element_blank(), strip.background = element_blank(), legend.position = "none") +
    labs(x = "Phenotype", fill = "Phenotype")

svg("Figure_3B-sade_signature_b_phenotypes.svg", width = 14, height = 2)
print(p1)
dev.off()

p1
```

**png:** 2

In [76]:

```
plotGenes <- function(genes, ncol = 4) {
    available.genes <- genes[genes %in% gene.plot.data$gene]
    
    plot.obj <- ggplot(gene.plot.data[gene.plot.data$gene %in% available.genes,], 
                       aes(x = Cell.type, y = Expression, fill = Cell.type)) +
        geom_bar(stat = "identity", position = "dodge") +
        facet_wrap(~gene, scales = "free_y", ncol = ncol) + 
        scale_fill_manual(values = cell.colors) +
        theme(axis.text.x = element_blank())
    
    return(plot.obj)
}

plotSignature <- function(n.sig) {
    sig.name <- names(signatures)[n.sig]
    nrows = ceiling(length(signatures[[sig.name]]) / 4)
    setPlotSize(2 * nrows, 10)
    plot.obj <- plotGenes(signatures[[sig.name]]) + labs(title = sig.name)
    print(plot.obj)
}
```

### Figure 3C¶

In [89]:

```
setPlotSize(5,6)

p1 <- plotGenes(c("CCL3", "CCL3L1", "CCL4", "CCL5", "CCL28", "CXCL16", "TGFB1", "IL10"), ncol = 2) + 
    theme(strip.background = element_blank()) +
    labs(x = "Phenotype", fill = "Phenotype")

svg("Figure_3C-sade_gene_expression.svg", width = 6, height = 5)
print(p1)
dev.off()

p1
```

**png:** 2

In [92]:

```
setPlotSize(4,10)
plotGenes(c("CSF1", "XCL1", "CCL22", "IL10", "TGFB1"))
```

### Signature gene plots¶

In [90]:

```
plotSignature(1)
```

In [91]:

```
plotSignature(2)
```

In [93]:

```
plotSignature(3)
```

In [94]:

```
plotSignature(4)
```

In [95]:

```
plotSignature(5)
```

In [96]:

```
plotSignature(6)
```

## B cell clinical correlation¶

In [97]:

```
b.cells@meta.data$response_time <- paste0(b.cells@meta.data$response, "-", b.cells@meta.data$timepoint)
b.cells.pre <- SubsetData(b.cells, subset.name = "timepoint", accept.value = "pre")
```

Combine the pre-sample data:

In [98]:

```
b.cells <- StashIdent(b.cells, "b.cell.ids")
tmp <- by(b.cells@meta.data, b.cells@meta.data$time_response, function(x) table(x$b.cell.ids, x$patient))

combined <- data.frame()

for (time.group in names(tmp)) {
    group.data <- data.frame(tmp[[time.group]])
    group.data$group <- time.group
    combined <- rbind(combined, group.data)
}

colnames(combined) <- c("Cell.type", "Pat", "Freq", "Group")
combined$Timepoint <- factor(gsub(".*-", "", combined$Group), levels = c("pre", "on"))
combined$Response <- gsub("-[^r].*", "", combined$Group)

head(combined)
```

| Cell.type | Pat | Freq | Group | Timepoint | Response |
| --- | --- | --- | --- | --- | --- |
| Activated B cell-like | 2 | 6 | Non-responder-on | on | Non-responder |
| Germinal center B cell-like | 2 | 0 | Non-responder-on | on | Non-responder |
| Memory B cell-like | 2 | 18 | Non-responder-on | on | Non-responder |
| Naive B cell-like | 2 | 9 | Non-responder-on | on | Non-responder |
| Plasmablast-like | 2 | 4 | Non-responder-on | on | Non-responder |
| Plasma cell-like | 2 | 13 | Non-responder-on | on | Non-responder |

### Figure 4D¶

In [99]:

```
# Compare the individual cell frequencies
setPlotSize(4,10)
p <- ggplot(combined, aes(x = Timepoint, y = log2(1+Freq), color = Response, fill = Response)) +
    geom_violin(alpha = 0.5, draw_quantiles = c(0.25, 0.5, 0.75)) +
    geom_jitter(height = 0, width = 0.2) +
    facet_wrap(~ Cell.type, scales = "free_y", ncol = 3) +
    theme(strip.background = element_blank()) +
    labs(y = "Cells per Patient (1 + log2)", color = "Outcome", fill = "Outcome")
    
svg("Figure_4D-sade_frequ_therapy.svg", width = 10, height = 4)
print(p)
dev.off()

p
```

**png:** 2

In [100]:

```
mean.cells <- by(combined$Freq, list(combined$Timepoint, combined$Response, combined$Cell.type), 
                 function(x) c("mean" = mean(x), "median" = median(x)))
mean.df <- data.frame()
for (time in dimnames(mean.cells)[[1]]) {
    for(resp in dimnames(mean.cells)[[2]]) {
        for (cell in dimnames(mean.cells)[[3]]) {
            mean.df <- rbind(mean.df, data.frame(
                timepoint = time,
                response = resp,
                cell.type = cell,
                mean.freq = mean.cells[[time, resp, cell]]["mean"],
                med.freq = mean.cells[[time, resp, cell]]["median"]
            ))
        }
    }
}

mean.df$timepoint <- factor(mean.df$timepoint, levels = c("pre", "on"))
setPlotSize(4,10)
ggplot(mean.df, aes(x = timepoint, y = med.freq, group = response, color = response)) +
    geom_line() +
    facet_wrap(~ cell.type, scales = "free_y", ncol = 3) +
    labs(y = "Median cells per patient") + 
                 theme(strip.background = element_blank()) + labs(x = "Timepoint", color = "Outcome")
```

### Subtype specific response statistics¶

Estimate the statistical significance for every B cell subtype that is enriched in responders pre-therapy:

In [101]:

```
combined.pre <- combined[combined$Timepoint == "pre"& !combined$Cell.type %in% c("Germinal center B cell-like", "Plasma cell-like"), ]
stat.res <- do.call("rbind", by(combined.pre, combined.pre$Cell.type, function(x) {
    response <- x$Freq[x$Response == "Responder"]
    non.resp <- x$Freq[x$Response == "Non-responder"]
    
    tes.res <- wilcox.test(response, non.resp)
    
    data.frame(
        p = tes.res$p.val,
        p.adj = ""
    )
}))

stat.res$p.adj <- p.adjust(stat.res$p, "BH")
stat.res <- round(stat.res, 2)
stat.res
```

```
Warning message in wilcox.test.default(response, non.resp):
“cannot compute exact p-value with ties”Warning message in wilcox.test.default(response, non.resp):
“cannot compute exact p-value with ties”Warning message in wilcox.test.default(response, non.resp):
“cannot compute exact p-value with ties”Warning message in wilcox.test.default(response, non.resp):
“cannot compute exact p-value with ties”
```

|  | p | p.adj |
| --- | --- | --- |
| Activated B cell-like | 0.14 | 0.14 |
| Memory B cell-like | 0.04 | 0.05 |
| Naive B cell-like | 0.01 | 0.04 |
| Plasmablast-like | 0.02 | 0.04 |

### B cell general distribution¶

Also assess the significance of the distribution of all B celles, irrespective of subtype:

In [104]:

```
# Total B cell number show no significant difference
combined.pre <- combined[combined$Timepoint == "pre",]
combined.pre$Pat <- droplevels(combined.pre$Pat)
total.b <- do.call("rbind", by(combined.pre, combined.pre$Pat, function(x) {
    data.frame(
        responder = sum(x$Freq[x$Response == "Responder"]),
        non.r = sum(x$Freq[x$Response == "Non-responder"]))
}))

non.r <- total.b$non.r[total.b$non.r > 0]
res <- total.b$responder[total.b$responder > 0]
```

In [106]:

```
setPlotSize(3,4)
boxplot(list(res = res, non.r = non.r))
wilcox.test(non.r, res)
```

```
Warning message in wilcox.test.default(non.r, res):
“cannot compute exact p-value with ties”
```

```
	Wilcoxon rank sum test with continuity correction

data:  non.r and res
W = 16.5, p-value = 0.1144
alternative hypothesis: true location shift is not equal to 0
```

## Environment¶

In [109]:

```
sessionInfo()
```

```
R version 3.4.4 (2018-03-15)
Platform: x86_64-pc-linux-gnu (64-bit)
Running under: Ubuntu 16.04.5 LTS

Matrix products: default
BLAS: /usr/lib/libblas/libblas.so.3.6.0
LAPACK: /usr/lib/lapack/liblapack.so.3.6.0

locale:
 [1] LC_CTYPE=en_US.UTF-8          LC_NUMERIC=C                 
 [3] LC_TIME=de_AT.UTF-8           LC_COLLATE=en_US.UTF-8       
 [5] LC_MONETARY=de_AT.UTF-8       LC_MESSAGES=en_US.UTF-8      
 [7] LC_PAPER=de_AT.UTF-8          LC_NAME=de_AT.UTF-8          
 [9] LC_ADDRESS=de_AT.UTF-8        LC_TELEPHONE=de_AT.UTF-8     
[11] LC_MEASUREMENT=de_AT.UTF-8    LC_IDENTIFICATION=de_AT.UTF-8

attached base packages:
[1] stats4    parallel  stats     graphics  grDevices utils     datasets 
[8] methods   base     

other attached packages:
 [1] GSVA_1.26.0          RColorBrewer_1.1-2   reshape2_1.4.3      
 [4] org.Hs.eg.db_3.5.0   AnnotationDbi_1.40.0 IRanges_2.12.0      
 [7] S4Vectors_0.16.0     Biobase_2.38.0       BiocGenerics_0.24.0 
[10] xlsx_0.6.1           bindrcpp_0.2.2       Seurat_2.3.4        
[13] Matrix_1.2-14        cowplot_0.9.3        ggplot2_3.0.0       
[16] jsonlite_1.5        

loaded via a namespace (and not attached):
  [1] uuid_0.1-2          snow_0.4-2          backports_1.1.2    
  [4] Hmisc_4.1-1         plyr_1.8.4          igraph_1.2.2       
  [7] repr_0.18           lazyeval_0.2.1      GSEABase_1.40.1    
 [10] splines_3.4.4       digest_0.6.15       foreach_1.4.4      
 [13] htmltools_0.3.6     lars_1.2            gdata_2.18.0       
 [16] magrittr_1.5        checkmate_1.8.5     memoise_1.1.0      
 [19] cluster_2.0.7-1     mixtools_1.1.0      ROCR_1.0-7         
 [22] annotate_1.56.2     R.utils_2.7.0       colorspace_1.3-2   
 [25] blob_1.1.1          dplyr_0.7.6         RCurl_1.95-4.11    
 [28] crayon_1.3.4        graph_1.56.0        bindr_0.1.1        
 [31] survival_2.42-6     zoo_1.8-4           iterators_1.0.10   
 [34] ape_5.2             glue_1.3.0          gtable_0.2.0       
 [37] kernlab_0.9-26      prabclus_2.2-6      DEoptimR_1.0-8     
 [40] scales_0.5.0        mvtnorm_1.0-8       DBI_1.0.0          
 [43] bibtex_0.4.2        Rcpp_0.12.18        metap_1.0          
 [46] dtw_1.20-1          xtable_1.8-2        htmlTable_1.12     
 [49] reticulate_1.10     foreign_0.8-71      bit_1.1-14         
 [52] proxy_0.4-22        mclust_5.4.1        SDMTools_1.1-221   
 [55] Formula_1.2-3       tsne_0.1-3          htmlwidgets_1.2    
 [58] httr_1.3.1          gplots_3.0.1        fpc_2.1-11.1       
 [61] acepack_1.4.1       modeltools_0.2-22   ica_1.0-2          
 [64] XML_3.98-1.13       pkgconfig_2.0.1     rJava_0.9-10       
 [67] R.methodsS3_1.7.1   flexmix_2.3-14      nnet_7.3-12        
 [70] tidyselect_0.2.4    labeling_0.3        rlang_0.2.1        
 [73] later_0.7.3         munsell_0.5.0       tools_3.4.4        
 [76] RSQLite_2.1.1       ggridges_0.5.1      evaluate_0.11      
 [79] stringr_1.3.1       npsurv_0.4-0        knitr_1.20         
 [82] bit64_0.9-7         fitdistrplus_1.0-11 robustbase_0.93-2  
 [85] caTools_1.17.1.1    purrr_0.2.5         RANN_2.6           
 [88] pbapply_1.3-4       nlme_3.1-137        mime_0.5           
 [91] R.oo_1.22.0         shinythemes_1.1.1   hdf5r_1.0.1        
 [94] compiler_3.4.4      rstudioapi_0.7      png_0.1-7          
 [97] lsei_1.2-0          geneplotter_1.56.0  tibble_1.4.2       
[100] stringi_1.2.4       lattice_0.20-38     trimcluster_0.1-2.1
[103] IRdisplay_0.7.0     pillar_1.3.0        Rdpack_0.10-1      
[106] lmtest_0.9-36       data.table_1.11.4   bitops_1.0-6       
[109] irlba_2.3.2         gbRd_0.4-11         httpuv_1.4.5       
[112] R6_2.2.2            latticeExtra_0.6-28 promises_1.0.1     
[115] KernSmooth_2.23-15  gridExtra_2.3       codetools_0.2-15   
[118] MASS_7.3-50         gtools_3.8.1        assertthat_0.2.0   
[121] xlsxjars_0.6.1      withr_2.1.2         diptest_0.75-7     
[124] doSNOW_1.0.16       grid_3.4.4          rpart_4.1-13       
[127] IRkernel_0.8.14     tidyr_0.8.1         class_7.3-14       
[130] segmented_0.5-3.0   Rtsne_0.15          pbdZMQ_0.3-3       
[133] shiny_1.1.0         base64enc_0.1-3
```
