# Supplementary material for: B cells sustain inflammation and predict response to immune checkpoint blockade in human melanoma
Source: Nat Commun. 2019 Sep 13;10:4186. doi: 10.1038/s41467-019-12160-2 (PMC6744450; doi:10.1038/s41467-019-12160-2)
Supplement: Supplementary file 8 — Supplementary Software 1 [file 41467_2019_12160_MOESM8_ESM.zip › 193518_1_other_0_psz9cz.zip!/Anti-CD20 analysis.html]

Anti-CD20 analysis


# Introduction¶

This is the analysis of the whole-tissue RNASeq data of the anti-CD20 samples.

The (clinical) results of both of these studies were published in Nat Commun 19 Sep 2017, 8(1):607 (Ofatumumab) and Mol Ther. 2012 May; 20(5): 1056–1062 (Rituximab).

In [1]:

```
# create plot style and save general options
library(ggplot2)
library(reshape2)
my_theme_bw <- theme_bw() +
    theme(strip.background = element_blank(),
          panel.border = element_rect(colour = "black"))

org.options <- options()

# add some convenience functions
setPlotSize <- function(height, width) {
    options(repr.plot.height=height, repr.plot.width = width)
}

resetPlotSize <- function() {
    options(org.options)
}

# if set to true, SVG plots are created in the "plots" subdirectory
createPlots <- F

if (!file.exists("plots")) {
    message("Created plots directory")
    dir.create("plots")
}

if (!file.exists("cache")) {
    message("Created cache directory")
    dir.create("cache")
}
```

# Load the data¶

In [2]:

```
library(edgeR)
library(org.Hs.eg.db)

load("data/anti_cd20_read_counts.RData")
colnames(read_counts_df) <- gsub(".*(P[0-9])", "\\1", colnames(read_counts_df))

# remove patient 5 since there are no matched pairs
read_counts_df <- read_counts_df[, colnames(read_counts_df) != "P5_after_OFA"]

# create patient data
samples <- data.frame(
    row.names = colnames(read_counts_df),
    patient = gsub("_.*", "", colnames(read_counts_df)),
    timepoint = gsub("[^_]*_([^_]*)_.*", " \\1", colnames(read_counts_df)),
    treatment = gsub(".*_", "", colnames(read_counts_df))
)

samples$timepoint <- gsub(" ", "", samples$timepoint)

# create gene data
genes <- data.frame(
    row.names = rownames(read_counts_df),
    gene = mapIds(org.Hs.eg.db, keys = rownames(read_counts_df), column = "SYMBOL", keytype = "ENSEMBL", multiVals = "first"),
    name = mapIds(org.Hs.eg.db, keys = rownames(read_counts_df), column = "GENENAME", keytype = "ENSEMBL", multiVals = "first")
)

# create the dge list object
dge <- DGEList(counts = read_counts_df, samples = samples, genes = genes)

dge$samples$treatment <- droplevels(dge$samples$treatment)
dge$samples$patient <- droplevels(dge$samples$patient)

dge$samples$treatment <- factor(dge$samples$treatment, levels = c("OFA", "RITUX"), labels = c("Ofatumumab", "Rituximab"))

saveRDS(dge, file = "cache/antiCD20.dge.rds")
```

```
Loading required package: limma
Loading required package: AnnotationDbi
Loading required package: stats4
Loading required package: BiocGenerics
Loading required package: parallel

Attaching package: ‘BiocGenerics’

The following objects are masked from ‘package:parallel’:

    clusterApply, clusterApplyLB, clusterCall, clusterEvalQ,
    clusterExport, clusterMap, parApply, parCapply, parLapply,
    parLapplyLB, parRapply, parSapply, parSapplyLB

The following object is masked from ‘package:limma’:

    plotMA

The following objects are masked from ‘package:stats’:

    IQR, mad, sd, var, xtabs

The following objects are masked from ‘package:base’:

    anyDuplicated, append, as.data.frame, basename, cbind, colMeans,
    colnames, colSums, dirname, do.call, duplicated, eval, evalq,
    Filter, Find, get, grep, grepl, intersect, is.unsorted, lapply,
    lengths, Map, mapply, match, mget, order, paste, pmax, pmax.int,
    pmin, pmin.int, Position, rank, rbind, Reduce, rowMeans, rownames,
    rowSums, sapply, setdiff, sort, table, tapply, union, unique,
    unsplit, which, which.max, which.min

Loading required package: Biobase
Welcome to Bioconductor

    Vignettes contain introductory material; view with
    'browseVignettes()'. To cite Bioconductor, see
    'citation("Biobase")', and for packages 'citation("pkgname")'.

Loading required package: IRanges
Loading required package: S4Vectors

Attaching package: ‘S4Vectors’

The following object is masked from ‘package:base’:

    expand.grid


'select()' returned 1:many mapping between keys and columns
'select()' returned 1:many mapping between keys and columns
```

Change to gene name and limit to genes expressed with at least 100 transcripts accross all patients:

In [3]:

```
dge <- readRDS("cache/antiCD20.dge.rds")

message("Original number of genes: ", nrow(dge))

# change to gene symbol
gene.expr <- rowSums(cpm(dge))
dge <- dge[order(gene.expr, decreasing = T), ]
dge <- dge[!is.na(dge$genes$gene) & !duplicated(dge$genes$gene), , keep.lib.sizes = F]
rownames(dge) <- dge$genes$gene

message("After change to gene symbol: ", nrow(dge))

# limit based on mean expression
gene.expr <- rowMeans(cpm(dge))
dge <- dge[gene.expr >= 0.01, , keep.lib.sizes = F]

message("After removing low-abundant genes: ", nrow(dge))

# calculate the normalization factors
dge <- calcNormFactors(dge, method = "TMM")
```

```
Original number of genes: 60239
After change to gene symbol: 24496
After removing low-abundant genes: 20030
```

In [4]:

```
dge$samples
```

|  | group | lib.size | norm.factors | patient | timepoint | treatment |
| --- | --- | --- | --- | --- | --- | --- |
| P1\_after\_OFA | 1 | 57379015 | 1.2239082 | P1 | after | Ofatumumab |
| P1\_before\_OFA | 1 | 52320532 | 0.8911709 | P1 | before | Ofatumumab |
| P2\_after\_OFA | 1 | 36735425 | 1.2060580 | P2 | after | Ofatumumab |
| P2\_before\_OFA | 1 | 31987827 | 0.9645985 | P2 | before | Ofatumumab |
| P4\_after\_OFA | 1 | 30931711 | 1.1901134 | P4 | after | Ofatumumab |
| P4\_before\_OFA | 1 | 32294272 | 1.1906710 | P4 | before | Ofatumumab |
| P6\_after\_OFA | 1 | 42319142 | 0.8243643 | P6 | after | Ofatumumab |
| P6\_before\_OFA | 1 | 37966373 | 0.9303491 | P6 | before | Ofatumumab |
| P7\_after\_RITUX | 1 | 45296566 | 0.6808742 | P7 | after | Rituximab |
| P7\_before\_RITUX | 1 | 82083180 | 1.1240526 | P7 | before | Rituximab |
| P8\_after\_RITUX | 1 | 43367038 | 0.8570228 | P8 | after | Rituximab |
| P8\_before\_RITUX | 1 | 47007414 | 1.1055649 | P8 | before | Rituximab |

# Dimensionality reduction¶

## MDS¶

In [5]:

```
options(repr.plot.height=4, repr.plot.width = 4)

plot.lbl <- paste0(dge$samples$patient, "_", ifelse(dge$samples$timepoint == "before", "B", "A"))

plotMDS(dge, 
        col = ifelse(dge$samples$treatment == "Rituximab", "red", "blue"),
        pch = ifelse(dge$samples$timepoint == "before", 15, 16),
        labels = plot.lbl)
```

## PCA¶

In [6]:

```
# limit to genes expressed in all pateints
exprs <- cpm(dge, prior.count=2, log=TRUE)

fit <- prcomp(exprs, center = T)

fit.summ <- summary(fit)
plot.xlab <- paste0("PC1 (", round(fit.summ[["importance"]]["Proportion of Variance", "PC1"] * 100, 1), "%)")
plot.ylab <- paste0("PC2 (", round(fit.summ[["importance"]]["Proportion of Variance", "PC2"] * 100, 1), "%)")

plot.data <- data.frame(fit$rotation)
plot.data <- cbind(plot.data, dge$samples)
plot.data$treatment <- factor(plot.data$treatment, levels = c("Ofatumumab", "Rituximab"),
                             labels = c("Therapeutic setting", "Adjuvant setting"))
plot.data$timepoint <- factor(plot.data$timepoint, levels = c("before", "after"), labels = c("Before", "On"))

setPlotSize(4,6)

plot.obj <- ggplot(plot.data, aes(x = PC1, y = PC2, color = treatment, shape = timepoint, group = patient)) + 
    geom_line() +
    geom_point(size = 3) +
    scale_color_brewer(palette = "Set2") +
    labs(x = plot.xlab, y = plot.ylab, shape = "Timepoint", color = "Treatment") +
    my_theme_bw

print(plot.obj)
svg("plots/Figure_5A.svg", width = 6, height = 4)
print(plot.obj)
dev.off()

ggplot(plot.data, aes(x = PC3, y = PC2, color = treatment, shape = timepoint, group = patient)) + 
    geom_line() +
    geom_point(size = 3) +
    scale_color_brewer(palette = "Set1") +
    labs(title = "PCA") +
    my_theme_bw

options(org.options)
```

**png:** 2

# Differential expression analysis¶

Differential expression analysis is performed using generalized linear models and the likelihood reatio test.

The model (design) takes the treatment timepoint and the patient into consideration. The actual treatment (Rituximab vs. Ofatumumab) is omitted since every patient belongs to a single treatment and no additional effect can be estimated for the treatment.

In [7]:

```
# create the design matrix
dge$samples$timepoint <- relevel(factor(dge$samples$timepoint), "before")
design <- model.matrix(~ dge$samples$timepoint + dge$samples$patient)

colnames(design)[2] <- "after"

# estimate the dispersion
dge <- estimateDisp(dge, design)
dge <- estimateGLMTagwiseDisp(dge, design)

# fit and get the DE genes for treatment
fit <- glmFit(dge, design)
treatment <- glmLRT(fit, coef = "after")

treatment.de <- topTags(treatment, n = Inf, p = 1)$table
```

In total, the following number of genes are differentially expressed at an FDR <= 0.05:

In [9]:

```
nrow(topTags(treatment, n = Inf, p = 0.05))
```

130

Top differentially expressed genes:

In [10]:

```
topTags(treatment, n = 20, p = 0.05)$table
```

|  | gene | name | logFC | logCPM | LR | PValue | FDR |
| --- | --- | --- | --- | --- | --- | --- | --- |
| DES | DES | desmin | 4.248755 | 2.7573590 | 56.12969 | 6.784478e-14 | 1.358931e-09 |
| JCHAIN | JCHAIN | joining chain of multimeric IgA and IgM | -2.395701 | 4.0112233 | 32.43295 | 1.233765e-08 | 8.561179e-05 |
| AWAT1 | AWAT1 | acyl-CoA wax alcohol acyltransferase 1 | 6.278016 | -2.3671901 | 32.03885 | 1.511201e-08 | 8.561179e-05 |
| KRTAP3-1 | KRTAP3-1 | keratin associated protein 3-1 | 9.400792 | 0.9420123 | 31.79915 | 1.709671e-08 | 8.561179e-05 |
| KRTAP1-3 | KRTAP1-3 | keratin associated protein 1-3 | 8.812245 | 0.3622035 | 29.32101 | 6.132729e-08 | 2.456771e-04 |
| MS4A1 | MS4A1 | membrane spanning 4-domains A1 | -4.756865 | 0.6909060 | 28.54364 | 9.161040e-08 | 3.058261e-04 |
| IRF7 | IRF7 | interferon regulatory factor 7 | -1.400752 | 5.7443578 | 25.76492 | 3.856350e-07 | 1.097377e-03 |
| ELF5 | ELF5 | E74 like ETS transcription factor 5 | 4.802495 | -2.2564178 | 25.51792 | 4.382932e-07 | 1.097377e-03 |
| KRTAP9-2 | KRTAP9-2 | keratin associated protein 9-2 | 5.847942 | -2.6442369 | 25.19650 | 5.177615e-07 | 1.152307e-03 |
| KRTAP4-9 | KRTAP4-9 | keratin associated protein 4-9 | 8.203211 | -0.1541509 | 24.87082 | 6.130324e-07 | 1.227904e-03 |
| AADACL3 | AADACL3 | arylacetamide deacetylase like 3 | 6.650349 | -1.8983463 | 23.74836 | 1.097889e-06 | 1.945764e-03 |
| SERPINE1 | SERPINE1 | serpin family E member 1 | -2.306439 | 5.8618355 | 23.63300 | 1.165710e-06 | 1.945764e-03 |
| SOCS3 | SOCS3 | suppressor of cytokine signaling 3 | -1.946903 | 6.0879874 | 23.33692 | 1.359650e-06 | 1.991595e-03 |
| BAAT | BAAT | bile acid-CoA:amino acid N-acyltransferase | 2.296777 | 3.2163883 | 23.18960 | 1.467890e-06 | 1.991595e-03 |
| KRTAP4-6 | KRTAP4-6 | keratin associated protein 4-6 | 7.859051 | -0.4114595 | 23.15897 | 1.491459e-06 | 1.991595e-03 |
| SBSPON | SBSPON | somatomedin B and thrombospondin type 1 domain containing | 2.846708 | 0.7076046 | 22.73148 | 1.862914e-06 | 2.332135e-03 |
| KRT35 | KRT35 | keratin 35 | 7.767022 | 0.5938583 | 22.45084 | 2.155911e-06 | 2.431102e-03 |
| MZB1 | MZB1 | marginal zone B and B1 cell specific protein | -2.537584 | 4.3464276 | 22.42535 | 2.184714e-06 | 2.431102e-03 |
| ADAMTS4 | ADAMTS4 | ADAM metallopeptidase with thrombospondin type 1 motif 4 | -1.572711 | 4.4176765 | 22.11869 | 2.563017e-06 | 2.701960e-03 |
| CD79A | CD79A | CD79a molecule | -3.036665 | 2.9776882 | 21.94181 | 2.810440e-06 | 2.814656e-03 |

## Analyze individual genes¶

Simply show the statstics for the main B-cell markers (CD19, CD20 = MS4A1), Plasmablast-like cell markers (CD27, CD38), and CD8+ T-cells (CD8A):

In [11]:

```
treatment.de[c("MS4A1", "CD19", "CD27", "CD38","CD8A"),]
```

|  | gene | name | logFC | logCPM | LR | PValue | FDR |
| --- | --- | --- | --- | --- | --- | --- | --- |
| MS4A1 | MS4A1 | membrane spanning 4-domains A1 | -4.756865 | 0.6909060 | 28.543637 | 9.161040e-08 | 0.0003058261 |
| CD19 | CD19 | CD19 molecule | -3.030096 | 0.1077703 | 15.225231 | 9.541999e-05 | 0.0248215905 |
| CD27 | CD27 | CD27 molecule | -1.294486 | 3.1744514 | 12.655029 | 3.745568e-04 | 0.0547618381 |
| CD38 | CD38 | CD38 molecule | -1.151295 | 2.0077791 | 6.600837 | 1.019309e-02 | 0.3283362639 |
| CD8A | CD8A | CD8a molecule | -1.385208 | 3.6771024 | 15.893142 | 6.702114e-05 | 0.0197416683 |

In [12]:

```
# Add a simple function to plot the abundance of genes
plotGenes <- function(genes) {
    library(ggplot2)
    library(reshape2)
    
    if (!all(genes %in% rownames(dge))) {
        stop("Unknown genes passed")
    }
    
    gene.expr <- cpm(dge[genes,])
    gene.expr <- melt(gene.expr)
    colnames(gene.expr) <- c("Gene", "Sample", "CPM")
    gene.expr <- merge(gene.expr, dge$samples, by.x = "Sample", by.y = 0)
    gene.expr$timepoint <- factor(gene.expr$timepoint, levels= c("before", "after"), labels = c("Before", "On"))
    
    gene.expr$Gene <- as.character(gene.expr$Gene)
    if ("MS4A1" %in% gene.expr$Gene) {
        gene.expr$Gene[gene.expr$Gene == "MS4A1"] <- "CD20 (MS4A1)"
    }
    
    plot.obj <- ggplot(gene.expr, aes(x = timepoint, y = log2(1 + CPM), color = treatment, group = patient, shape = treatment)) +
        geom_point() +
        geom_line() +
        scale_color_brewer(palette = "Set2") +
        facet_wrap(~ Gene, scales = "free_y", ncol = 2) +
        labs(x = "Timepoint", y = "log2(1 + CPM)", color = "Treatment", shape = "Treatment") +
        my_theme_bw
    
    return(plot.obj)
}


setPlotSize(5,4)

plot.obj <- plotGenes(c("CD19", "MS4A1", "CD8A", "XCL1", "CSF1", "CCL22", "TGFB1", "IL10"))

print(plot.obj)
svg("plots/Figure_5B_gene_expr.svg", width = 4, height = 5)
print(plot.obj)
dev.off()

resetPlotSize()
```

**png:** 2

# Analyze signatures¶

In [13]:

```
signatures <- list(
    "TIPB" = c("CD38", "CD27", "PAX5"),
    "Activation" = c("CD69", "SELPLG", "CD72", "CFLAR", "FGFR1", "CD86"),
    "Co-Stimulation" = c("ICAM3", "TNFRSF13C", "CD40", "CD72", "C3",
                         "CD80", "CD86", "CD27", "CD28", "ICOS", "TNFRSF9",
                         "CD40LG", "ARHGDIB" ),
    "Immune-checkpoint" = c("CD274", "PDCD1LG2", "TNFRSF14", "LGALS9",
                            "BTLA", "LAG3", "HAVCR2", "ADORA2A"),
    "Exhaustion" = c("PDCD1", "FCRL4", "SIGLEC6", "CD22"),
    "Pro-Inflammation" = c("TNF", "IL12B", "IL18", "LTA", "TNFAIP2", "C3", "HCK"),
    "Cytokines-Immunsupp" = c("IL10", "TGFB1"))
```

Estimate the abundance of our functional signatures using the `ssGSEA` algorithm:

In [16]:

```
library(GSVA)

sig.expr <- gsva(expr = as.matrix(cpm(dge, log = T, prior.count = 2)), 
                 signatures, 
                 kcdf="Gaussian", method="ssgsea")
saveRDS(sig.expr, file = "cache/ofa.sig.rds")
```

```
Estimating ssGSEA scores for 7 gene sets.
  |                                                                      |   0%Using parallel with 4 cores
  |======================================================================| 100%
```

## Test the correlation of the signatures with the phenotype¶

In [14]:

```
sig.expr <- readRDS("cache/ofa.sig.rds")

options(repr.plot.height=6, repr.plot.width = 8)
par(mfrow=c(2,3))
for (sig.index in 2:nrow(sig.expr)) {
    # get the correlation
    cor.res <- cor.test(sig.expr["TIPB", ], sig.expr[sig.index,],
                       method = "spearman", alternative = "two.sided")
    
    plot(sig.expr["TIPB", ], sig.expr[sig.index,],
        xlab = "TIPB",
        ylab = rownames(sig.expr)[sig.index],
        main = paste0("cor = ", round(cor.res$estimate, 2), 
                      " (p = ", round(cor.res$p.value, 3), ")"))
}

options(org.options)
```

## Correlate the signatures with treatment¶

In [15]:

```
plot.data <- melt(sig.expr)
colnames(plot.data) <- c("Signature", "Sample", "Expression")
plot.data <- merge(plot.data, dge$samples, by.x = "Sample", by.y = 0)
plot.data$timepoint <- factor(plot.data$timepoint, levels = c("before", "after"), labels = c("Before", "On"))
plot.data$treatment <- factor(plot.data$treatment,
                             levels = c("Ofatumumab", "Rituximab"),
                             labels = c("Therapeutic setting", "Adjuvant setting"))

options(repr.plot.height=3, repr.plot.width = 8)

plot.obj <- ggplot(plot.data, aes(x = timepoint, y = Expression, color = treatment, group = patient)) +
    geom_line() +
    facet_wrap(~ Signature, scales = "free_y", ncol = 4) +
    scale_color_brewer(palette = "Set2") +
    my_theme_bw +
    labs(color = "Treatment", x = "Timepoint")

print(plot.obj)

svg("plots/Supplementary_Figure_7A-anti_cd20_sig_corr.svg", width = 8, height = 3)
print(plot.obj)
dev.off()

options(org.options)
```

**png:** 2

Test whether the differences are statistically significant using a paired, one-sided t-test:

In [16]:

```
sig.diff <- do.call("rbind", apply(sig.expr, 1, function(expr.values) {
    before <- grepl("_before_", colnames(sig.expr))
    
    t.res <- t.test(expr.values[before], expr.values[!before], paired = T, alternative = "greater")
    
    data.frame(
        p = t.res$p.value,
        df = t.res$parameter["df"],
        t = t.res$statistic["t"]
    )
}))

# correct for multiple testing
sig.diff$p.adj <- p.adjust(sig.diff$p, "BH")
sig.diff <- round(sig.diff, 2)

sig.diff
```

|  | p | df | t | p.adj |
| --- | --- | --- | --- | --- |
| TIPB | 0.00 | 5 | 4.92 | 0.01 |
| Activation | 0.01 | 5 | 3.82 | 0.01 |
| Co-Stimulation | 0.01 | 5 | 3.45 | 0.02 |
| Immune-checkpoint | 0.02 | 5 | 2.76 | 0.02 |
| Exhaustion | 0.02 | 5 | 2.85 | 0.02 |
| Pro-Inflammation | 0.04 | 5 | 2.28 | 0.04 |
| Cytokines-Immunsupp | 0.00 | 5 | 4.79 | 0.01 |

Based on this analysis, all signatures show a signficiant down-regulation upon treatment with anti-CD20 antibodies.

# Test published signatures¶

Test how published signatures that represent the inflammatory TME change after anti-CD20 therapy.

In [20]:

```
published.sig <- list(
    # TIS: 10.1186/s40425-018-0367-1
    "TIS" = c("PSMB10", "HLA-DQA1", "HLA-DRB1", "CMKLR1", "HLA-E", "NKG7", "CD8A", "CCL5", "CXCL9", "CD27", "CXCR6", "IDO1", "STAT1", "TIGIT", "LAG3", "CD274", "PDCD1LG2", "CD276"),
    # Taken from doi.org/10.1172/JCI91190
    "IFN-Gamma" = c("IFNG", "STAT1", "CCR5", "CXCL9", "CXCL10", "CXCL11", "IDO1", "PRF1", "GZMA", "MHCII", "HLA-DRA"),
    # T-cell exhaustion: Immunity 2007; 27: 670–684, doi: 10.3389/fimmu.2018.01786 
    "T-cell_Exhaustion" = c("HAVCR2", "CD38", "CD101", "CD244", "TIGIT"),
    # 10.1182/bloodadvances.2016000786.
    "T-Effector" = c("GZMA", "GZMB", "PRF1", "IFNG", "EOMES", "CD8A"),
    "CD4/FOXP3" = c("FOXP3", "CD4")
)

pubslished.sig.expr <- gsva(expr = as.matrix(cpm(dge, log = T, prior.count = 2)), published.sig, kcdf="Gaussian", method="ssgsea")
```

```
Estimating ssGSEA scores for 5 gene sets.
  |                                                                      |   0%Using parallel with 4 cores
  |======================================================================| 100%
```

## Test correlation with phenotype¶

In [21]:

```
setPlotSize(3, 10)

if (createPlots) {
    svg("plots/Supplementary_Figure_7B-anti_cd20_pub_sig.svg", width=10, height = 3)
}
par(mfrow=c(1, 4))
for (sig.index in 1:(nrow(pubslished.sig.expr)-1)) {
    # get the correlation
    cor.res <- cor.test(sig.expr["TIPB", ], 
                        pubslished.sig.expr[sig.index,],
                       method = "spearman", alternative = "two.sided")
    
    if (round(cor.res$p.value, 2) < 0.01) {
        p.text <- " (p < 0.01)"
    } else {        
        p.text <- paste0(" (p = ", round(cor.res$p.value, 2), ")")
    }
    
    plot(sig.expr["TIPB", ], pubslished.sig.expr[sig.index,],
        xlab = "TIPB",
        ylab = rownames(pubslished.sig.expr)[sig.index],
        main = paste0("cor = ", round(cor.res$estimate, 2), 
                      p.text))
}
dev.off()
```

**null device:** 1

## Correlate with treatment¶

In [22]:

```
pub.sig.diff <- do.call("rbind", apply(pubslished.sig.expr[1:4,], 1, function(expr.values) {
    before <- grepl("_before_", colnames(sig.expr))
    
    t.res <- t.test(expr.values[before], expr.values[!before], paired = T, alternative = "greater")
    
    data.frame(
        p = t.res$p.value,
        df = t.res$parameter["df"],
        t = t.res$statistic["t"]
    )
}))

# correct for multiple testing
pub.sig.diff$p.adj <- p.adjust(pub.sig.diff$p, "BH")

pub.sig.diff <- round(pub.sig.diff, 2)

pub.sig.diff
```

|  | p | df | t | p.adj |
| --- | --- | --- | --- | --- |
| TIS | 0.00 | 5 | 5.46 | 0.01 |
| IFN-Gamma | 0.01 | 5 | 3.11 | 0.01 |
| T-cell\_Exhaustion | 0.01 | 5 | 3.19 | 0.01 |
| T-Effector | 0.01 | 5 | 3.25 | 0.01 |

In [23]:

```
plot.data <- melt(pubslished.sig.expr)
colnames(plot.data) <- c("Signature", "Sample", "Expression")
plot.data <- merge(plot.data, dge$samples, by.x = "Sample", by.y = 0)

plot.data$Signature <- as.character(plot.data$Signature)
plot.data$Signature[plot.data$Signature == "T-cell_Exhaustion"] <- "T-cell exhaustion"
plot.data$timepoint <- factor(plot.data$timepoint, levels = c("before", "after"), labels = c("Before", "On"))

plot.data.foxp3 <- plot.data[plot.data$Signature == "CD4/FOXP3",]

plot.data <- plot.data[!plot.data$Signature == "CD4/FOXP3",]

setPlotSize(2,8)

plot.obj <- ggplot(plot.data, aes(x = timepoint, y = Expression, color = treatment, group = patient)) +
    geom_line() +
    facet_wrap(~ Signature, scales = "free_y", ncol = 5) +
    scale_color_brewer(palette = "Set2") +
    my_theme_bw +
    labs(color = "Treatment", x = "Timepoint", y = "Expression (ssGSEA)")

print(plot.obj)
svg("plots/Figure_5C.svg", width = 8, height = 2)
print(plot.obj)
dev.off()

options(org.options)
```

**png:** 2

# Analyze cell type abundance¶

Cell type abundance is estimated using xCell:

In [24]:

```
library(xCell)

# run xCell
xcell.exprs <- xCellAnalysis(cpm(dge, log = T, prior.count = 2), rnaseq = T)
```

```
[1] "Num. of genes: 10293"
Estimating ssGSEA scores for 489 gene sets.
  |                                                                      |   0%Using parallel with 4 cores
  |======================================================================| 100%
```

## Cell type changes on therapy¶

In [25]:

```
colnames(plot.data.foxp3)[2] <- "Cell.type"
plot.data.foxp3$Cell.type <- "CD4/FOXP3 (ssGSEA)"

plot.data <- melt(xcell.exprs)
colnames(plot.data) <- c("Cell.type", "Sample", "Expression")
plot.data <- merge(plot.data, dge$samples, by.x = "Sample", by.y = 0)

plot.data <- plot.data[plot.data$Cell.type %in% 
                       c("CD8+ T-cells", "Macrophages"), ]

plot.data$timepoint <- factor(plot.data$timepoint, levels = c("before", "after"), labels = c("Before", "On"))

plot.data <- rbind(plot.data, plot.data.foxp3)

setPlotSize(5, 3.5)

plot.obj <- ggplot(plot.data, aes(x = timepoint, y = Expression, color = treatment, group = patient)) +
    geom_line() +
    facet_wrap(~ Cell.type, scales = "free_y", ncol = 1)+
    scale_color_brewer(palette = "Set2")+
    my_theme_bw +
    labs(color = "Treatment", x = "Timepoint", y = "Est. abundance")

print(plot.obj)
svg("plots/Figure_5D.svg", width = 3.5, height = 5)
print(plot.obj)
dev.off()

resetPlotSize()
```

**png:** 2

Apparently, anti-CD20 therapy is dramatically altering the composition of the tumor microenvironment in all patients.

In [26]:

```
is.before <- dge$samples$timepoint == "before"

all.ps <- do.call("rbind", apply(xcell.exprs[c("CD8+ T-cells", "Macrophages"),], 1, function(cell.expr) {
    
    t.res <- t.test(cell.expr[is.before], cell.expr[!is.before], alternative = "greater", paired = T)
    
    data.frame(
        p = t.res$p.value,
        df = t.res$parameter["df"],
        t = t.res$statistic["t"]
    )
}))

all.ps <- all.ps[order(all.ps$p), ]
all.ps$p.adj <- p.adjust(all.ps$p, "BH")
all.ps <- round(all.ps, 2)
all.ps
```

|  | p | df | t | p.adj |
| --- | --- | --- | --- | --- |
| Macrophages | 0.04 | 5 | 2.17 | 0.07 |
| CD8+ T-cells | 0.07 | 5 | 1.74 | 0.07 |

# Supplementary Table 2¶

In [27]:

```
library(xlsx)
write.xlsx(treatment.de[round(treatment.de$FDR, 2) <= 0.05,], file = "plots/Supplementary_Table_2-Anti_CD20.xlsx", sheetName = "RNASeq (<= 5% FDR)")
```

# Package versions¶

In [28]:

```
sessionInfo()
```

```
R version 3.5.1 (2018-07-02)
Platform: x86_64-pc-linux-gnu (64-bit)
Running under: Ubuntu 18.04.1 LTS

Matrix products: default
BLAS: /usr/lib/x86_64-linux-gnu/blas/libblas.so.3.7.1
LAPACK: /usr/lib/x86_64-linux-gnu/lapack/liblapack.so.3.7.1

locale:
 [1] LC_CTYPE=en_US.UTF-8          LC_NUMERIC=C                 
 [3] LC_TIME=de_AT.UTF-8           LC_COLLATE=en_US.UTF-8       
 [5] LC_MONETARY=de_AT.UTF-8       LC_MESSAGES=en_US.UTF-8      
 [7] LC_PAPER=de_AT.UTF-8          LC_NAME=de_AT.UTF-8          
 [9] LC_ADDRESS=de_AT.UTF-8        LC_TELEPHONE=de_AT.UTF-8     
[11] LC_MEASUREMENT=de_AT.UTF-8    LC_IDENTIFICATION=de_AT.UTF-8

attached base packages:
[1] parallel  stats4    stats     graphics  grDevices utils     datasets 
[8] methods   base     

other attached packages:
 [1] xlsx_0.6.1           xCell_1.1.0          GSVA_1.28.0         
 [4] org.Hs.eg.db_3.6.0   AnnotationDbi_1.42.1 IRanges_2.14.12     
 [7] S4Vectors_0.18.3     Biobase_2.40.0       BiocGenerics_0.26.0 
[10] edgeR_3.22.5         limma_3.36.5         reshape2_1.4.3      
[13] ggplot2_3.1.0       

loaded via a namespace (and not attached):
 [1] bit64_0.9-7          jsonlite_1.5         splines_3.5.1       
 [4] shiny_1.1.0          assertthat_0.2.0     getPass_0.2-2       
 [7] xlsxjars_0.6.1       blob_1.1.1           pillar_1.3.0        
[10] RSQLite_2.1.1        lattice_0.20-35      quadprog_1.5-5      
[13] glue_1.3.0           uuid_0.1-2           digest_0.6.18       
[16] RColorBrewer_1.1-2   promises_1.0.1       colorspace_1.3-2    
[19] htmltools_0.3.6      httpuv_1.4.5         plyr_1.8.4          
[22] GSEABase_1.42.0      XML_3.98-1.16        pkgconfig_2.0.2     
[25] purrr_0.2.5          xtable_1.8-3         scales_1.0.0        
[28] later_0.7.5          pracma_2.1.8         tibble_1.4.2        
[31] annotate_1.58.0      withr_2.1.2          repr_0.15.0         
[34] lazyeval_0.2.1       magrittr_1.5         crayon_1.3.4        
[37] mime_0.6             memoise_1.1.0        evaluate_0.12       
[40] MASS_7.3-51          graph_1.58.2         tools_3.5.1         
[43] stringr_1.3.1        munsell_0.5.0        locfit_1.5-9.1      
[46] bindrcpp_0.2.2       compiler_3.5.1       rlang_0.3.0.1       
[49] grid_3.5.1           RCurl_1.95-4.11      pbdZMQ_0.3-3        
[52] IRkernel_0.8.12.9000 bitops_1.0-6         base64enc_0.1-3     
[55] labeling_0.3         gtable_0.2.0         DBI_1.0.0           
[58] R6_2.3.0             dplyr_0.7.7          bit_1.1-14          
[61] bindr_0.1.1          shinythemes_1.1.1    rJava_0.9-10        
[64] stringi_1.2.4        IRdisplay_0.6        Rcpp_0.12.19        
[67] geneplotter_1.58.0   tidyselect_0.2.5
```
